# The diagnostic utility of DNA copy number analysis of core needle biopsies from soft tissue and bone tumors

**DOI:** 10.1038/s41374-022-00770-2

**Published:** 2022-03-22

**Authors:** Jan Köster, Paul Piccinelli, Linda Arvidsson, Fredrik Vult von Steyern, Camila Bedeschi Rego De Mattos, Martin Almquist, Jenny Nilsson, Linda Magnusson, Fredrik Mertens

**Affiliations:** 1 Division of Clinical Genetics, Department of Laboratory Medicine, Lund University, Lund, Sweden; 2 Department of Clinical Genetics and Pathology, Division of Laboratory Medicine, Lund, Sweden; 3 Department of Orthopedics, Department of Clinical Sciences Lund, Skåne University Hospital, Lund, Sweden; 4 Department of Surgery, Skåne University Hospital, Lund, Sweden

**Keywords:** Cancer genomics, Cancer genomics

## Abstract

Morphologic and immunohistochemical analysis of preoperative core needle biopsies (CNB) is important in the management of patients with soft tissue and bone tumors (STBTs). Most SBTB subtypes have more or less extensive DNA copy number aberrations (CNA), potentially providing useful diagnostic information. To evaluate the technical feasibility of single nucleotide polymorphism (SNP) array analysis and the diagnostic usefulness of the copy number profiles, we studied CNBs from 171 patients with suspected STBTs. SNP array analysis could be performed on 168 (98%) of the samples. The CNA profile was compatible with the CNB diagnosis in 87% of the cases. Discrepant cases were dominated by false-negative results due to nonrepresentative material or contamination with normal cells. 70 genomic profiles were indicative of specific histopathologic tumor entities and in agreement with the corresponding CNB diagnoses in 83%. In 96 of the cases with aberrant CNA profiles, the SNP profiles were of sufficient quality for segmentation, allowing clustering analysis on the basis of the Jaccard similarity index. The analysis of these segment files showed three major CNA clusters, based on the complexity levels and the predominance of gains versus losses. For 43 of these CNB samples, we had SNP array data also from their corresponding surgical samples. In 33 of these pairs, the two corresponding samples clustered next to each other, with Jaccard scores ranging from 0.61 to 0.99 (median 0.96). Also, for those tumor pairs that did not cluster together, the Jaccard scores were relatively high (median 0.9). 10 cases showed discrepant results, mainly due to varying degrees of normal cell contamination or technical issues. Thus, the copy number profile seen in a CNB is typically highly representative of the major cell population in the tumor.

## Abstract (web summary)

In this study, the technical feasibility and the diagnostic value of SNP array analysis on 171 core needle biopsies (CNB) from soft tissue and bone tumors was evaluated. The analysis succeeded technically in 98% of the cases. The copy number profiles were compatible with the CNB diagnoses in 87% of the cases and was in 77% of the cases representative for the whole lesion.

## Introduction

Soft tissue and bone tumors (STBTs) comprise a heterogeneous group of neoplasms with benign entities outnumbering malignant tumors (sarcomas) by far. Main elements within the diagnostic chain of STBTs are the clinical setting and radiological picture, as well as tissue sampling with subsequent morphologic analysis. Cyto- and histopathologic analyses of mesenchymal tumors can be challenging because morphologic features may be shared by several mesenchymal and non-mesenchymal neoplasms and because of intratumoral morphologic heterogeneity. Furthermore, immunohistochemical (IHC) profiles can be inconsistent. In the diagnostic process, morphologic/IHC approaches are thus often complemented with genetic analyses.

The morphologic differences among STBTs are reflected by an extensive genetic variation. Some tumors are driven by gene fusions, while some develop through single nucleotide variants (SNVs) or copy number aberrations (CNAs); often, tumors show a combination of these features. Some of the mutations, especially gene fusions, are strongly associated with specific morphologic subtypes, and a subset of them, or even their epigenetic consequences, have guided the development of IHC stains as surrogate markers for the underlying aberration^1^.

While the diagnostic relevance of gene fusion detection in the management of STBT patients is undisputed, it should be pointed out that most morphologic entities lack such biomarkers. Instead, those tumors tend to display mutation profiles dominated by CNAs, a class of mutation that has been less extensively explored as a diagnostic tool; evaluation of the copy number level of *MDM2* in the differential diagnosis of well- and dedifferentiated liposarcomas is currently the only exception in routine clinical practice^2^. However, it is well known that also types and patterns of other CNAs show strong association with tumor type^2^, and the identification of such copy number patterns is thus of potential differential diagnostic interest. Chromosome banding is, in theory, ideal for identifying both balanced and unbalanced chromosomal rearrangements, but it has been shown that this method is suboptimal for pre-treatment samples like fine needle aspirates and core needle biopsies (CNBs)^3^. Genome-wide copy number variation can, however, be examined by other methods, such as whole-genome sequencing (WGS), but this remains an expensive option. Another possibility is to use genomic arrays, including single nucleotide polymorphism (SNP) arrays, that provide genome-wide information on CNAs and allelic imbalances.

Here, we present a series of 171 patients with soft tissue or bone lesions that were analyzed with high-resolution SNP arrays. The aims of the study were: (i) to examine the technical feasibility of SNP array analysis on CNB material in a clinical setting, (ii) to evaluate the representativeness of the genomic profiles of CNBs by comparing with SNP array data on the surgical specimen from the same tumor, and (iii) to evaluate the diagnostic impact of the genomic profile.

## Materials and methods

### Patients and tumors

The study included 171 patients, diagnosed with a soft tissue (*n* = 148) or bone (*n* = 23) condition at the University Hospital in Lund, Sweden between 2016 and 2019. Three to six CNBs (18 to 14 gauge needles) were taken per lesion; of those, one or two were sent for genetic analysis and the remaining CNBs were forwarded for routine microscopic examination. CNBs and samples from resected tumors were sent for SNP array analysis in culture medium and saline solution, respectively, and were stored in a refrigerator until DNA extraction within 72 h for CNBs and stored at −20 °C in a freezer for maximum of 7 days before DNA extraction for resected tumors after sampling. During the procedure, the representativity of the obtained CNB material was in most cases cytologically checked on Diff-Quick stains of imprint material. Samples from surgical specimens were obtained from tumor parts that macroscopically looked representative and viable but were not cytologically checked for representativeness. All cases were primary lesions except six local relapses and two metastases. Of all lesions 126 (73%) were located in the deep soft tissue or bone and 45 (27%) were located superficially (skin or subcutaneous fatty tissue). Of all 171 patients, 94 (55%) underwent surgical treatment. All diagnoses on both CNB and surgical material were based on established criteria^2^.

The decision to perform SNP array analysis, rather than other genetic analyses, on the CNBs was based on the clinical information (patient age, tumor location, duration of growth, radiologic features, preliminary suggestion from the cytopathologist) accompanying the sample. In general, SNP array was the method of choice whenever a malignant lesion was among the differential diagnoses, except for when a translocation-associated sarcoma was suspected; most such tumors were instead prioritized for FISH, G-banding, and/or RNA-sequencing. The clinical context was crucial also for the interpretation of the SNP array results.

### SNP array analysis

SNP array analysis was attempted on the CNBs in all cases; from 62 (36%) of them, we had the possibility to analyze also the resection specimens. DNA was extracted from fresh tumor samples using the QIAamp Fast DNA Tissue kit, following the manufacturer’s instructions (Qiagen, Hilden, Germany). Quality and concentration of the extracted material were measured with a 2100 Bioanalyzer (Agilent Technologies, Santa Clara, CA) and a NanoDrop ND-1000 (Thermo Fisher Scientific, Waltham, MA). Tumor DNA (60–250 ng) was then genotyped on Affymetrix CytoScan HD arrays, containing more than 2.6 million markers (Affymetrix, Santa Clara, USA). The position of the probes was aligned according to the UCSC hg19/NCBI Build 37 sequences. Genomic aberrations were identified by visual inspection using the Chromosome Analysis Suite (ChAS) version 4.0.0.385 (Affymetrix); the clinical report was based on the visual inspection in ChAS. In addition, for 96 patients where the genomic profile was abnormal and the data were of sufficient quality, the Tumor Aberration Prediction Suite (TAPS) and Rawcopy were used for segmentation of copy number data, copy number evaluation, and visualization of the results^4,5^. In the visual analysis of SNP array data, constitutional copy number variations (CNVs) were excluded through comparison with the Database of Genomic Variants (http://dgv.tcag.ca/dgv/app/home). Matching segmentation profiles from both types of specimen were available from 43 of the patients.

### Case clustering and data visualization

Case clustering was performed on CNBs and surgical samples with segmented CNA data. Only autosomal copy number changes were considered, and in order to reduce the impact of constitutional CNVs, as well as technical noise, only segments ≥100 kb with ≥50 probes were included for further analysis. Thus, a total of 86 CNB samples and 52 surgical samples could be analyzed. Hierarchical clustering of the cases was performed according to the Jaccard similarity coefficient. The Jaccard index was used to measure the similarity among lesions based on the overlap of their CNAs and refers to the ratio of the number of overlapping base pairs between two samples and the length of the union. The index can range from 0 to 1, where 1 represents complete overlap. The hclust function in the basic statistic package of R (R version 3.6.0, https://www.r-project.org) was used for calculating the Jaccard index, as well as for Wilcoxon tests. Heatmaps of the copy number segments along the genome were plotted using the R-package Cellscape (Cellscape version 1.8.0, https://rdrr.io/bioc/cellscape/man/cellscape.html). The R-packages Easyalluvial and ggplot2 were used for alluvial plots and boxplots, respectively (Easyalluvial version 0.3.0, https://rdrr.io/cran/easyalluvial; ggplot2 version 3.3.3, https://cran.r-project.org/web/packages/ggplot2/index.html).

## Results

### Technical feasibility of SNP array analysis

DNA of sufficient quantity and quality for SNP array analysis could be extracted from 168/171 (98%) CNBs and all 62 surgical samples. The three failures were due to degraded DNA in two (suspected well-differentiated liposarcomas) and insufficient amount of DNA in one (normal bone with inflammatory changes). The following text will only discuss the 168 successfully analyzed cases. Rawcopy images of cases with available segmentation files can be found in Supplementary Fig. 1.

### Tumor cohort

Based on the CNB diagnoses, 58 of the cases were benign neoplasms, the most frequent being desmoid fibromatosis (*n* = 8), lipoma (*n* = 7), neurofibroma (*n* = 5), and schwannoma (*n* = 5). 85 lesions were classified as malignant, with undifferentiated pleomorphic sarcoma (UPS; *n* = 20), dedifferentiated liposarcoma (DDLS; *n* = 8), and leiomyosarcoma (LMS; *n* = 6) as the most common diagnoses. Six tumors were diagnosed as neoplasms of unknown malignant potential (UMP) and 19 cases revealed nonneoplastic tissue or insufficient material. Information on the CNB diagnoses, as well as the final diagnoses based on examination of surgical specimens and/or additional ancillary data, can be found in Supplementary Table 1.

The SNP array and the morphologic analyses of the CNBs were performed independently in parallel, resulting in two separate clinical reports. Discrepant results were discussed at the weekly sarcoma conferences. SNP array analyses without CNAs were reported as providing no support for a neoplastic lesion, with the caveats that the sample might be nonrepresentative or that the lesion might be a neoplasm without CNAs. For the 95 CNBs with CNAs, the report stated that the finding strongly indicated a neoplastic disease and, depending on the nature of the CNAs, that they were indicative of a certain type of neoplasia or group of neoplasms (Supplementary Table 1).

Because clinical decisions to a large extent are based on the results from the analyses of the CNBs, the summary below focuses on the correspondence, or lack thereof, between the morphological and genetic features of the CNBs.

### Morphologic subgroups among CNBs

#### Lesions diagnosed as nonneoplastic and CNBs considered nonrepresentative

19 CNB specimens showed morphologically nonneoplastic or nonrepresentative tissue; three of these had abnormal SNP array profiles. Case 3, morphologically displaying insufficient material, had uniparental isodisomy for all chromosomes except chromosomes 5, 18, 20, 21, and 22 at SNP array analysis. The patient underwent surgical treatment with inflammatory leiomyosarcoma as the final diagnosis. SNP array analysis on the resection specimen showed an identical genomic profile. Case 43, which morphologically looked like regular fatty tissue, had a deletion in 13q14 as the sole genomic change. The lesion has not been resected. Case 165, with only necrotic material in the CNB material, but diagnosed as pleomorphic sarcoma on the cytologic material, had an abnormal SNP profile with complex structural aberrations involving the majority of chromosomes. Of the 16 cases with normal SNP array profiles, five underwent surgical treatment; three of those were diagnosed as neoplasms (one each of meningioma, inflammatory myofibroblastic tumor, and mesenterial lipoma).

#### Neoplasms diagnosed as benign

CNAs were found in 16/58 (28%) CNBs diagnosed as benign neoplasms. Based on the SNP array data, all of the cases were interpreted to have a near-diploid chromosome number. The observed changes were typically few (median, 1.5), ranging from single segments showing loss of heterozygosity (LOH), deletions or duplications to up to 9 CNAs affecting parts of or entire chromosomes.

Among the more common diagnostic entities, 3/8 desmoid fibromatoses showed abnormal SNP array profiles: two (Cases 47 and 75) had deletions in 6q and one (Case 151) had trisomy 20. None of the patients with lesions diagnosed as desmoid fibromatosis underwent surgical treatment. Only 1/7 lipomas showed an abnormal SNP array profile, with a deletion in chromosome band 13q14 as the sole change (Case 52). Three of the tumors were surgically removed, but SNP array analysis was not performed on the resected specimens. None of the five neurofibromas had any CNA, but two samples (Cases 99 and 166) from patients with neurofibromatosis type 1 showed LOH at the *NF1* locus, possibly representing the somatic event in the functional inactivation of *NF1*. Two of the patients underwent surgical treatment, and renewed SNP array analysis was performed on one of them (Case 99), now also showing a deletion of *CDKN2A* in 9p and a deletion in Xq. Finally, 3/5 schwannomas had CNAs. One (Case 81) had deletions in 13q and 17q, one (Case 105) had a duplication in 5q, a homozygous deletion of *CDKN2A*, and LOH at the *NF1* locus, and one (Case 107) showed LOH in 22q as the sole change. Two of the patients underwent surgical treatment and renewed SNP array analysis. In the resection specimen of Case 81 monosomy 21 was the sole change, and Case 107 showed the same LOH in 22q as in the CNB.

#### Neoplasms diagnosed as UMP

Four of the six cases diagnosed as UMP were described as (atypical) spindle cell tumor of unknown type. The two remaining cases were myxoid tumors, not otherwise specified (NOS). Four cases showed CNAs. Two had only a few aberrations, with trisomy 2 and a deletion in 10q as the sole changes in one (Case 66) and a deletion in 9q as the only change in the other (Case 89). Case 51 showed multiple structural changes involving chromosomes 1, 4, 6, 9, 13, and 17. One of the myxoid tumors (Case 168) had a more complex profile, including loss and duplication in 6q, gains in 7q and 9q, losses in 10p and 13q, and monosomy 21. The tumors of three of the patients (Cases 51, 66 and 89) were surgically resected with recurrent atypical spindle cell neoplasm of UMP (Case 51), spindle cell sarcoma NOS (Case 66) and low-grade fibromyxoid sarcoma (LGFMS) (Case 89) as the final diagnoses. SNP array analysis was performed on all three cases and confirmed the genomic profiles found in the CNB material.

#### Neoplasms diagnosed as malignant

Aberrant SNP array profiles were found in 72/85 (85%) cases diagnosed as malignant. In the most common entity (UPS), 17/20 cases had abnormal SNP array profiles; all cases with CNAs showed aneuploidy and complex chromosomal imbalances involving the majority of the chromosomes. Due to the complex copy number shifts, the exact ploidy levels were difficult to determine visually, but were interpreted to range from hypo-diploid to hyper-pentaploid. All patients except one underwent surgical treatment, and in 7/20 cases the final diagnosis was changed to myxofibrosarcoma (MFS; Cases 5, 27, 34, and 134), pleomorphic liposarcoma (Cases 61 and 68) or dedifferentiated chondrosarcoma (Case 158). SNP array analysis on surgical material from the three patients without CNAs in the CNB (Cases 22, 158, and 169) showed aneuploidy and complex structural aberrations engaging all chromosomes.

Eight cases were diagnosed as DDLS, all of which showed CNAs, including amplicons in 12q (always including the *MDM2* and *CDK4* loci). Six of these tumors showed additional aberrations supporting the diagnosis: amplification of *JUN* in 1p and/or genome-wide copy number changes. Atypical lipomatous tumor (ALT)/well-differentiated liposarcoma (WDLS) was diagnosed on CNB in five cases (Cases 38, 50, 70, 114, and 120). Three of these had amplicons in 12q, always including *MDM2* and in one also *CDK4*, one failed, and one (Case 114) had a 5 Mb deletion in 1p as the sole aberration.

All six leiomyosarcomas had abnormal SNP array profiles with aneuploidy and often complex copy number shifts. Deletions affecting the *DMD* gene were seen in two cases, and deletion of or copy number change in *ATRX* and *TP53* in one case each. One case showed amplification of *MYOCD* and *KIT*.

### Genetic subgroups among CNBs

#### Copy number profiles compatible with CNB diagnoses

**Fig. 1:**
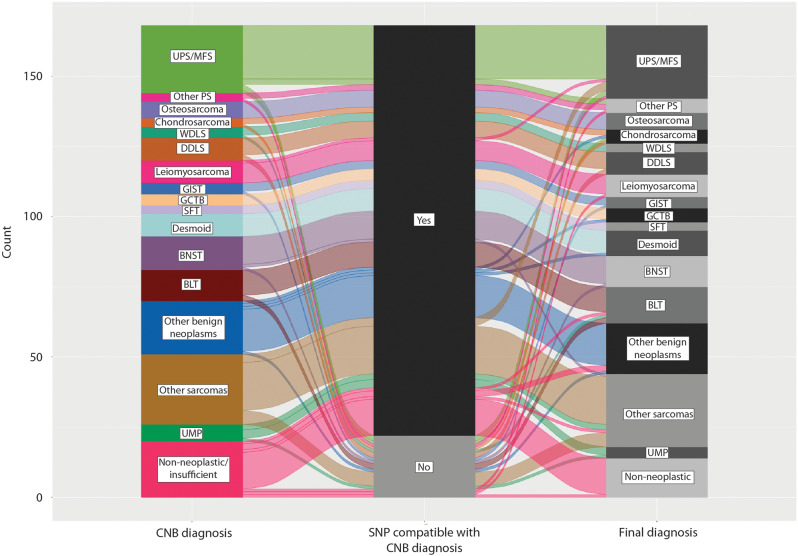
Alluvial plot with CNB diagnosis (left), compatibility of the genomic profile of CNB with the CNB diagnosis (middle), and final diagnosis (right). The height of columns and flows represent the number of cases. SNP = single nucleotide polymorphism array, UPS/MFS = undifferentiated pleomorphic sarcoma/ myxofibrosarcoma, PS = pleomorphic sarcoma, WDLS = well-differentiated liposarcoma, DDLS = dedifferentiated liposarcoma, GIST = gastrointestinal stromal tumor, GCTB = giant cell tumor of bone, SFT = solitary fibrous tumor, BNST = benign nerve sheath tumors, BLT = benign lipomatous tumors, UMP = neoplasms of unknown malignant potential.

SNP profiles that were compatible with the corresponding CNB diagnoses were found in 146/168 (87%) cases: 54 were diagnosed as benign, 71 as malignant, 5 as neoplasms of UMP, and 16 as nonneoplastic/insufficient material. CNAs were found in 86 of them, and 60 had normal SNP array profiles. The data are summarized in Fig. 1.

#### Copy number profiles not compatible with the CNB diagnoses

SNP profiles that were not compatible with the corresponding CNB diagnoses were found in 22/168 (13%) cases: 14 were diagnosed as malignant, four as benign, three as nonneoplastic/insufficient material, and one as neoplasm of UMP. Of the 14 malignant CNBs with discrepant genetic results, 10 had normal CNA profiles; five of these had the expected CNAs in the surgical biopsy, whereas the remaining three cases that underwent surgery remained normal; the final diagnoses in these three cases were liposarcoma, NOS (Case 49), chondrosarcoma (Case 67), and MPNST in the background of a neurofibroma (Case 159). Of the discrepant cases with normal SNP array profiles that did not undergo surgery, there were two diagnosed as spindle cell lipoma.

Five malignant lesions had CNAs that did not fit with the morphologically suggested subtype: two dedifferentiated liposarcomas had copy number profiles suggestive of ALT/WDLS (see below), one LGFMS had copy number changes not previously reported in this tumor (and the surgical biopsy was negative for *FUS*-rearrangement at FISH), a well-differentiated liposarcoma had a deletion in chromosome 1 as the sole change (the final diagnosis was changed to lipoma), and the sample diagnosed as malignant, but with necrosis/insufficient material (Case 165), had complex changes suggestive of UPS/myxofibrosarcoma; the final diagnosis in Case 165 was pleomorphic sarcoma. For a summary, see Fig. 1.

#### Copy number profiles indicative of specific diagnoses

Some copy number profiles are more or less strongly associated with particular tumor entities; they were here defined as “indicative of” a certain diagnosis (Supplementary Table 1). Below follows a short summary of the aberrant SNP array profiles in tumor types or groups of lesions that were represented at least three times in our series.

##### Myxofibrosarcoma/UPS

On the basis of a non-diploid stem line and the presence of >50 structural rearrangements affecting more than half of the chromosomes, the SNP array profile was interpreted as suggestive of MFS/UPS in 23 cases. The final diagnoses were MFS or UPS in 19 of these cases, pleomorphic liposarcoma in two, and leiomyosarcoma and pleomorphic sarcoma NOS in one case each.

##### Liposarcoma

The copy number profile of six cases was considered indicative of DDLS on the basis of amplicons in chromosome arm 12q (always including *MDM2* and *CDK4*) in combination with genome-wide copy number changes and/or amplification of *JUN* in 1p. The final diagnosis was DDLS in all six cases. In five cases, the finding of similar 12q amplicons, but without concomitant *JUN* amplification or genome-wide CN changes, was interpreted to suggest ALT or WDLS, depending on the location of the tumor. The final diagnoses in these five cases were WDLS/ALT in three and DDLS in two.

##### Osteosarcoma

The finding of extremely complex SNP array profiles (aneuploidy, >30 structural rearrangements) in five bone lesions was interpreted to be indicative of high-grade osteosarcoma (OS), which was also the final diagnosis in all these cases. In one CNB from a tumor with unspecified relation to the bone, the finding of characteristic amplicons in 12q was reported as suggestive of either ALT or parosteal OS, the latter being the final diagnosis.

##### Leiomyosarcoma

The SNP array profiles were interpreted to be indicative of conventional LMS or inflammatory LMS (ILMS) in three and two cases, respectively. All profiles suggestive of conventional LMS were complex with structural CN changes affecting more than half of the chromosomes; in addition, two of them had amplification of the *MYOCD* gene and one was a suspected metastasis in a patient with a known primary LMS. The final diagnosis was LMS in all three cases. Two cases had genomic profiles strongly suggestive of ILMS, i.e., haploidization with retained heterozygosity for chromosomes 5 and 22; the final diagnosis was ILMS in both cases.

##### GIST

Three intraabdominal tumors showed various combinations of deletions of 1p and loss of 14q and/or 22q material, strongly suggesting GIST, which was the final diagnosis in all three cases.

##### Chondrosarcoma

SNP array profiles showing aneuploidy and mostly numerical changes in three bone lesions from patients aged 25–43 years were interpreted to be suggestive of chondrosarcoma, which was the final diagnosis in two of them while a differential diagnosis between chondrosarcoma or osteosarcoma remained in one case.

#### Copy number profiles indicative of broad disease categories

Some genomic profiles were regarded as not being specific for distinct tumor types, but still suggestive of groups of neoplasms: high-grade sarcoma NOS, sarcoma NOS, and neoplasia NOS.

In ten cases the SNP array profile was reported to be suggestive of high-grade sarcoma, NOS. The decision to suggest high-grade sarcoma NOS, rather than MFS/UPS, was based on assumed near-diploidy, structural copy number changes affecting less than half of the chromosomes, and/or presence of amplicons not previously reported in MFS/UPS. The final diagnoses were MFS/UPS in two cases, LMS or spindle cell sarcoma in two each, and mesenchymal chondrosarcoma, pleomorphic liposarcoma, and myxoid sarcoma NOS in one each. The copy number profiles in four cases had <20 structural copy number changes affecting 3–9 chromosomes and were suggested to represent sarcoma NOS. The final diagnoses were UPS, superficial angiomyxoma, and two neoplasms of UMP (one each of atypical spindle cell and myxoid neoplasm). Nine cases had aberrant copy number profiles with 1–4 CNAs in what was interpreted as a near-diploid context. None of the CNAs had any known association with any particular tumor entity, but the presence of a clonal CNA strongly suggested that the lesion was neoplastic rather than reactive. The final diagnoses were giant cell tumor of bone in three cases, desmoid fibromatosis and LGFMS in two each, and low-grade spindle cell sarcoma and lipoma in one case each.

### Jaccard similarity index and representativity of copy number profiles in CNB material

**Fig. 2:**
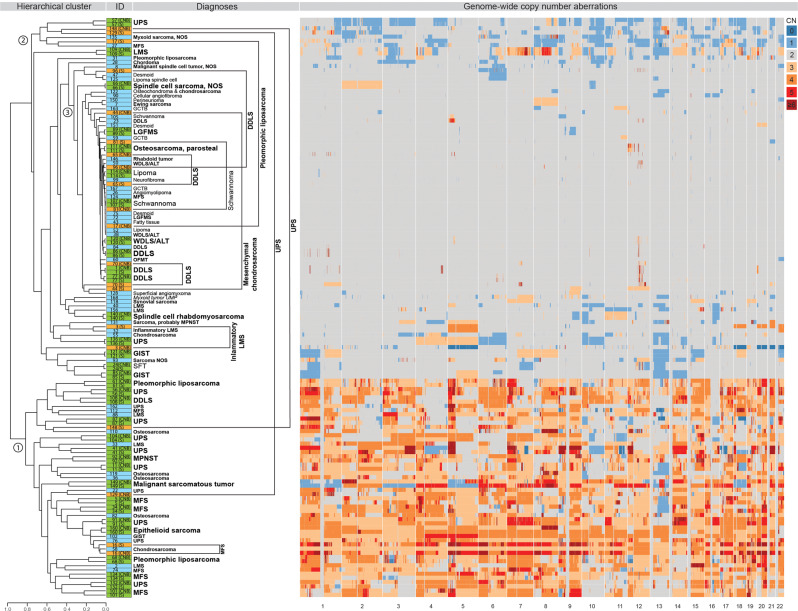
Hierarchical cluster based on Jaccard similarity index. Each sample is indicated by a colored box with case ID and associated diagnosis. Bold letters indicate malignant diagnoses, regular letters indicate benign diagnoses and letters in italics indicate neoplasms of unknown malignant potential (UMP). Blue = single sample, green = matched samples clustering next to each other, orange = matched samples, spread in hierarchical cluster. Matched samples not clustered next to each other are connected by an arc. Associated whole-genome heatmap of absolute copy numbers represented by different color shades from blue (loss) to red (gain). ID = Case ID, CN = absolute copy number, UPS = undifferentiated pleomorphic sarcoma, NOS = not otherwise specified, MFS = myxofibrosarcoma, LMS = leiomyosarcoma, GCTB = giant cell tumor of bone, DDLS = dedifferentiated liposarcoma, LGFMS = low-grade fibromyxoid sarcoma, WDLS/ALT = well-differentiated liposarcoma/atypical lipomatous tumor, MPNST = malignant peripheral nerve sheath tumor, OFMT = ossifying fibromyxoid tumor, GIST = gastrointestinal stromal tumor, SFT = solitary fibrous tumor.

A total of 138 samples from 95 cases (43 cases had data on both CNB and surgical specimen) had SNP profiles of sufficient quality for segmentation; in the segmentation analysis, the final diagnosis was used. The segment files were clustered using the Jaccard similarity index (Fig. 2). Two clusters were largely composed of high-grade sarcomas with complex, genome-wide CNAs; one cluster was dominated by gains and one by losses (Clusters 1 and 2). Tumor types within these clusters included UPS, MFS, osteosarcoma, LMS, and pleomorphic liposarcoma. The third, and largest, cluster encompassed tumors with fewer CNAs. All benign diagnoses, except one SFT (Case 24), were included here. The remaining samples formed sub-clusters of varying size, and were composed of various malignant or UMP tumors that displayed intermediate levels of complexity.

For 43 of the 96 CNB cases—including 4 benign and 39 malignant lesions—corresponding segmentation files could be made for the SNP array data on the surgical biopsy. The median Jaccard score for matching samples was 0.90 (range 0.12–0.99); in 33 (77%) of them, the matching CNB and surgical samples clustered next to each other (Jaccard score between 0.61 and 0.99, median 0.96; Fig. 2). In general, tumor pairs with less complex SNP array profiles reached higher Jaccard scores; for example, most ALTs and DDLSs had Jaccard scores above 0.98, whereas tumors with more complex CNAs tended to have Jaccard scores below 0.9.

Ten sample pairs did not cluster with each other. In six of those (Cases 16, 44, 65, 70, 81, and 96), the Jaccard scores were nonetheless relatively high (0.83–0.97). The remaining four pairs of samples (Cases 3, 17, 129, 148), which had SNP profiles of varying complexity, had much lower Jaccard scores (0.12–0.57); in two of them this could be explained by strong contamination with normal cells or suboptimal technical quality in one of the samples (Cases 17 and 148). In one case (Case 3), that, visually, had identical SNP array profiles in the CNB and the surgical biopsy, the discrepancy was due to different ploidy level assumptions in the two samples. Distinct differences in the SNP array profile were found in only one case, a UPS with highly complex CNA profile (Case 129).

### Quantification of CNAs

**Fig. 3:**
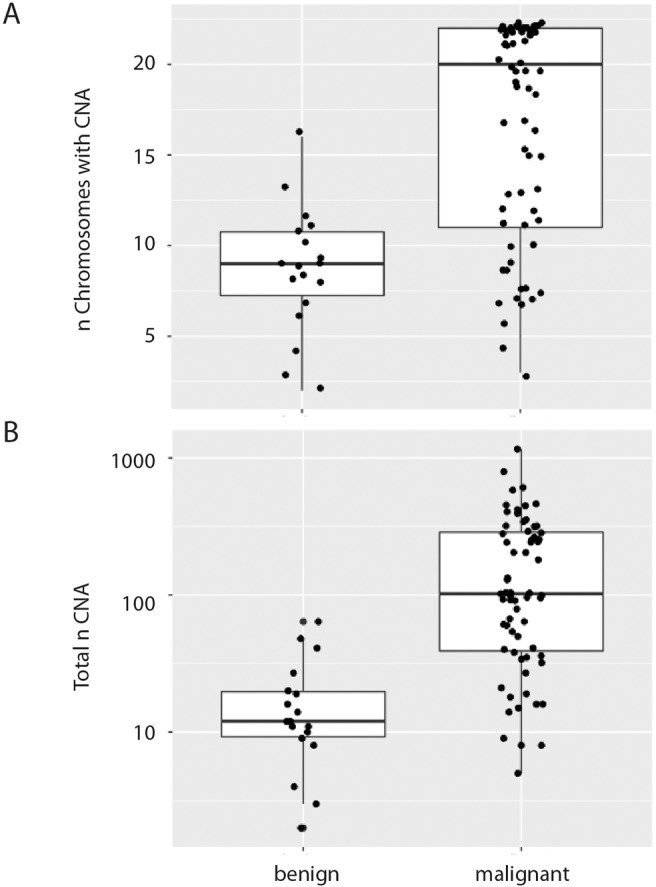
Number of copy number aberrations among benign and malignant neoplasms. Boxplots comparing benign and malignant neoplasms regarding (**A**) the number of chromosomes with copy number aberrations and (**B**) the total number of copy number aberrations. CNA copy number aberrations.

Filtered segmentation files retrieved from SNP array analyses on CNB material were used for CNA quantification (*n* = 87). The cases included 18 benign and 67 malignant neoplasms, one neoplasm of UMP and one nonneoplastic lesion. The distribution of the total number of copy number shifts per sample and the number of chromosomes displaying CNAs were significantly higher in malignant tumors than in benign lesions (Wilcoxon test, *p* < 0.001; Fig. 3).

## Discussion

In the present study we have evaluated the technical feasibility of obtaining global copy number information with SNP arrays in CNBs from STBT patients, and assessed the diagnostic information that can be derived from the copy number profiles. From a technical point of view, the results were excellent: SNP array analysis could be performed on 168/171 (98%) of the CNB samples, showing that the amount of DNA extracted from preoperative CNBs is sufficient for SNP array analysis. The failure rate (2%) was thus much lower than what has been reported for chromosome banding analysis (19%) on CNB samples^3^. Another important technical aspect concerns the representativity of the genomic profile in the CNB, i.e., to what extent the genetic features of the tumor can be captured through the analysis of only a small fraction of the tumor cells. As most malignancies display intratumoral heterogeneity with regard to CNAs, as well as to SNVs, indels, and structural variants^6,7^, it is unlikely that the analysis of a single biopsy can capture the full mutational spectrum. Nevertheless, in those 43 cases from which we had access to abnormal SNP array profiles from a CNB and its matching excision biopsy, the results were in most cases in good agreement with each other. In general, the results were highly similar, with a median Jaccard score of 0.90, and distinct differences that could not be explained by low tumor cell fraction or suboptimal technical quality in one of the two samples in a pair, were found in only one (2%) case. This tumor (Case 44) was a mesenchymal chondrosarcoma, which is gene fusion-driven. Hence, all the CNAs observed are presumably secondary events, with as yet unknown biological relevance. Similar, distinct CNA profiles have been observed before in multiple samples from fusion-driven sarcomas^8^. Thus, we conclude that the aberrant genomic profile seen in CNBs in the vast majority of cases provides an accurate view of the tumor stem line.

The role of global CNA information in STBT diagnostics is, for several reasons, more difficult to evaluate. First, STBT diagnostics relies on different sets of information, where the final diagnosis depends on findings supporting the diagnosis, as well as on findings arguing against potential differential diagnostic entities. Consequently, the analysis of samples from suspected malignant STBTs today typically includes a variety of IHC stains to aid the morphologic analysis. Although some genetic features should have a very strong impact on the final diagnosis, such as the finding of an *SS18*::*SSX1* fusion in a suspected synovial sarcoma, they are never sufficient. Indeed, the latest WHO classification of STBTs introduced two levels (“essential” and “desirable”) of diagnostic criteria for STBTs. The vast majority of entities do not have a genetic feature listed as “essential”; curiously enough, this includes tumor types named after the underlying driver mutation (e.g., *CIC*-rearranged sarcoma). From this standpoint, the genetic data could thus at best be used to support or argue against a diagnosis, and to what extent that information affects the final diagnoses will depend on the strength of all data combined. Second, a substantial fraction – higher for CNBs than for surgical biopsies^9^ – of STBTs cannot with certainty be classified as a recognized tumor entity. This was illustrated in the present study by the cases that were diagnosed as, e.g., “atypical spindle cell neoplasm” or “myxoid tumor with unknown malignant potential”. Third, there is a substantial inter-observer variation in the diagnostics of STBTs, making it likely that some tumors in the present series would have obtained alternate diagnoses at other sarcoma centers^10–12^. Finally, the genetics of many STBTs remain poorly explored, not least when it comes to the spectrum of CNAs.

Taking all these considerations into account, we decided to designate the copy number profiles as “compatible with” and, in cases with CNAs, the stronger “indicative of” certain diagnoses or broad diagnostic groups. As for the general diagnostic issues mentioned above, the interpretation of the copy number profile of an STBT is far from straightforward. For instance, a disadvantage of using CNAs for clinical purposes, compared to the use of SNVs, gene fusions, and karyotypes, is that databases on CNAs, in which one could search for combinations of CNAs to obtain diagnostic suggestions, are currently lacking. Hence, the interpretation of the results relies on data that can be found in publications. Furthermore, the significance of the profile is context-dependent; the finding of multiple amplicons in chromosome arm 12q as the sole change is indicative of a parosteal osteosarcoma when found in a tumor arising from the surface of a bone, but of an ALT when arising in soft tissue. Finally, the terms used here (“compatible with”, “indicative of”) are vague. For instance, not only reactive lesions but also many benign STBTs, and some gene fusion-associated sarcomas, do not have any CNAs, let alone any highly recurrent ones; hence, the lack of CNAs was here considered compatible with benign and nonneoplastic lesions alike, as well as with some sarcomas.

With these caveats in mind, we considered the SNP array results on CNB material to be compatible with the corresponding CNB diagnoses in 146/168 (87%) of the cases, showing that, in the great majority of the cases, the presence or absence of CNAs could provide support for the diagnosis. Of the 22 (13%) cases where the CNA data were interpreted not to be compatible with the CNB diagnoses, false-negative SNP array results predominated. The most common type of false-negativity was complete lack of CNAs in lesions that ought to have imbalances, e.g., spindle cell lipoma and various malignancies; such lack of CNAs was found in 13 of the 22 cases with discrepant results, presumably due to contamination with normal cells or biopsy material that was not representative for the sampled lesion. Arguably, though, the final diagnoses in two of the lesions without CNAs (neurofibroma and liposarcoma NOS, respectively) could have been compatible with a normal SNP array profile. Furthermore, the possibility that a conventional lipoma, a tumor type that usually does not display CNAs, was misdiagnosed as a spindle cell lipoma cannot be excluded. Another type of false-negative result was represented by two cases with CNA profiles compatible with ALT/WDLS, rather than with the final diagnosis dedifferentiated liposarcoma (Cases 77 and 96). Dedifferentiated cells were scarce in these CNB samples, suggesting that SNP array profiles represented the more predominant well-differentiated fraction. In summary, the results indicate that ~10–15% of the SNP array analyses yielded false-negative results. This illustrates a major drawback with SNP array analysis compared to whole-genome sequencing; with the latter approach, the absence/presence of SNVs and/or structural variants would have helped in deciding whether a sample was tumor-representative or not.

In contrast to the false-negative cases, some cases had aberrant SNP array profiles that were in disagreement with the CNB morphology, fitting better with the final diagnoses. Two such examples were the ILMS (Case 3) and undifferentiated pleomorphic sarcoma (Case 169) that had characteristic CNA profiles, but that were considered nonrepresentative at morphologic analysis. Another example (Case 114) included a lipoma that was diagnosed as ALT on CNB material, but that had an abnormal profile without the characteristic 12q amplicons, excluding an ALT diagnosis. Finally, a tumor that was diagnosed as LGFMS was negative for *FUS*-rearrangement at FISH and had CNAs not previously described in that tumor, strongly suggesting a misdiagnosis (Case 72).

Aberrant CNA profiles in CNBs (*n* = 95) were considered “indicative of” a certain diagnosis or diagnostic group, but the degree of association with certain diagnoses varied among these profiles. One end of this spectrum included profiles that so far have only been described in one tumor type (ILMS), whereas the other end (neoplasia NOS) contained a variety of CNAs of unknown biological and clinical significance. There was agreement between the specific diagnostic suggestion based on the SNP array profiles and corresponding specific morphologic CNB diagnoses in 83% and the final diagnoses in 86%. One example of such discordance is Case 105, diagnosed as schwannoma but showing a SNP array profile that was indicative of a neurofibroma (duplication in 5q, a homozygous deletion of *CDKN2A*, and LOH at *NF1*). The tumor was not resected but the possibility of a misdiagnosis (without clinical consequences) has to be considered. Another recurrent misclassification concerned MFS/UPS. The criteria we used to classify the copy number profile as indicative of MFS/UPS obviously missed a number of cases with slightly less complex genomes. This illustrates that many more cases need to be analyzed before we have a complete picture of the spectrum of genomic changes in these tumors.

CNAs as well as the number of chromosomes with CNAs were distributed significantly higher in malignant compared to benign neoplasms. However, the CNA numbers among both benign and malignant tumors were highly variable. Three benign tumors had CNA numbers above the lower quartile of CNA numbers in malignant tumors (CNA > 39). On the other hand, there were three malignant tumors with CNA counts around and below the 25th quartile of benign tumors (9 CNAs). The latter included two sarcomas (a MFS, Case 124, 8 CNAs and a pleomorphic liposarcoma, Case 17, 9 CNAs), that usually display highly complex genetic profiles. However, the results in both cases were corrupted by either a high amount of normal cells (Case 124) or a suboptimal technical quality of the analysis (Case 17), resulting in a low number of segmented CNAs.

In summary, the results show that it is feasible to obtain genome-wide copy number profiles from CNBs from STBTs. Furthermore, the copy number profiles in the CNBs are in most cases representative of the tumor stem line. From a clinical, diagnostic point of view, the copy number profiles were in most cases supportive of the morphological diagnosis, and in a few cases, they were instrumental in changing the diagnosis. A drawback with the current approach is the inability to tell whether a sample without CNAs is representative of the tumor cell population or not. In that respect, WGS, or even gene panels^13^, have important advantages. However, it should be emphasized that the genome-wide copy number information obtained through SNP arrays is far superior to what can be extracted even from large (>300) gene panels. Finally, it could be pointed out that although the clinical information on copy number changes in STBTs currently is limited to their diagnostic impact, it may well turn out that the copy number profiles—genome-wide as well as for individual genes—may have an impact on outcome^14^ and/or be suggestive of targeted therapies^15–20^. However, also other types of genetic aberration—such as gene fusions, single nucleotide variants, or methylation patterns—are likely to become more important in the management of sarcoma patients within the next few years, prompting a strategy for optimal handling of CNB material. In our study, the CNB material often could be used only for one type of analysis; ideally, the material should suffice for both RNA and DNA extraction and FISH analysis, but that would require more CNB material per patient than we obtained in the current study.

## Supplementary information

Supplementary Table 1

Supplementary Figure 1

## Data Availability

The datasets used and/or analyzed during the current study are available from the corresponding author on reasonable request.

## References

[1] Anderson WJ, Hornick JL (2019). Immunohistochemical correlates of recurrent genetic alterations in sarcomas. Genes Chromosomes Cancer.

[2] WHO Classification of Tumours. Soft Tissue and Bone Tumours, 5th edn. (IARC Press, 2020).

[3] Walther C, Domanski HA, Vult von Steyern F, Mandahl N, Mertens F (2011). Chromosome banding analysis of cells from fine-needle aspiration biopsy samples from soft tissue and bone tumors: is it clinically meaningful?. Cancer Genet.

[4] Rasmussen M (2011). Allele-specific copy number analysis of tumor samples with aneuploidy and tumor heterogeneity. Genome Biol.

[5] Mayrhofer M, Viklund B, Isaksson A (2016). Rawcopy: Improved copy number analysis with Affymetrix arrays. Sci. Rep.

[6] McGranahan N, Swanton C (2017). Clonal heterogeneity and tumor evolution: past, present, and the future. Cell.

[7] Dentro SC (2021). Characterizing genetic intra-tumor heterogeneity across 2,658 human cancer genomes. Cell..

[8] Hofvander J (2018). Different patterns of clonal evolution among different sarcoma subtypes followed for up to 25 years. Nat. Commun..

[9] Köster J, Ghanei I, Domanski HA (2021). Comparative cytological and histological assessment of 828 primary soft tissue and bone lesions, and proposal for a system for reporting soft tissue cytopathology. Cytopathology..

[10] Hasegawa T (2002). Validity and reproducibility of histologic diagnosis and grading for adult soft-tissue sarcomas. Hum. Pathol..

[11] Skeletal Lesions Interobserver Correlation among Expert Diagnosticians (SLICED) Study Group. (2007). Reliability of histopathologic and radiologic grading of cartilaginous neoplasms in long bones. J. Bone Joint Surg. Am..

[12] Eefting D (2009). Assessment of interobserver variability and histologic parameters to improve reliability in classification and grading of central cartilaginous tumors. Am. J. Surg. Pathol..

[13] Joseph NM, McGill KC, Horvai AE (2021). Genomic profiling of low-grade intramedullary cartilage tumors can distinguish enchondroma from chondrosarcoma. Am. J. Surg. Pathol.

[14] Lartigue L (2015). Genomic index predicts clinical outcome of intermediate-risk gastrointestinal stromal tumours, providing a new inclusion criterion for imatinib adjuvant therapy. Eur. J. Cancer.

[15] Arnaud-Coffin P (2020). Therapeutic relevance of molecular screening program in patients with metastatic sarcoma: analysis from the ProfiLER 01 trial. Transl Oncol.

[16] Chibon F (2010). Validated prediction of clinical outcome in sarcomas and multiple types of cancer on the basis of a gene expression signature related to genome complexity. Nat. Med..

[17] Cissé MY (2020). Targeting MDM2-dependent serine metabolism as a therapeutic strategy for liposarcoma. Sci. Transl. Med.

[18] Lucchesi C (2018). Targetable alterations in adult patients with soft-tissue sarcomas: insights for personalized therapy. JAMA Oncol..

[19] Que Y (2021). Frequent amplification of HDAC genes and efficacy of HDAC inhibitor chidamide and PD-1 blockade combination in soft tissue sarcoma. J. Immunother. Cancer.

[20] Suehara Y (2019). Clinical genomic sequencing of pediatric and adult osteosarcoma reveals distinct molecular subsets with potentially targetable alterations. Clin. Cancer Res.

