# Supplementary material for: The diagnostic utility of DNA copy number analysis of core needle biopsies from soft tissue and bone tumors
Source: Lab Invest. 2022 Mar 22;102(8):838–45. doi: 10.1038/s41374-022-00770-2 (PMC9309094; doi:10.1038/s41374-022-00770-2)

Supplementary Figure 1. Whole genome view of Single Nucleotide Polymorphism array log-ratio, allele frequency and allelic imbalance. Depicted are genomic profiles with available segmentation files.

CNB = core needle biopsies, NOS = not otherwise specified, UPS = undifferentiated pleomorphix sarcoma, GIST = gastrointestinal stromal tumor, MPNST = malignant peripheral nerve sheath tumor, UMP = unknown malignant potential

Case 1

Dedifferentiated liposarcoma

CNB

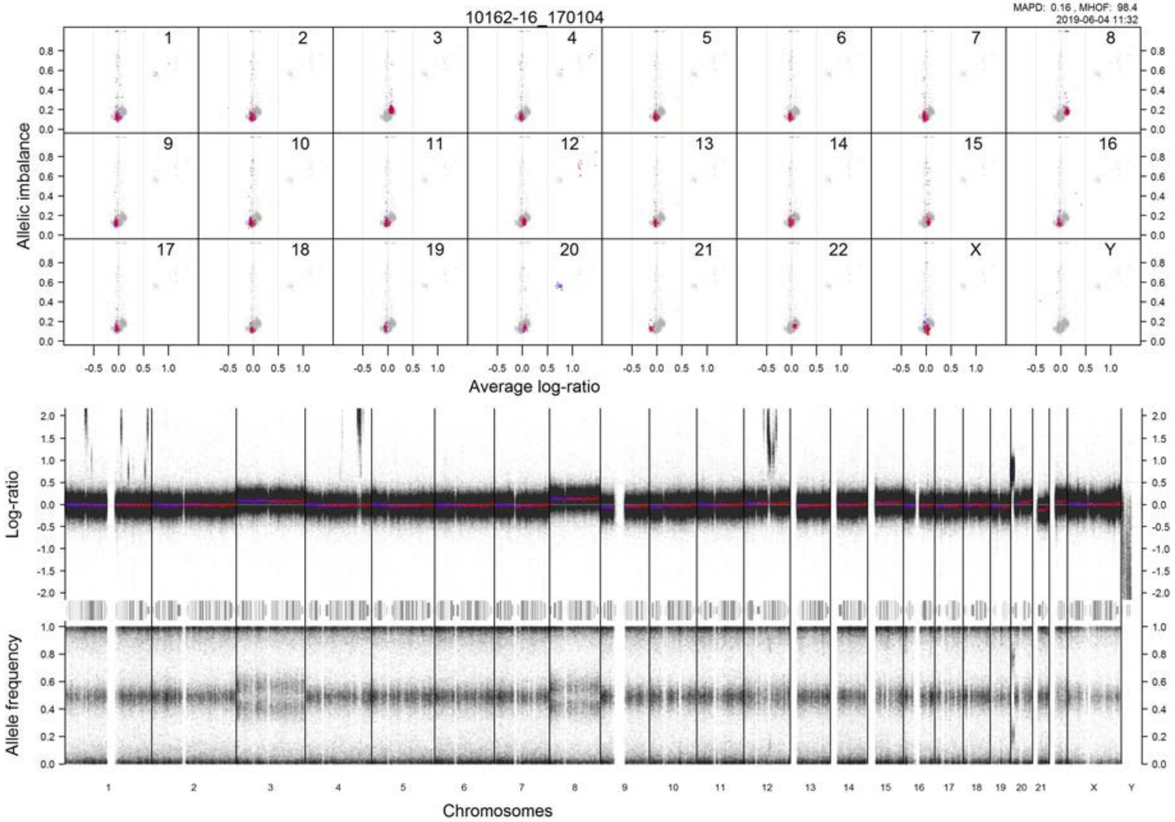

Surgical specimen

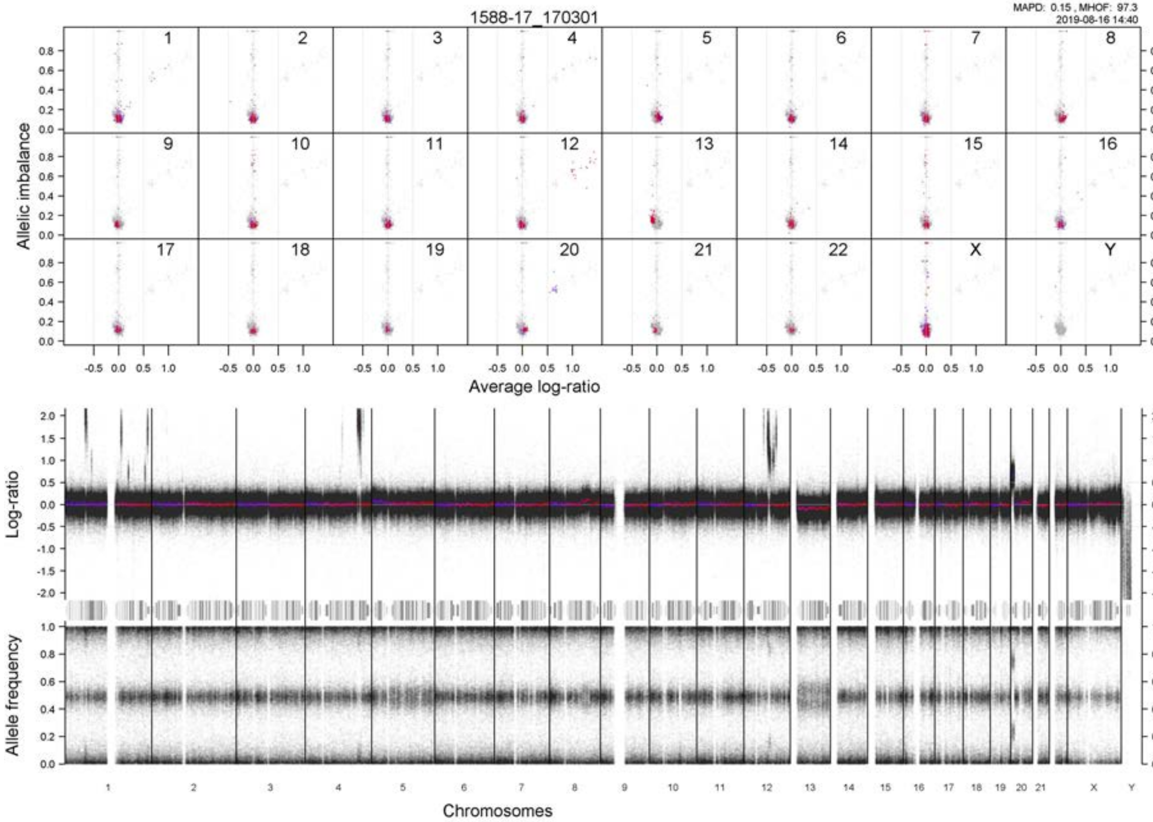

Case 2 (CNB)

Leiomyosarcoma

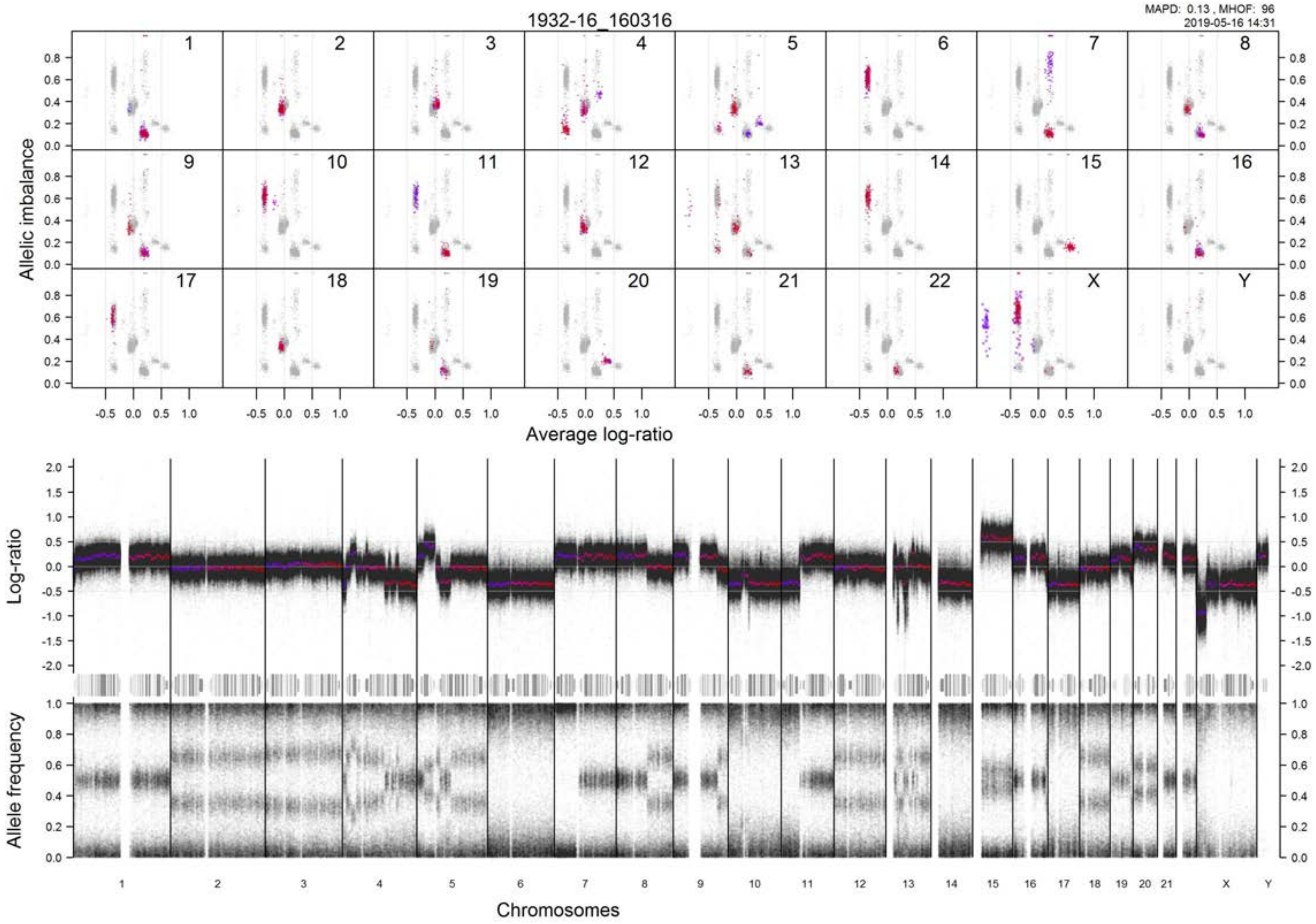

Case 3

Inflammatory leiomyosarcoma

CNB

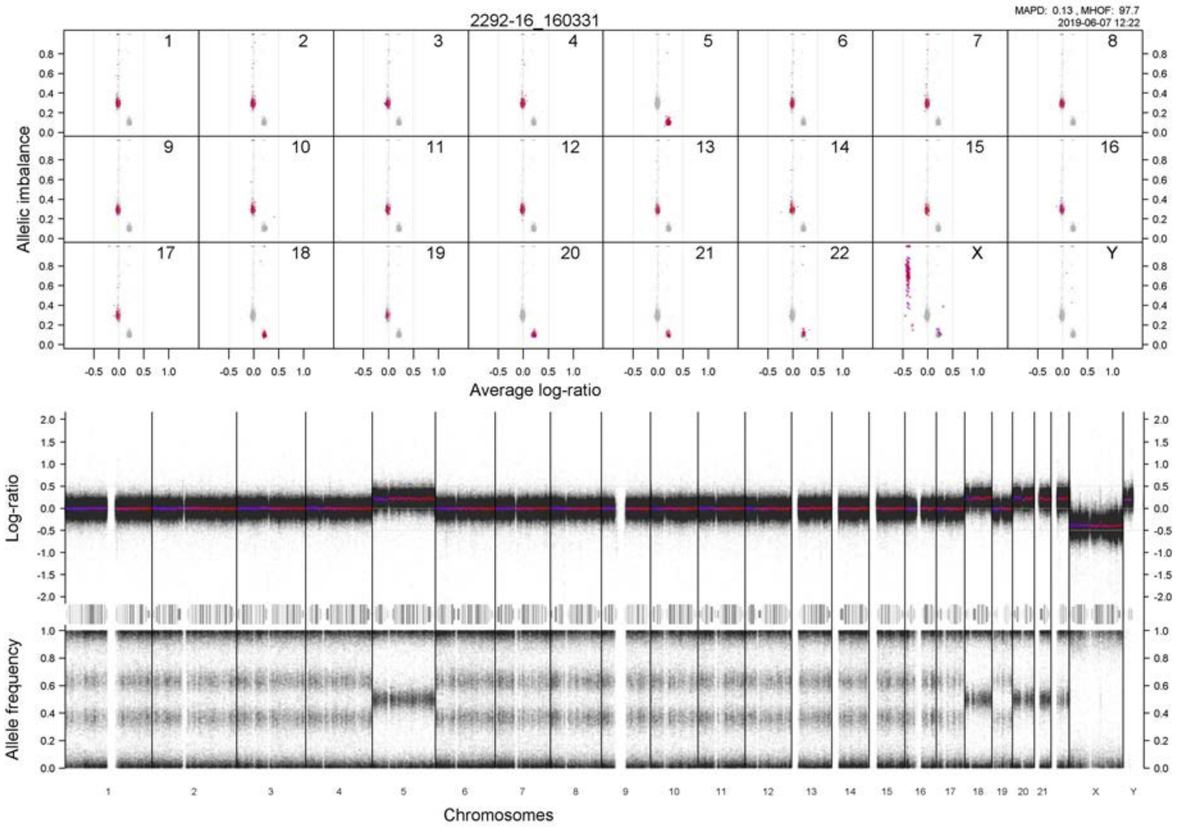

Surgical specimen

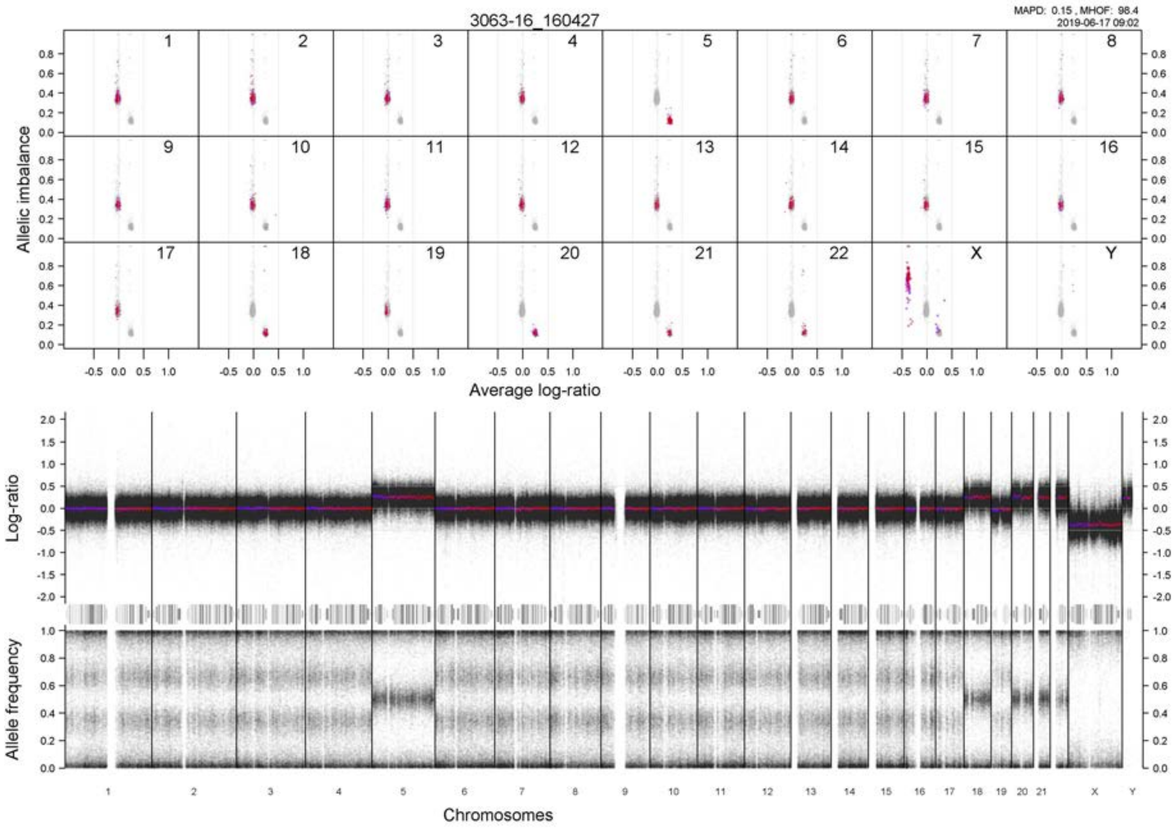

Case 5

Myxofibrosarcoma

CNB

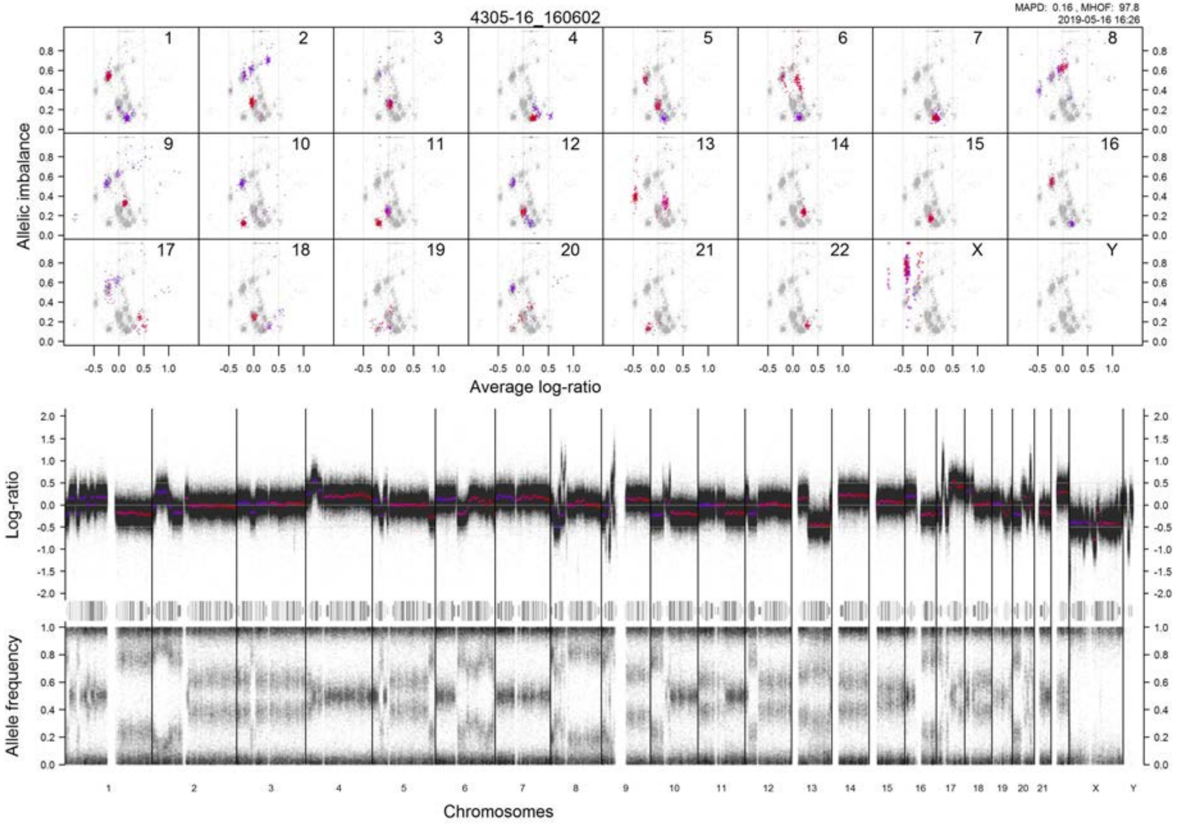

Surgical specimen

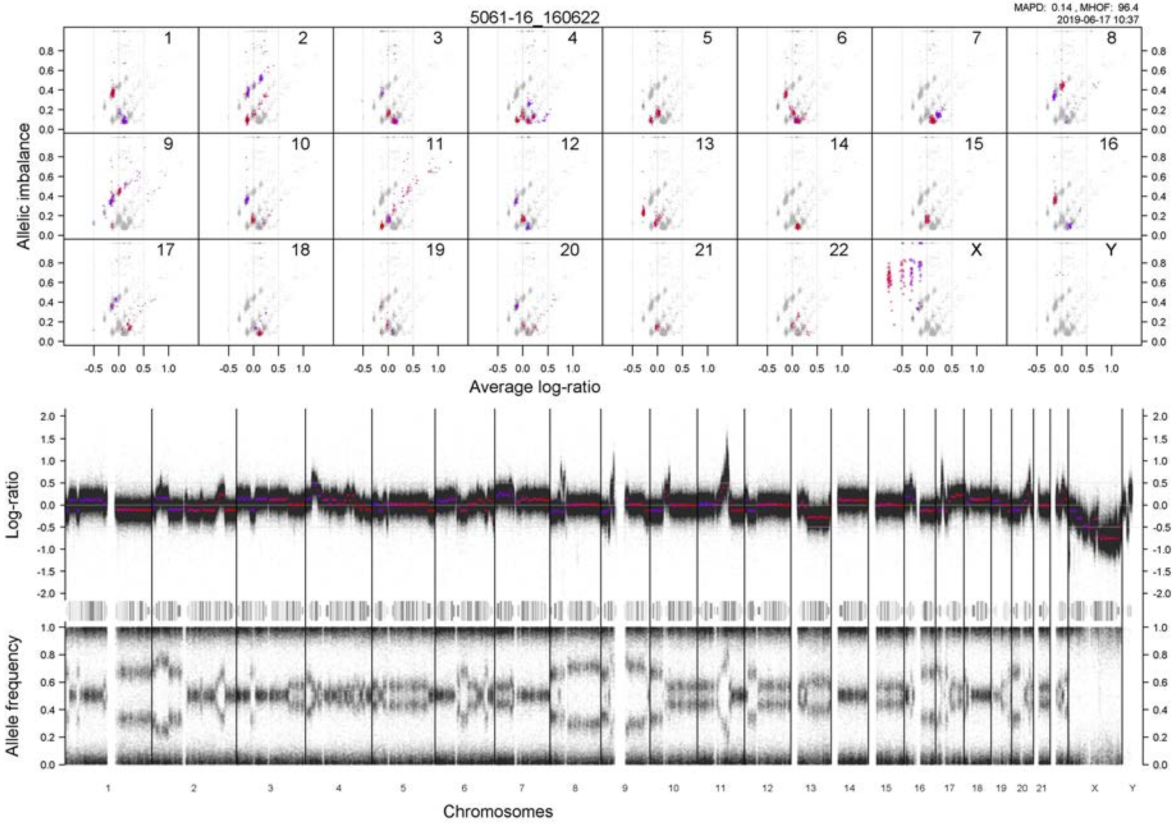

Case 7 (CNB)

Ewing sarcoma

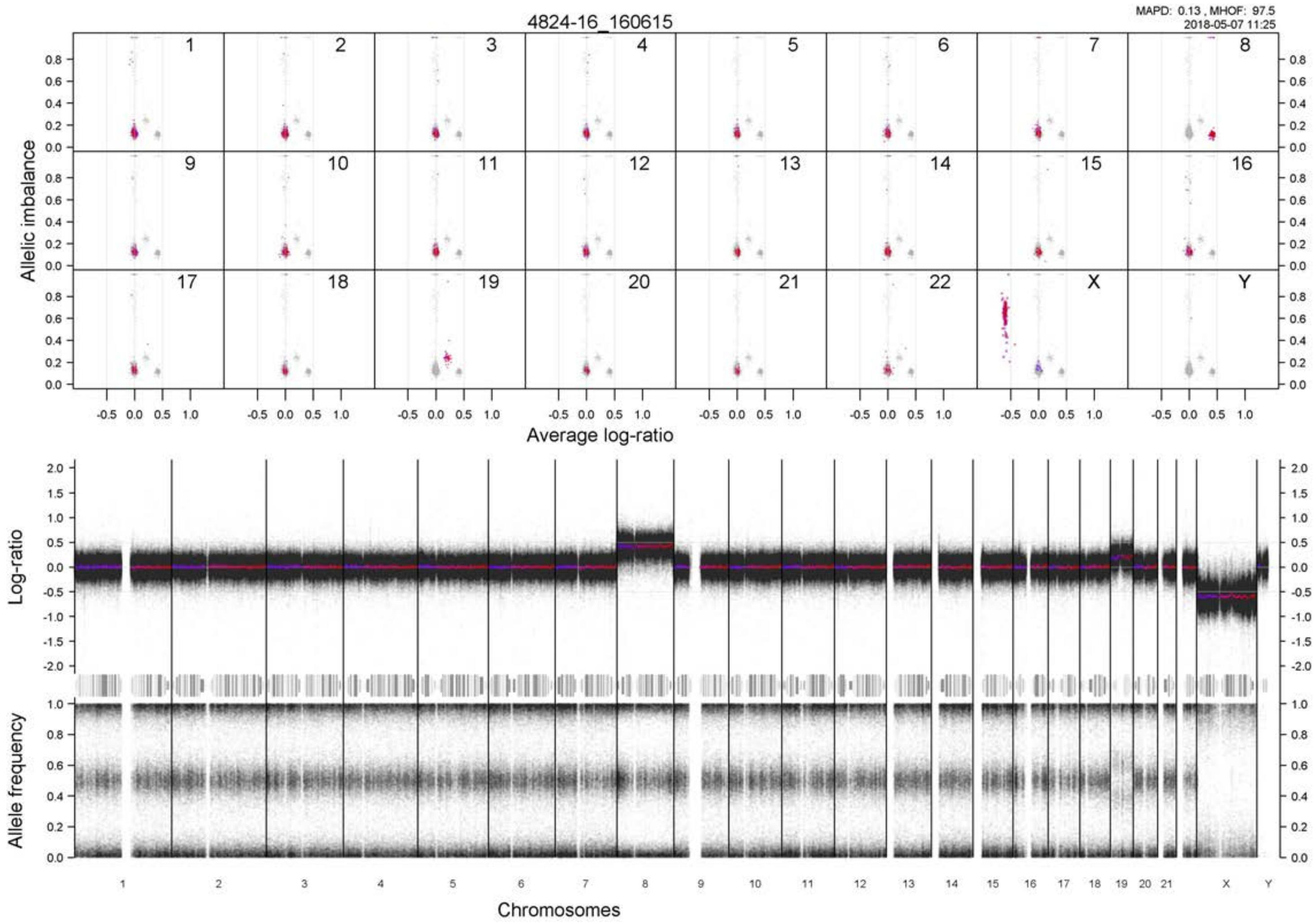

Case 8 (CNB)

Spindle cell sarcoma, NOS

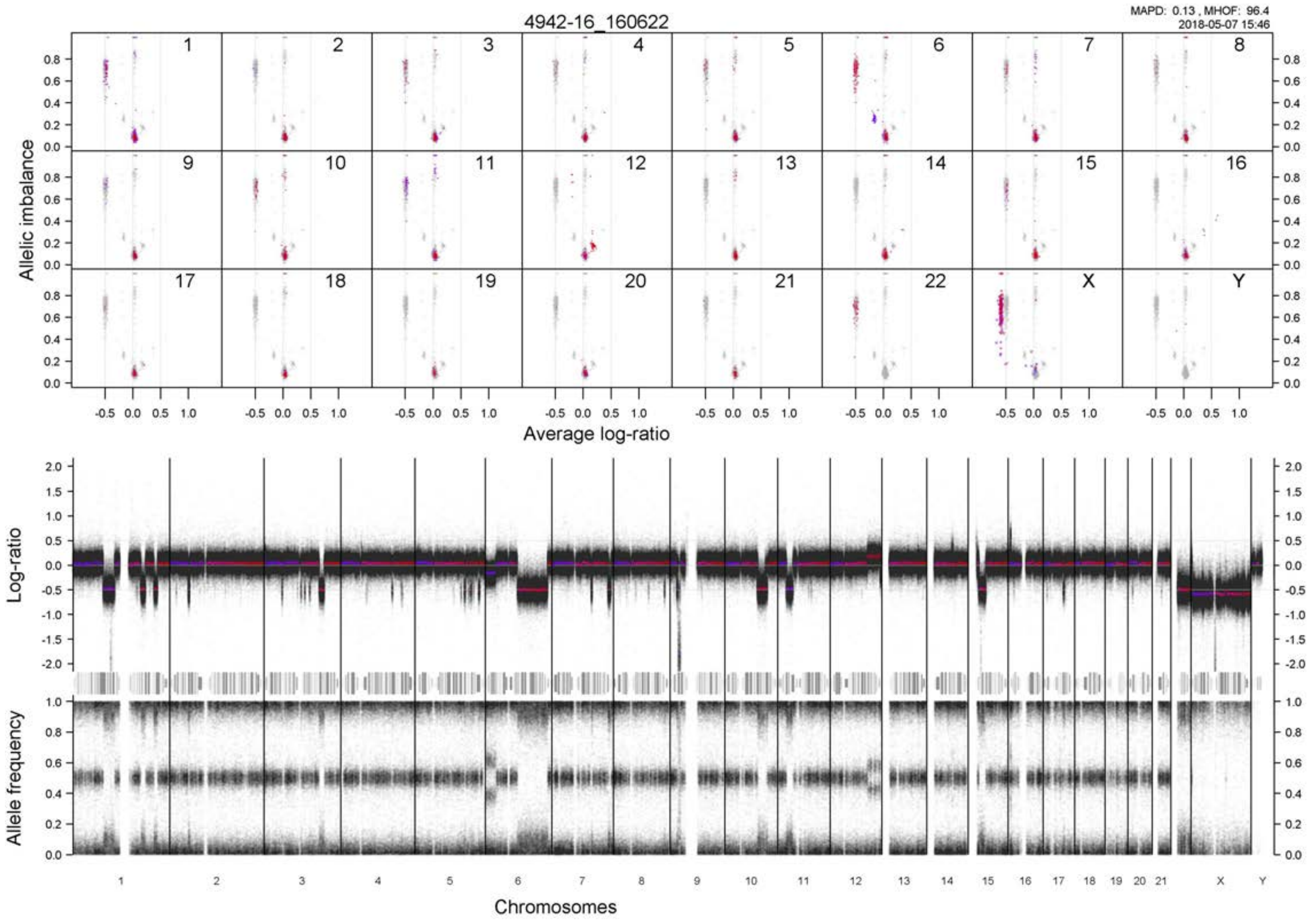

Case 11

UPS

CNB

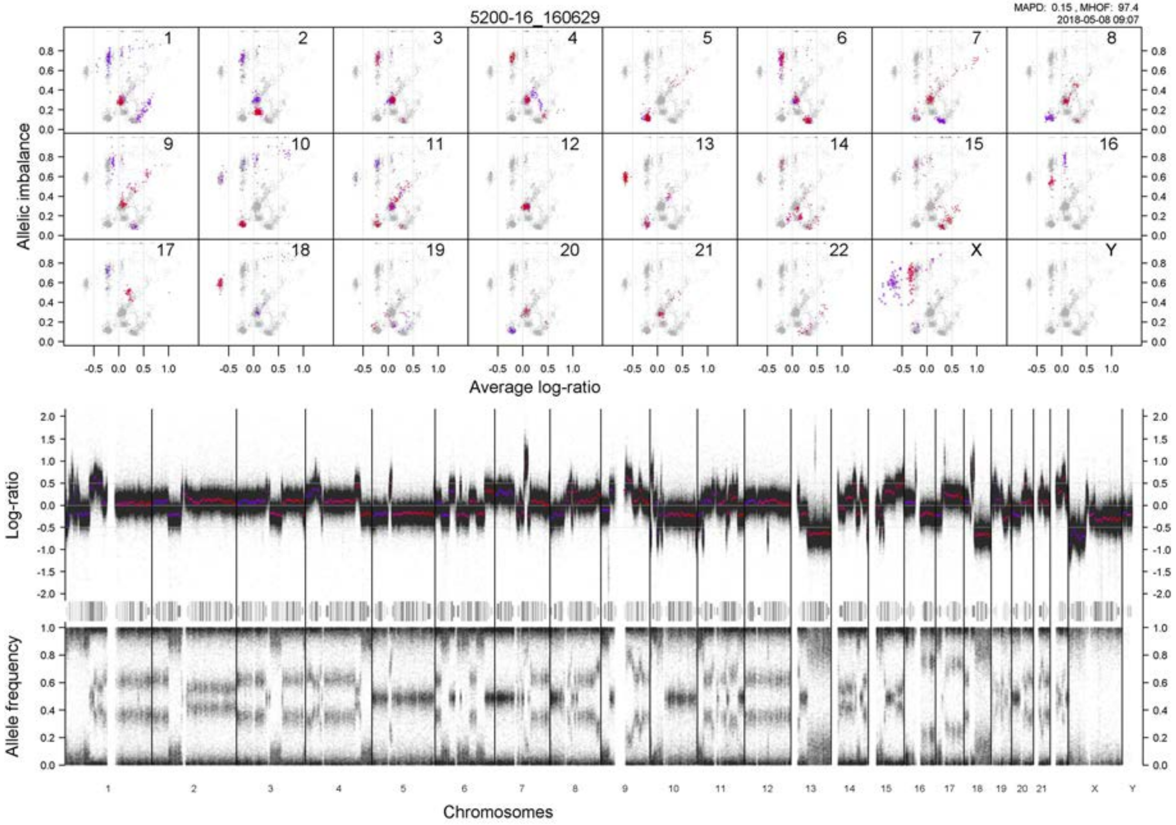

Surgical specimen

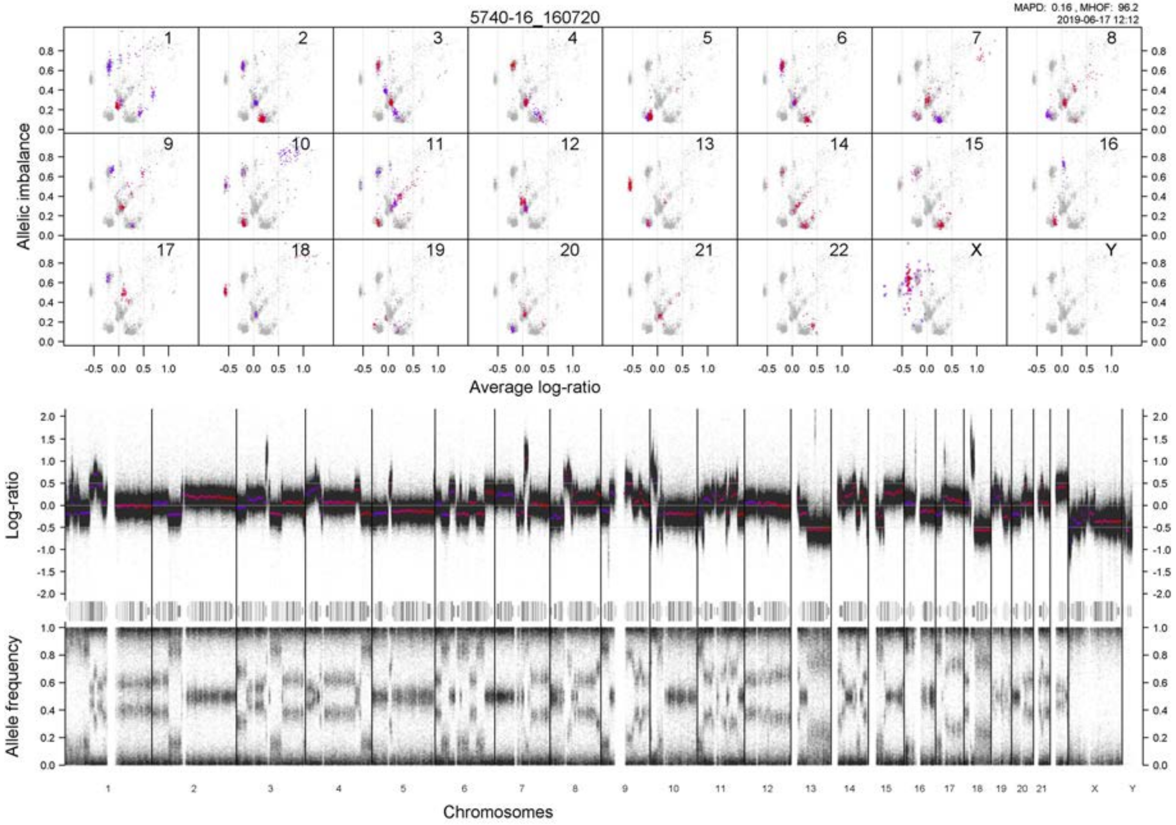

Case 16

Myxofibrosarcoma

CNB

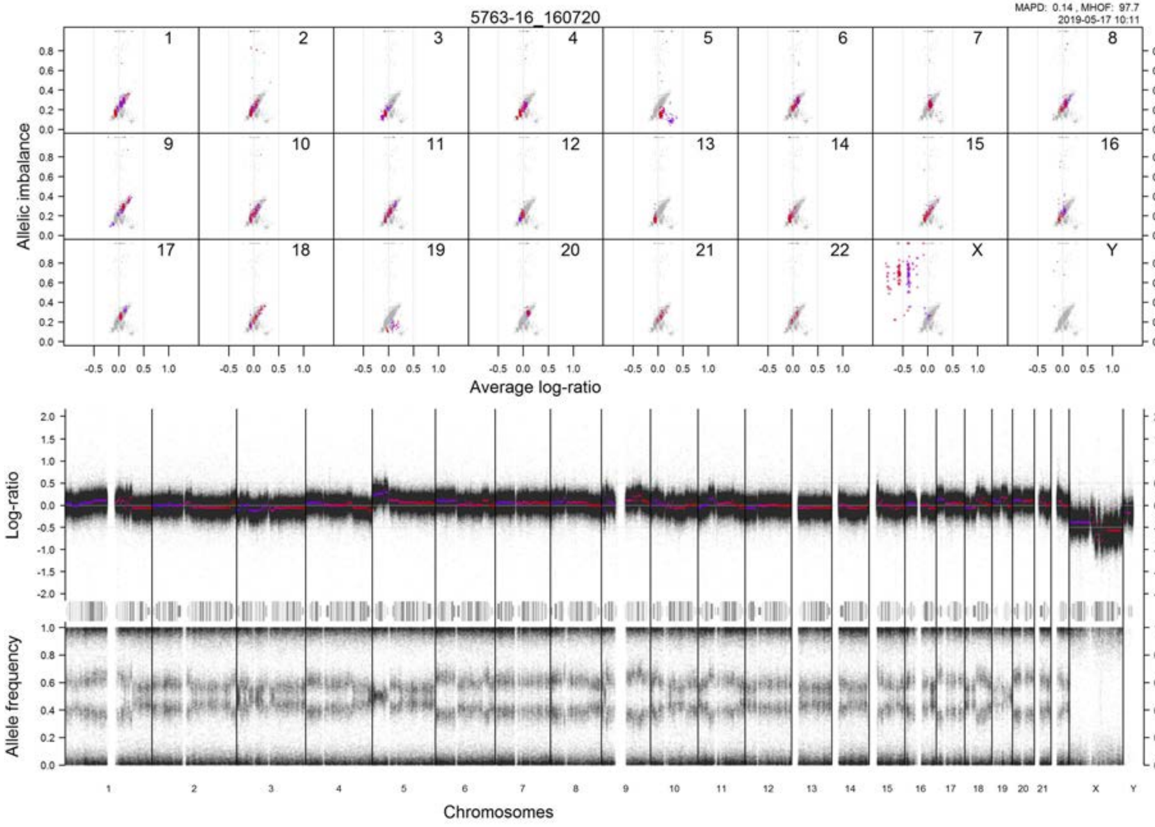

Surgical specimen

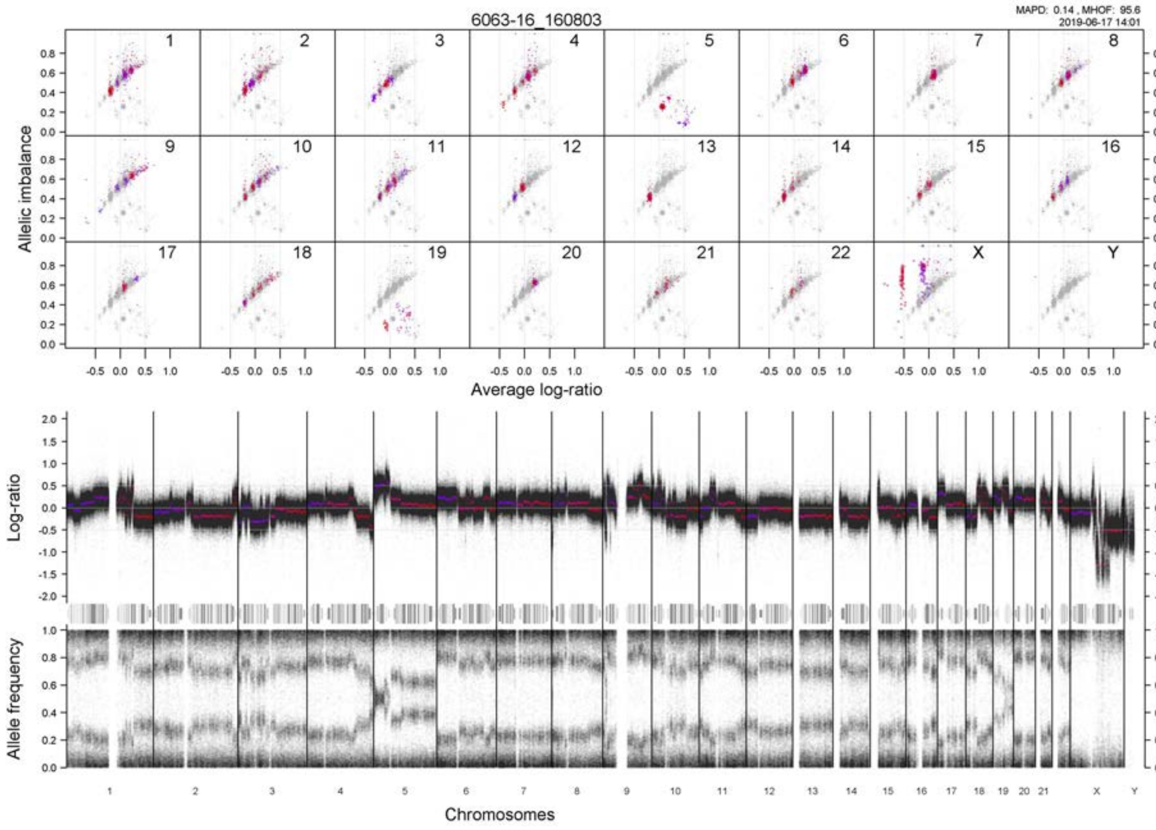

Case 17

Pleomorphic liposarcoma

CNB

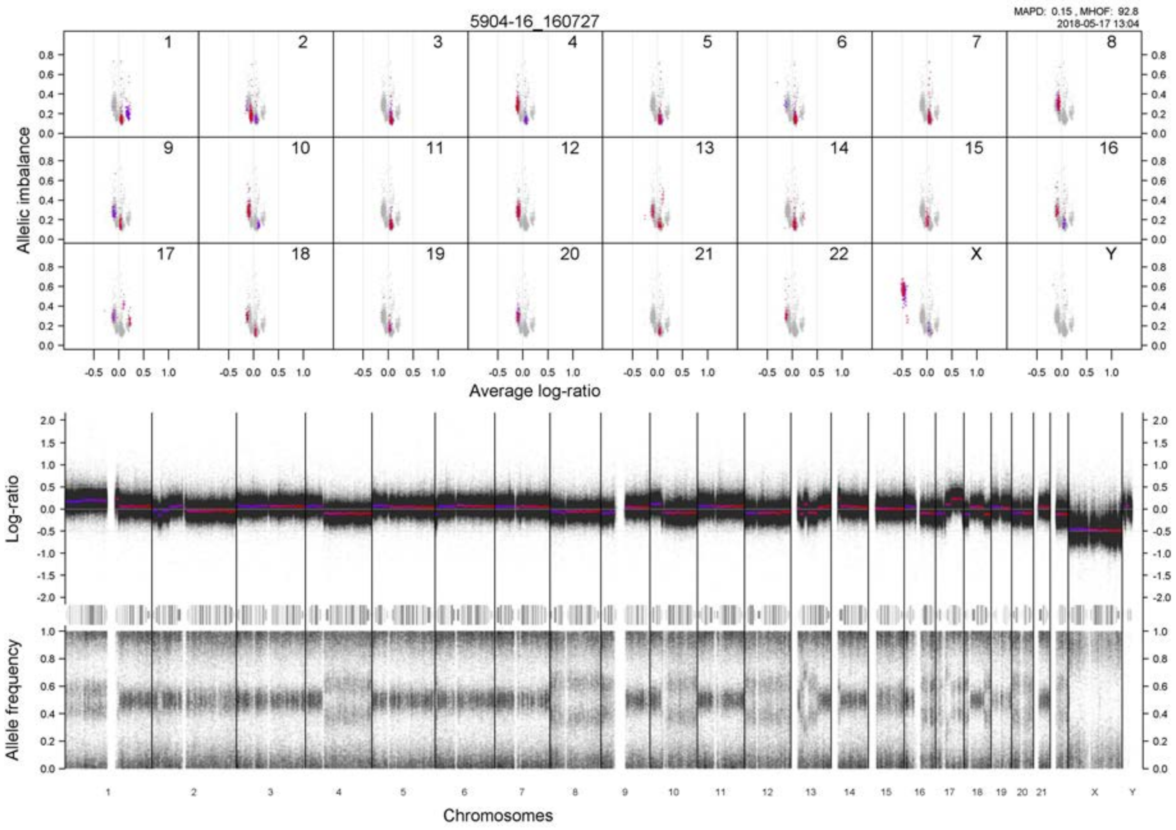

Surgical specimen

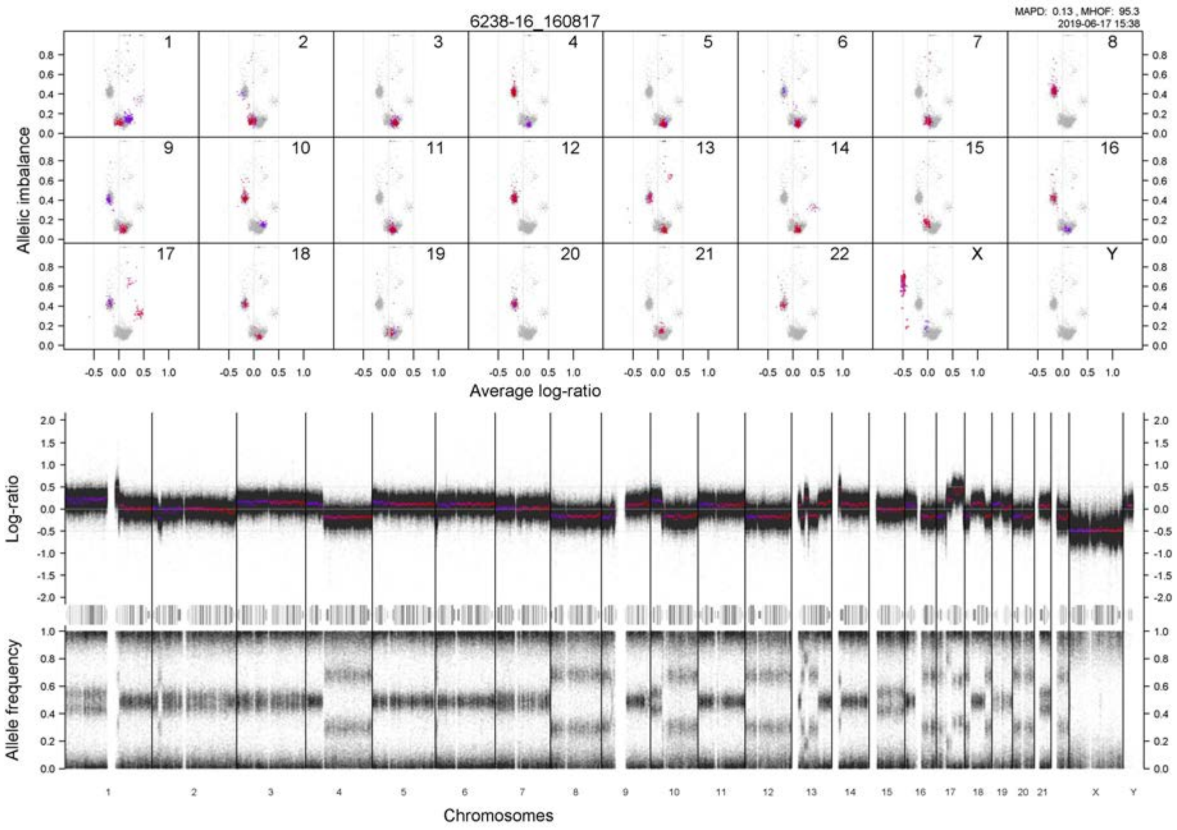

Case 22 (Surgical specimen)

UPS

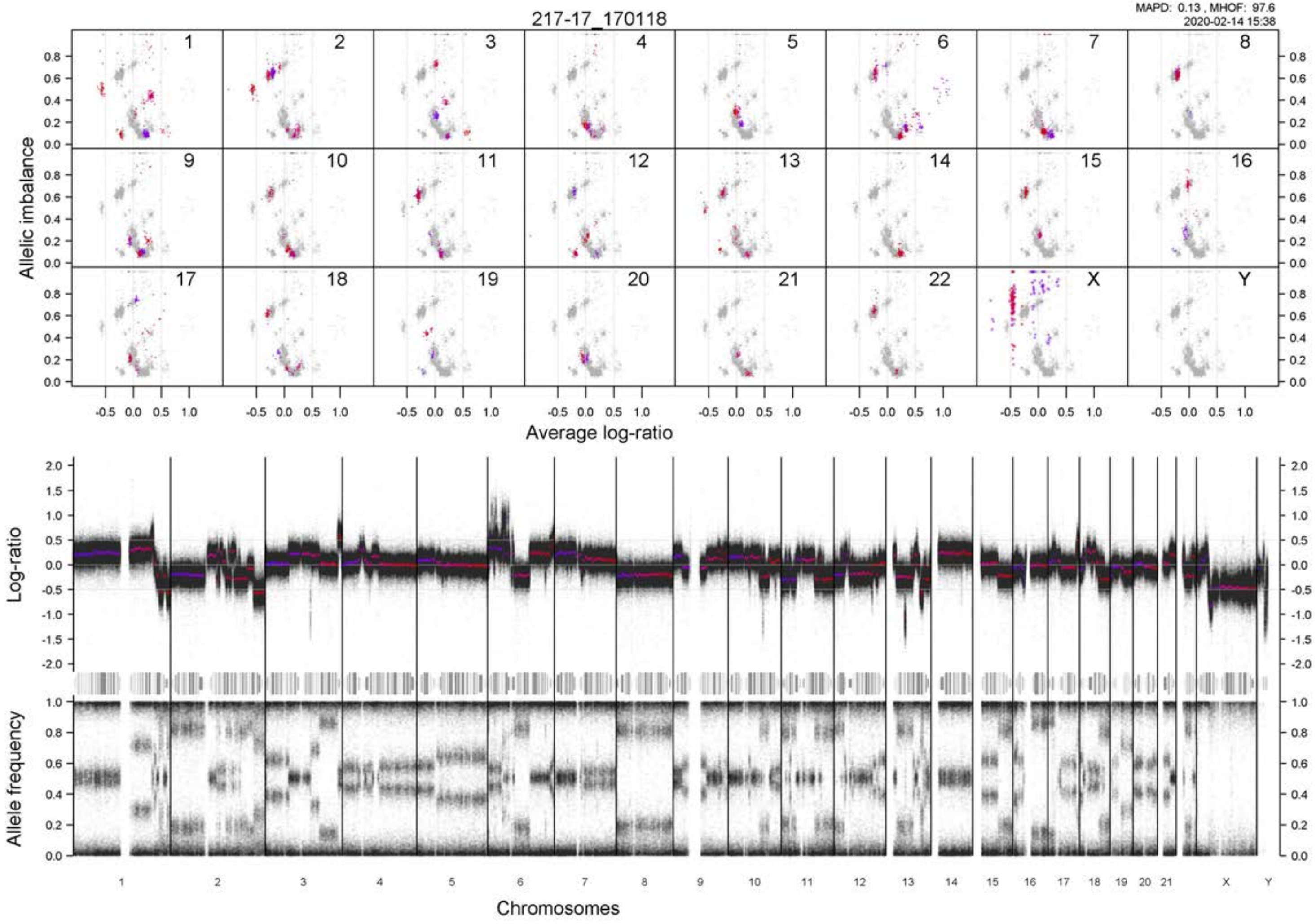

Case 23 (CNB) Dedifferentiated liposarcoma

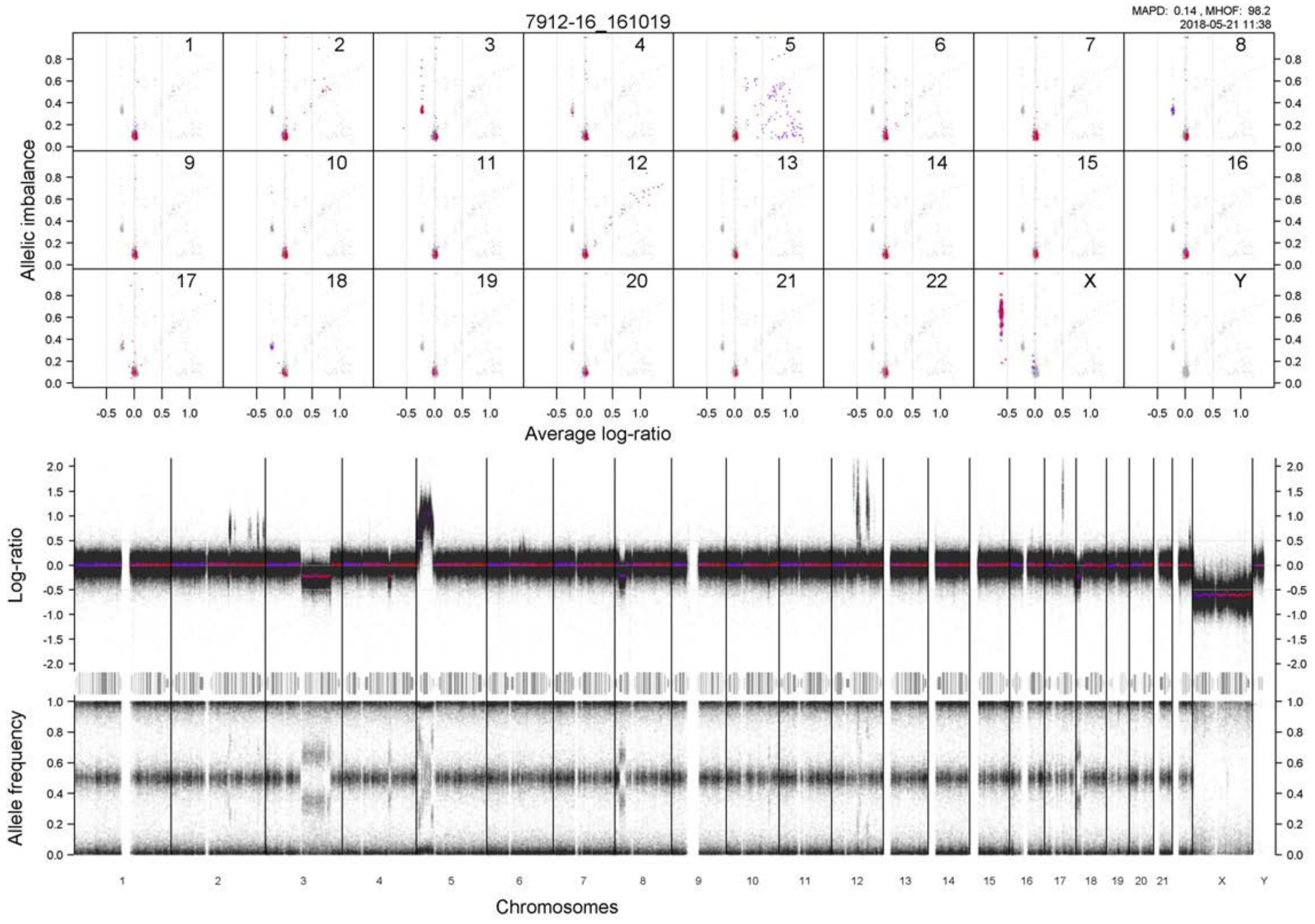

Case 24

Solitary fibrous tumor

CNB

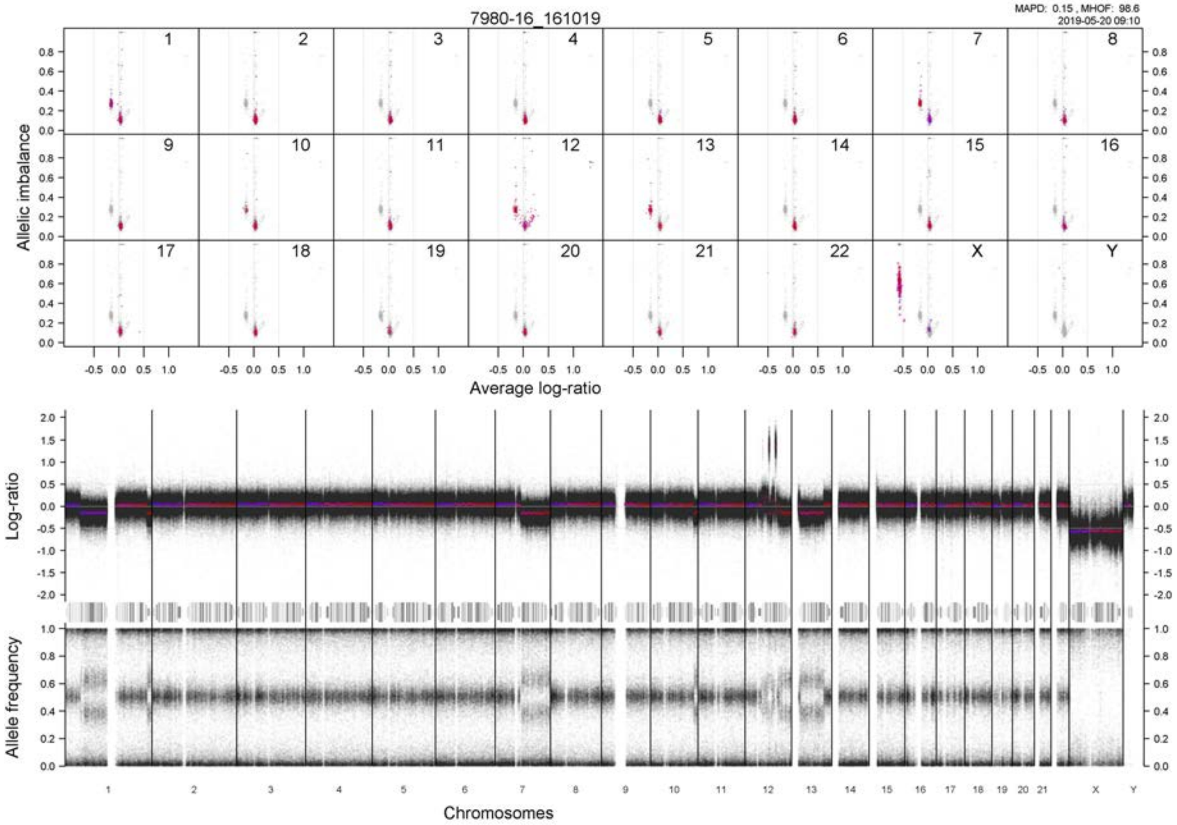

Surgical specimen

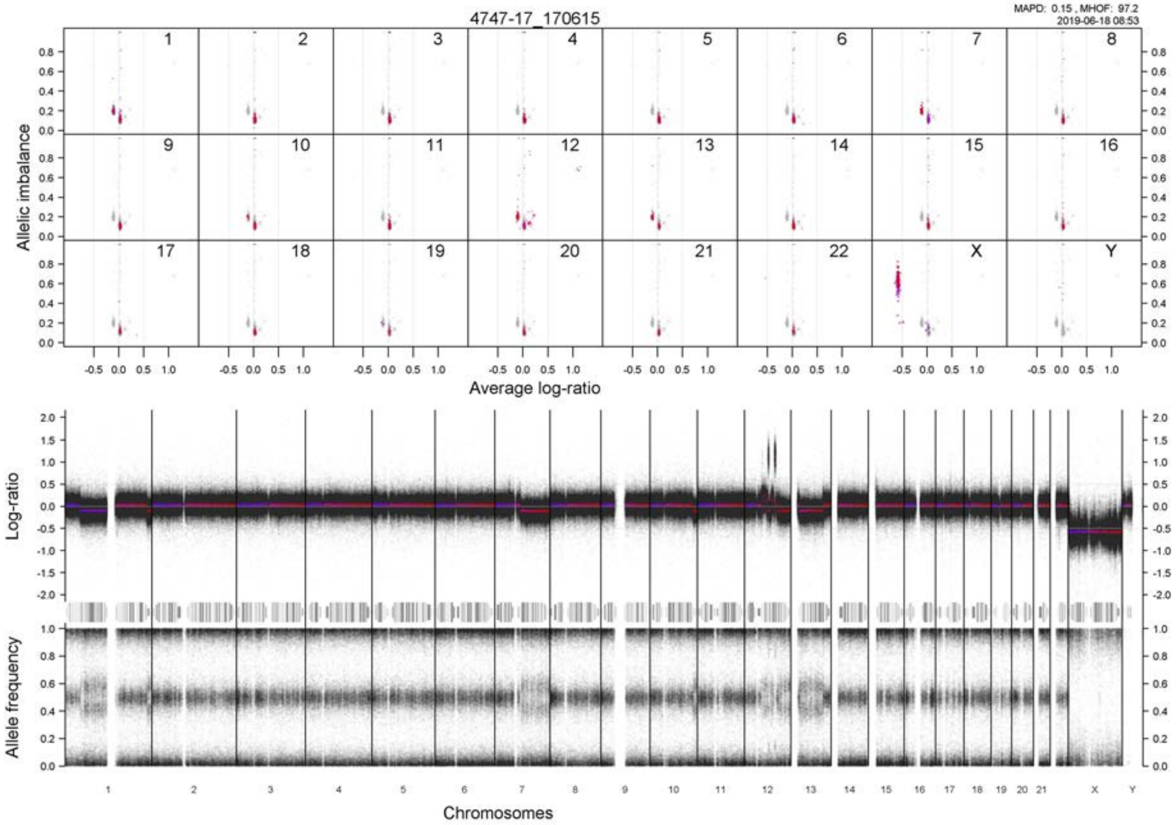

Case 26 (CNB)

Angiomyolipoma

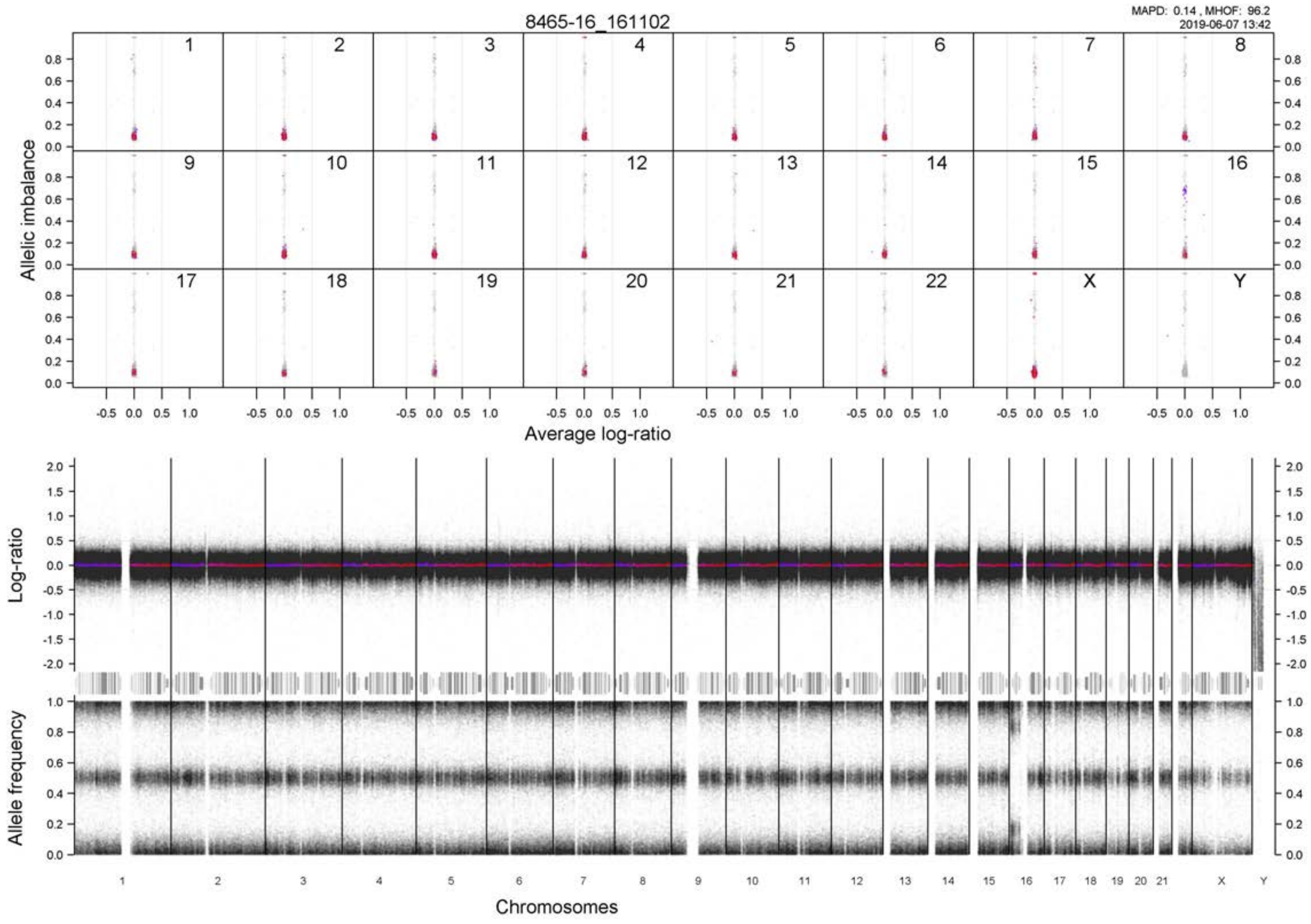

Case 27 (Surgical specimen)   Myxofibrosarcoma

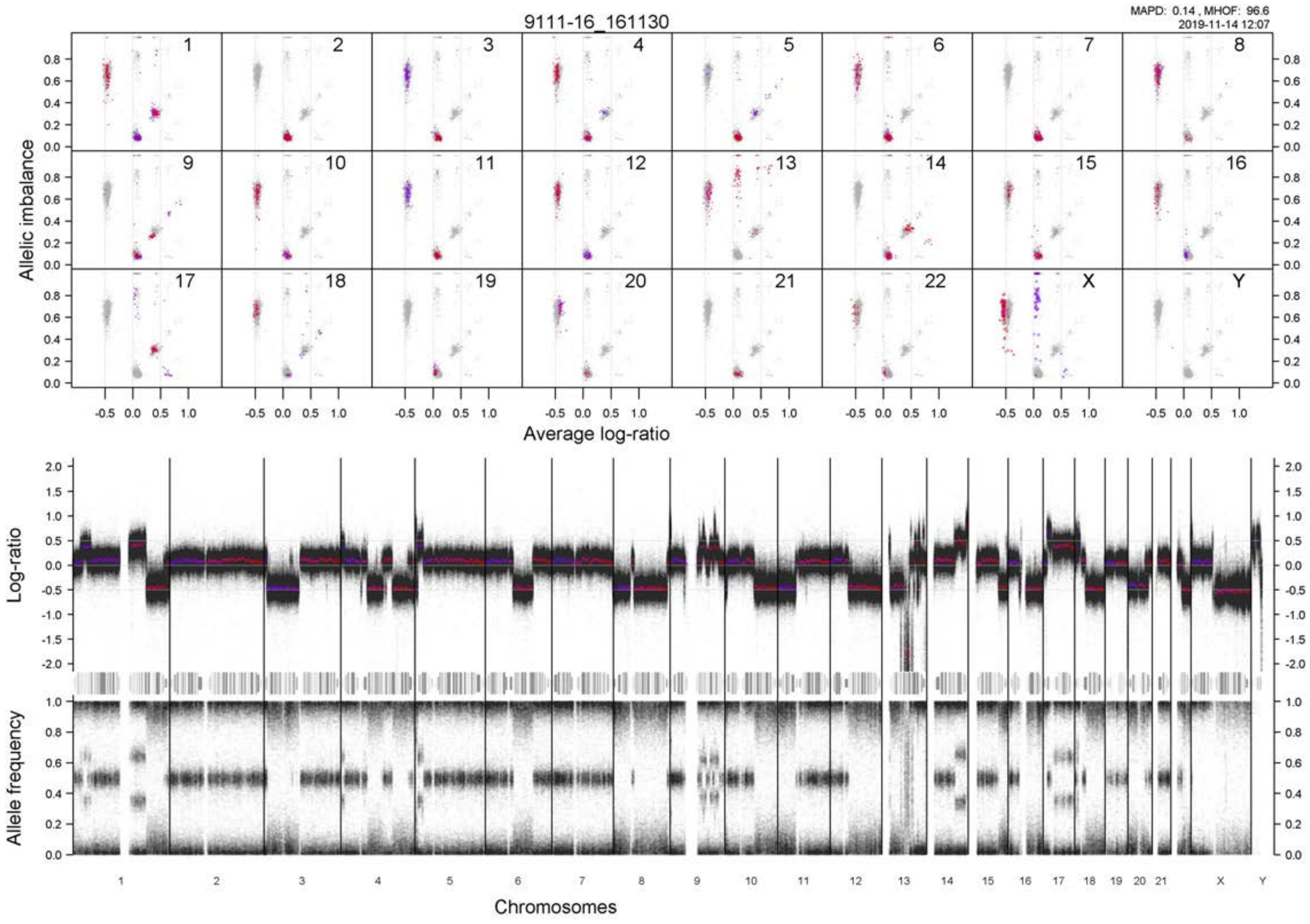

Case 28 (CNB)

Chordoma

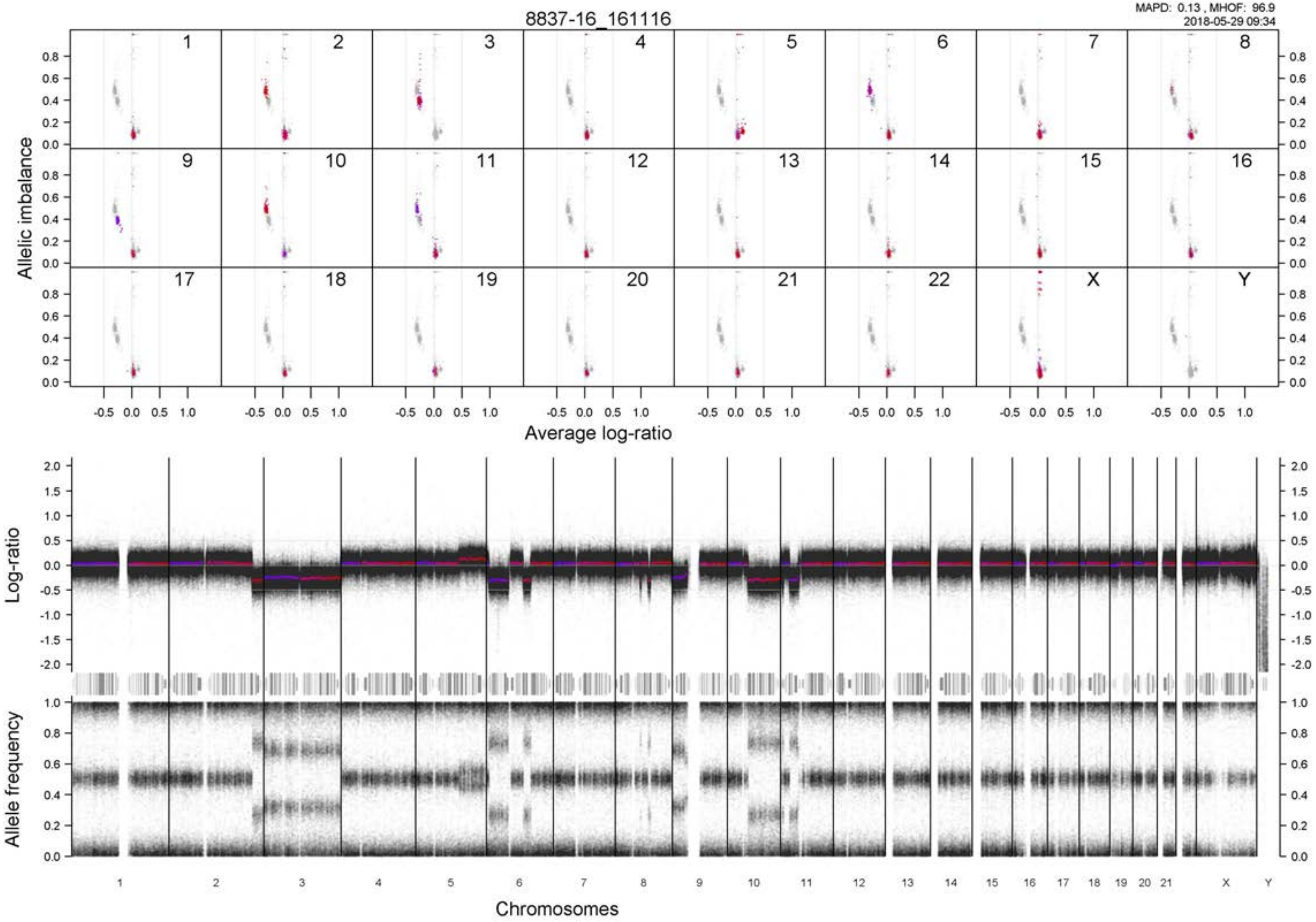

Case 31 (CNB)

Pleomorphic liposarcoma

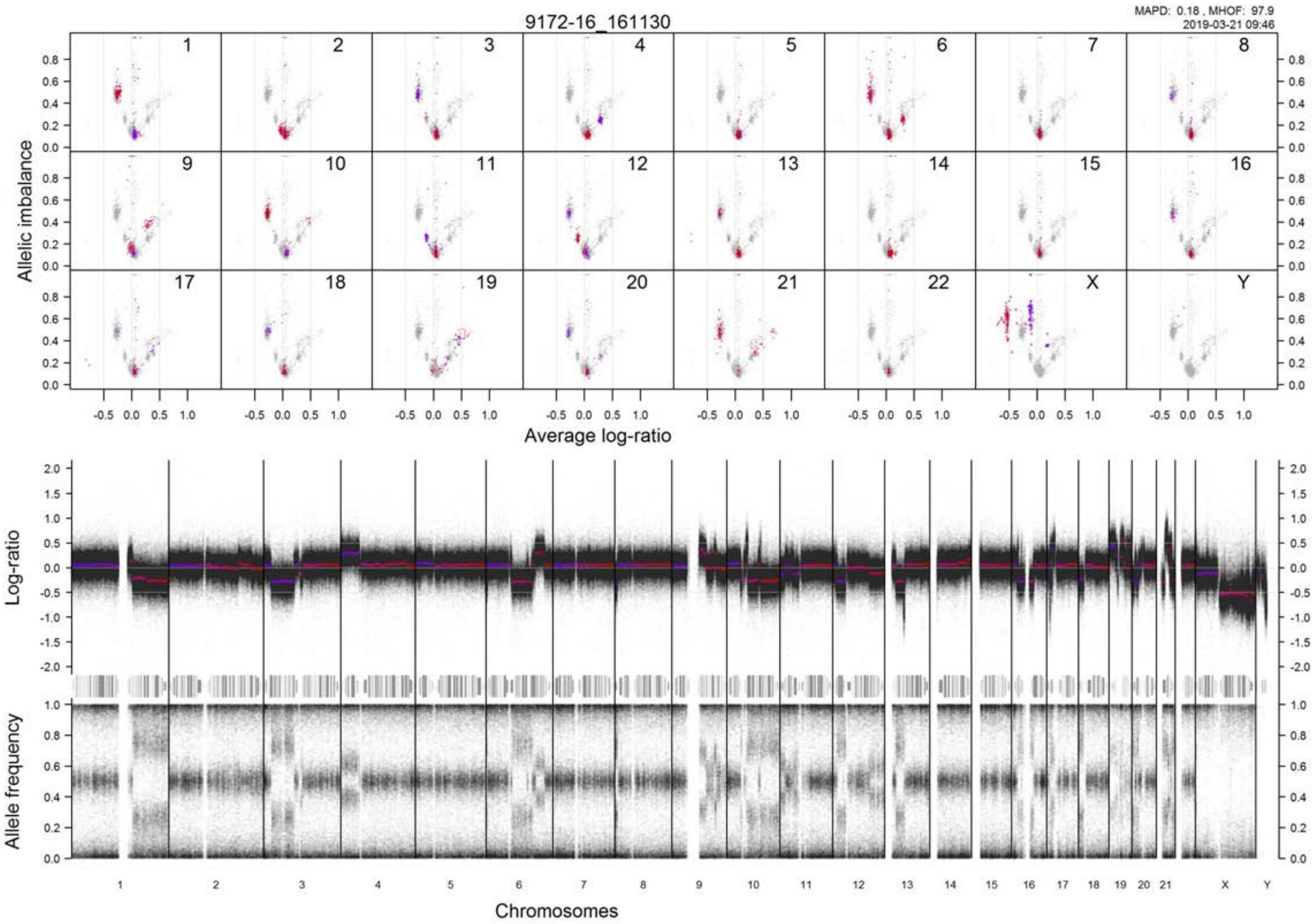

Case 32 (CNB) Inflammatory leiomyosarcoma

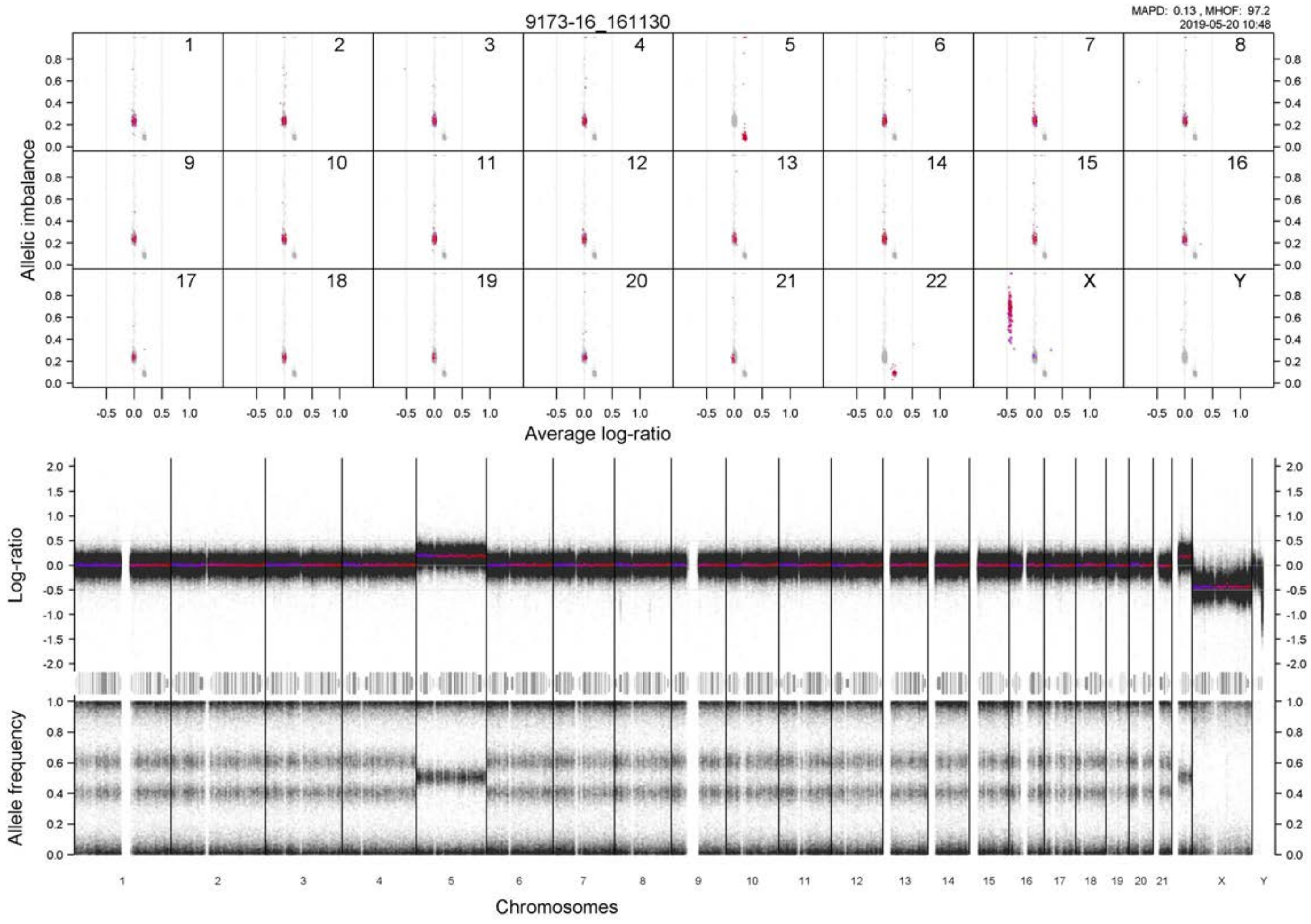

Case 33 (CNB)

Leiomyosarcoma

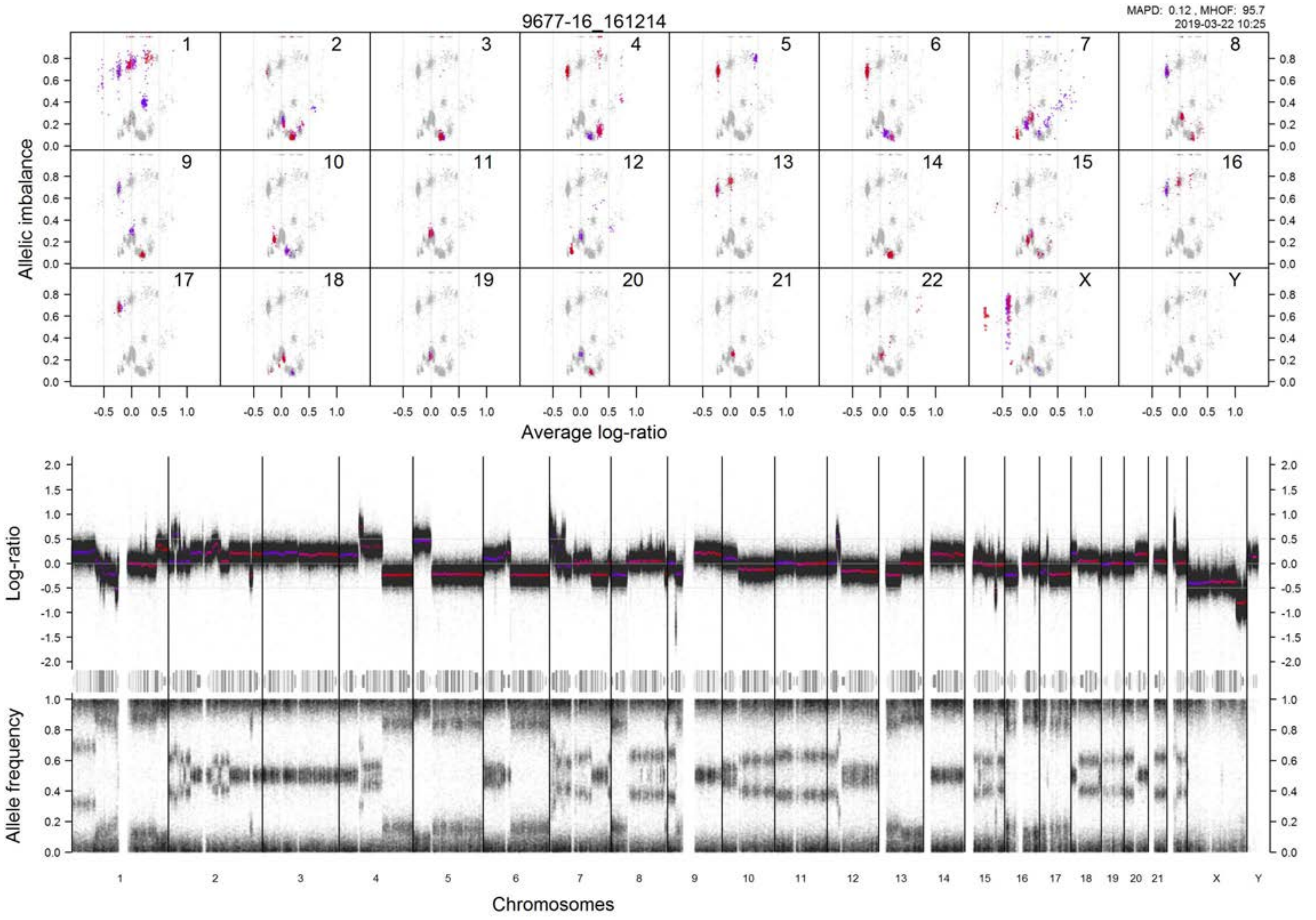

Case 34

Myxofibrosarcoma

CNB

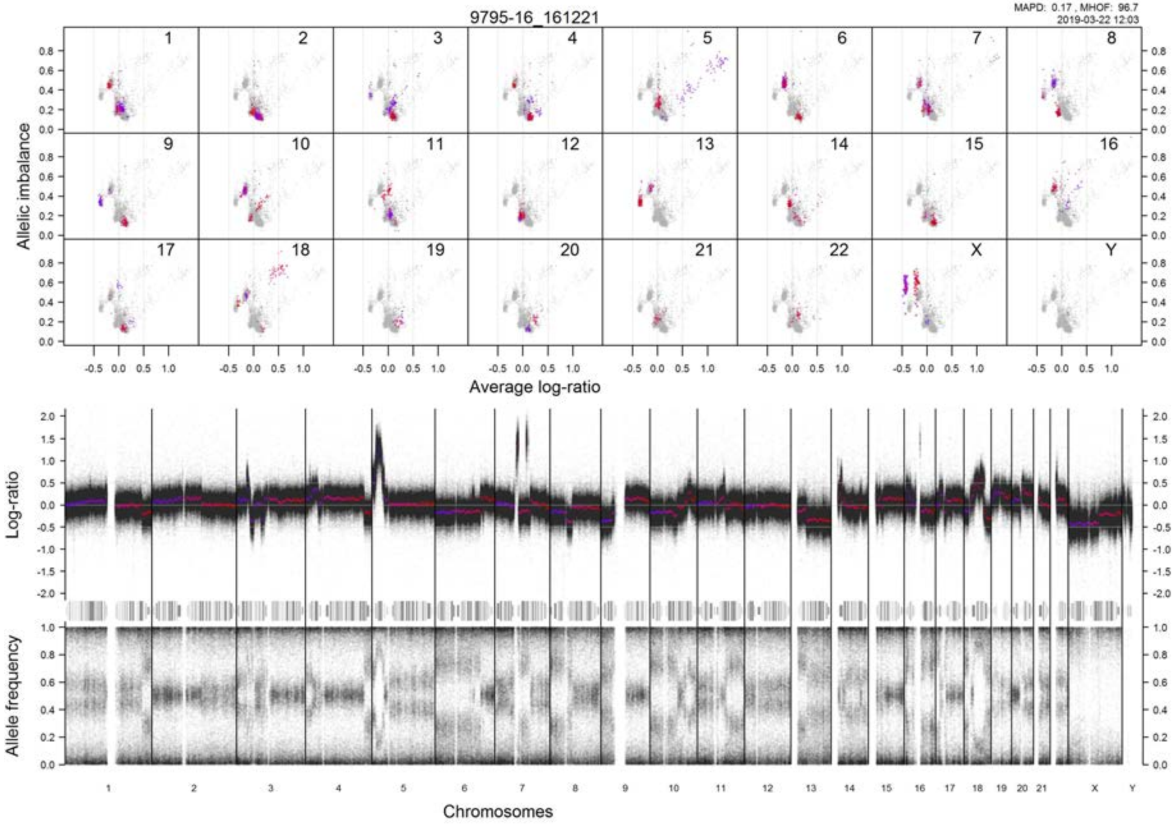

Surgical specimen

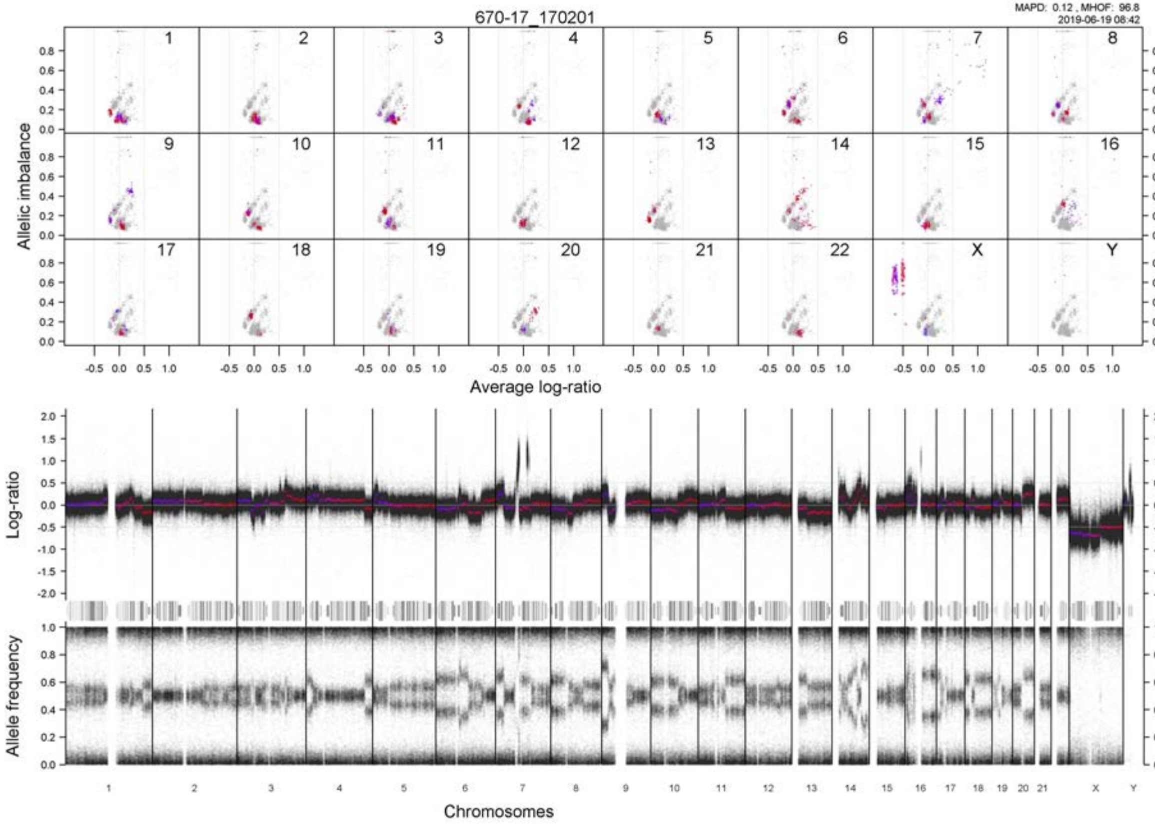

Case 36 (CNB)

Osteosarcoma

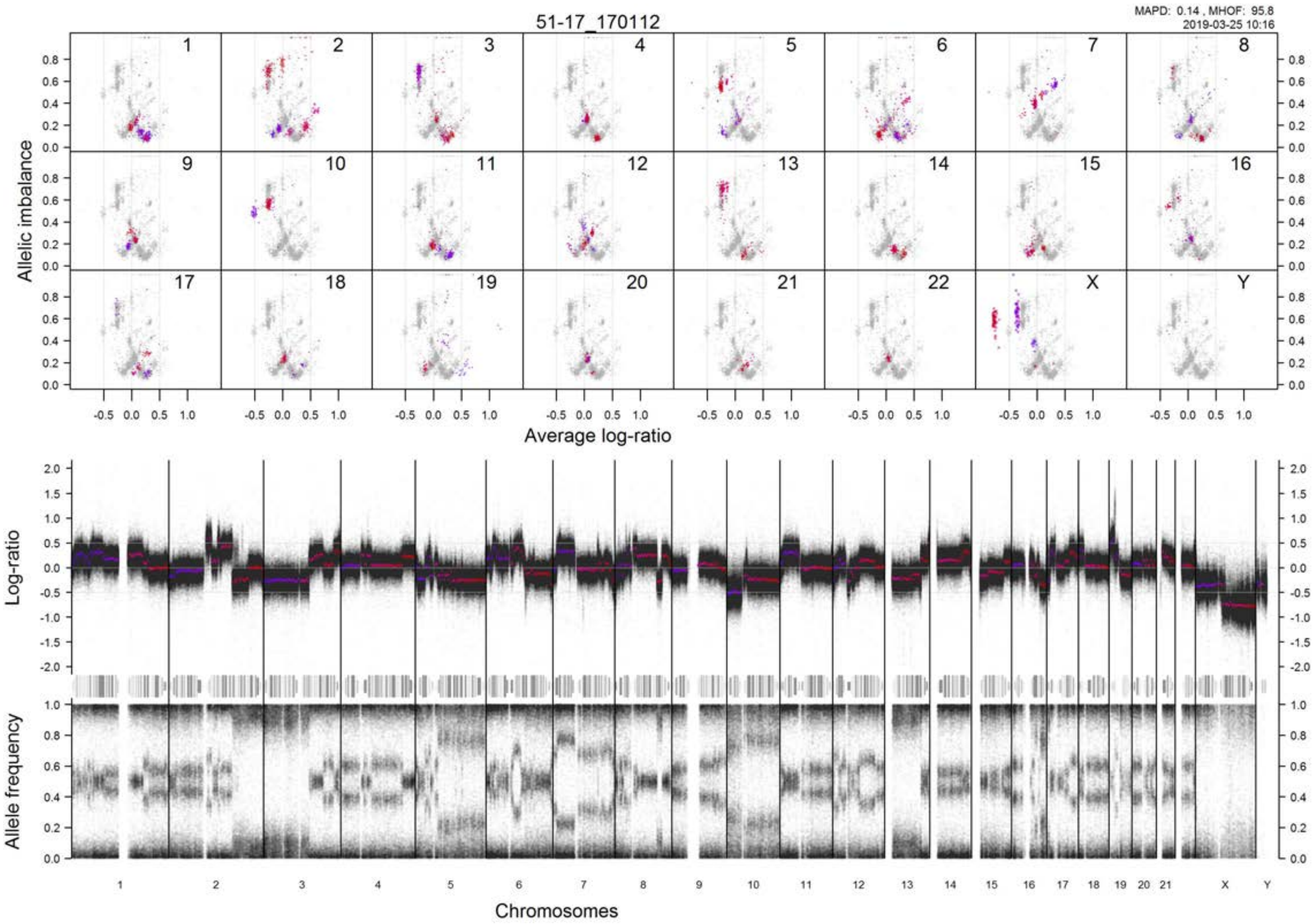

Case 38 (Surgical) Well-differentiated liposarcoma

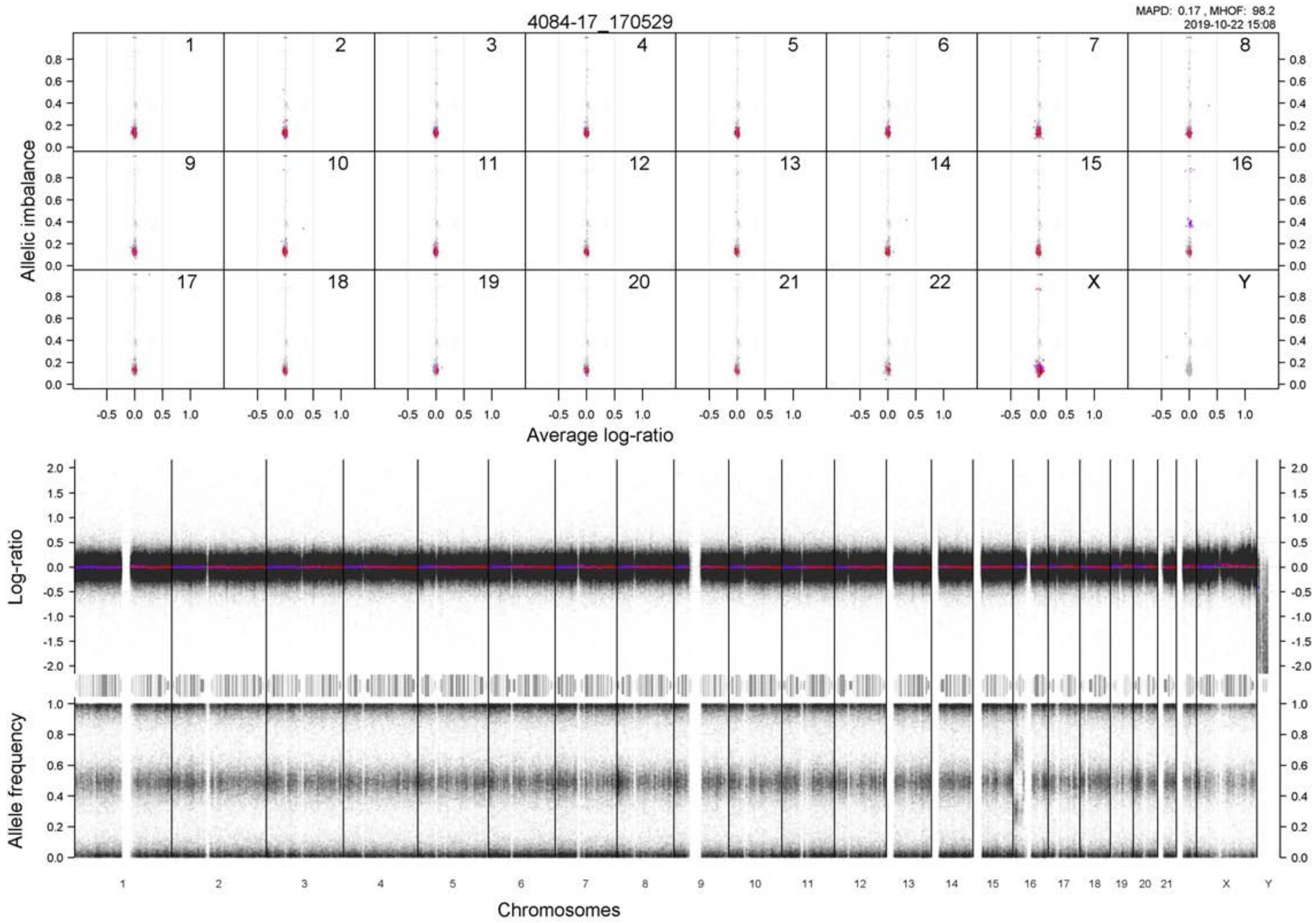

Case 40 (CNB)

Leiomyosarcoma (metastasis)

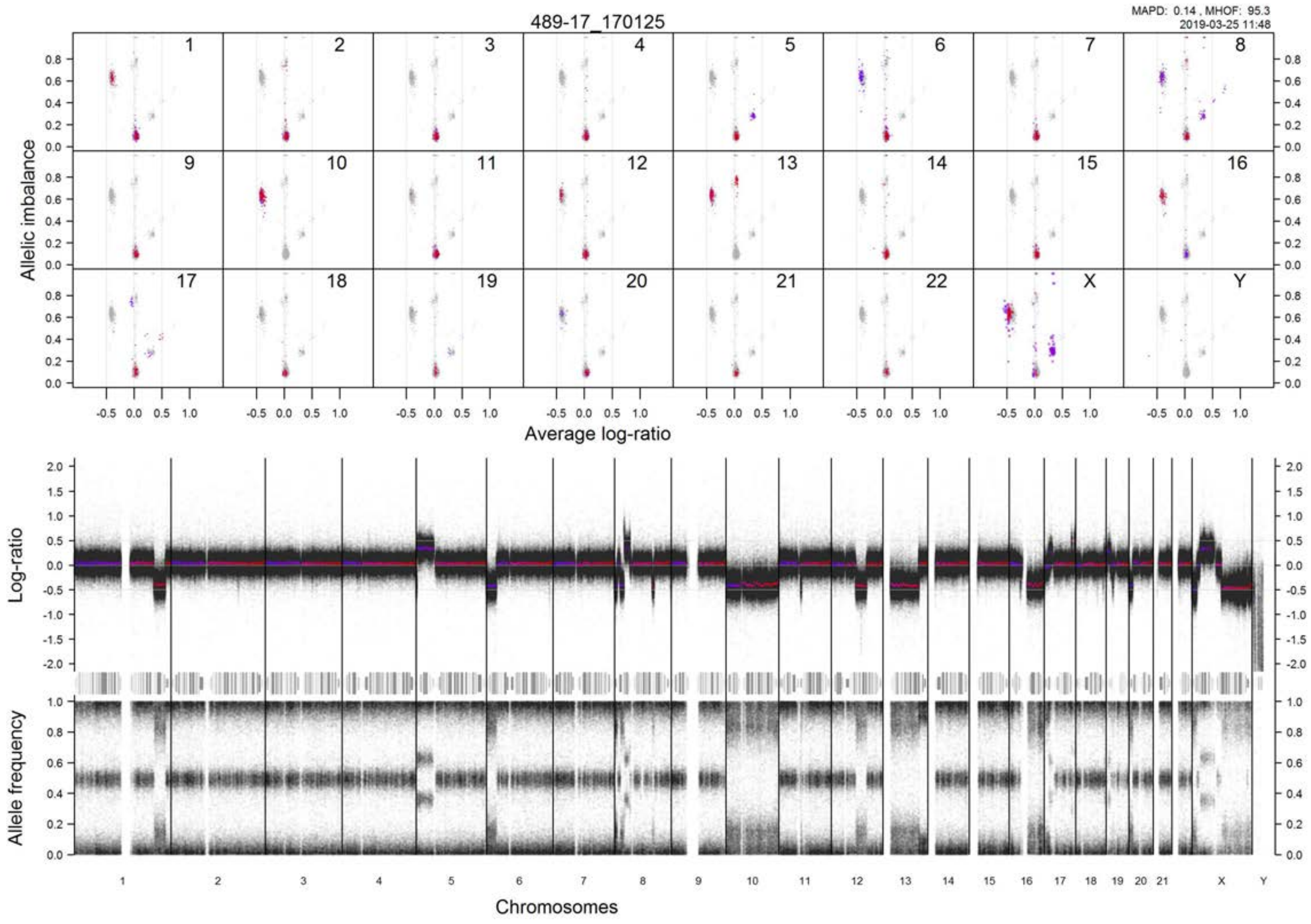

Case 41

UPS

CNB

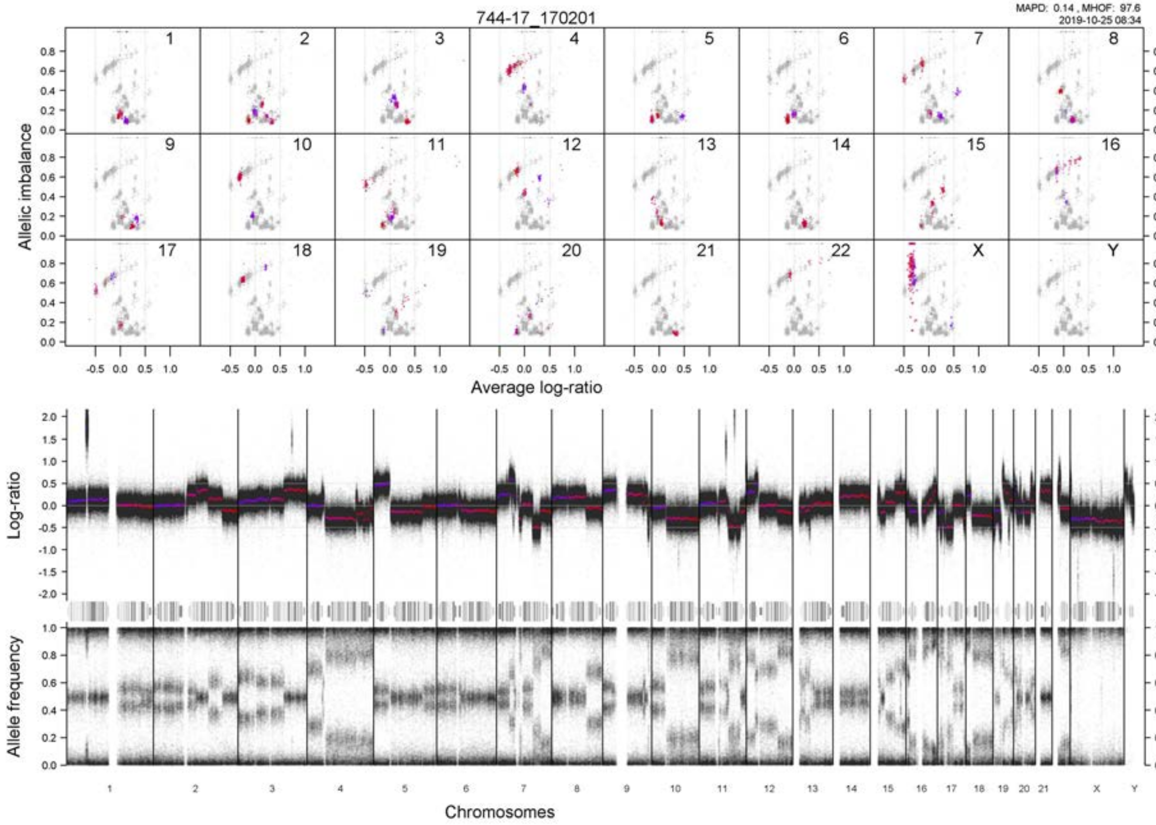

Surgical specimen

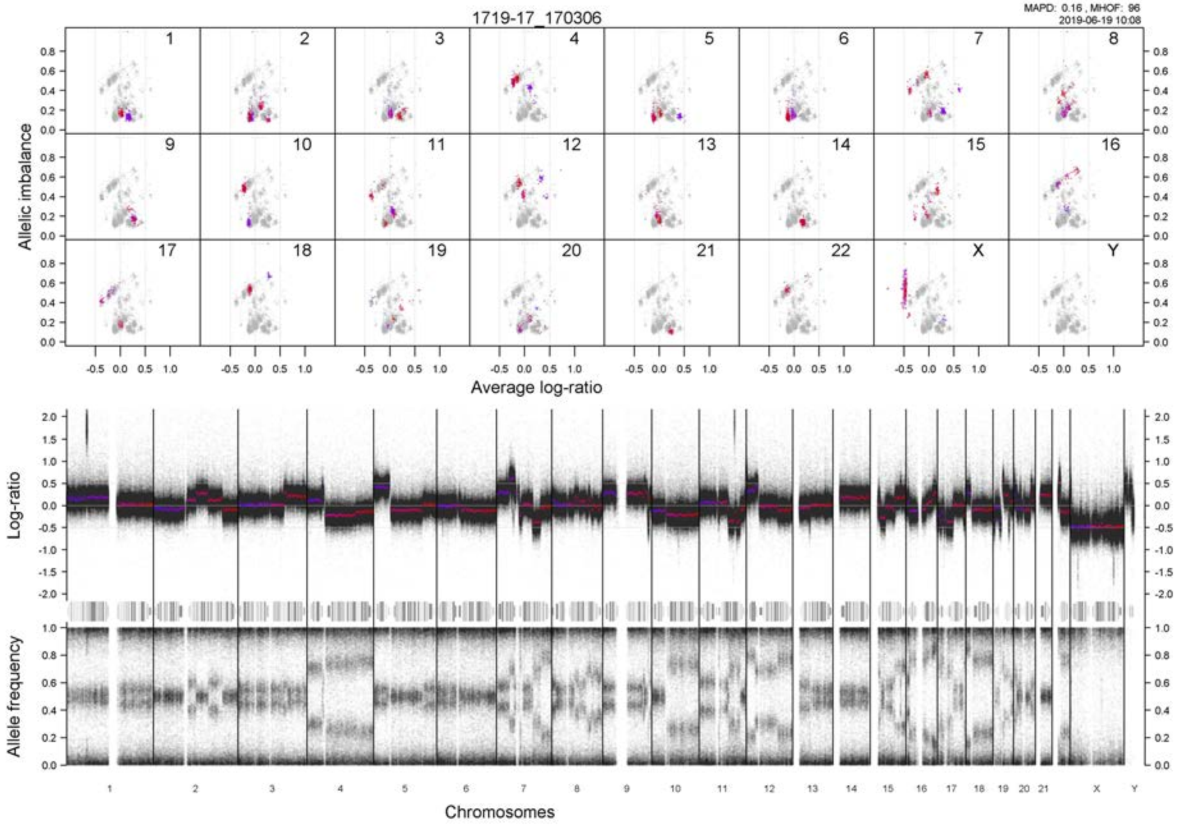

Case 43 (CNB)

Fatty tissue

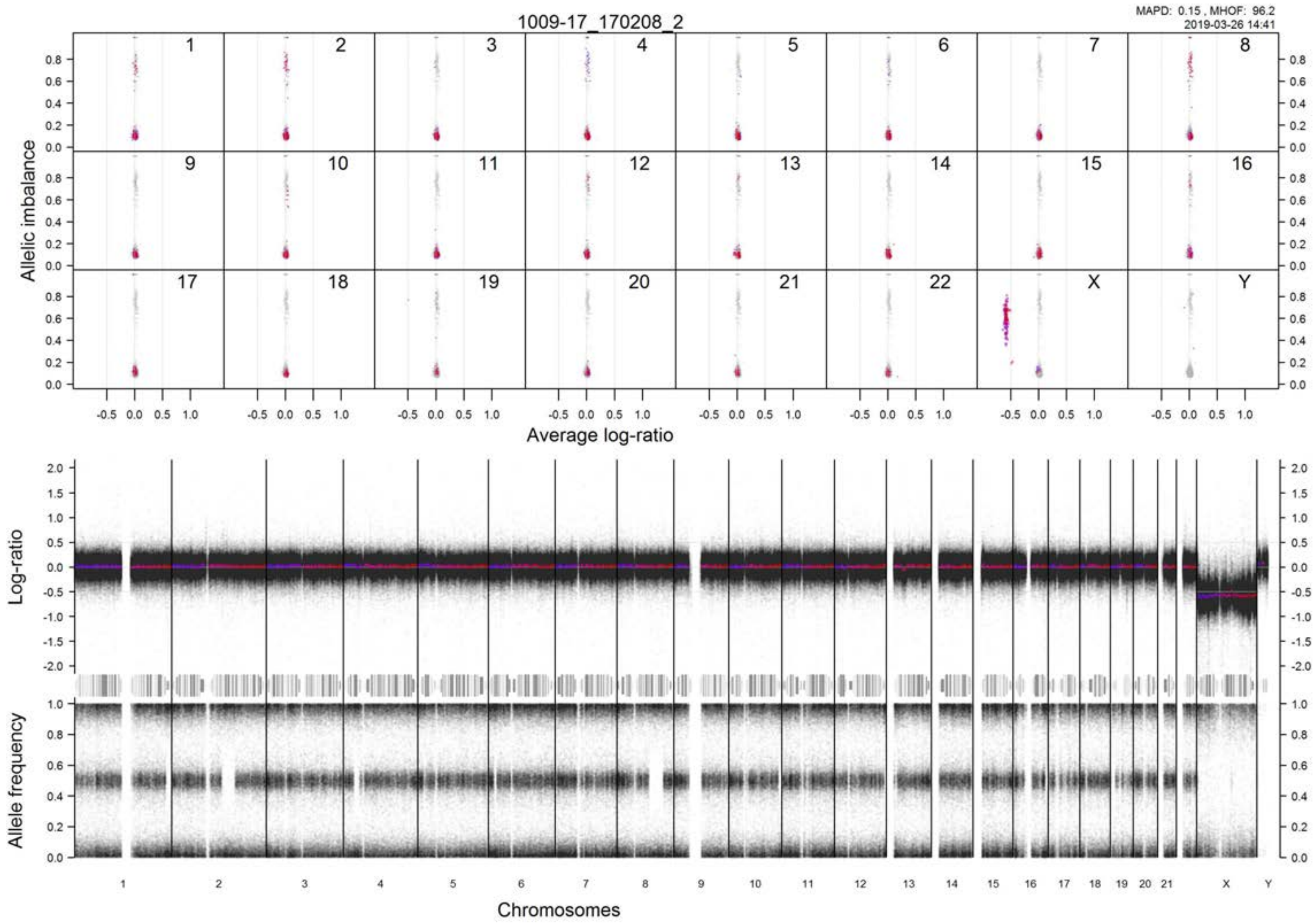

Case 44

Mesenchymal chrondrosarcoma

CNB

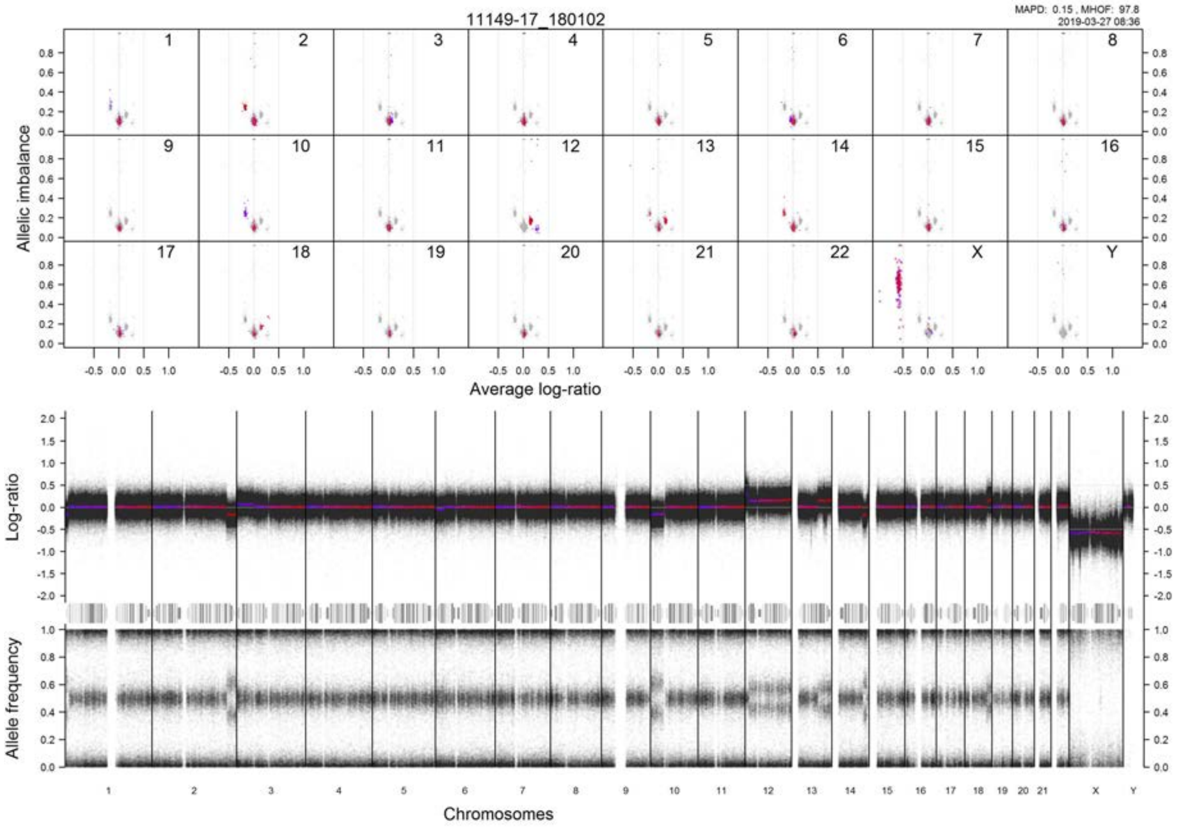

Surgical specimen

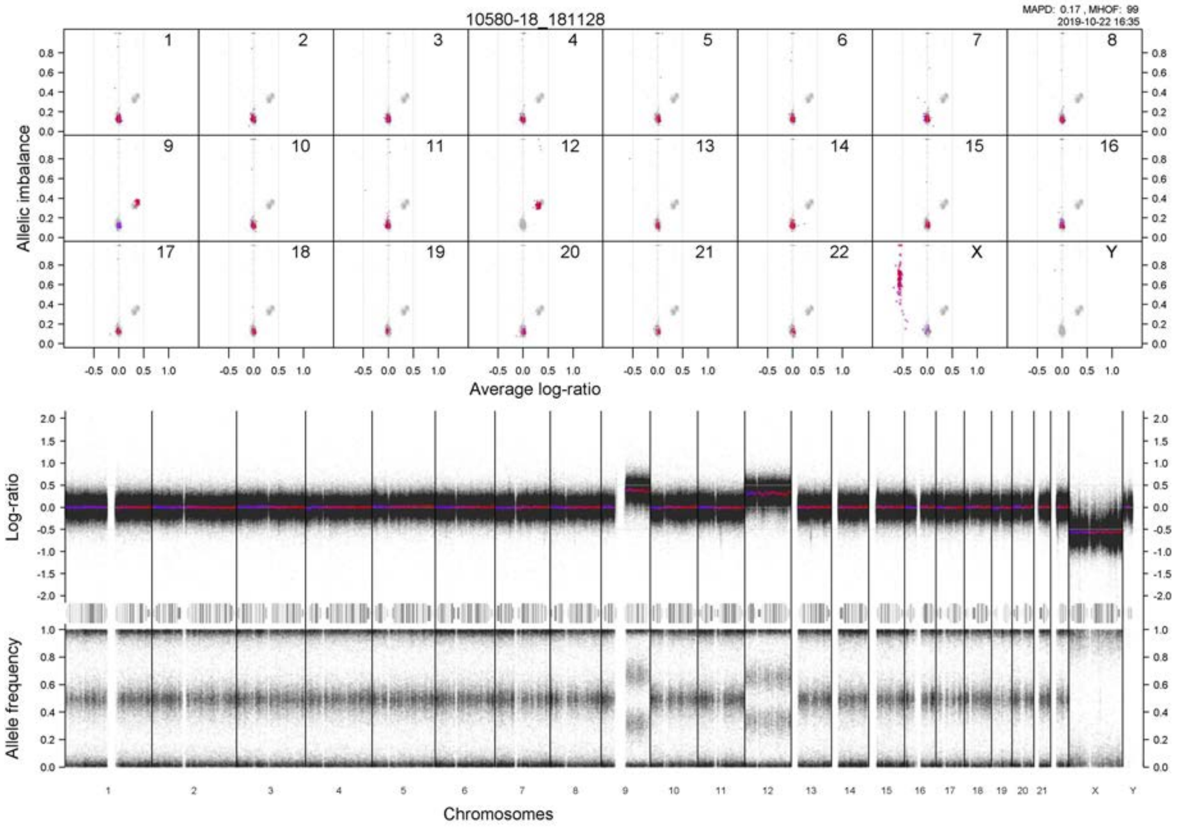

Case 47 (CNB) Desmoid fibromatosis

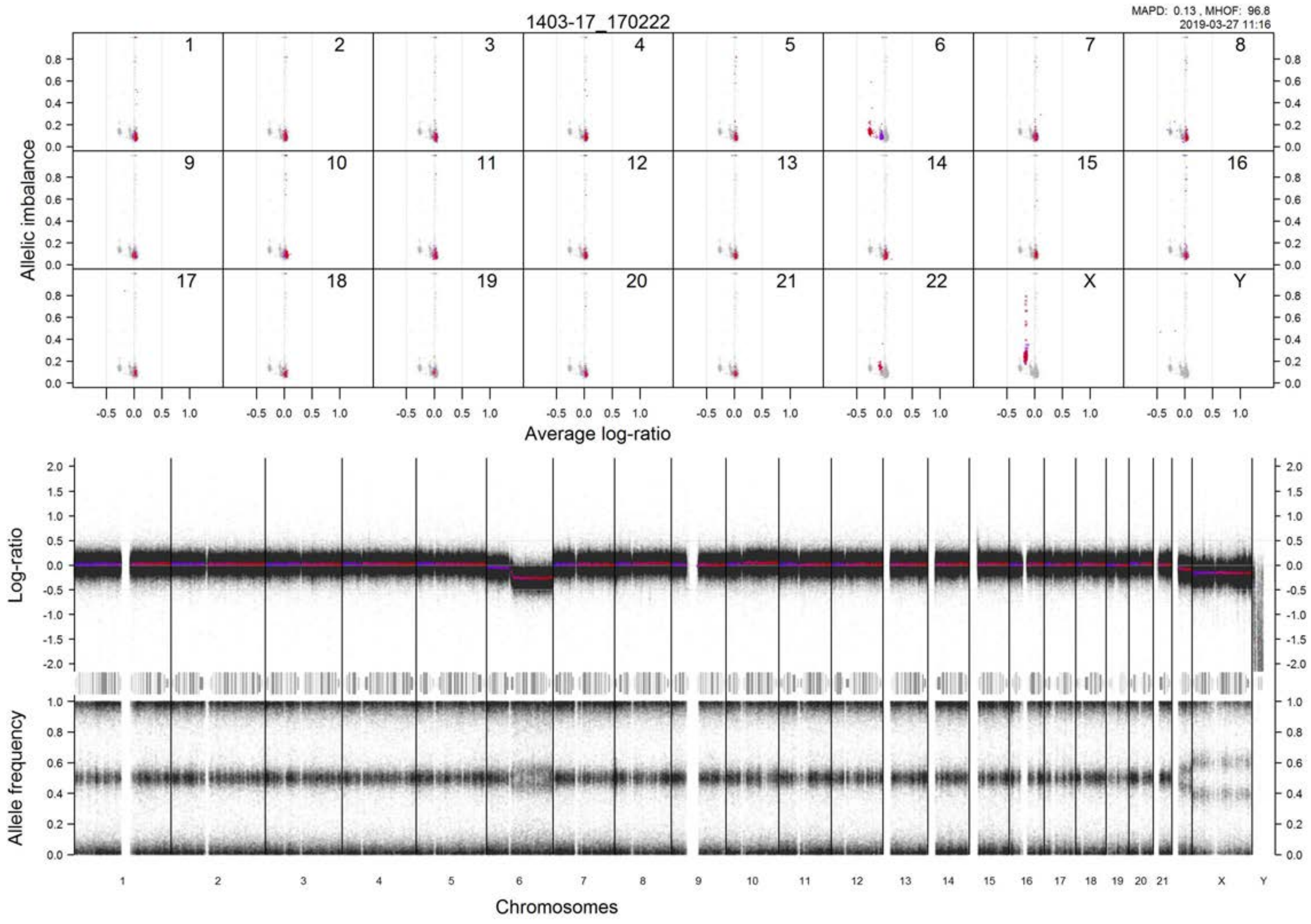

Case 50 (CNB) Well-differentiated liposarcoma

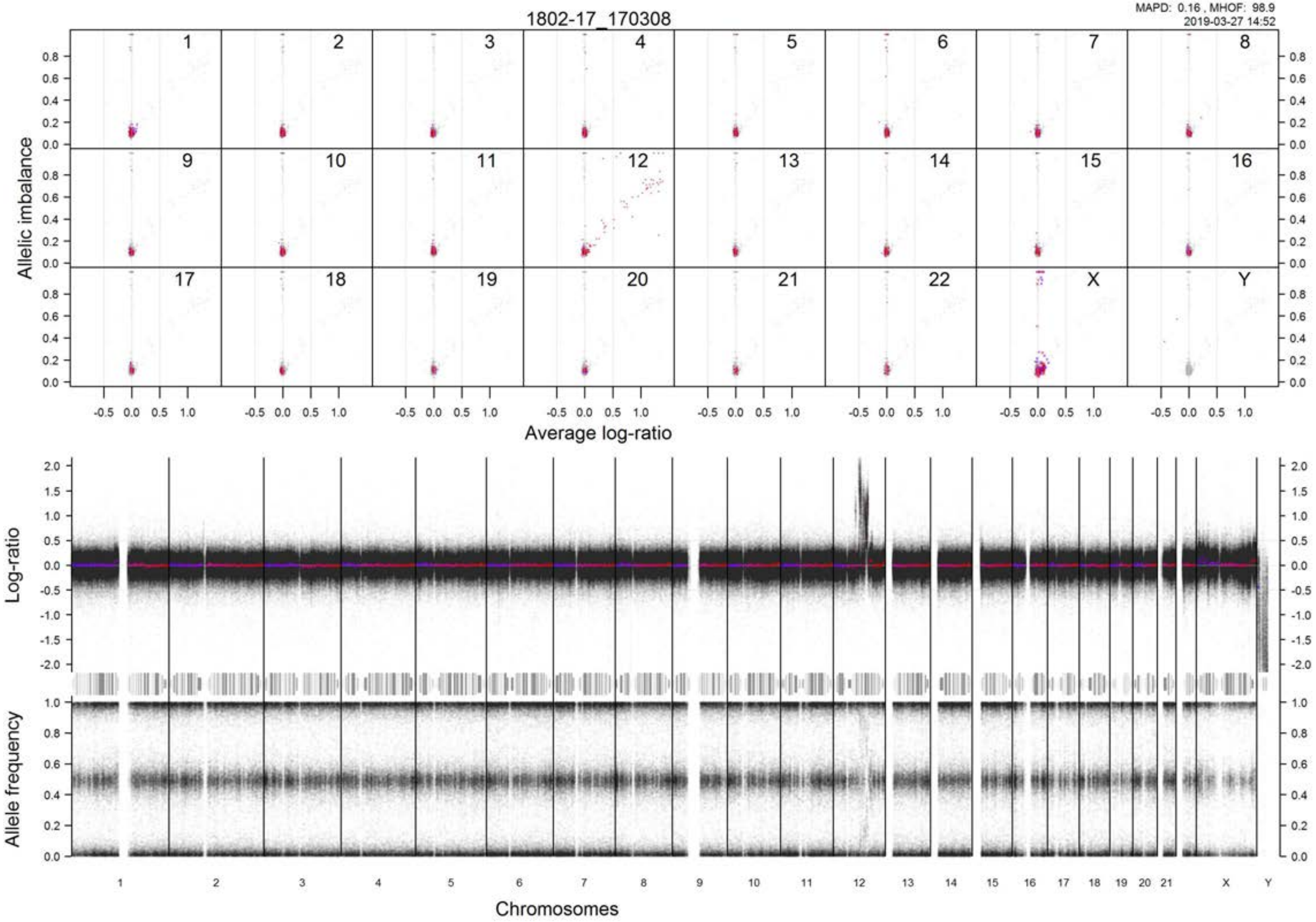

Case 52 (CNB)

Lipoma

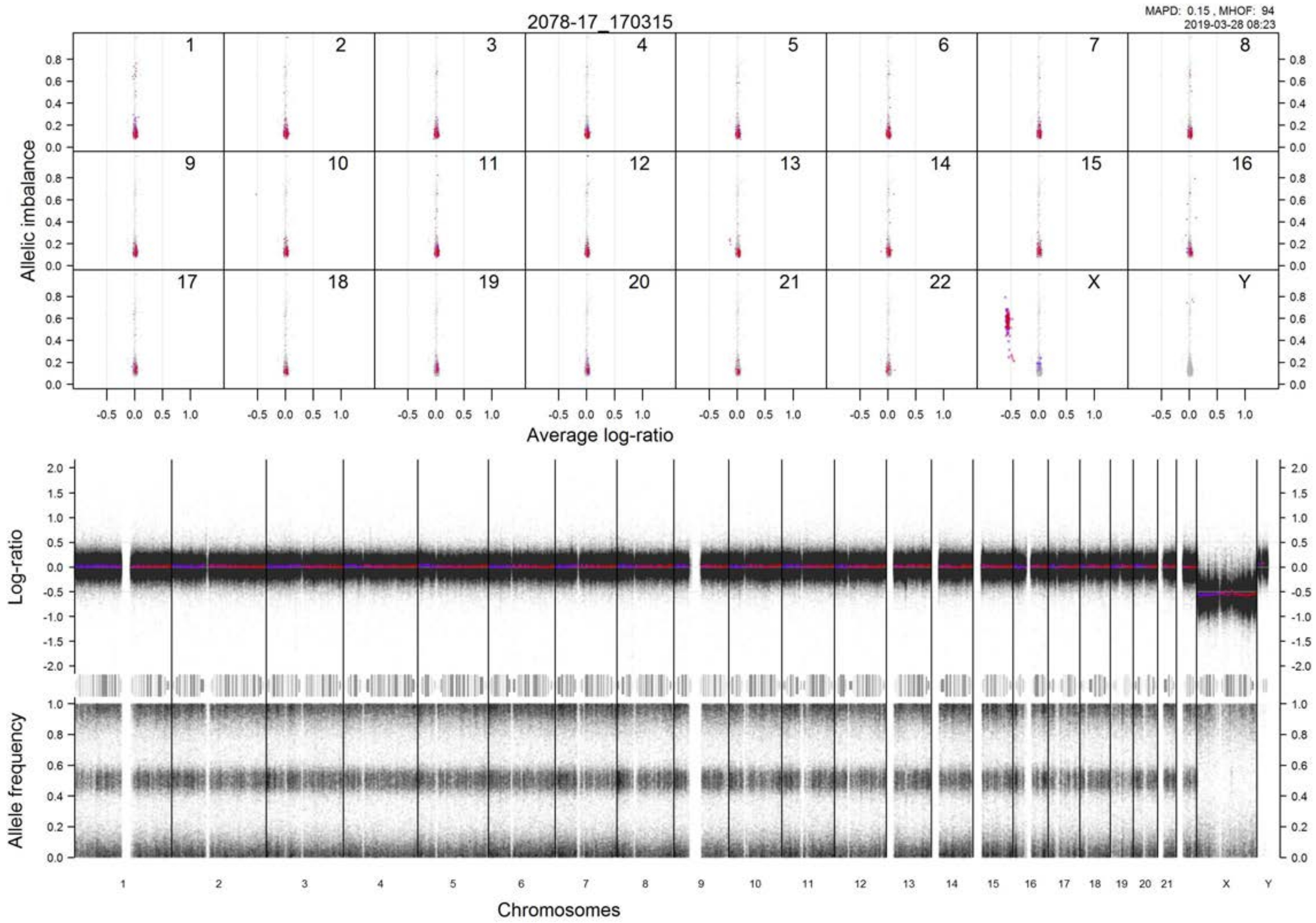

Case 55 (Surgical)

Chondrosarcoma, conventional

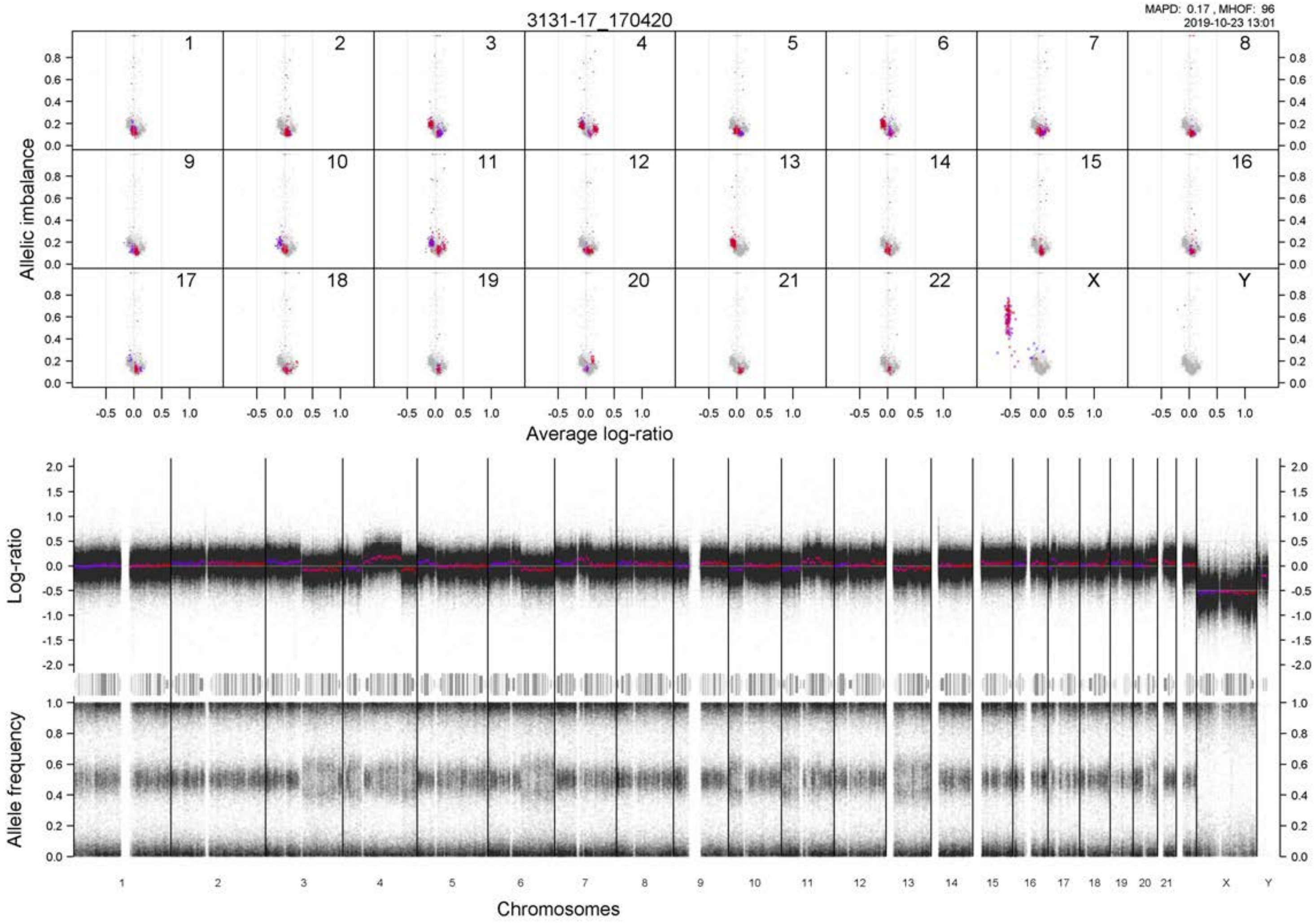

Case 56

UPS

CNB

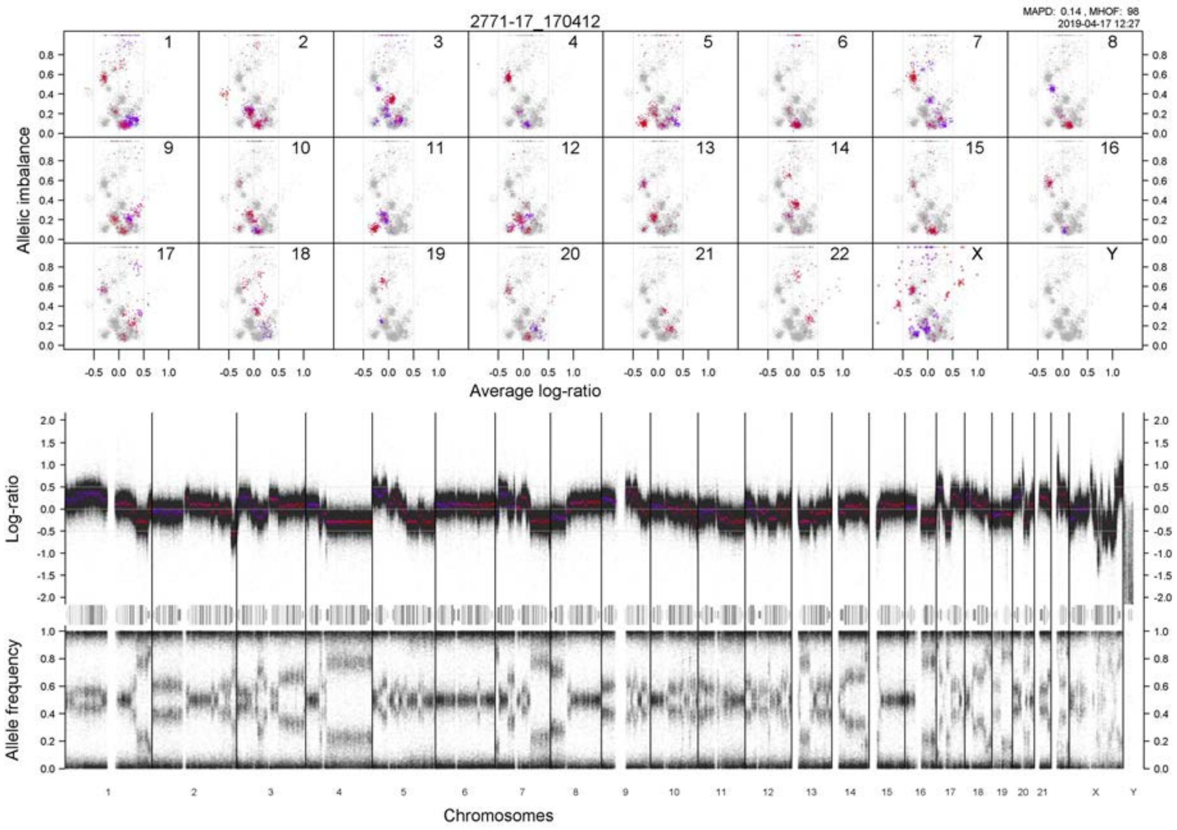

Surgical specimen

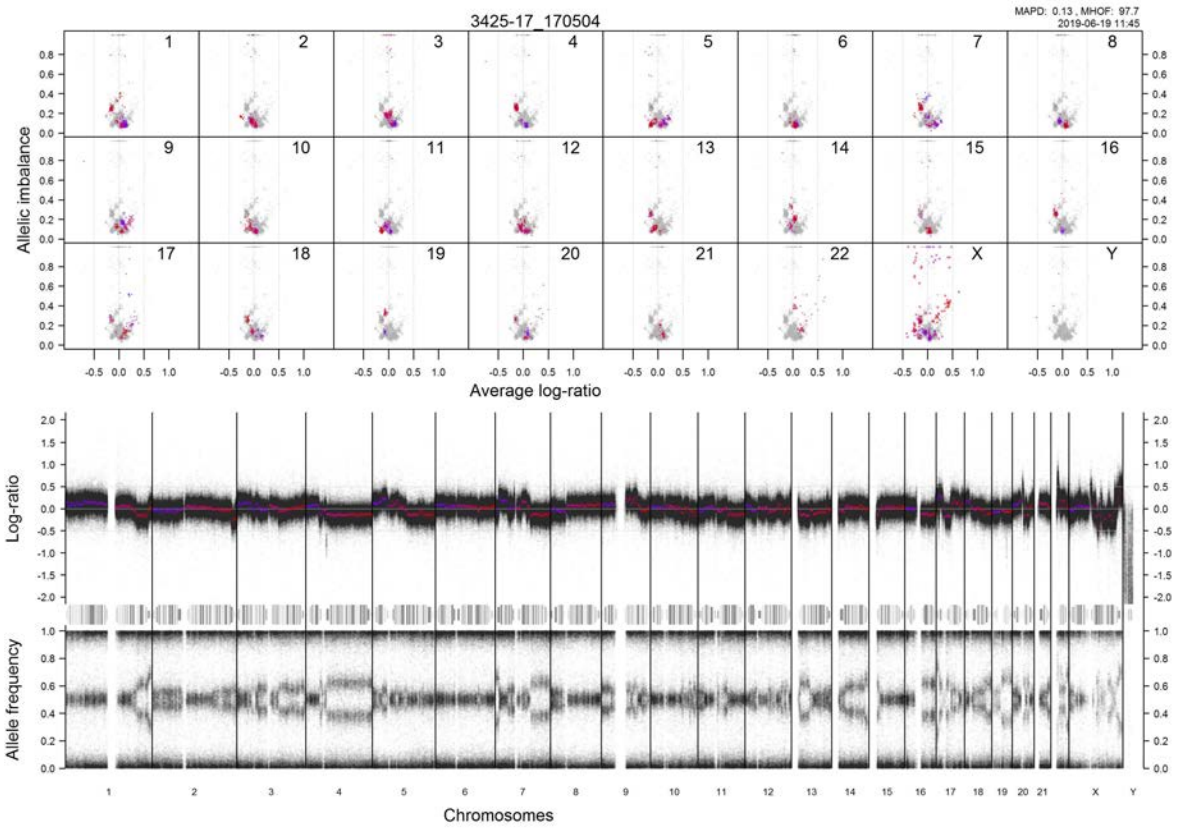

Case 57

UPS

CNB

Surgical specimen

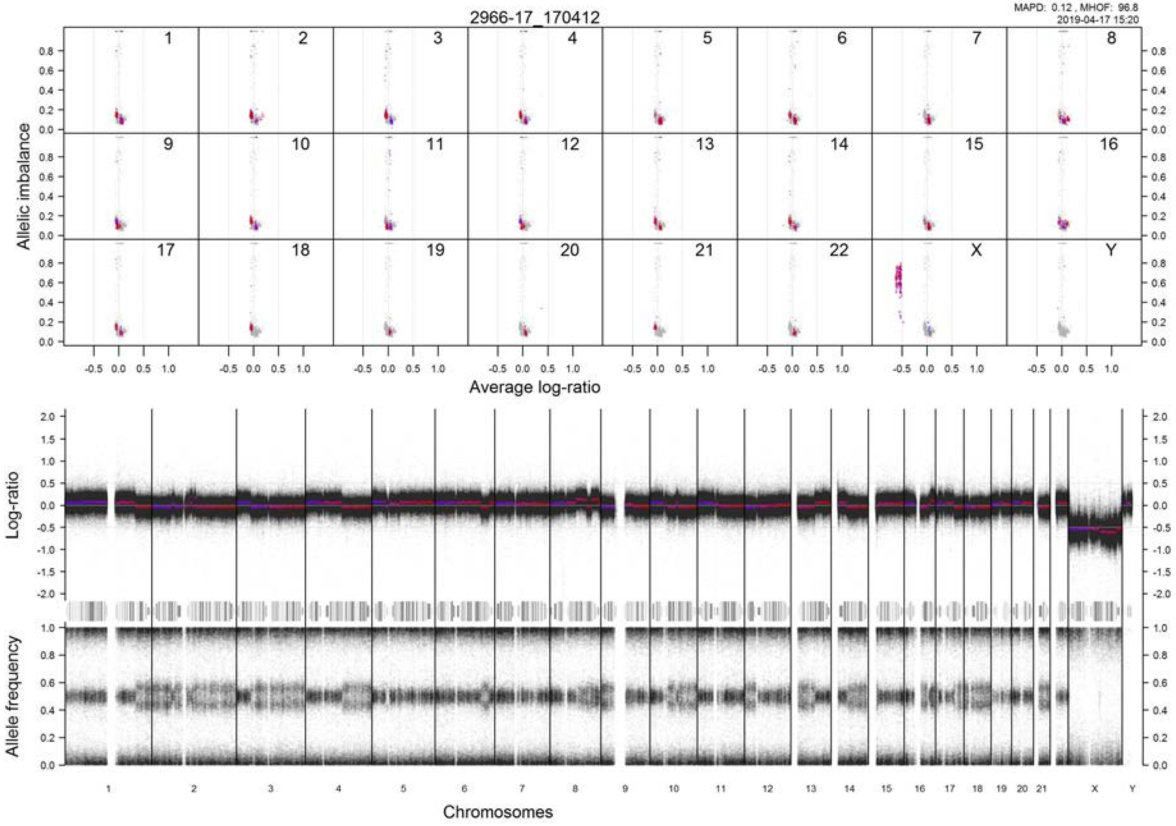

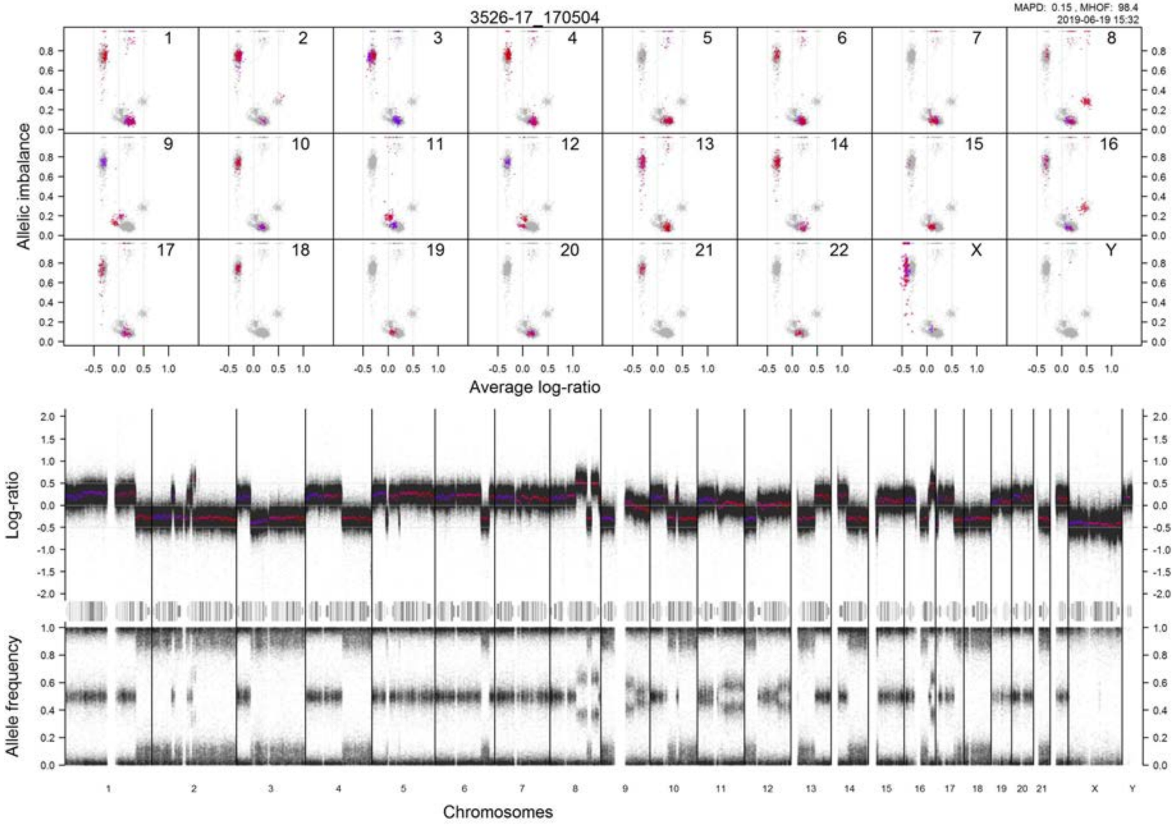

Case 58 (Surgical)

Chondrosarcoma, conventional

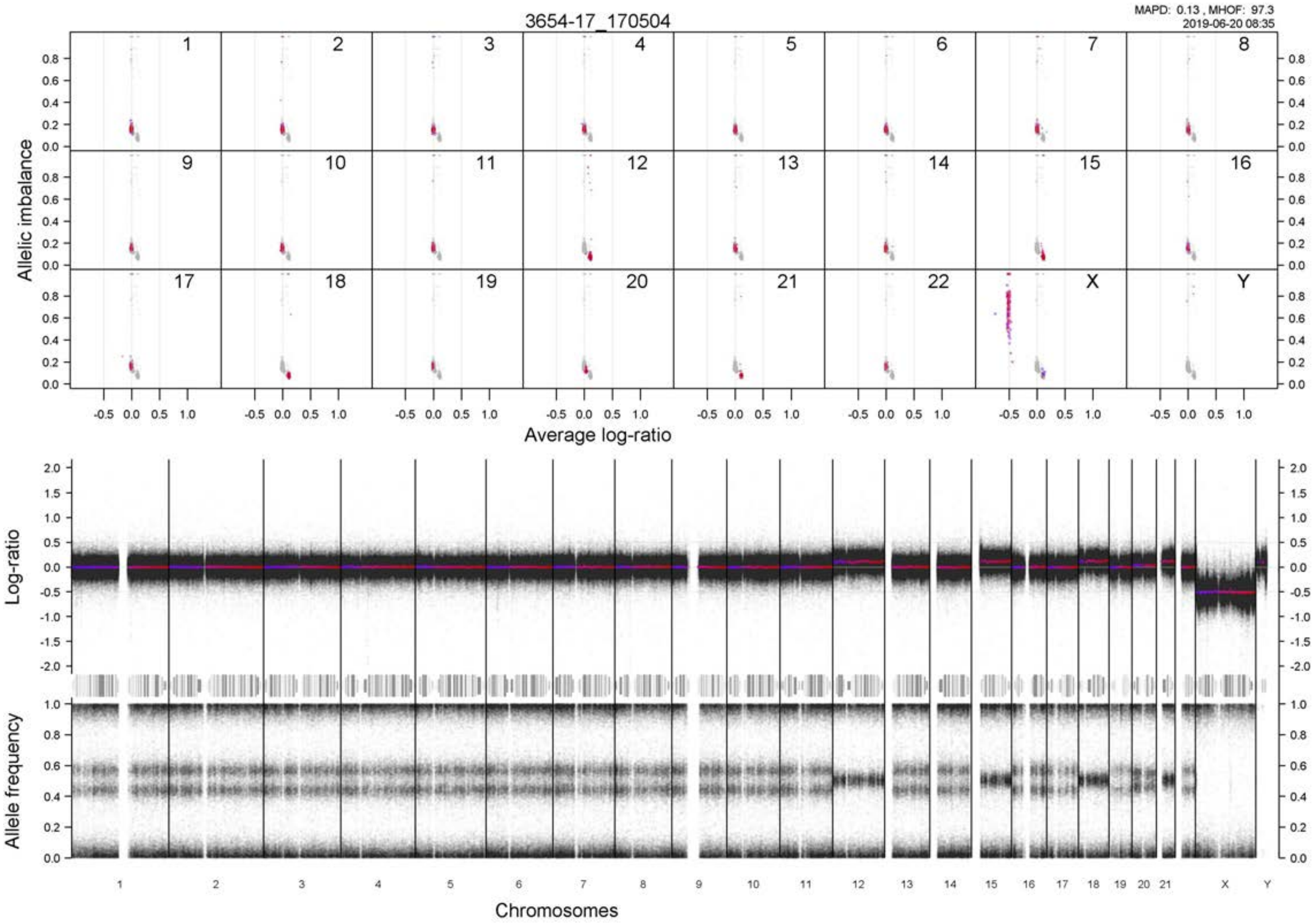

Case 59 (CNB) Giant cell tumor of bone

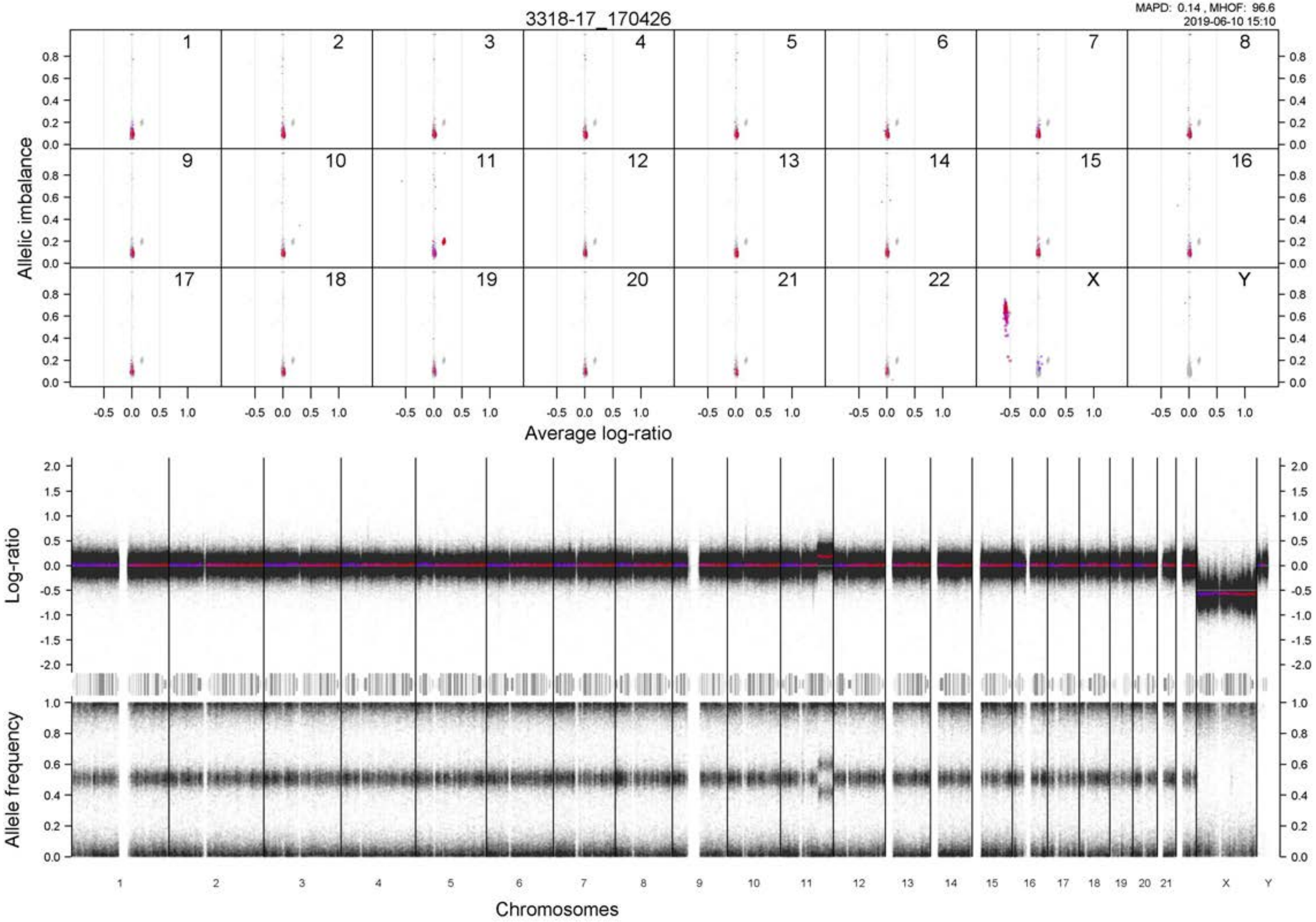

Case 60 (CNB) Ossifying fibromyxoid tumor

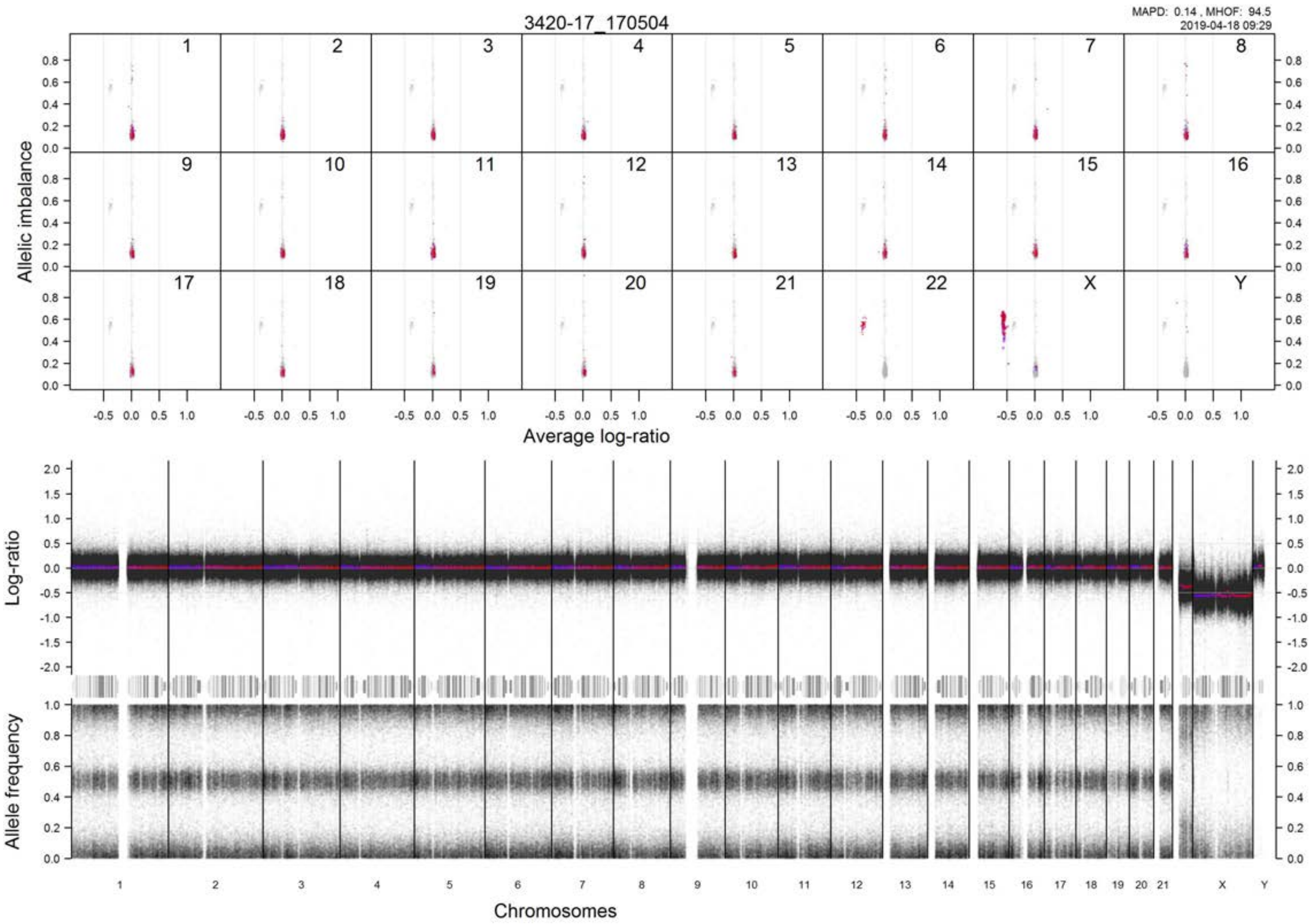

Case 61 Pleomorphic liposarcoma

CNB

Surgical specimen

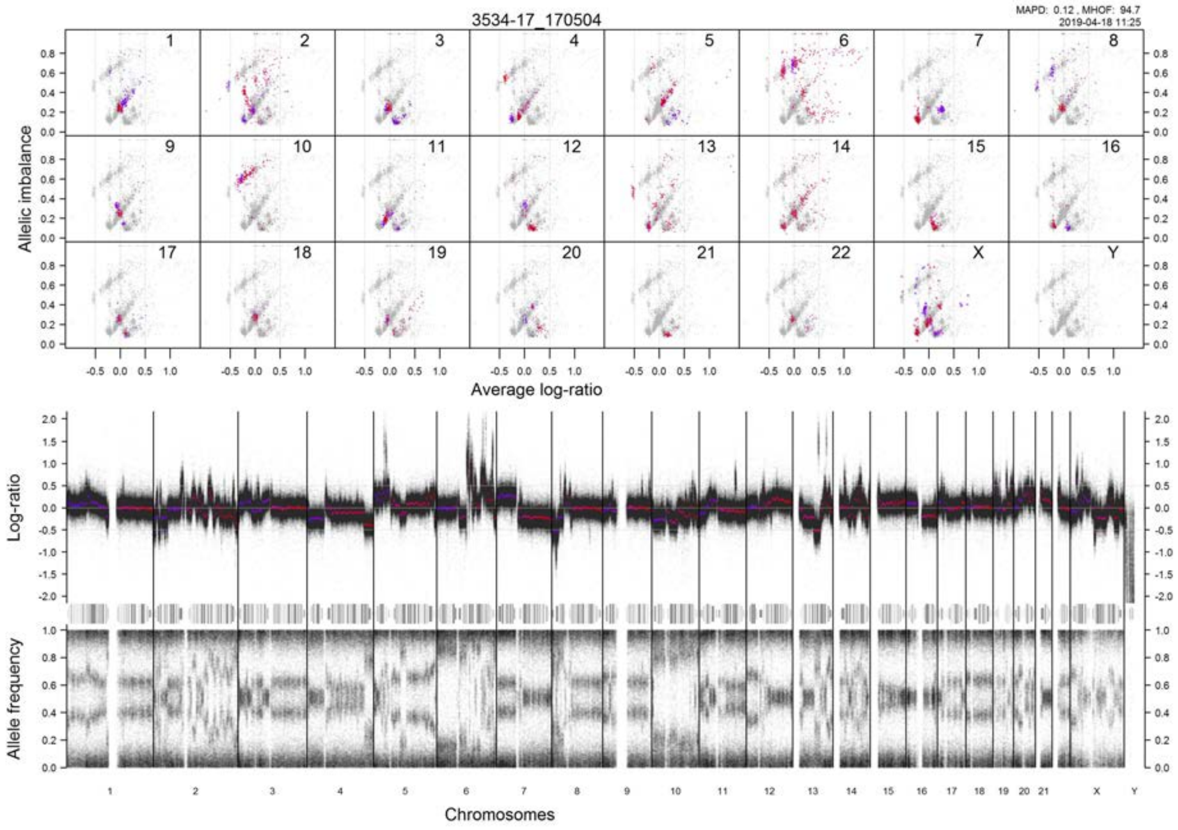

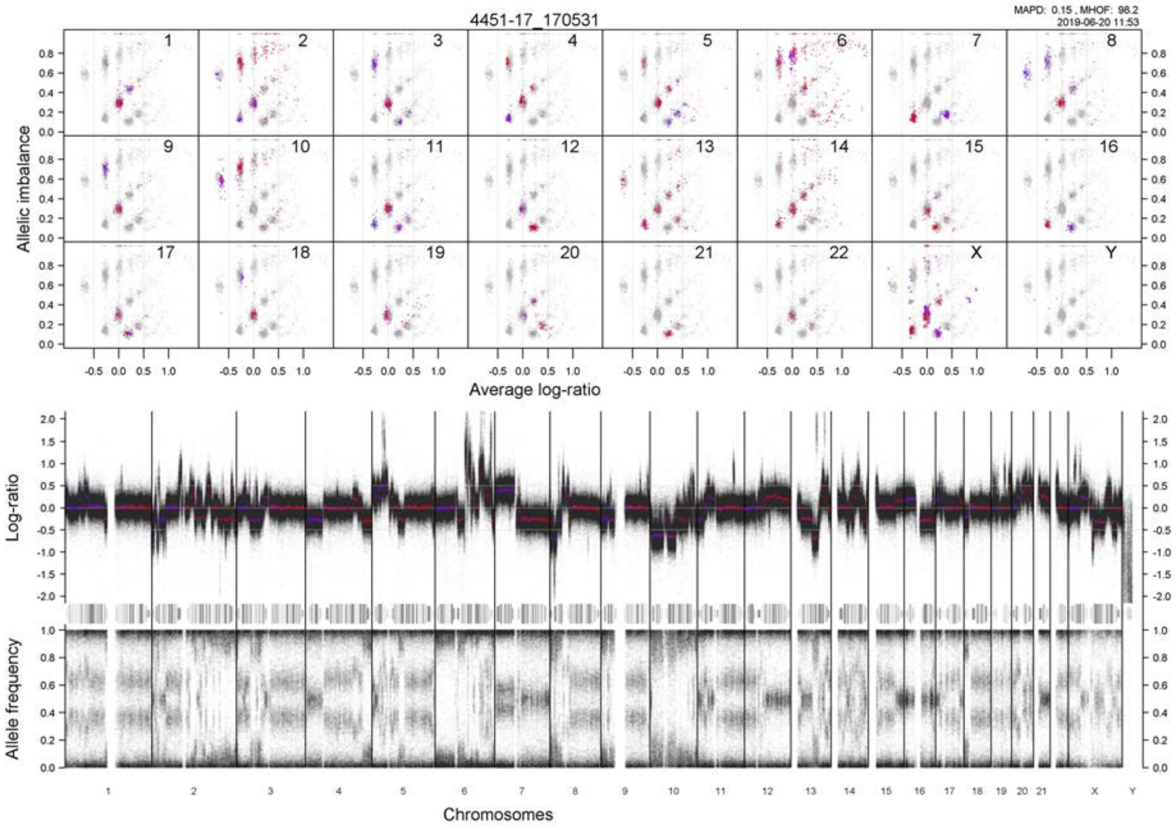

Case 65

Dedifferentiated liposarcoma

CNB

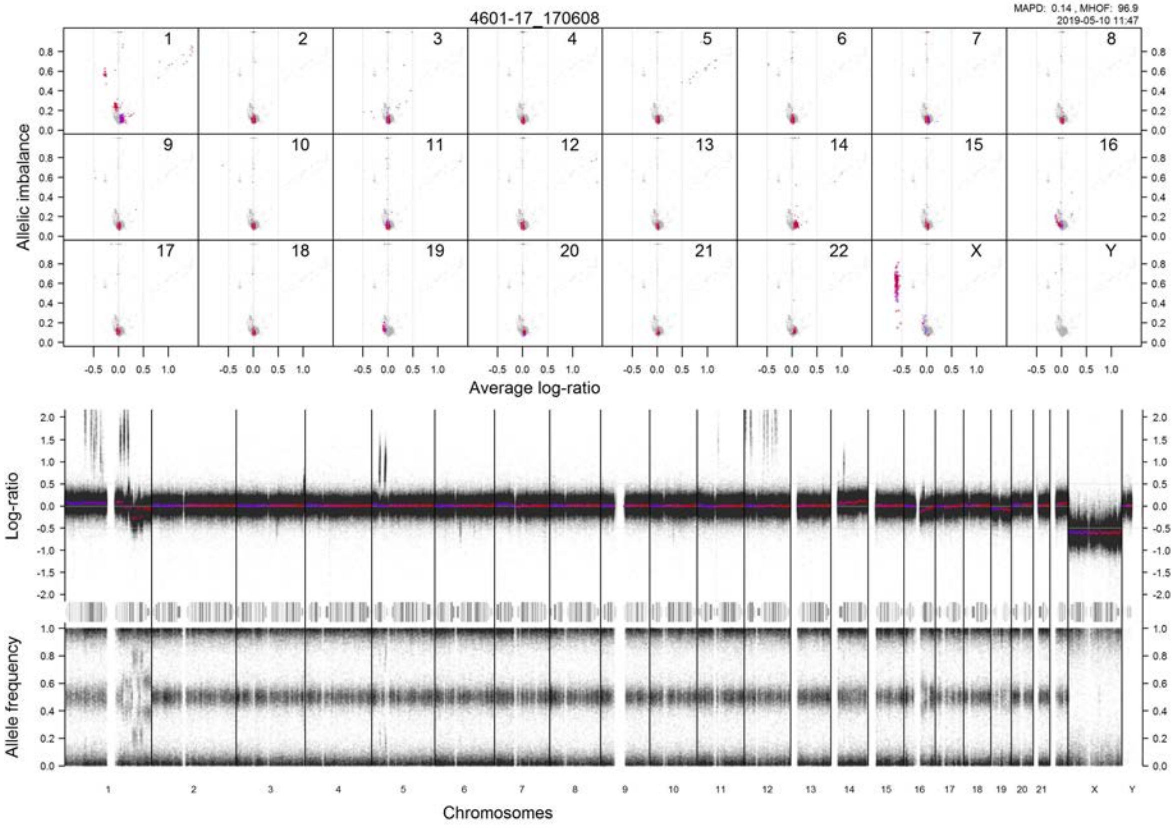

Surgical specimen

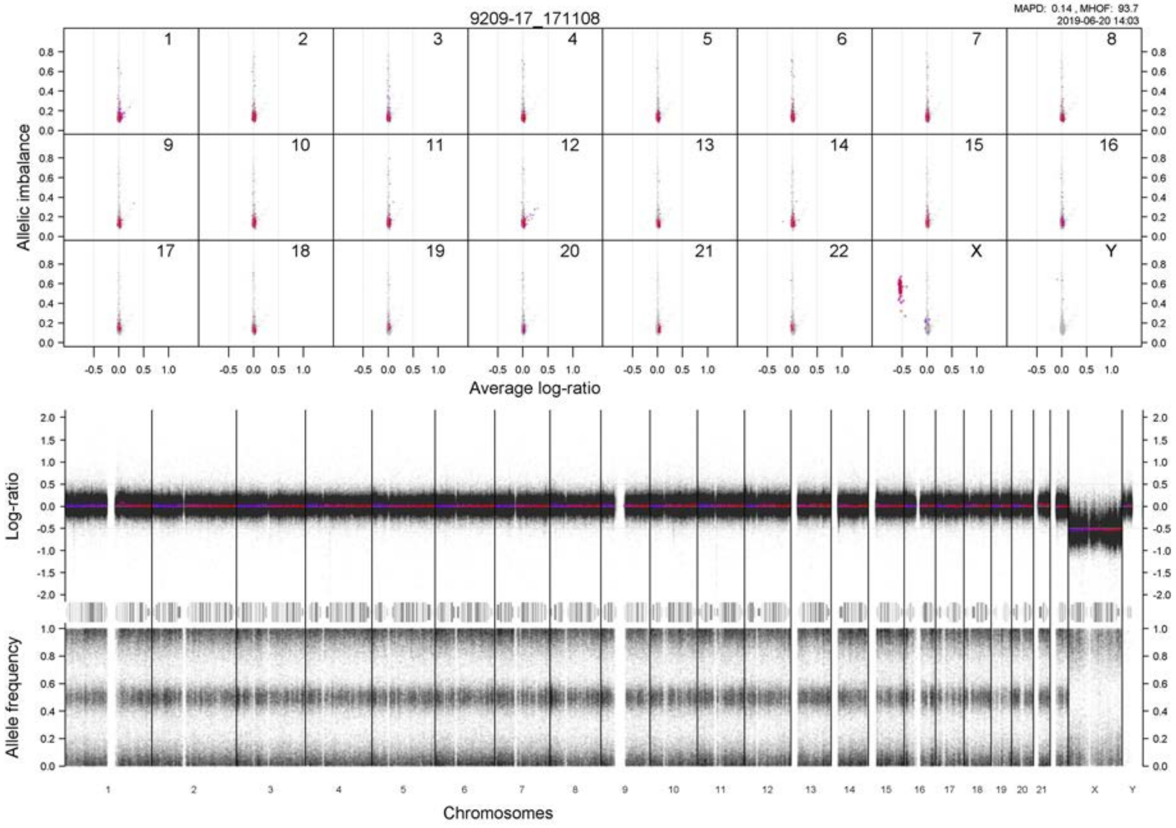

Case 66

Spindle cell sarcoma, NOS (low-grade)

CNB

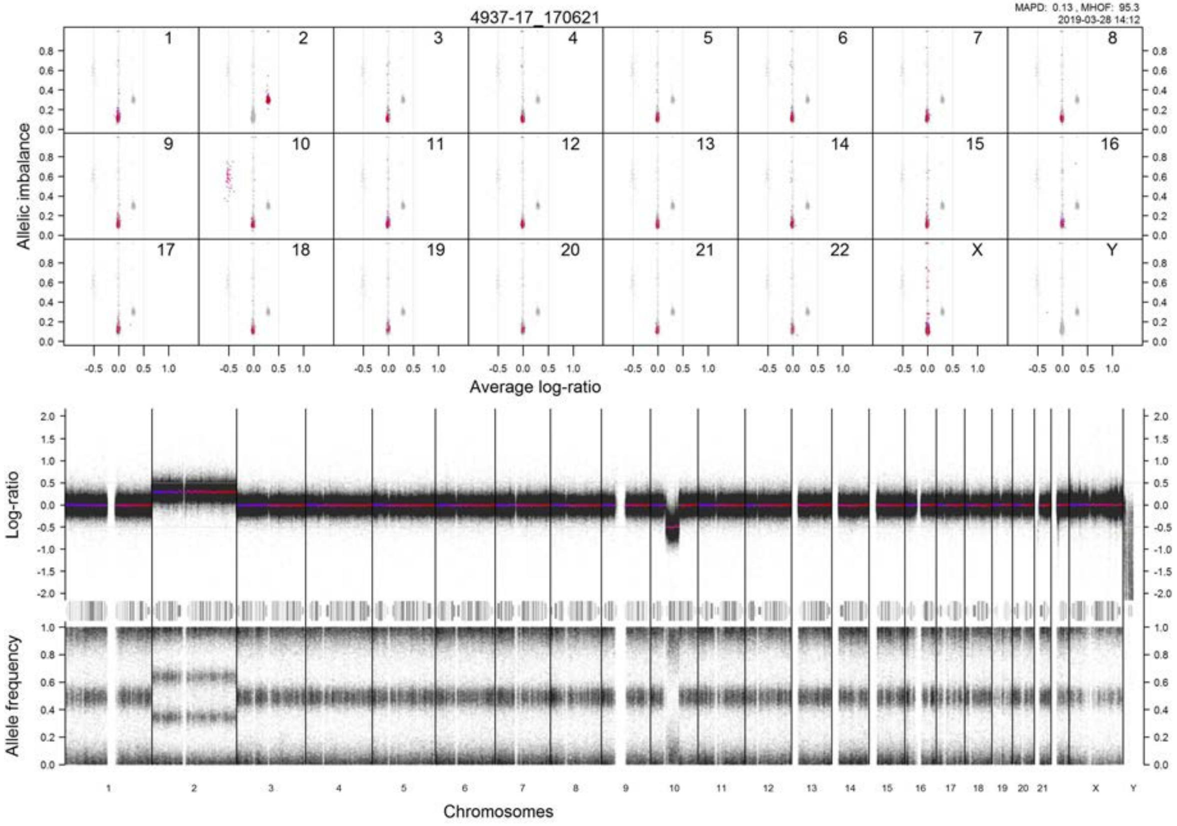

Surgical specimen

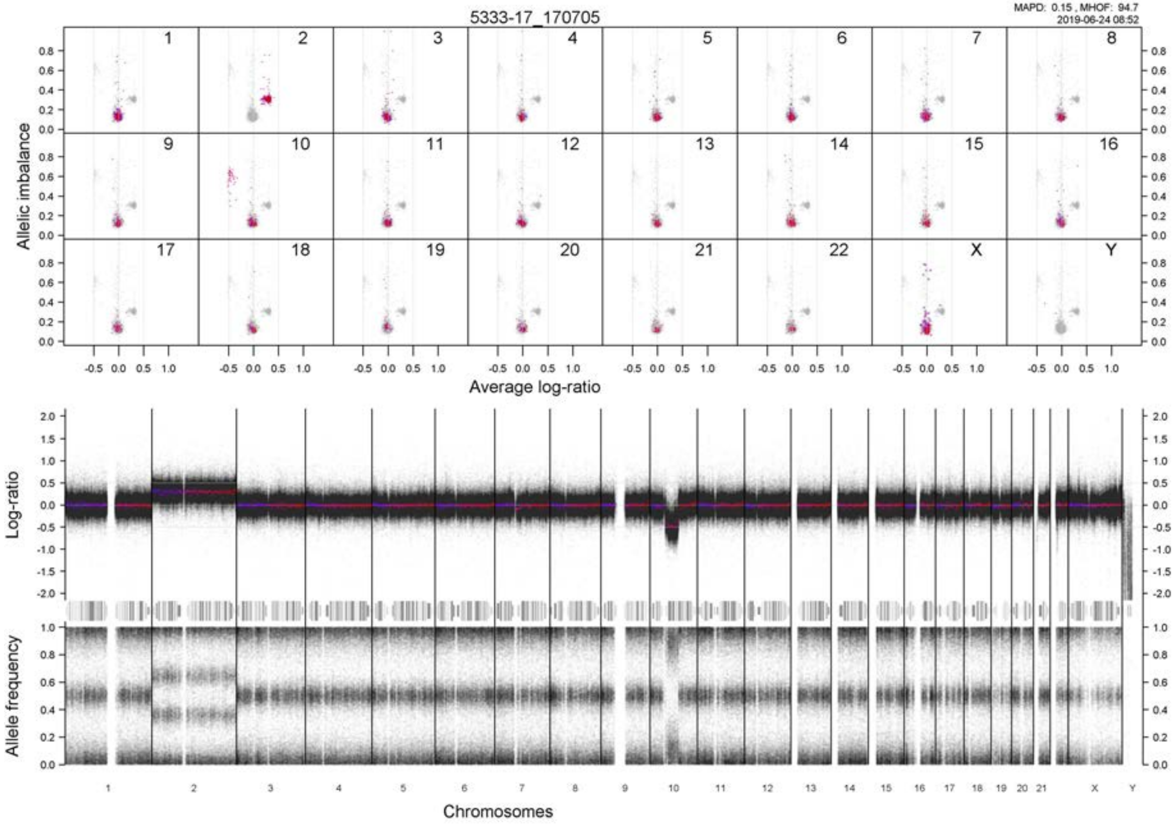

Case 68 Pleomorphic liposarcoma

CNB

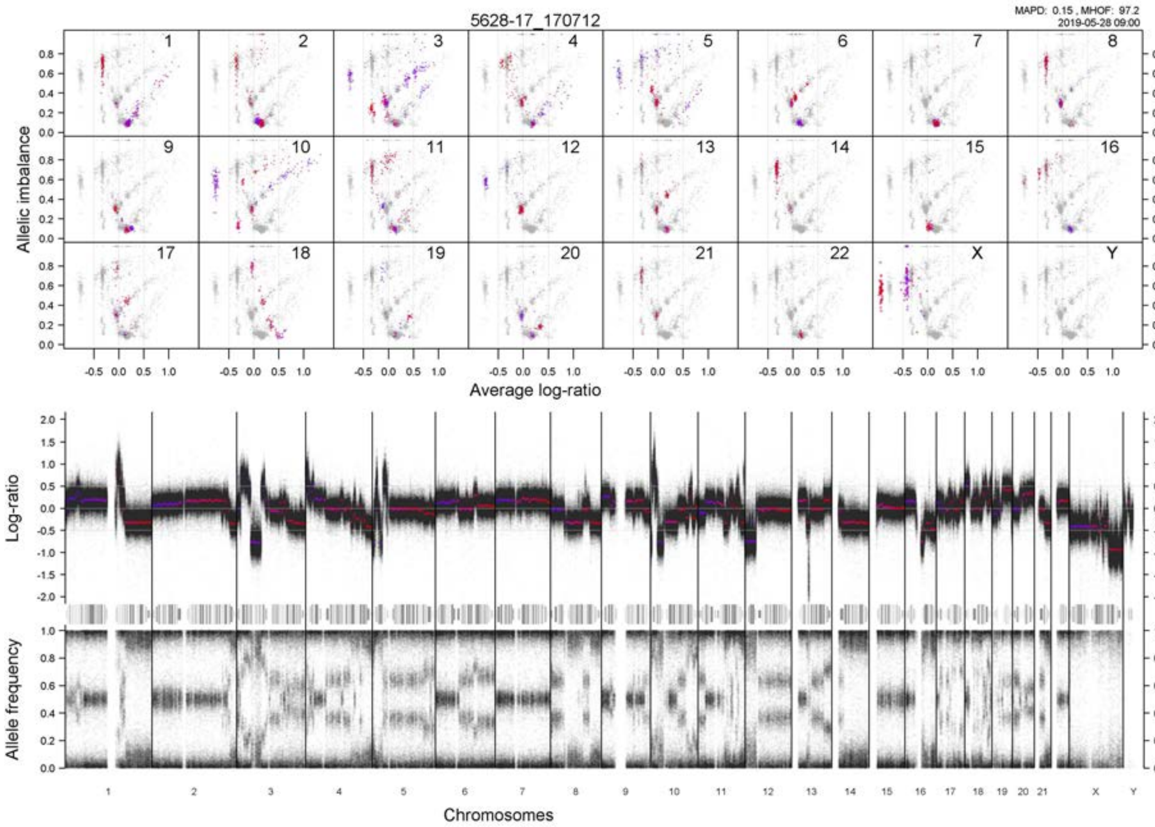

Surgical specimen

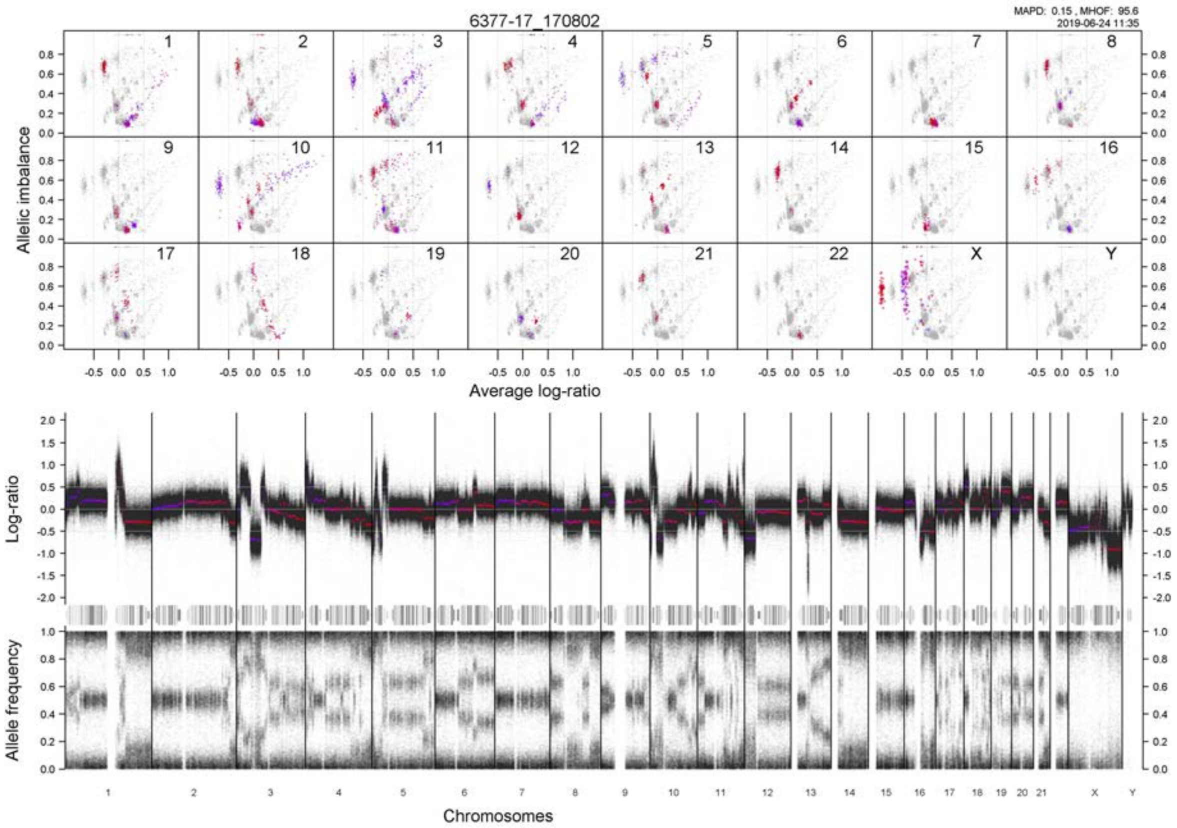

Case 70

Well-differentiated liposarcoma

CNB

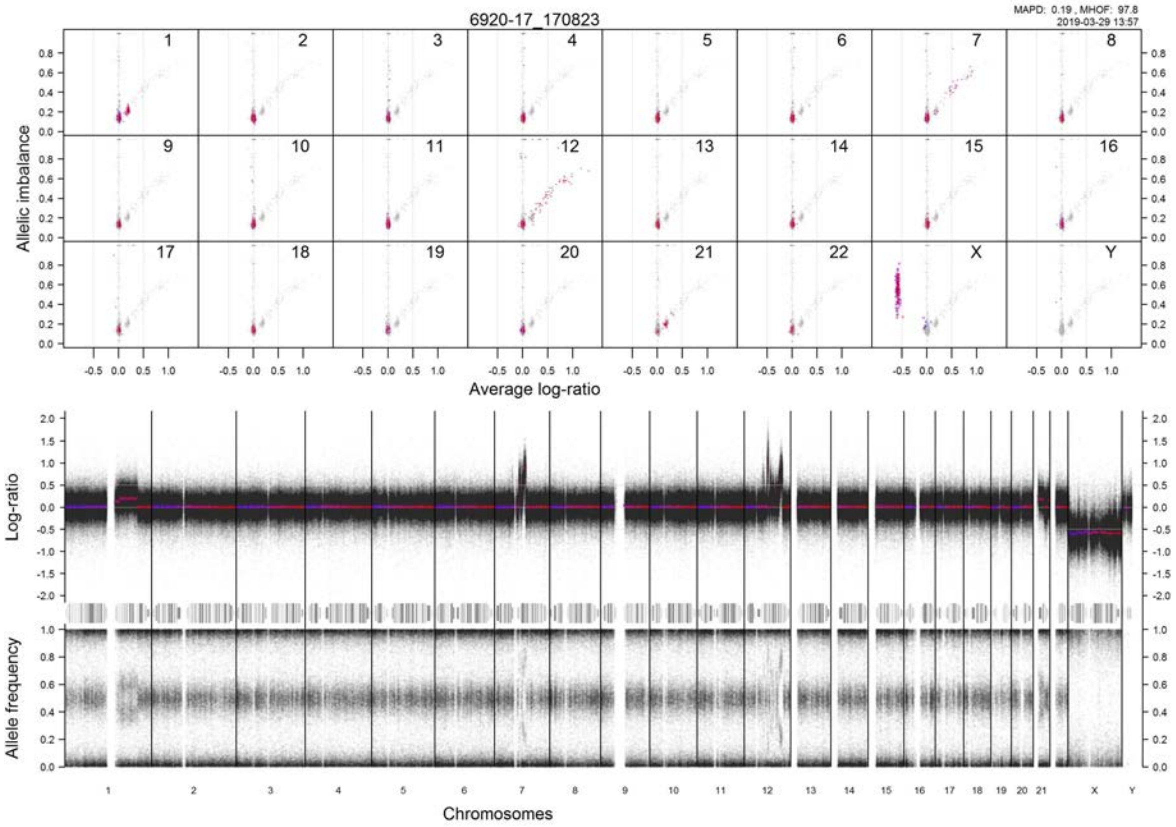

Surgical specimen

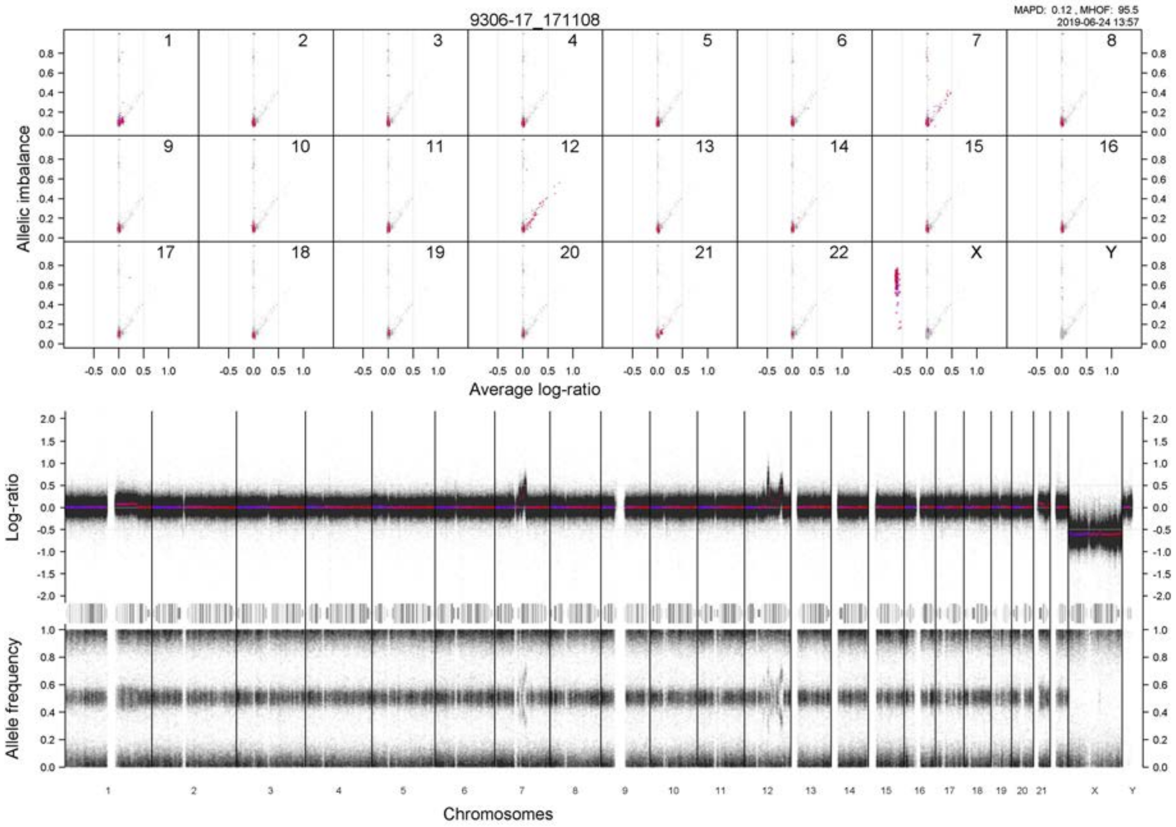

Case 72 (CNB)

Low-grade fibromyxoid sarcoma

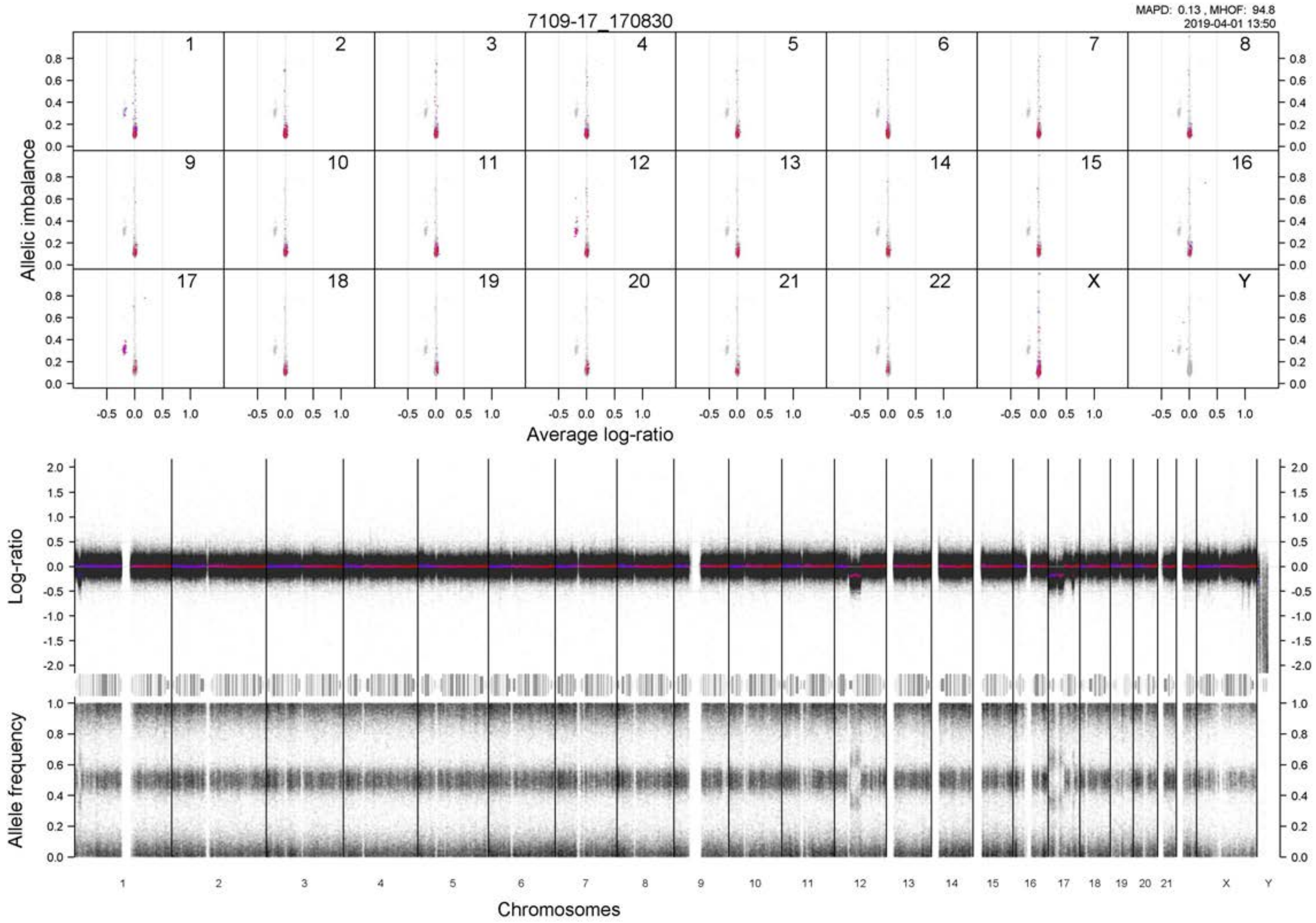

Case 74 (CNB) Myxofibrosarcoma

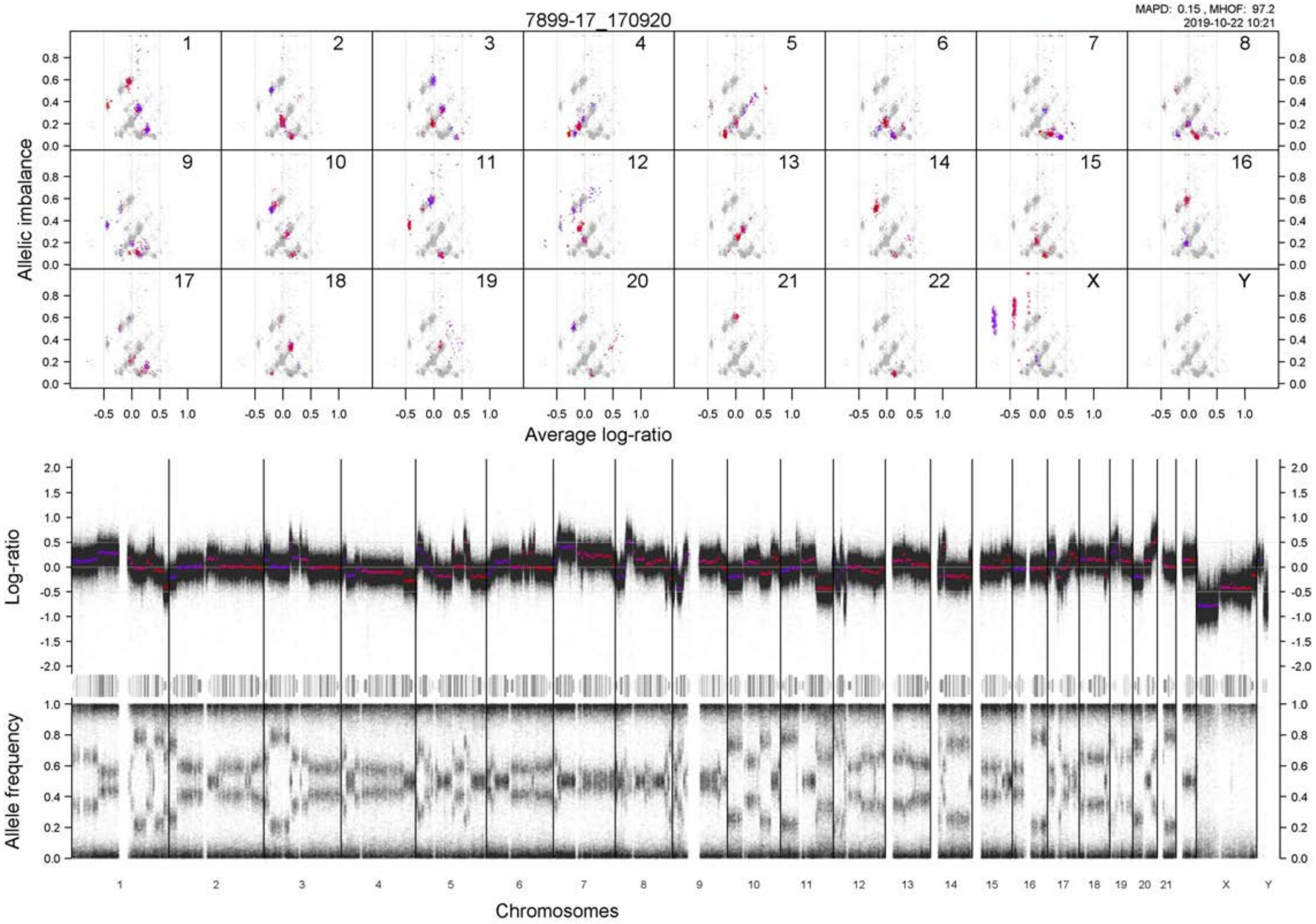

Case 75 (CNB)

Desmoid fibromatosis

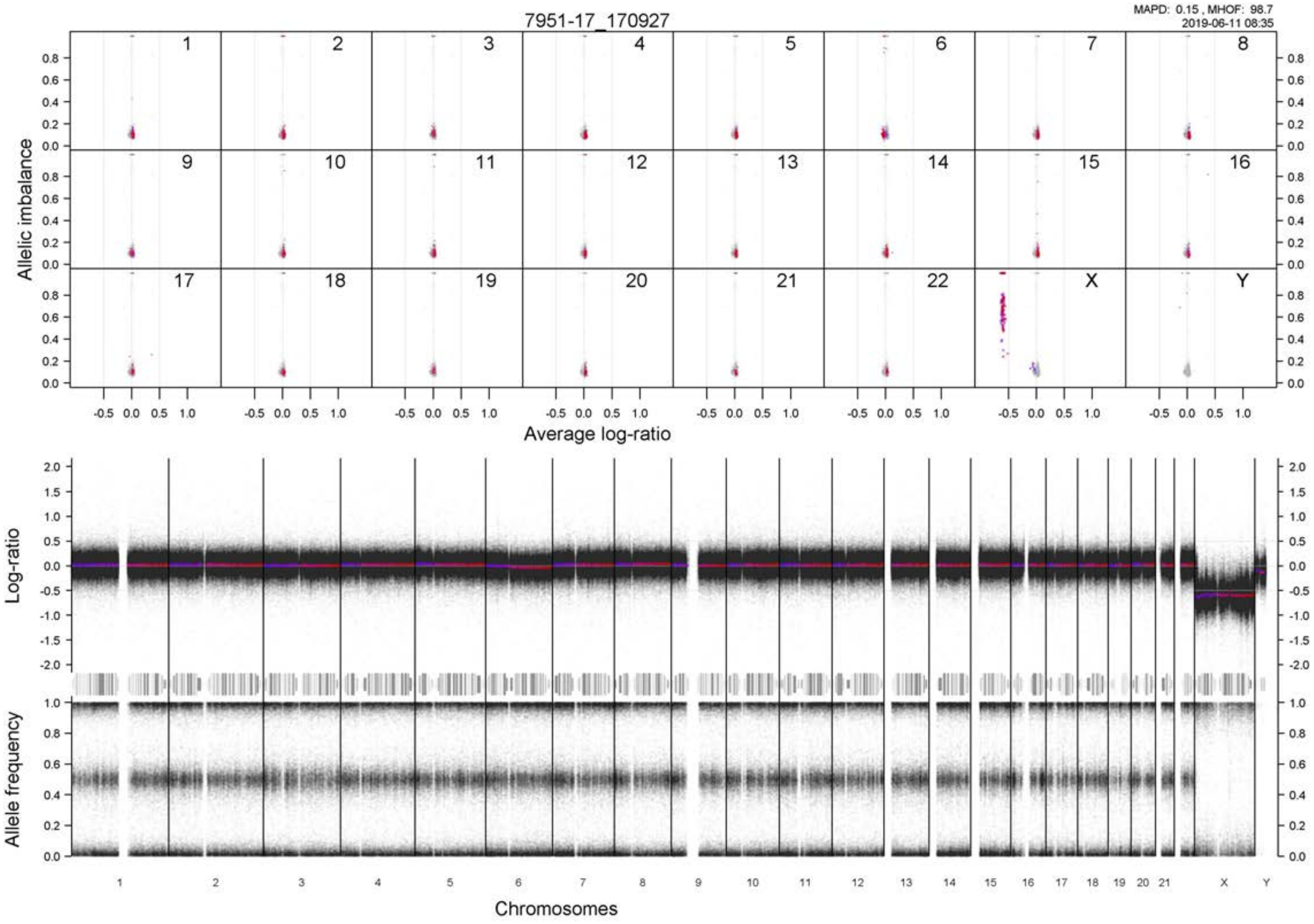

Case 76 (CNB) UPS

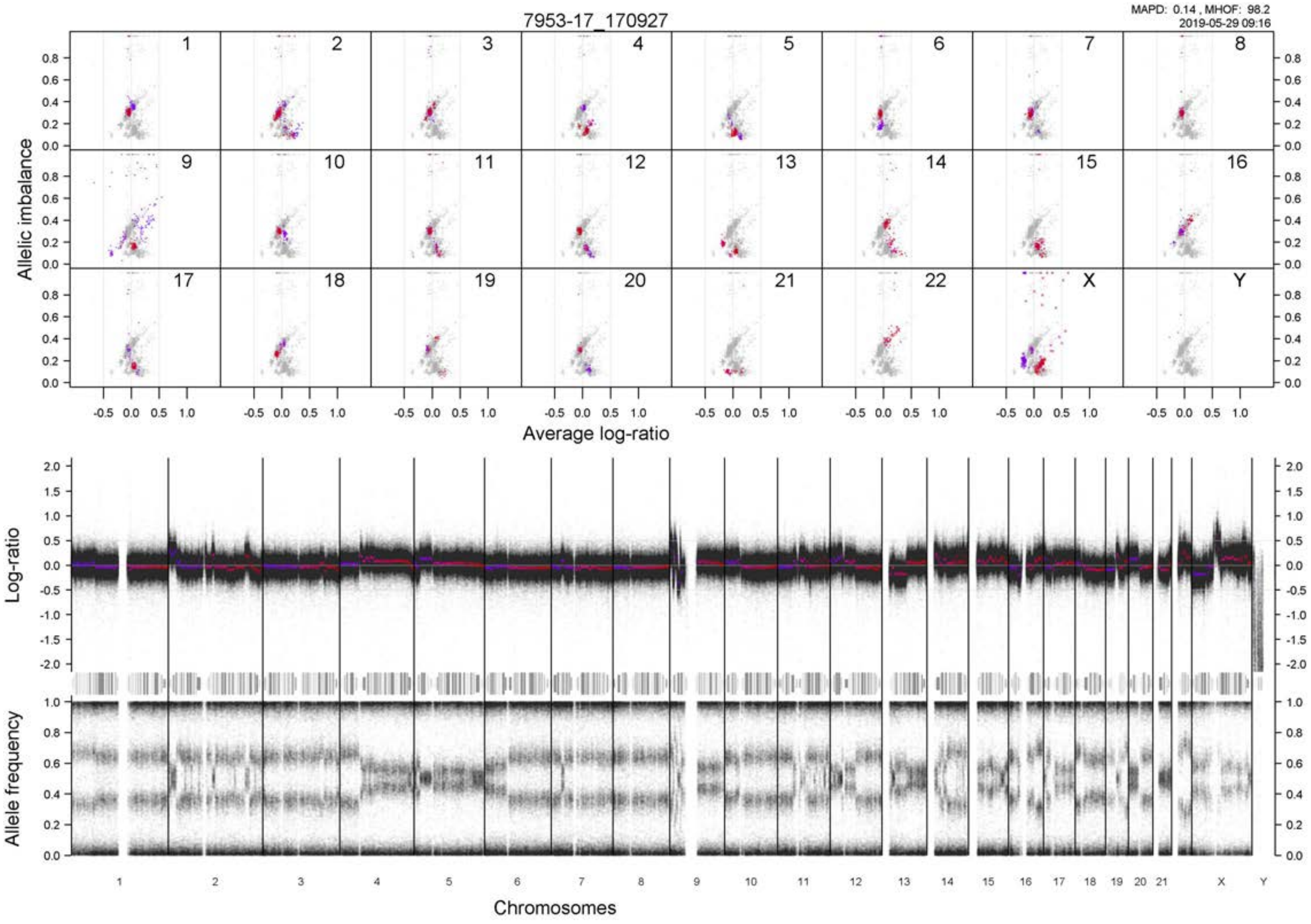

Case 77

Dedifferentiated liposarcoma

CNB

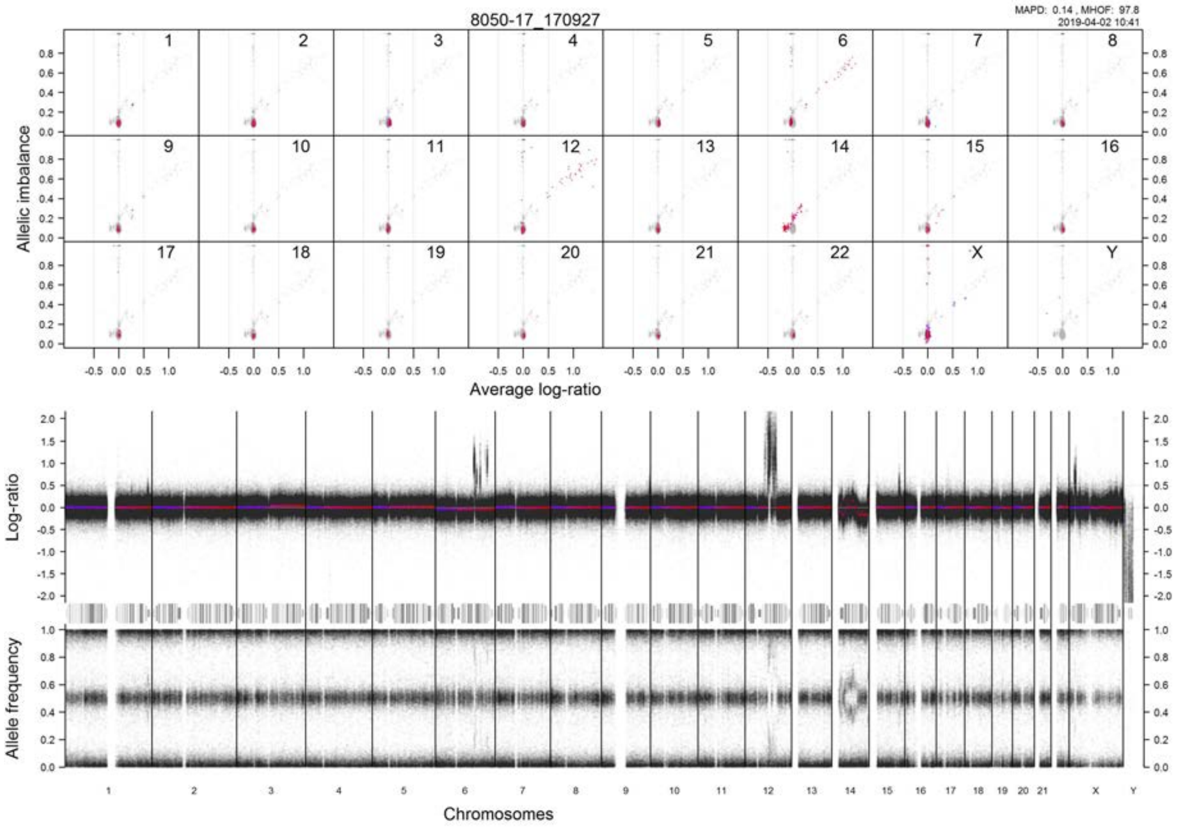

Surgical specimen

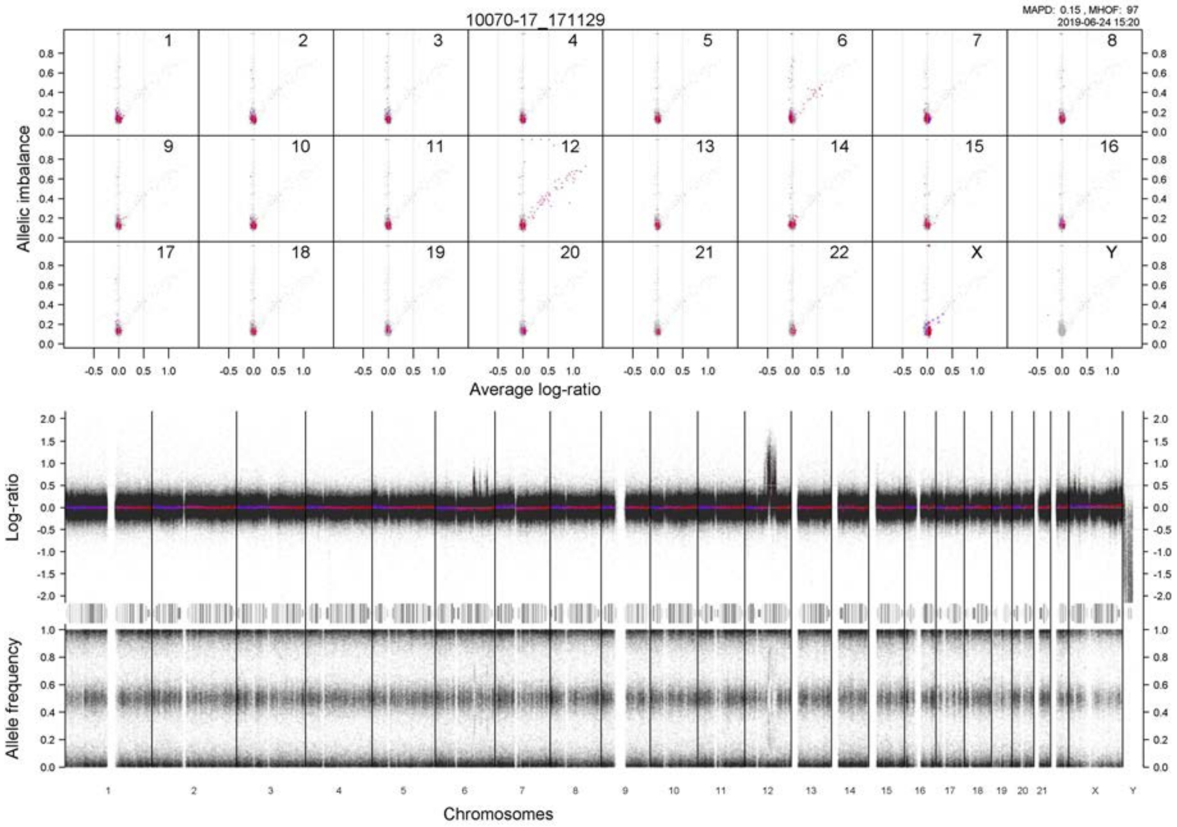

Case 81

Schwannoma

CNB

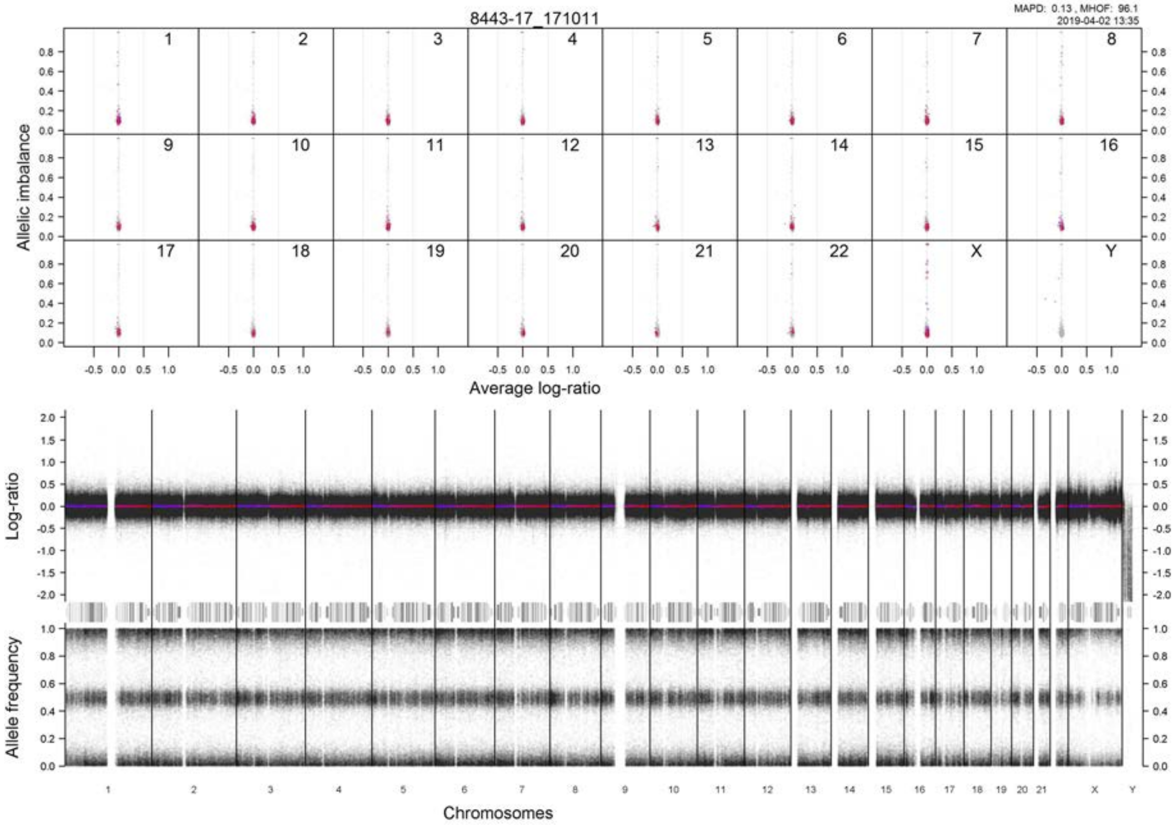

Surgical specimen

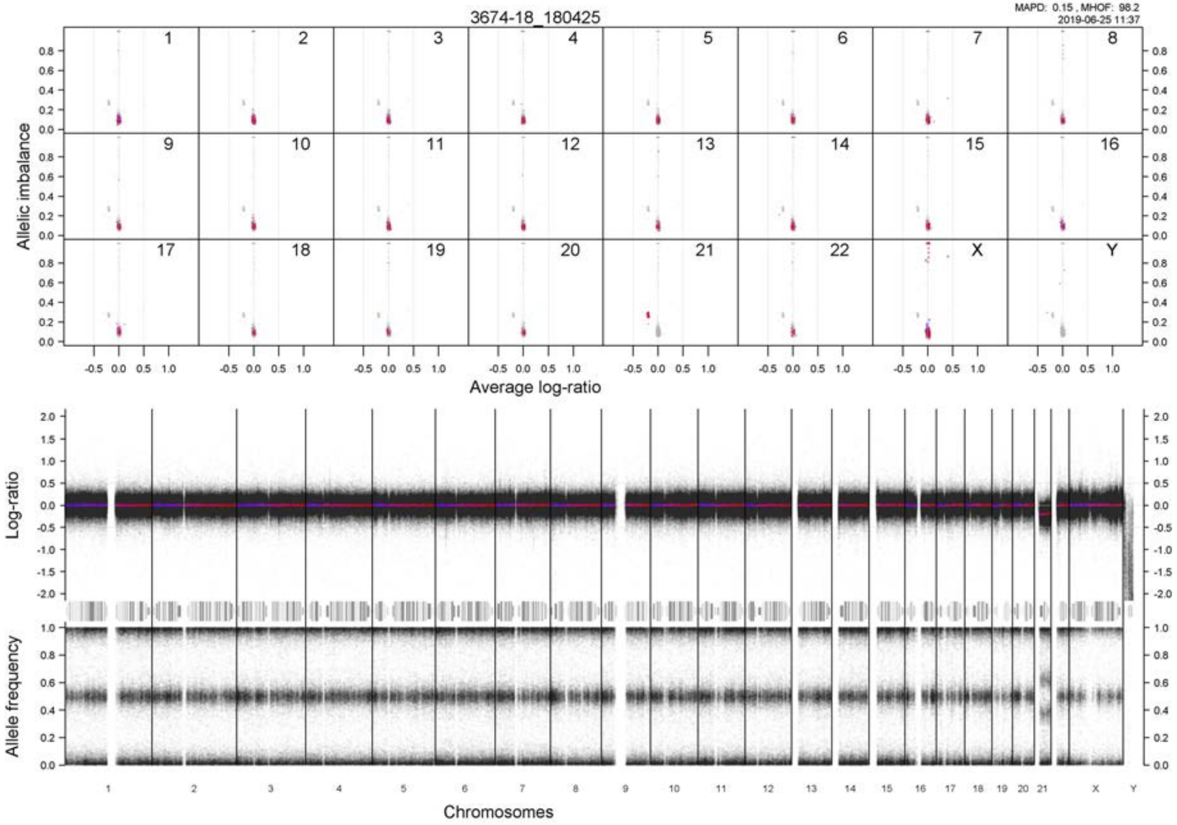

Case 82 (CNB) Osteosarcoma

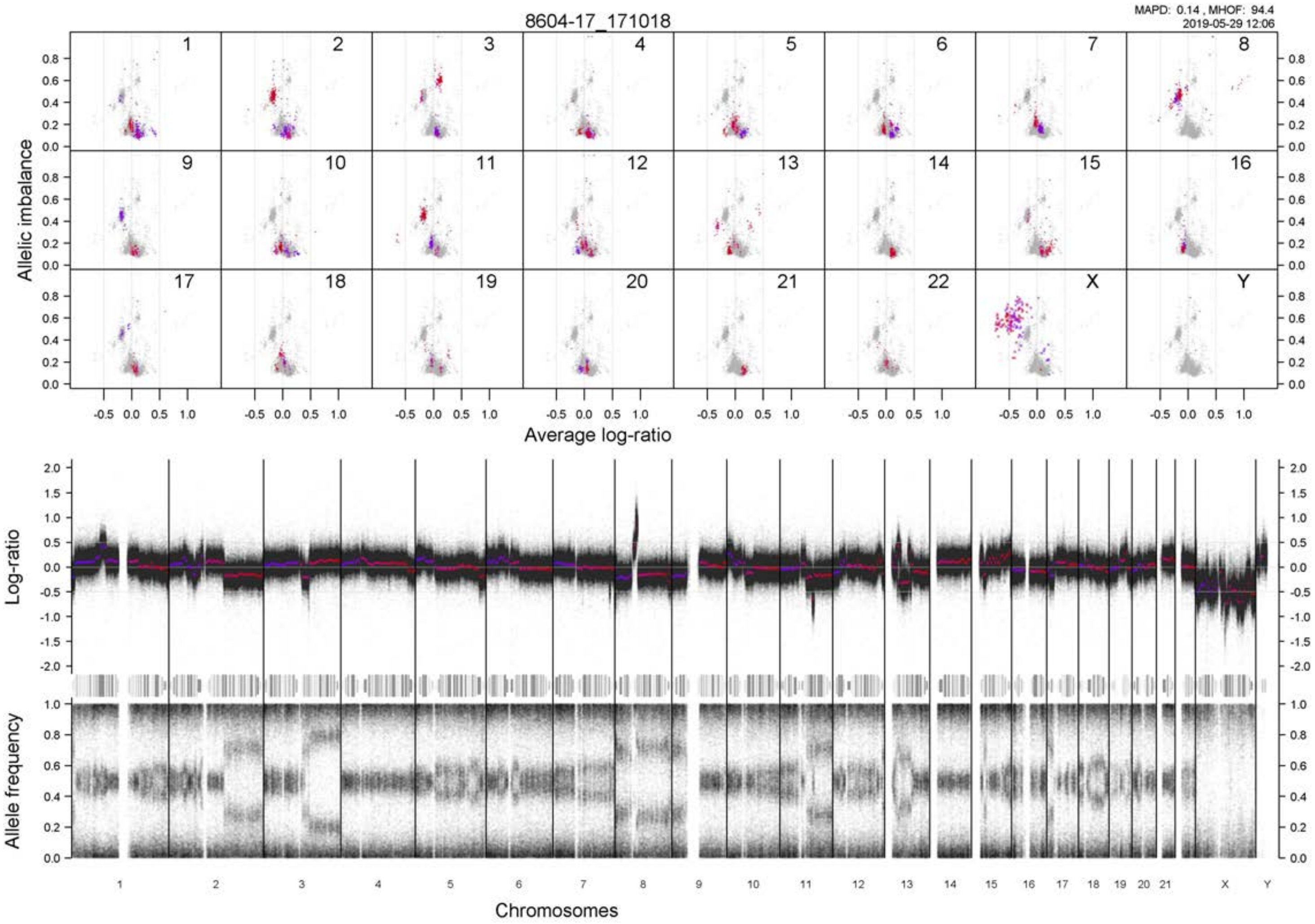

Case 84 (CNB) Dedifferentiated liposarcoma

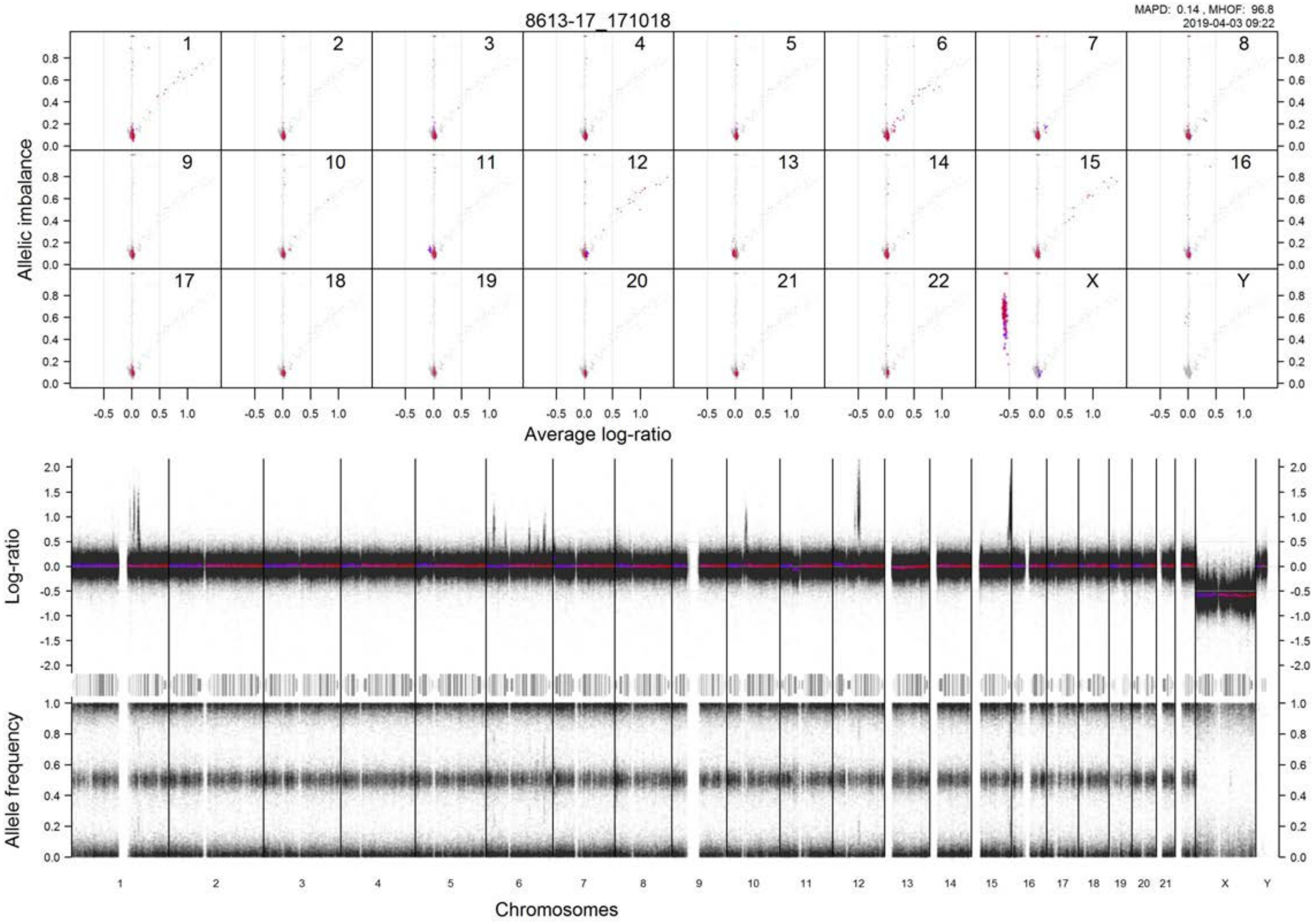

Case 85

GIST

CNB

Surgical specimen

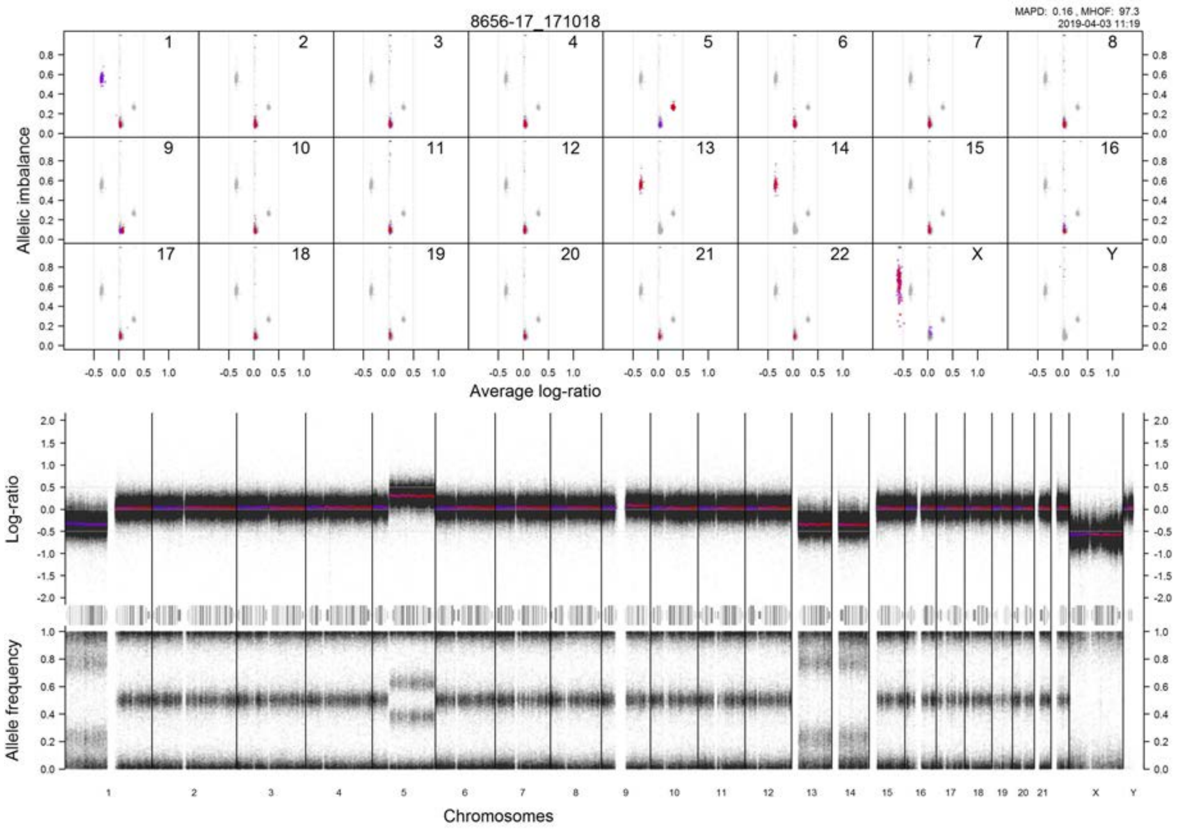

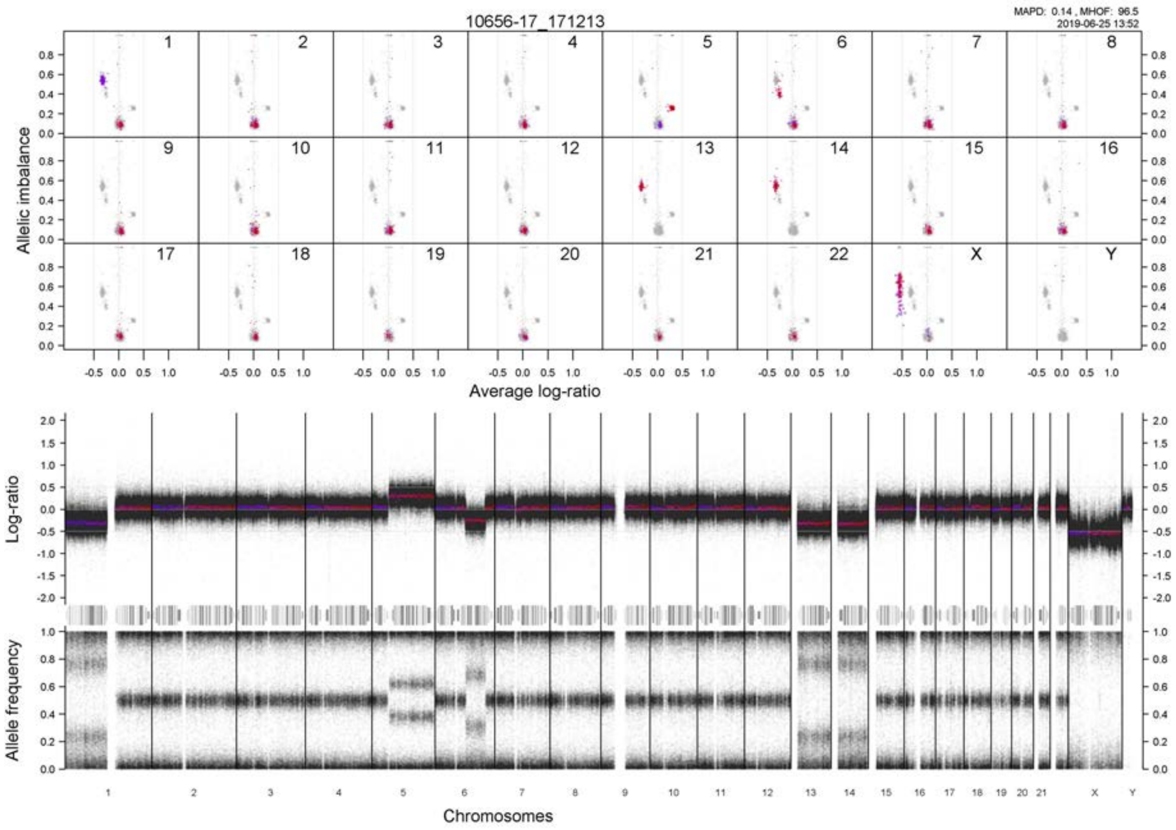

Case 86

Dedifferentiated liposarcoma

CNB

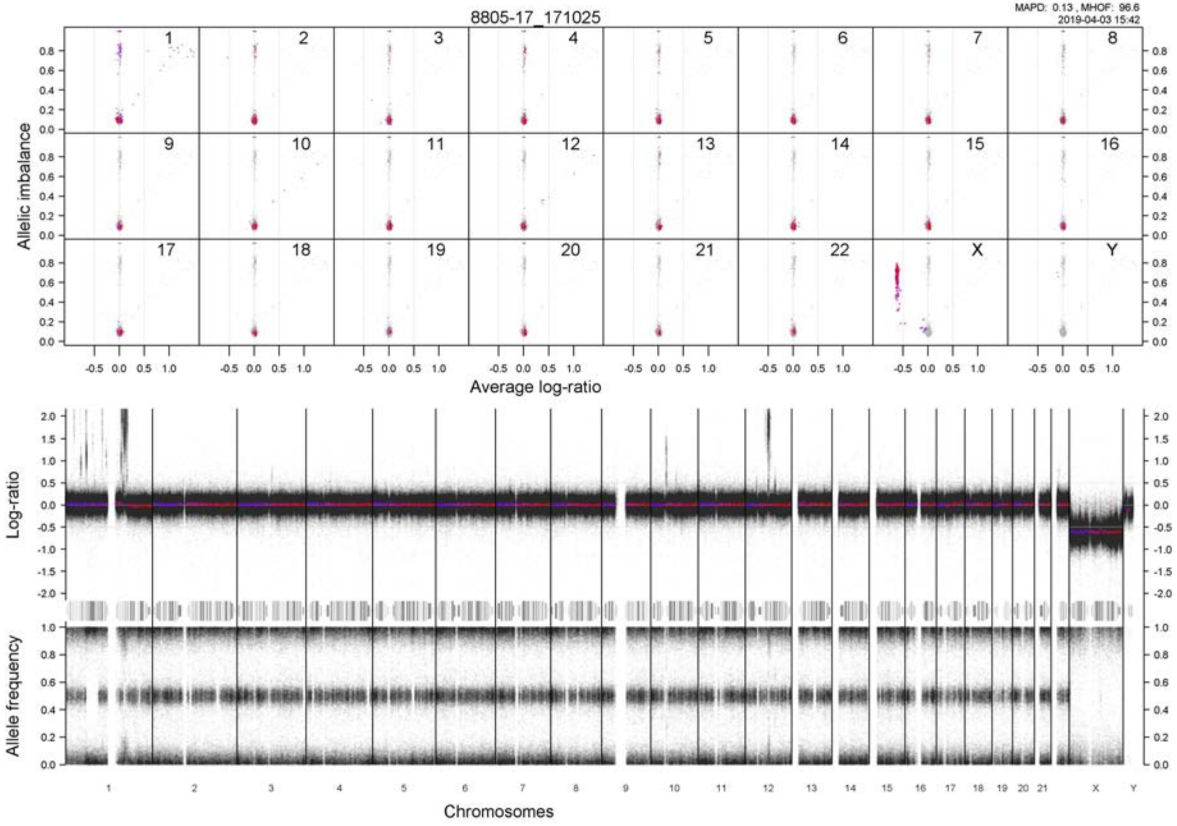

Surgical specimen

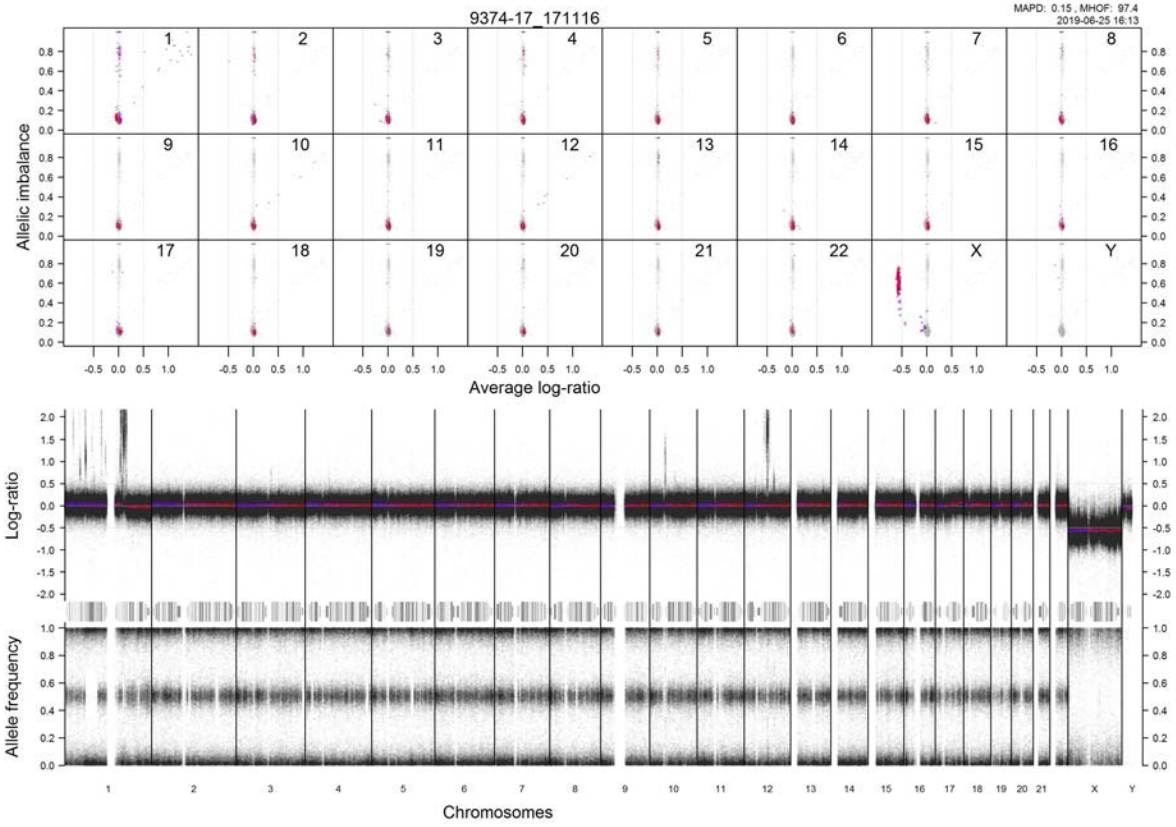

Case 87

UPS

CNB

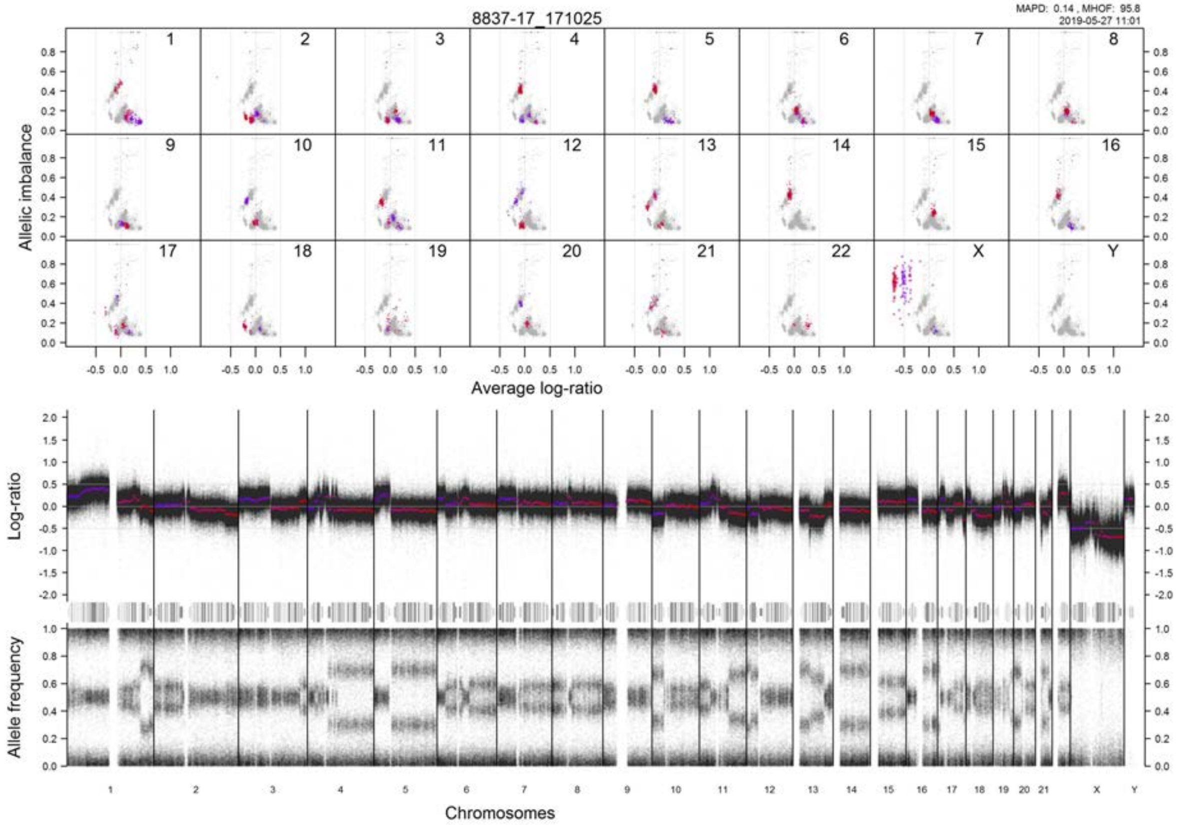

Surgical specimen

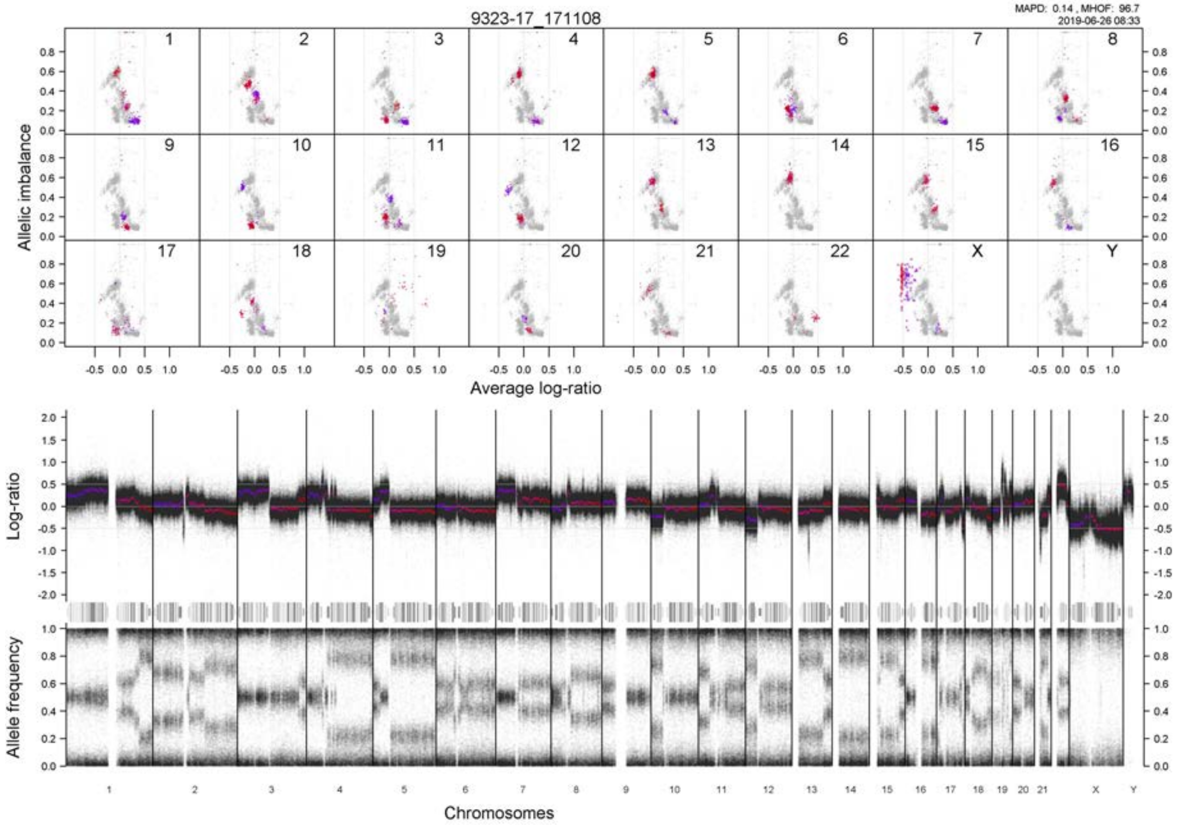

Case 88 (CNB)

Leiomyosarcoma

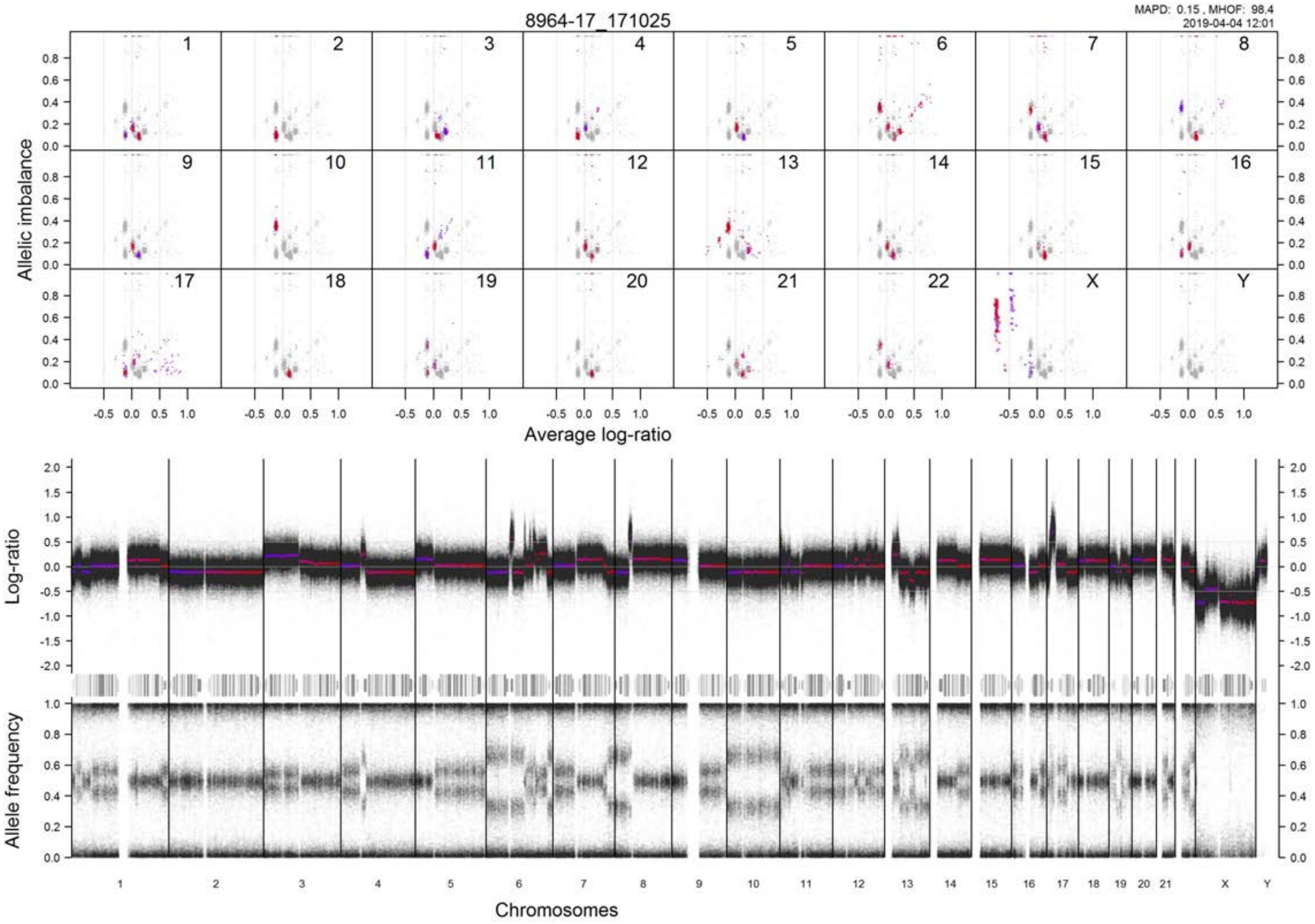

Case 89

Low-grade fibromyxoid sarcoma

CNB

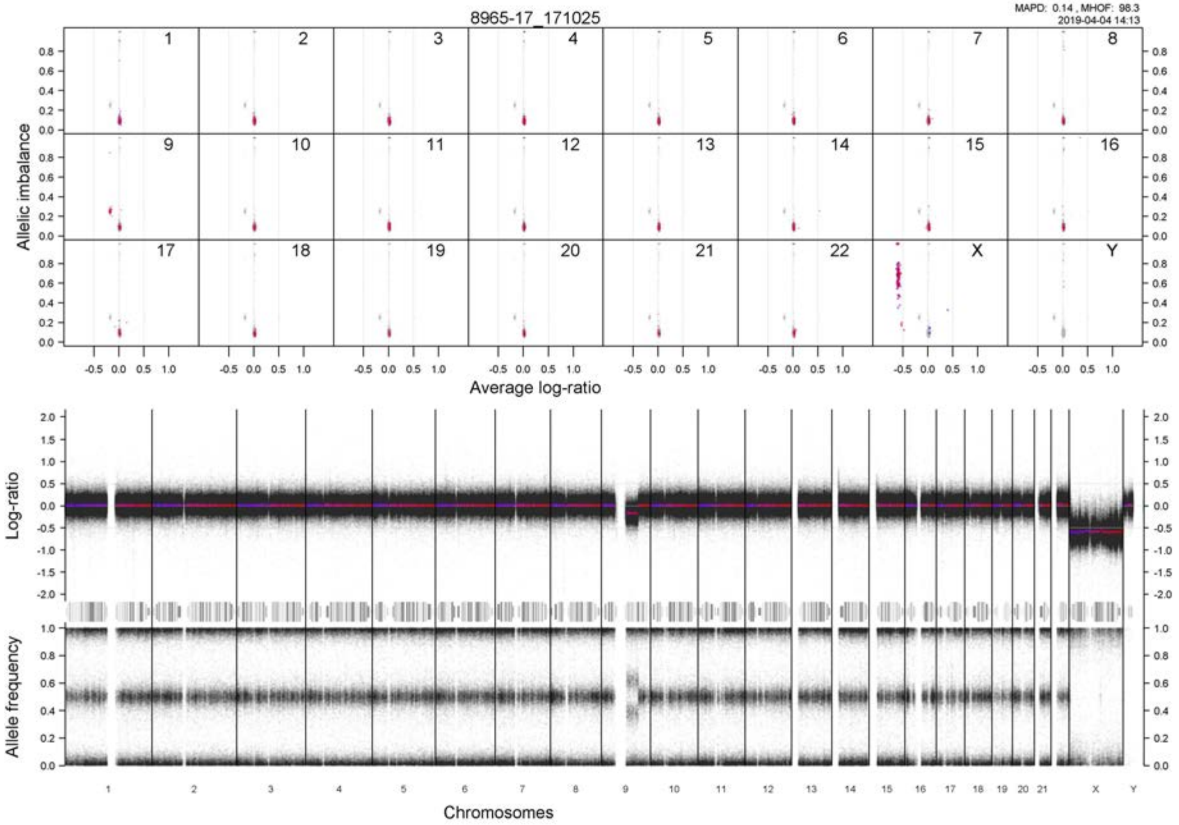

Surgical specimen

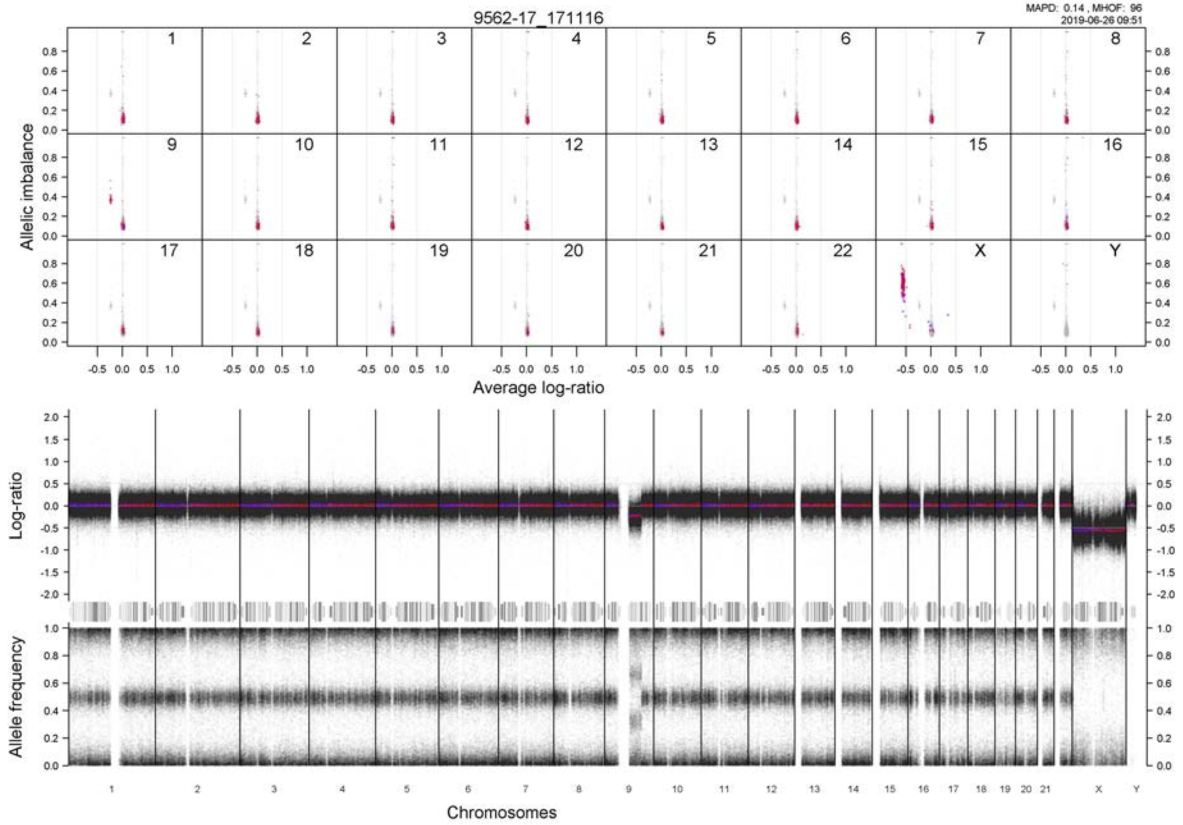

Case 90 (CNB)

Cellular angiofibroma

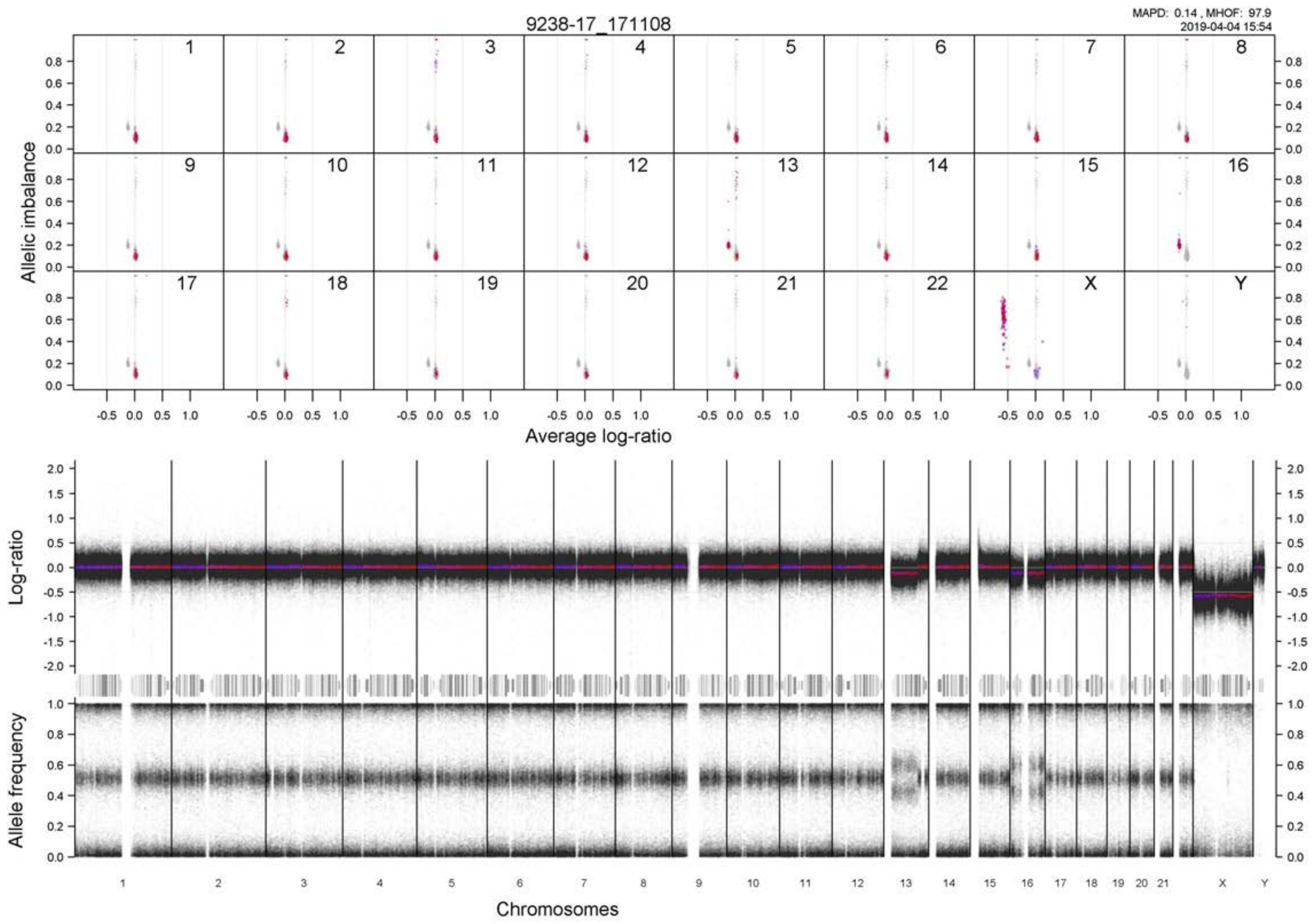

Case 91 UPS

CNB

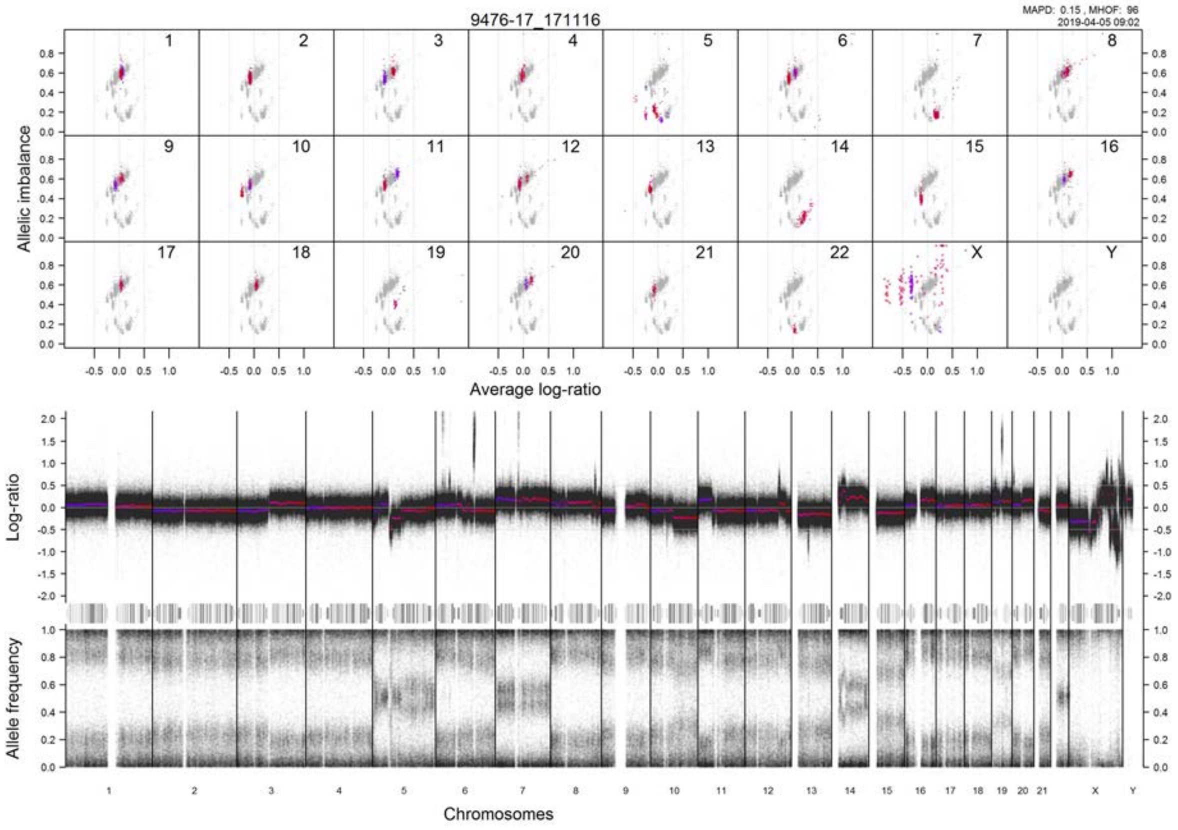

Surgical specimen

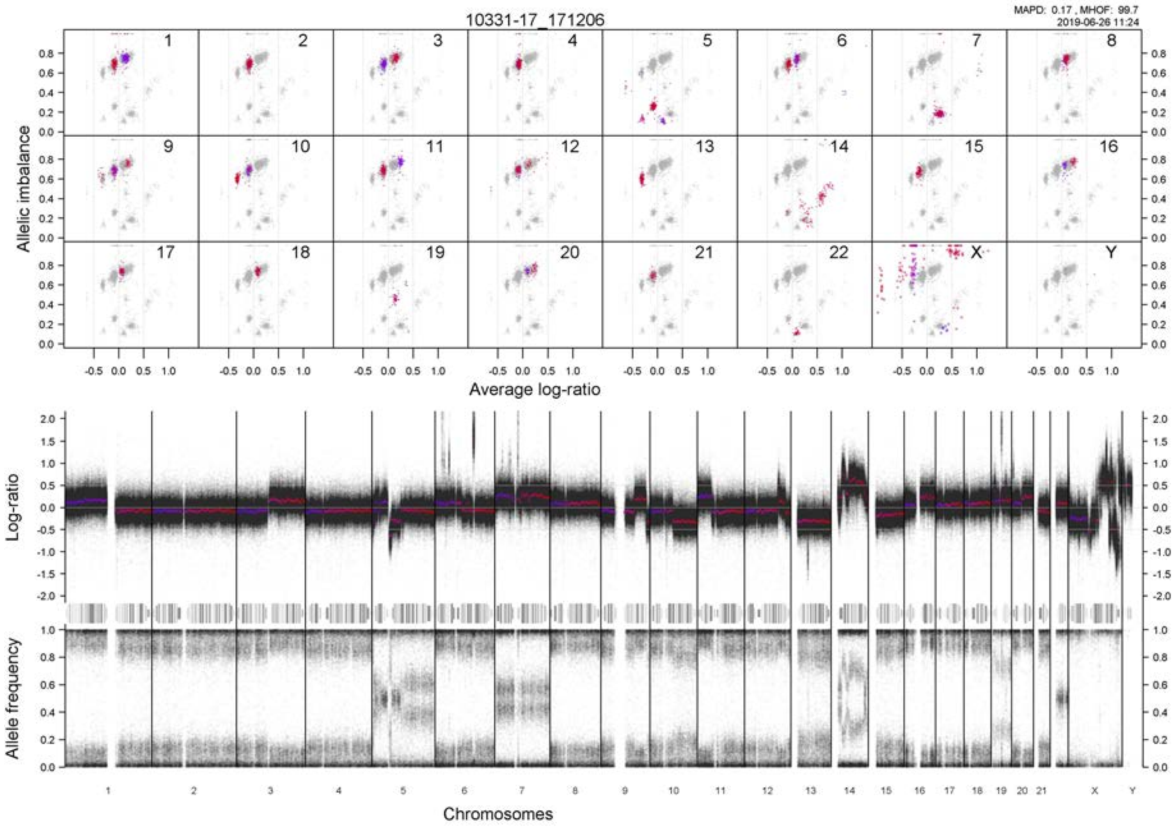

Case 92 MPNST

CNB

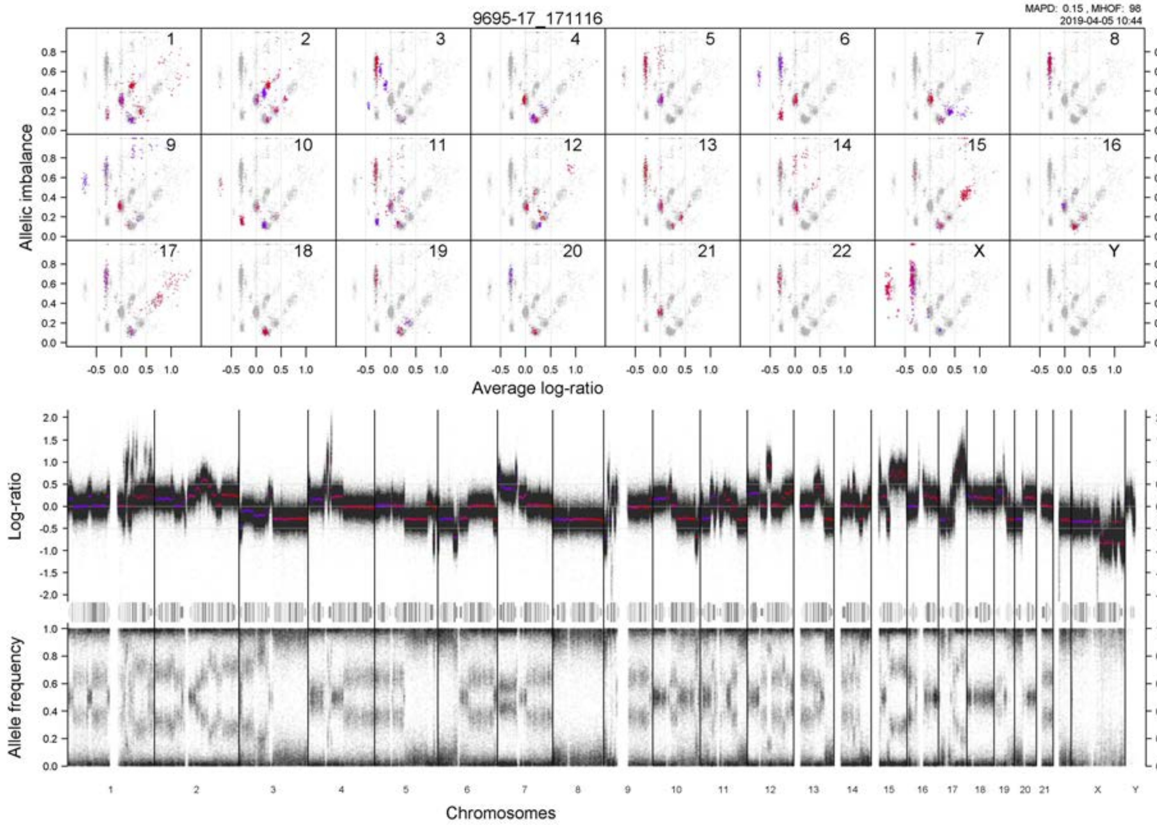

Surgical specimen

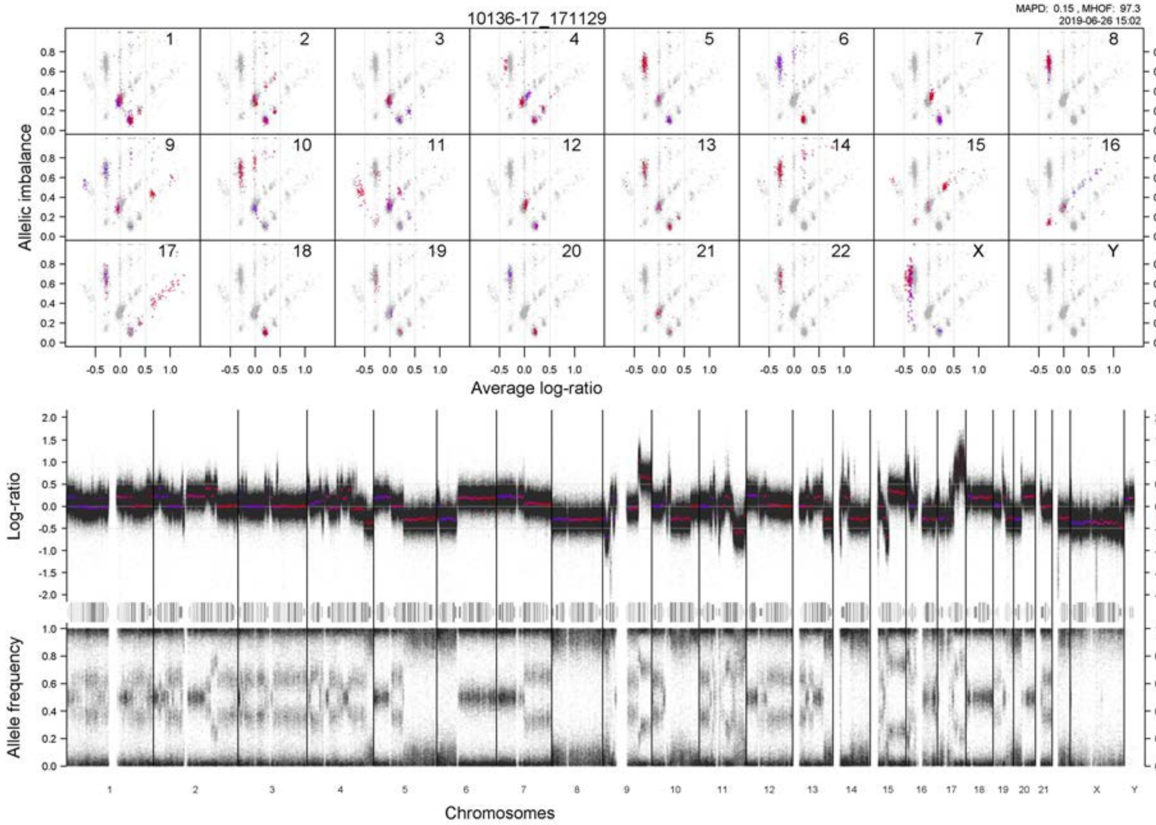

Case 93 (Surgical specimen) Spindle cell sarcoma, NOS (recurrent)

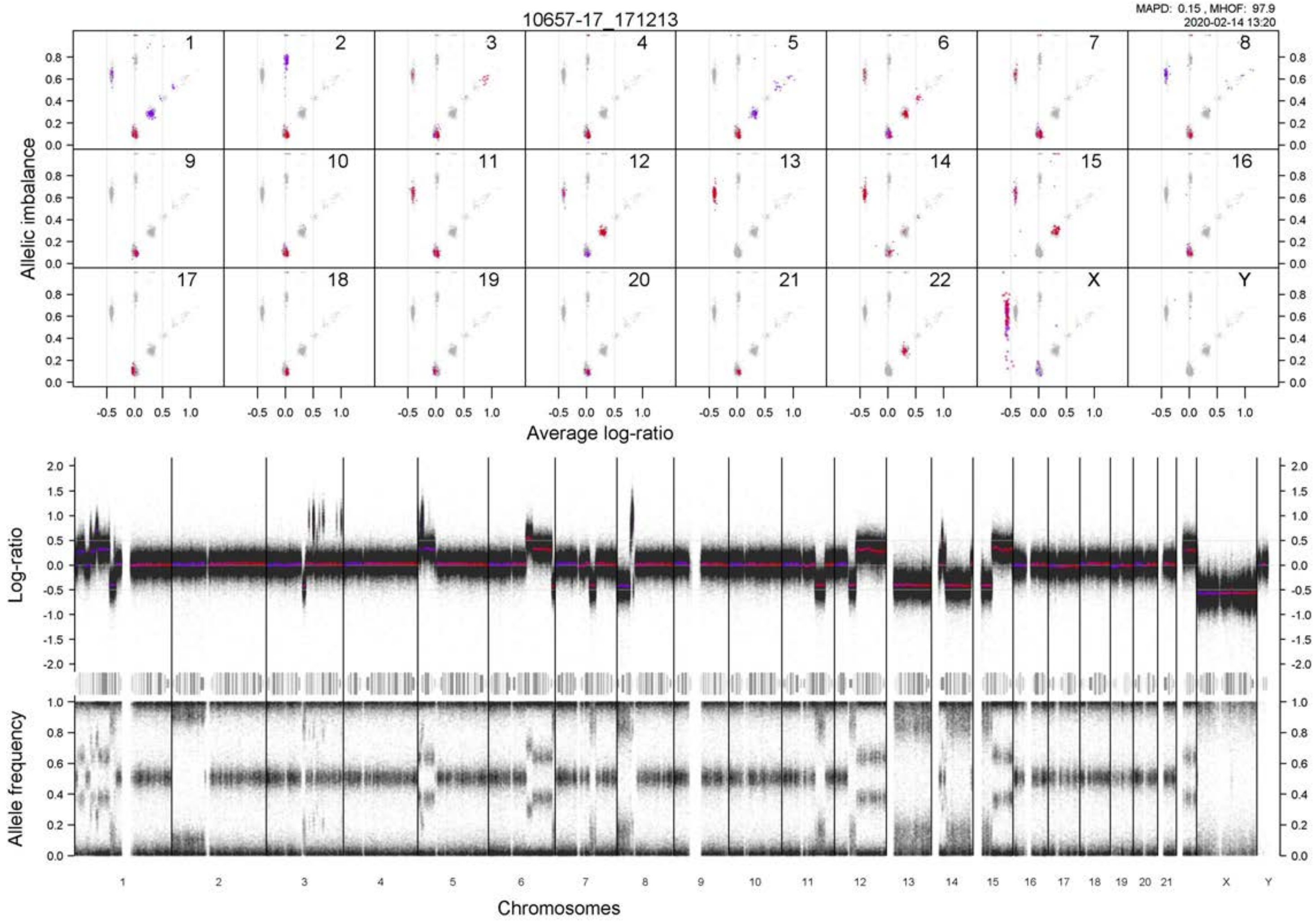

Case 96

Dedifferentiated liposarcoma

CNB

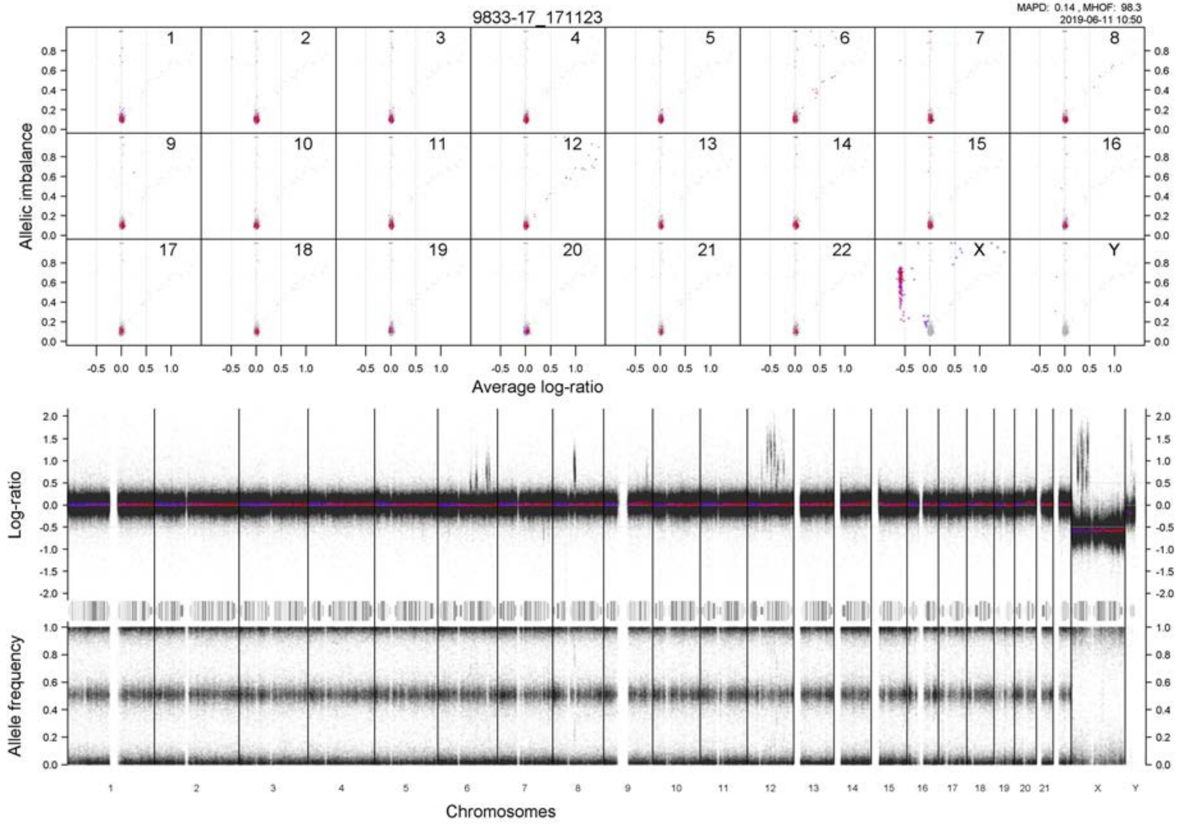

Surgical specimen

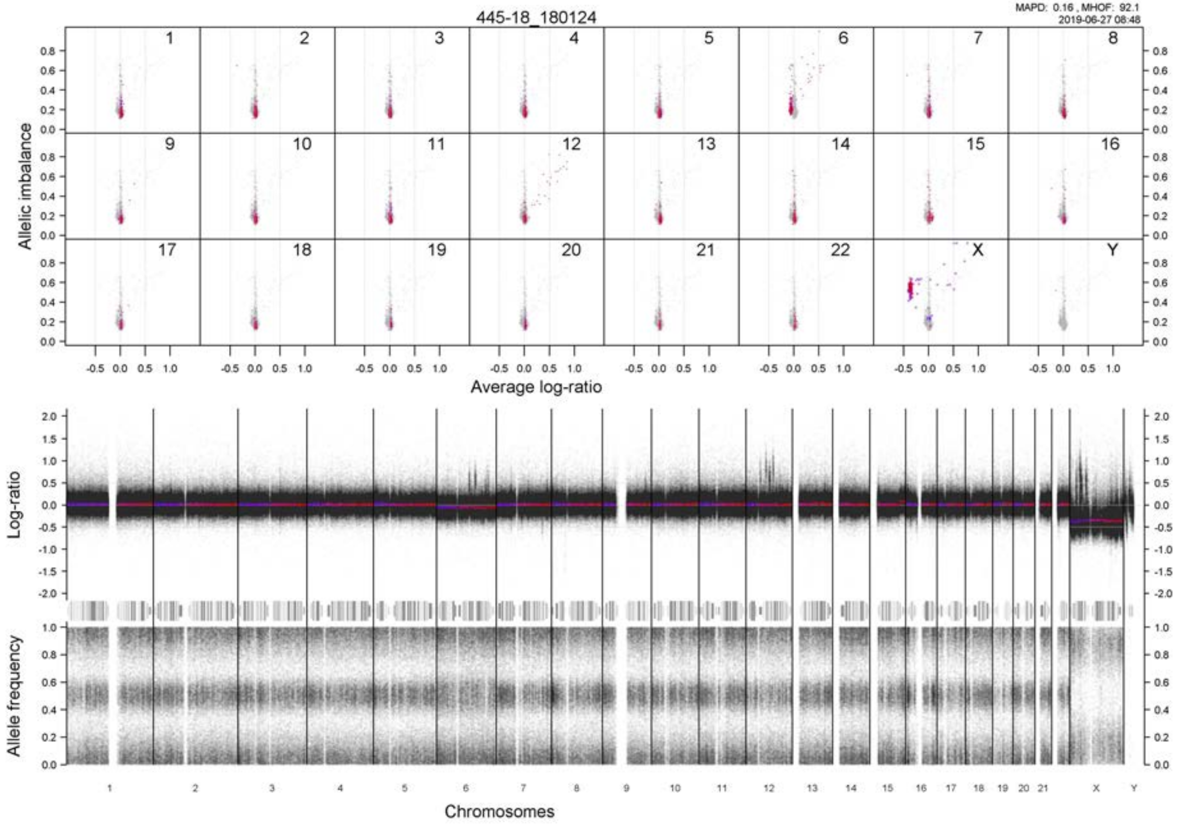

Case 99 (Surgical specimen) Neurofibroma

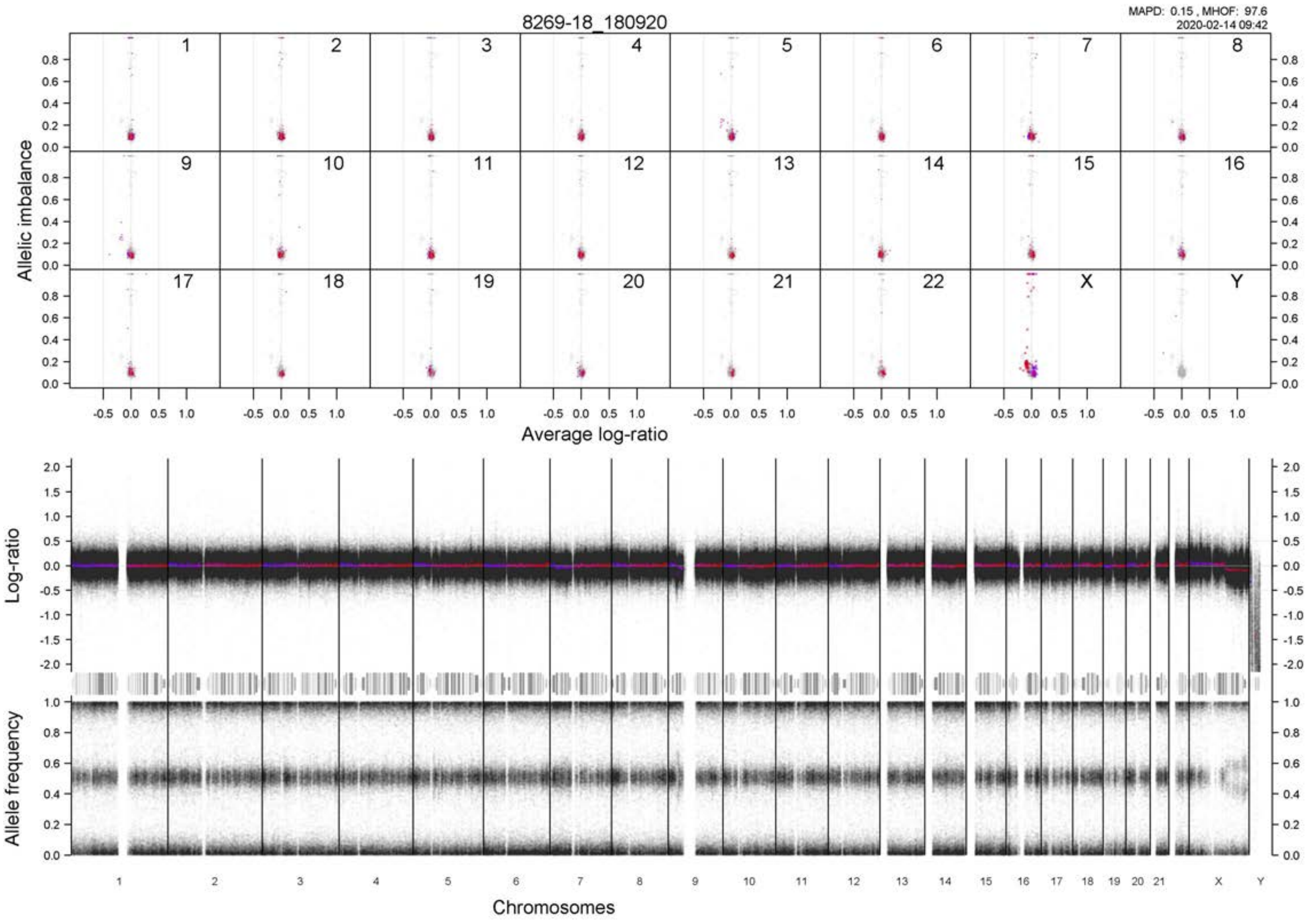

Case 101

Myxofibrosarcoma

CNB

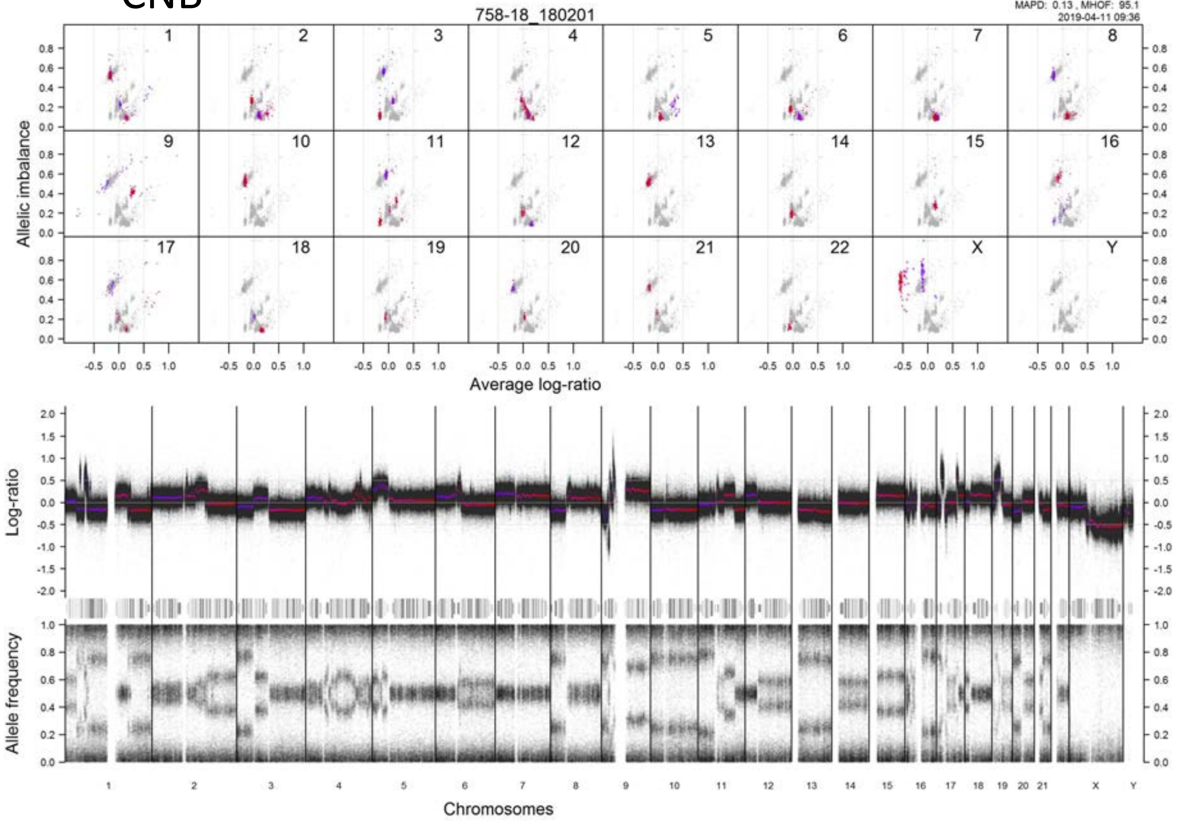

Surgical specimen

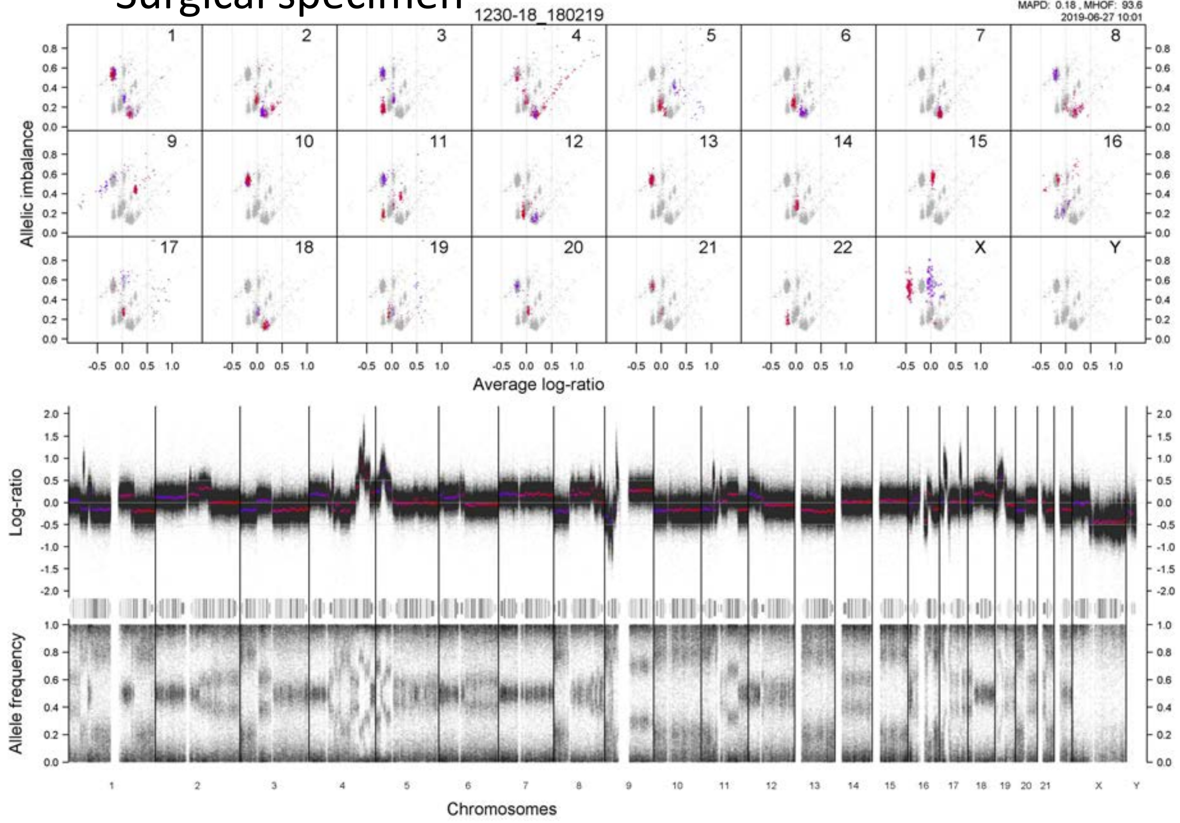

Case 102 (CNB)

GIST

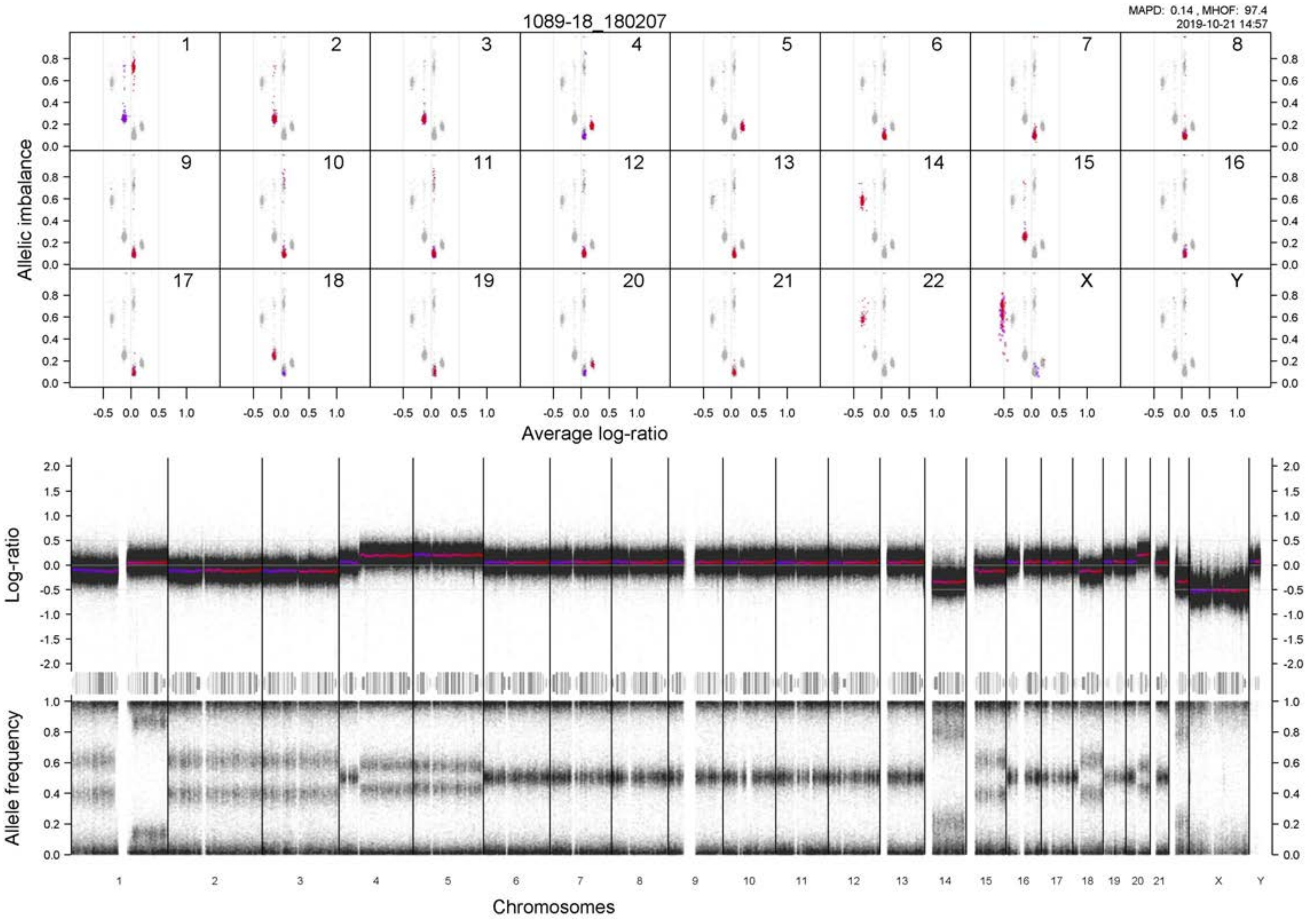

Case 104 UPS

CNB

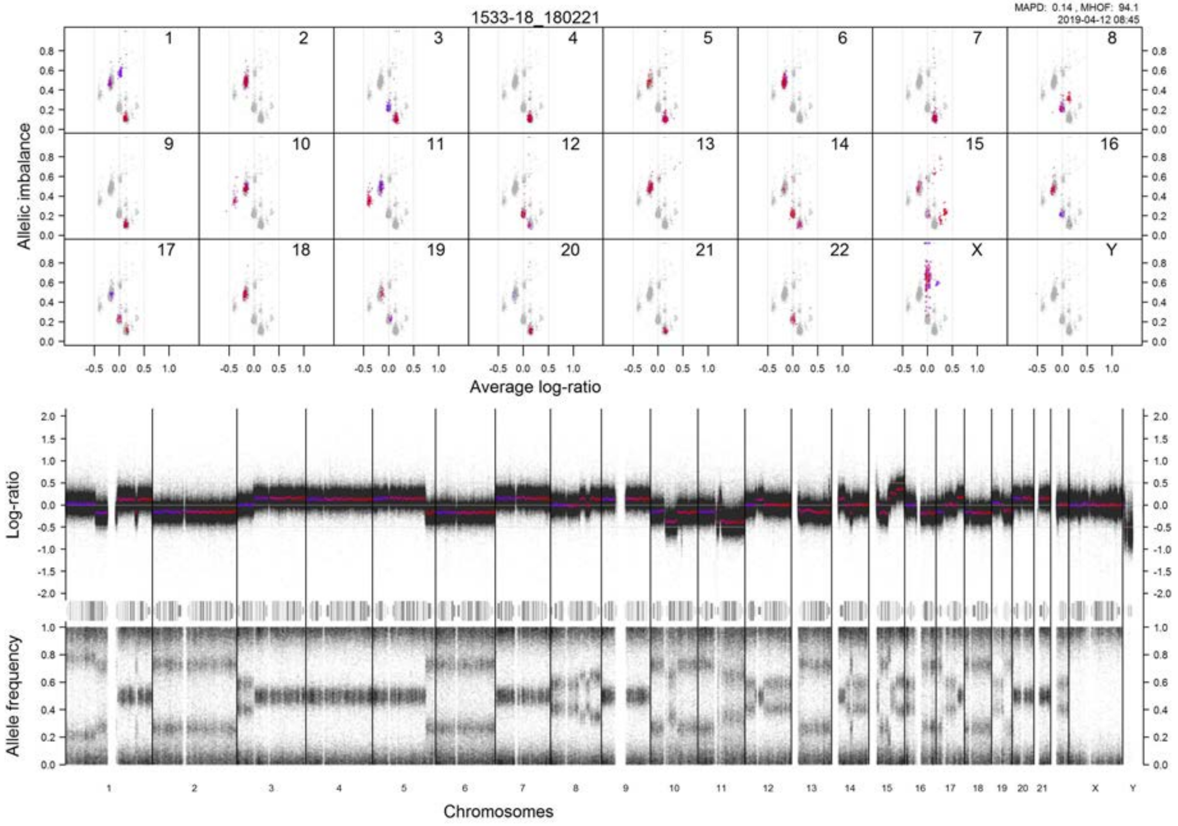

Surgical specimen

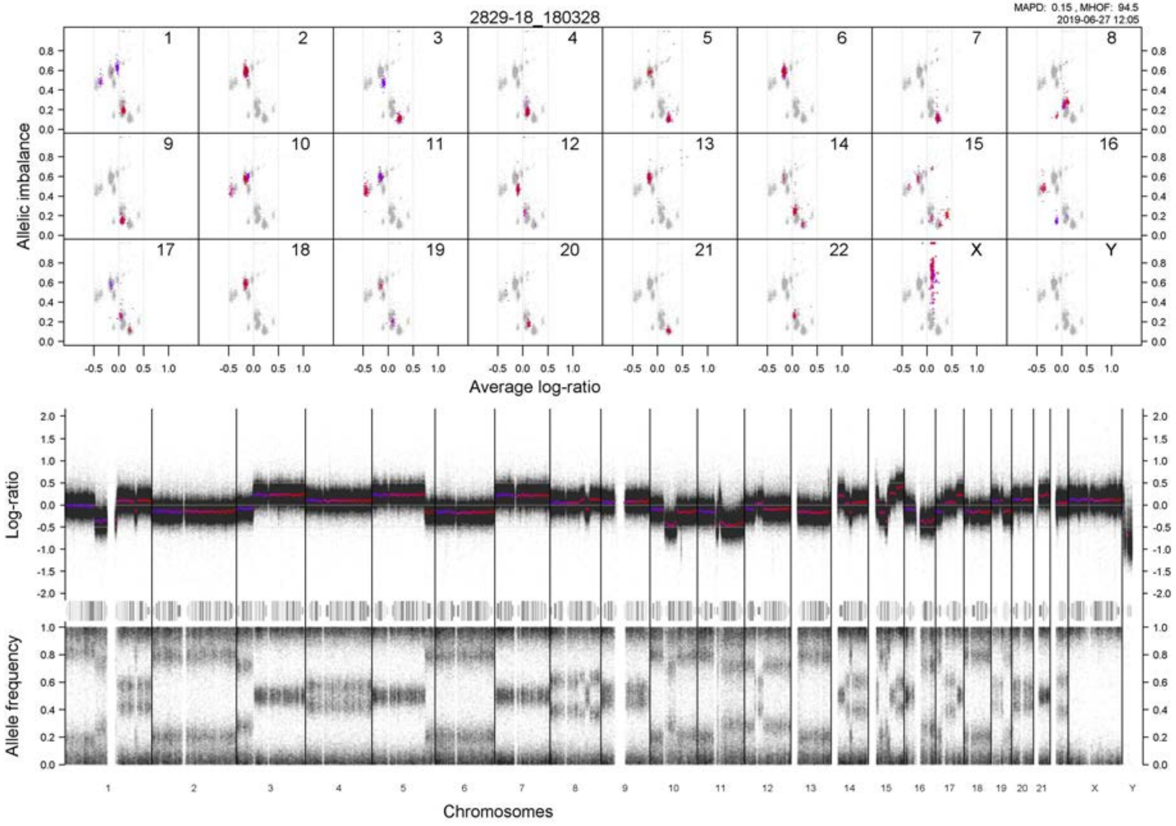

Case 105 (CNB) Schwannoma

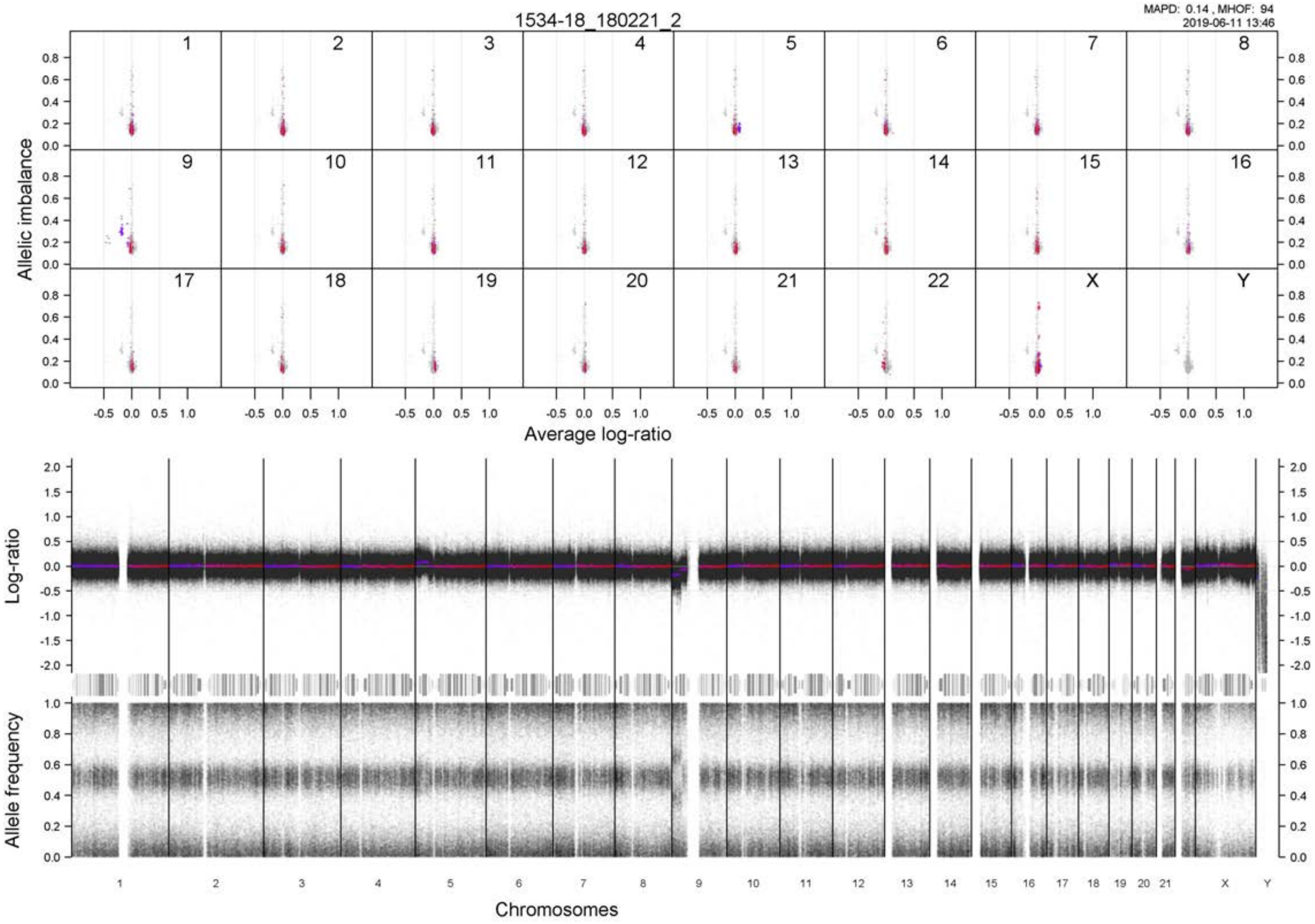

Case 107

Schwannoma

CNB

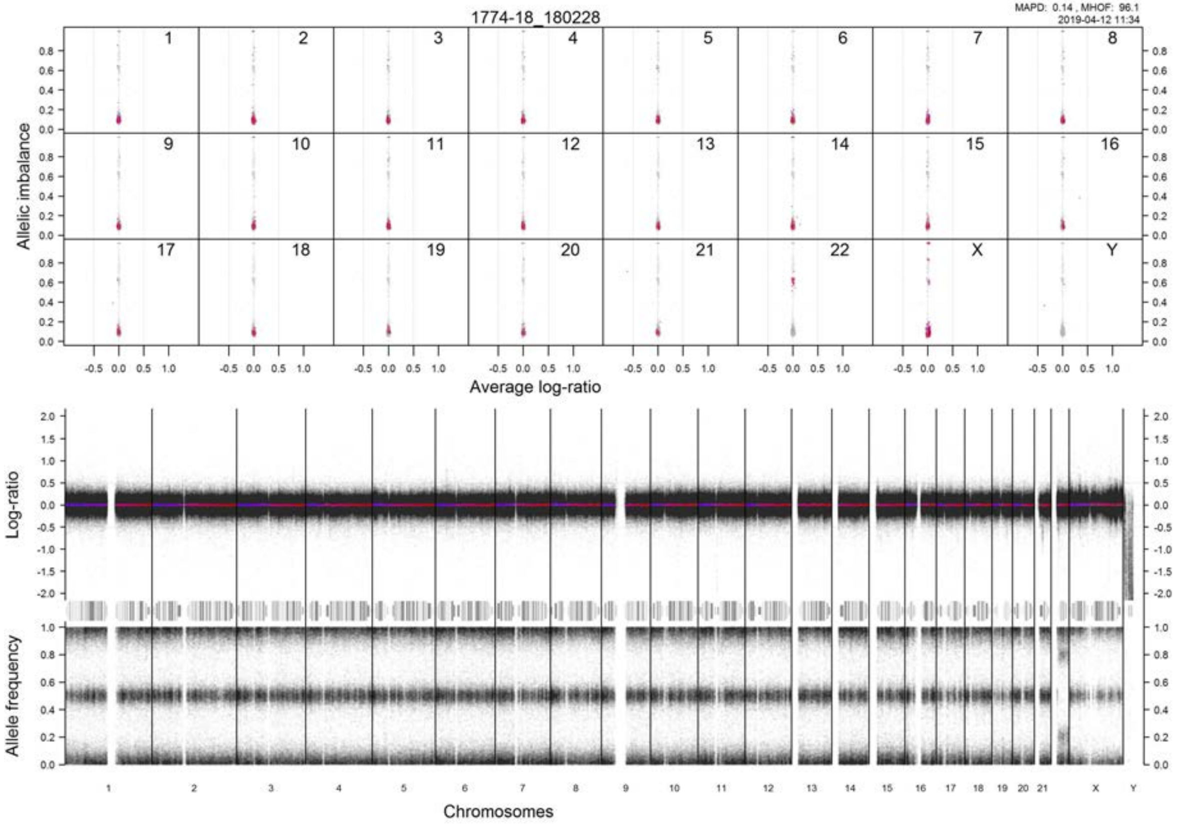

Surgical specimen

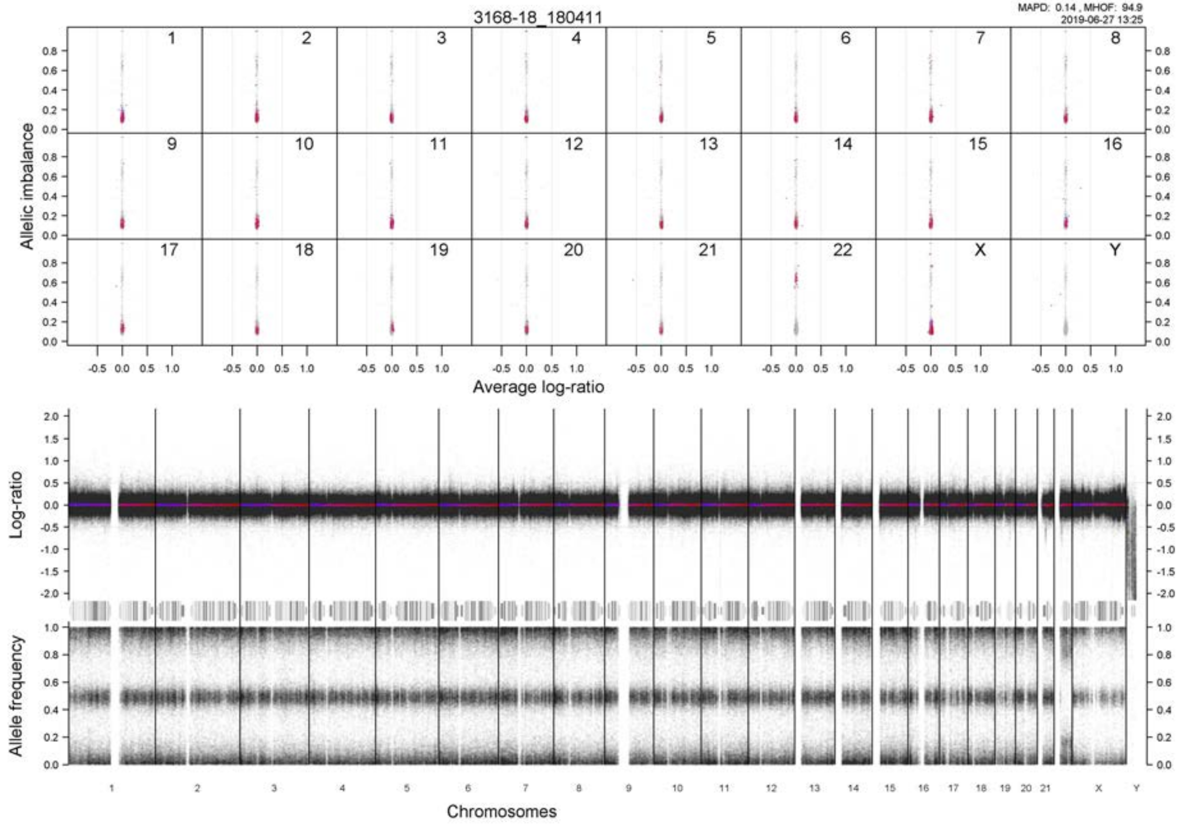

Case 108

Dedifferentiated liposarcoma

CNB

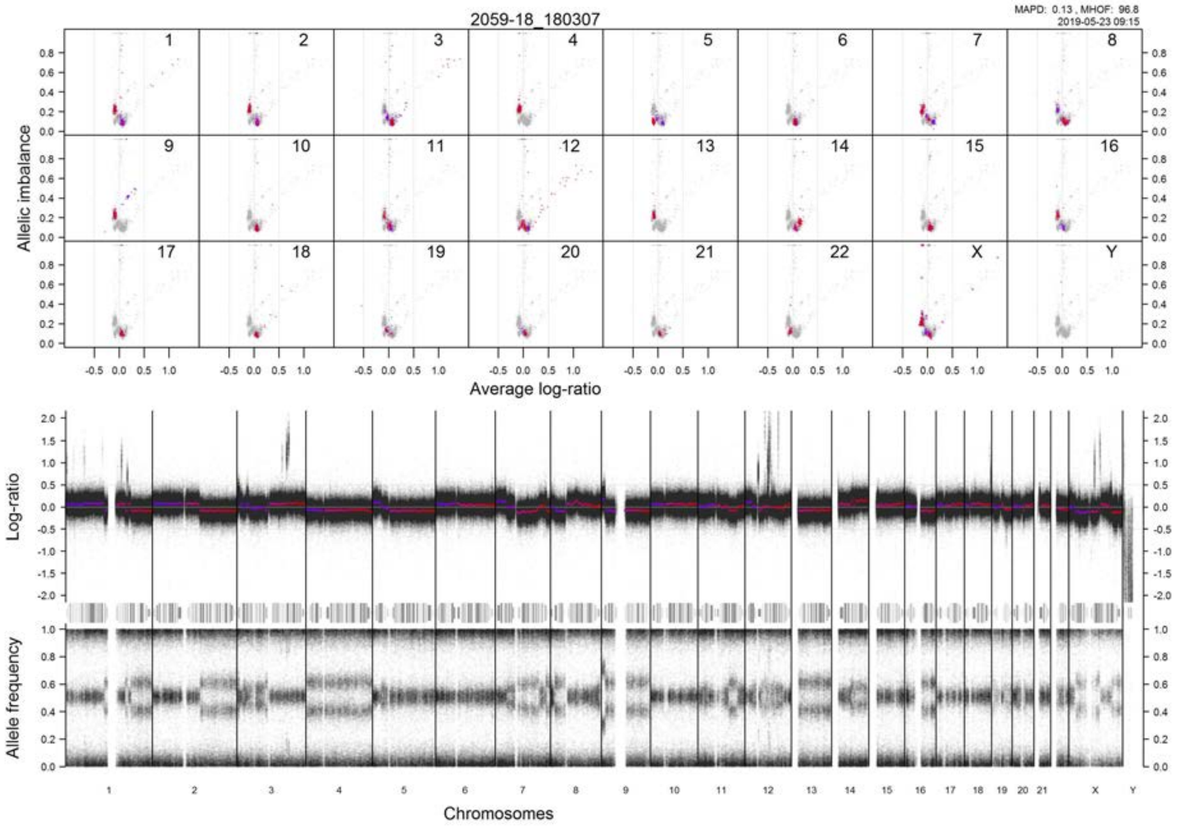

Surgical specimen

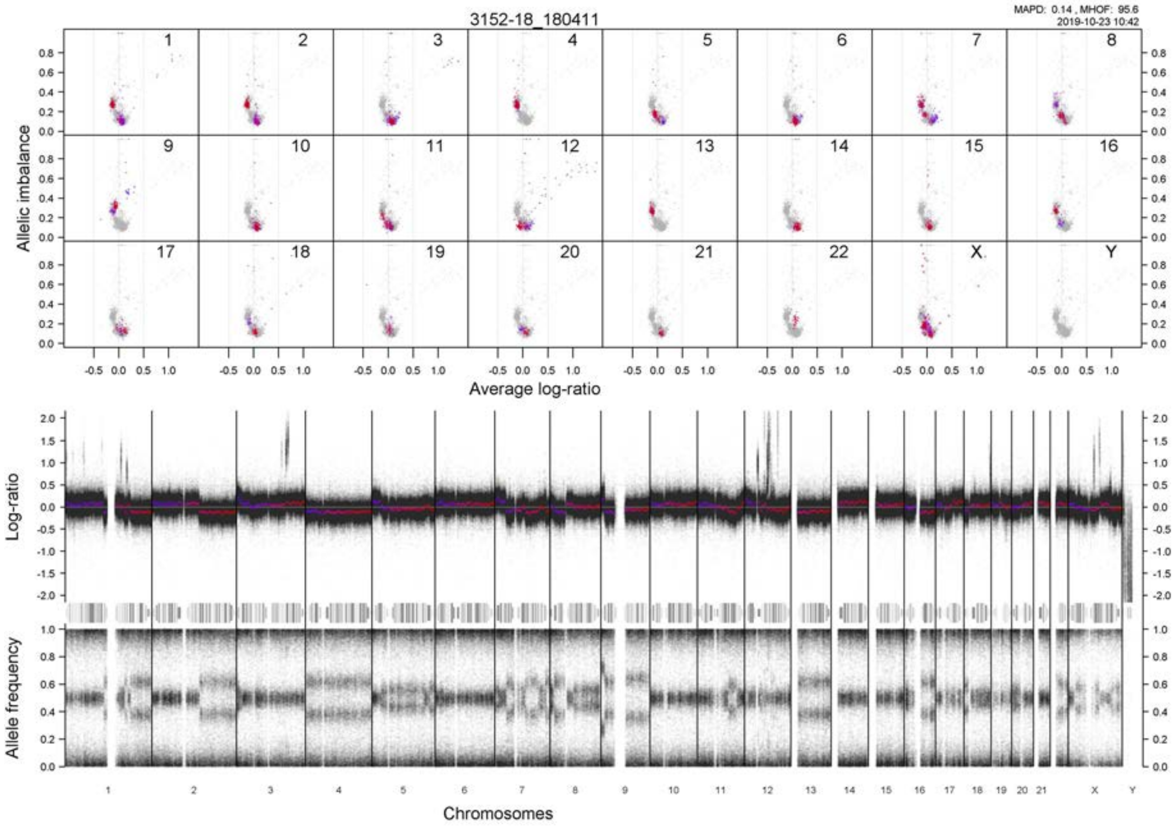

Leiomyosarcoma

Case 109

CNB

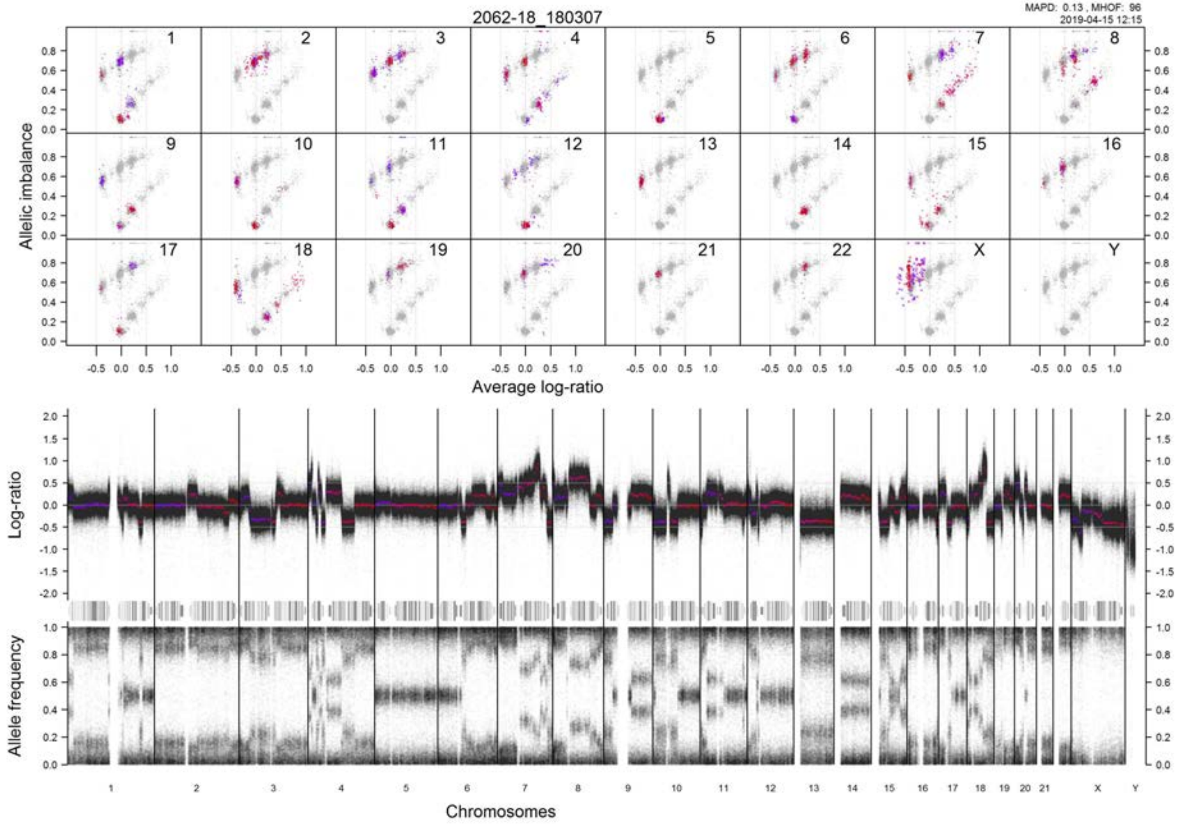

Surgical specimen

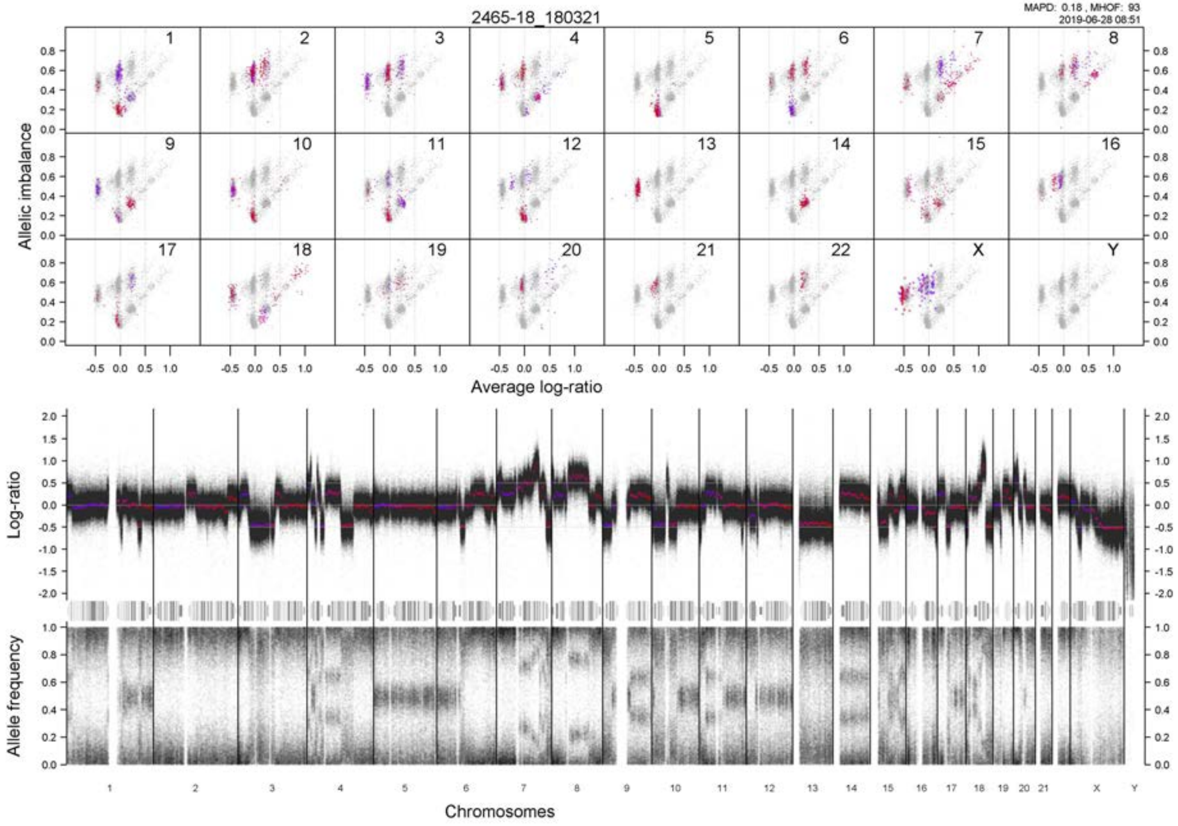

Case 110 (CNB)

Osteosarcoma (metastasis)

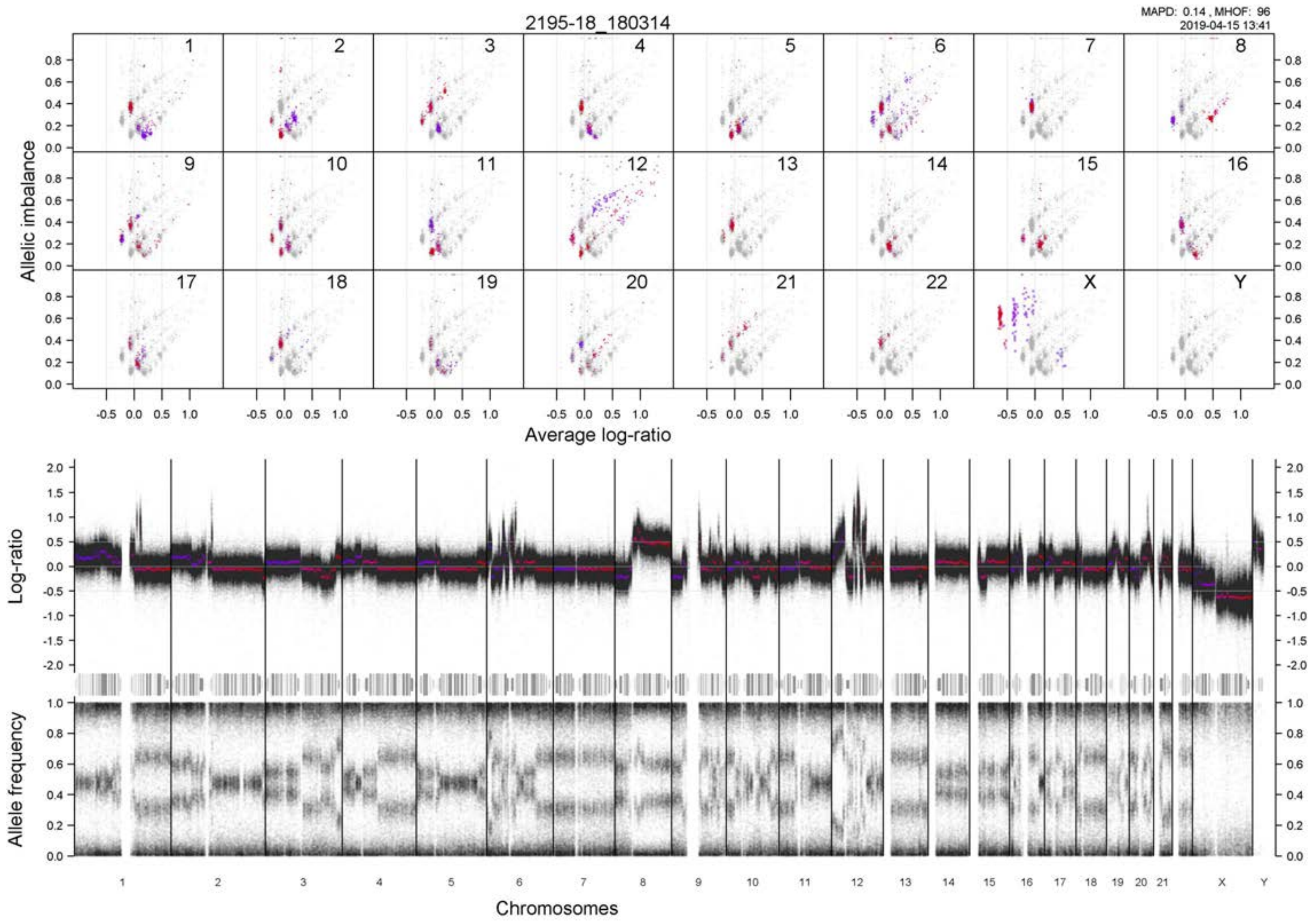

Case 111

Parosteal osteosarcoma

CNB

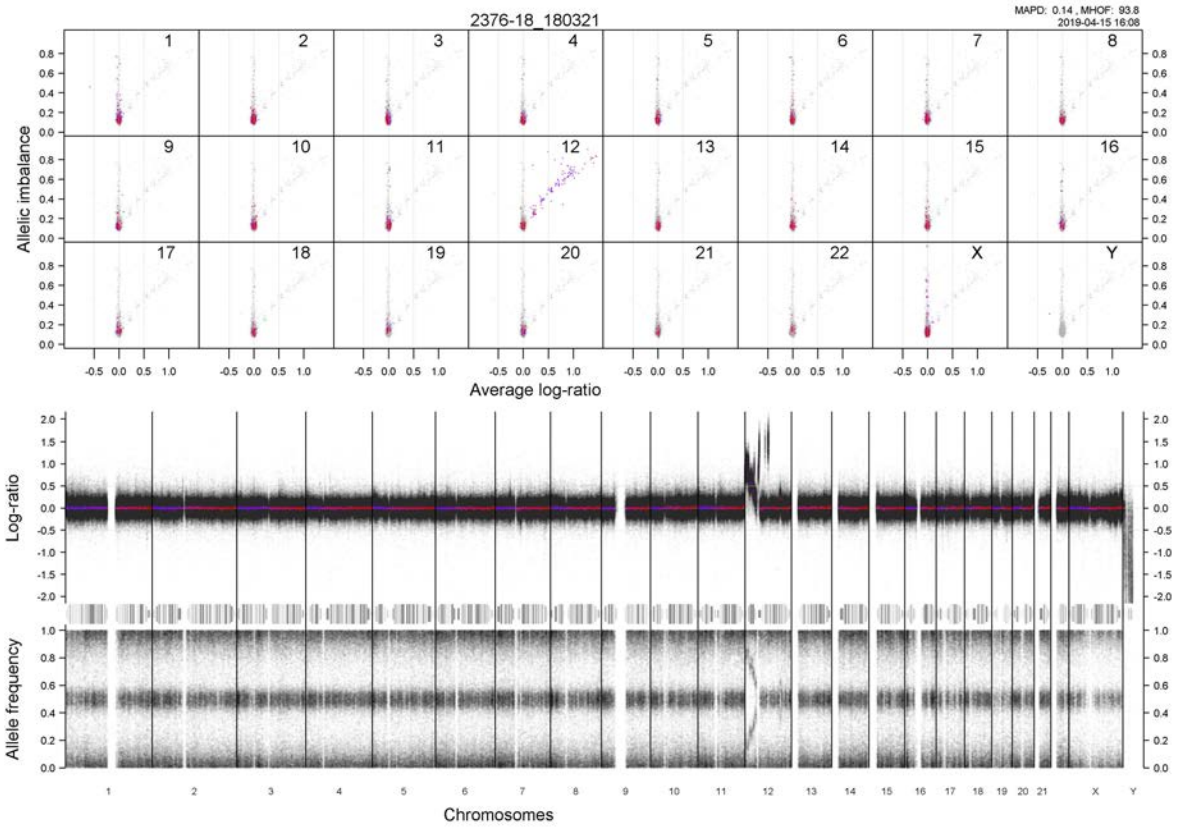

Surgical specimen

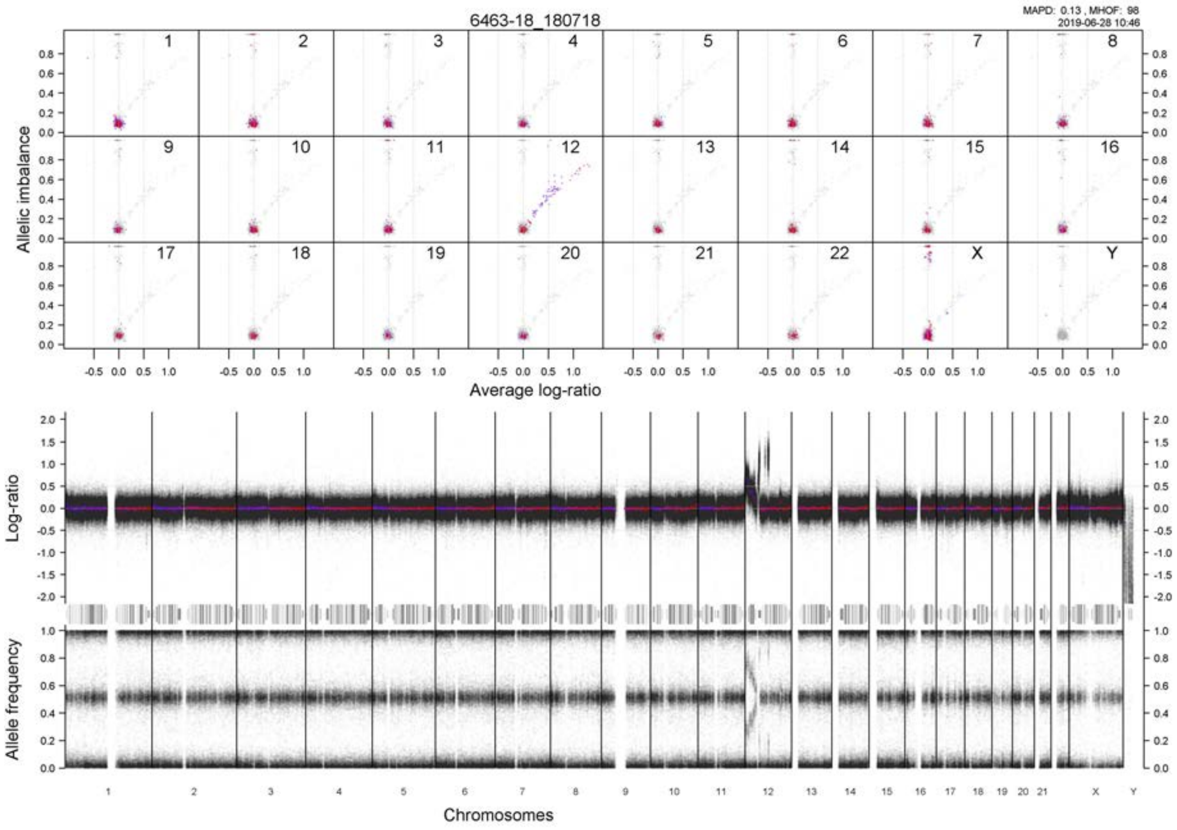

Case 112 (Surgical)

Myxofibrosarcoma

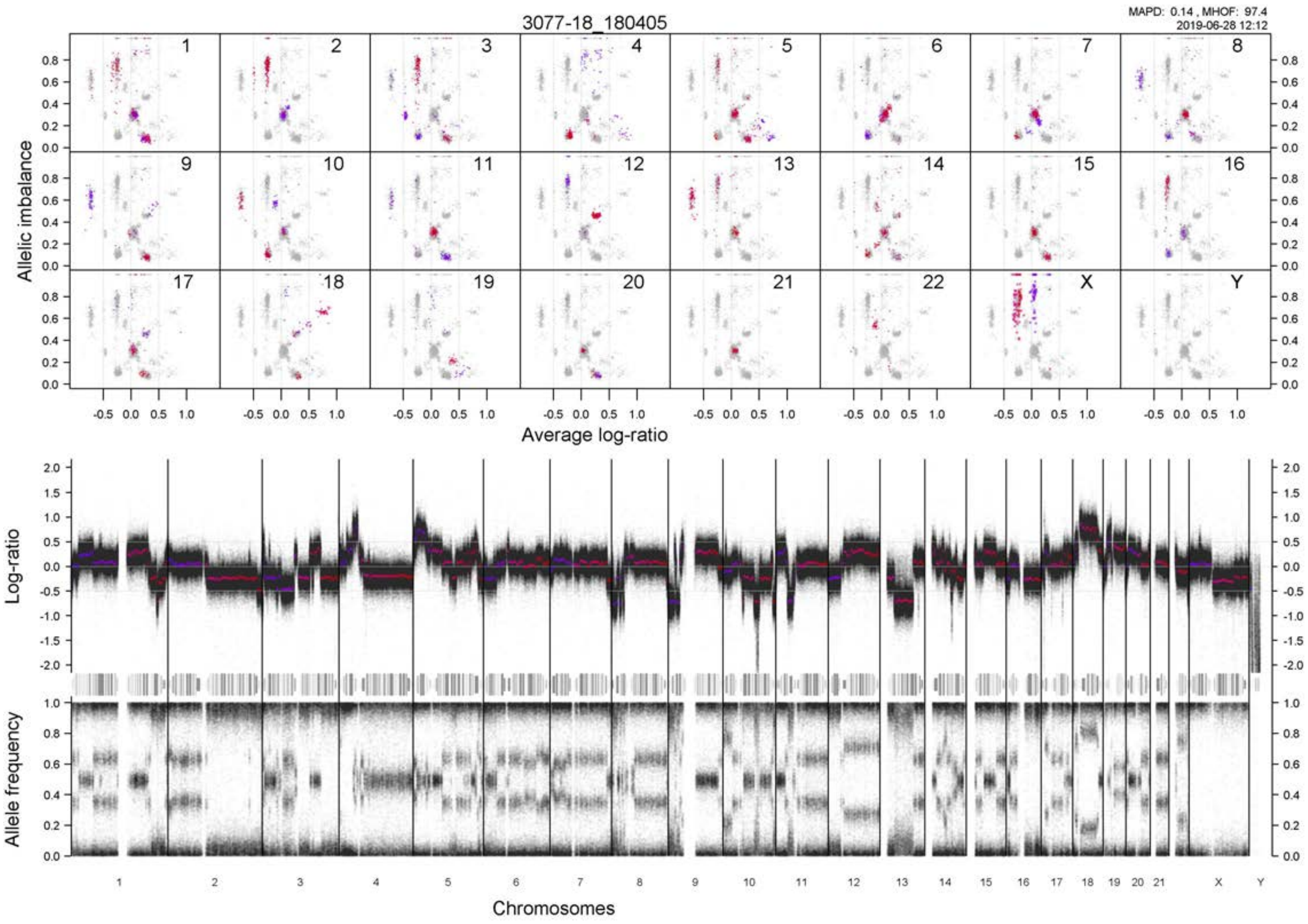

Case 114

Lipoma

CNB

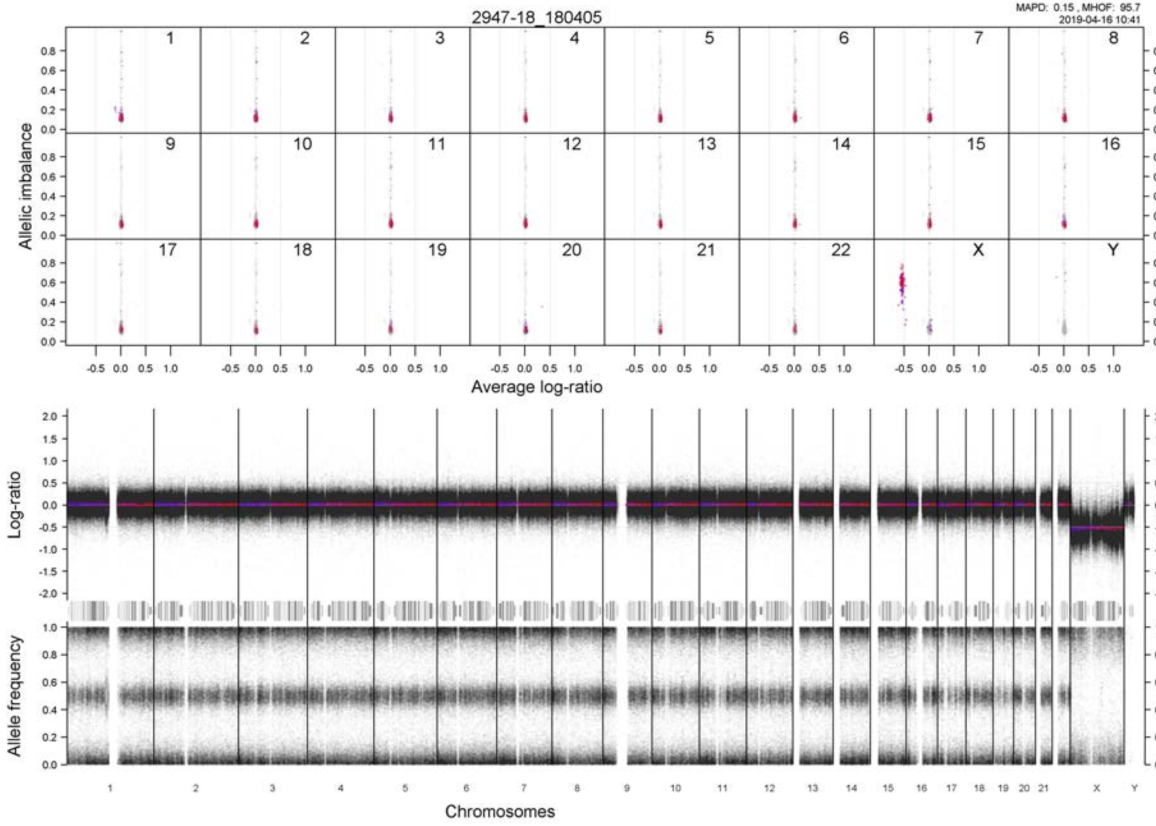

Surgical specimen

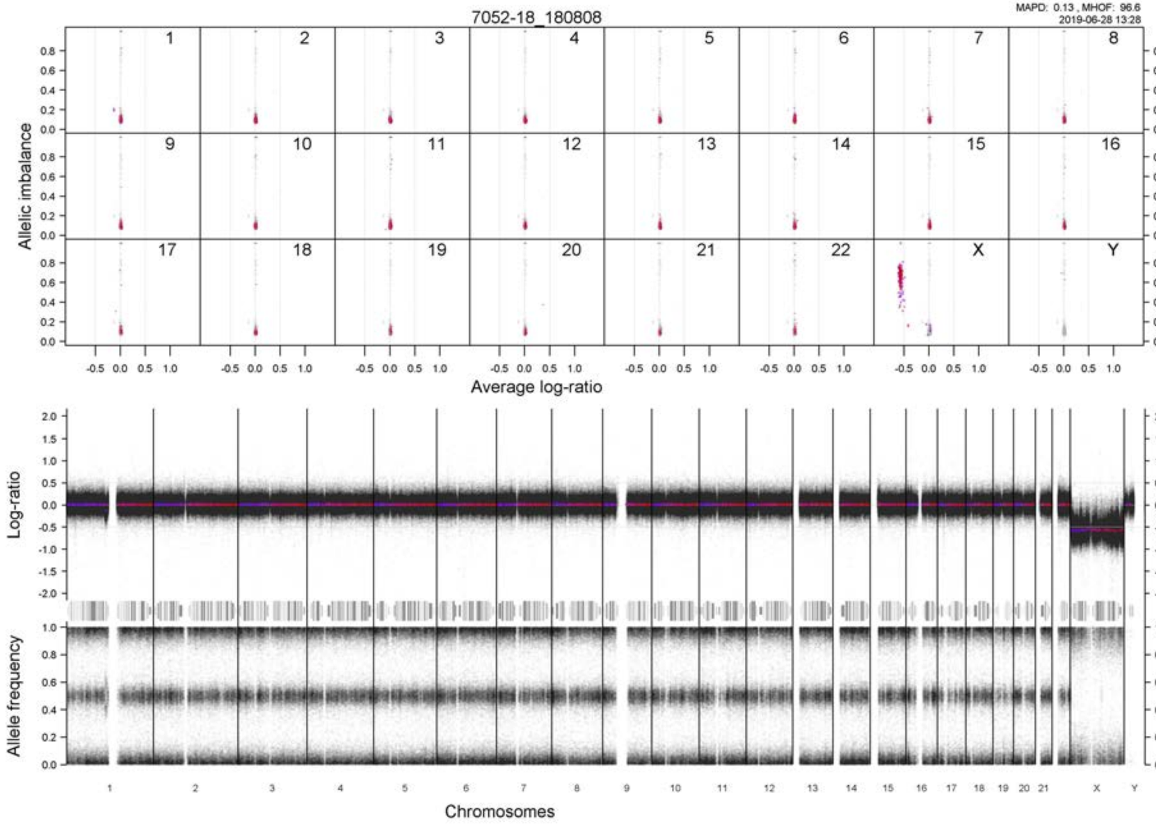

Case 118 (CNB)

Synovial sarcoma

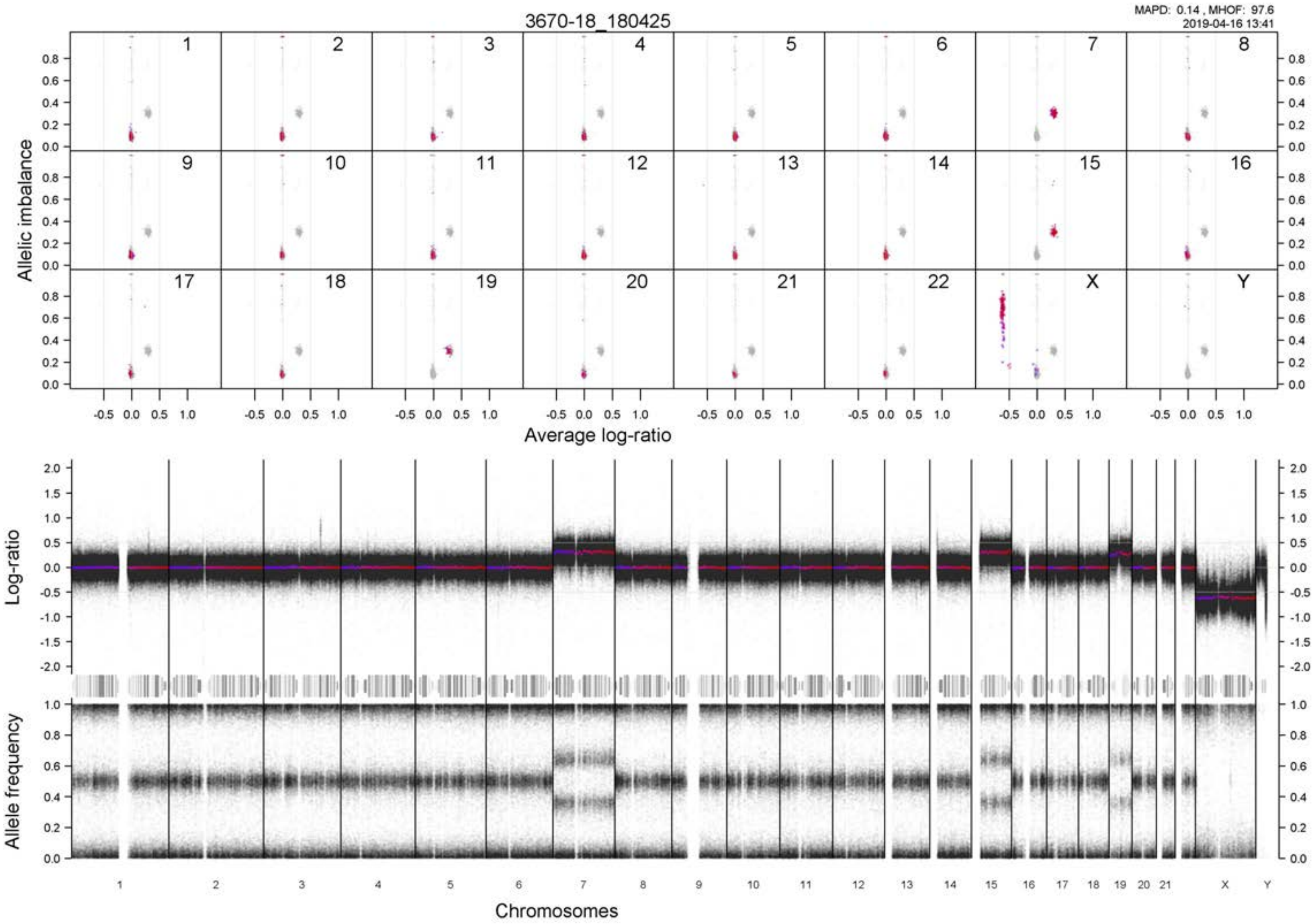

Case 119 (CNB)

Osteosarcoma

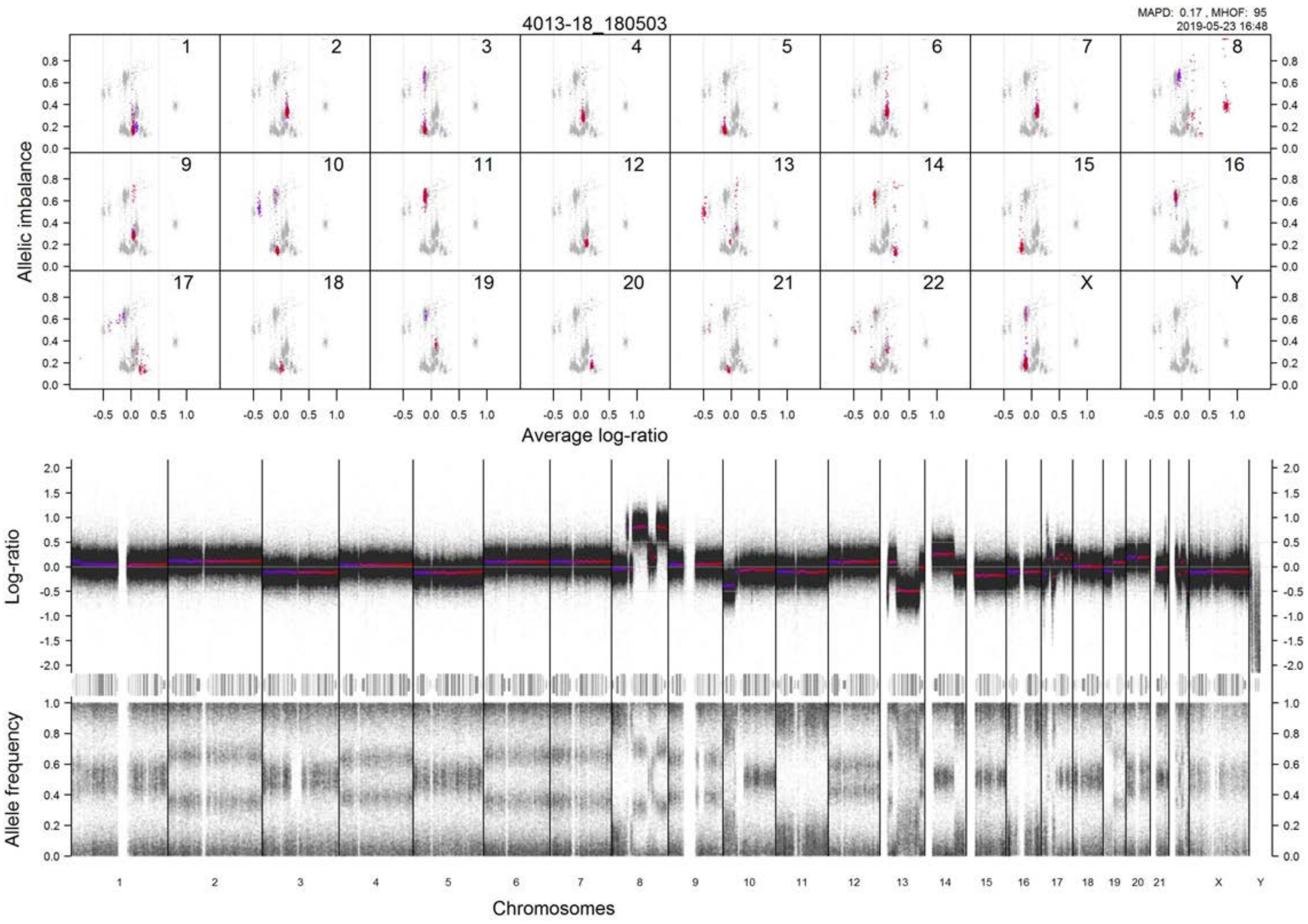

Case 120

Atypical lipomatous tumor

CNB

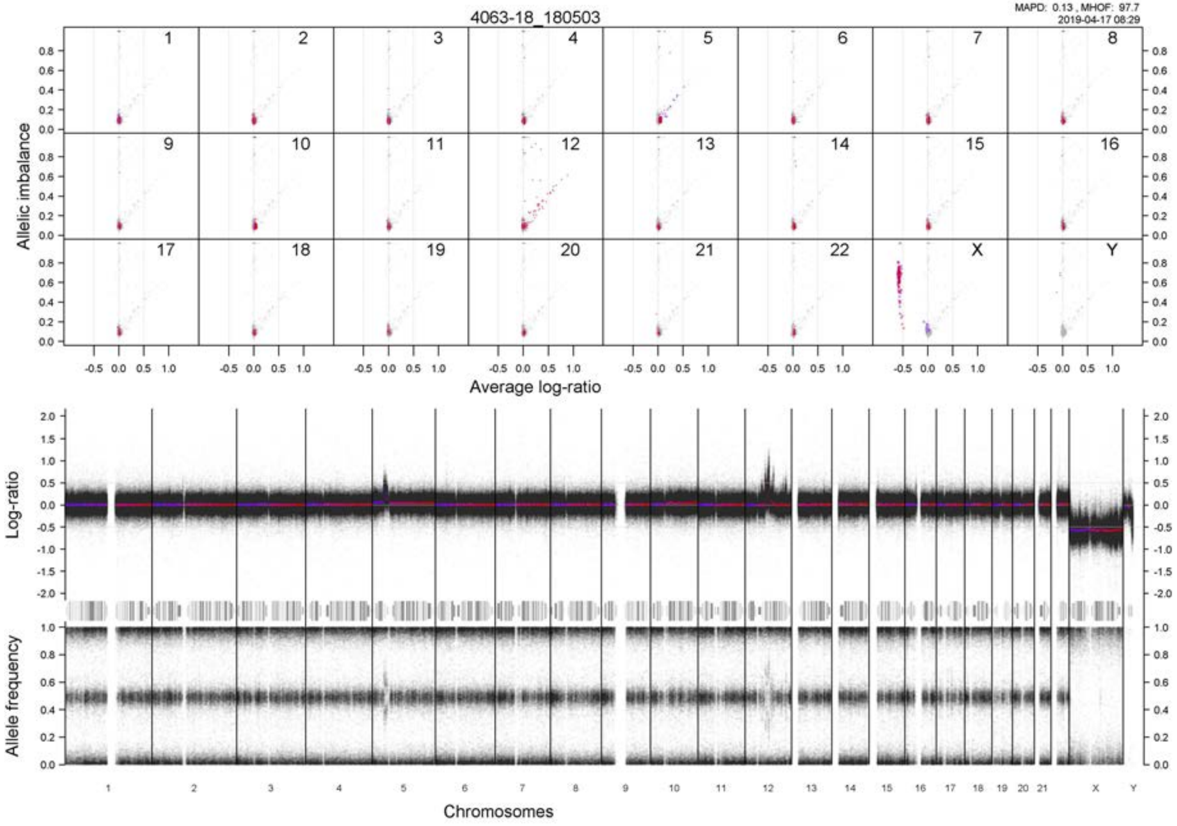

Surgical specimen

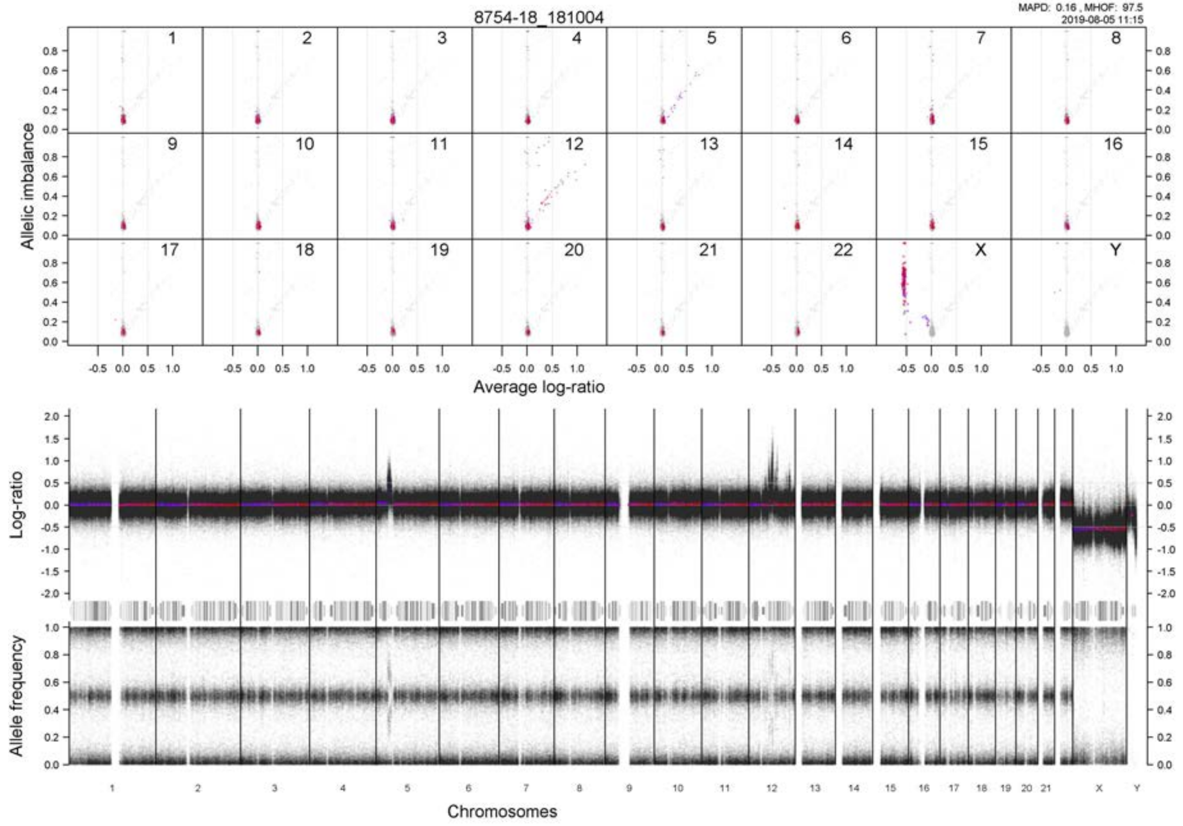

Case 121 GIST

CNB

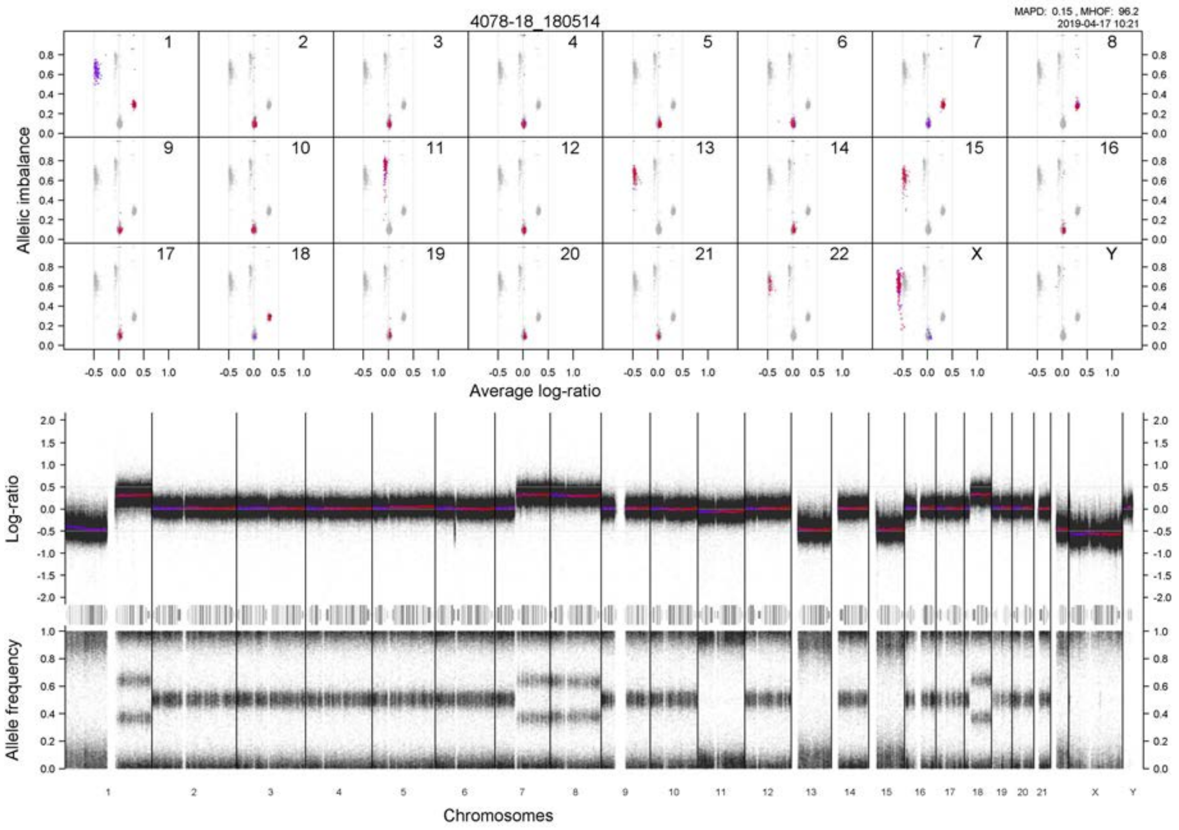

Surgical specimen

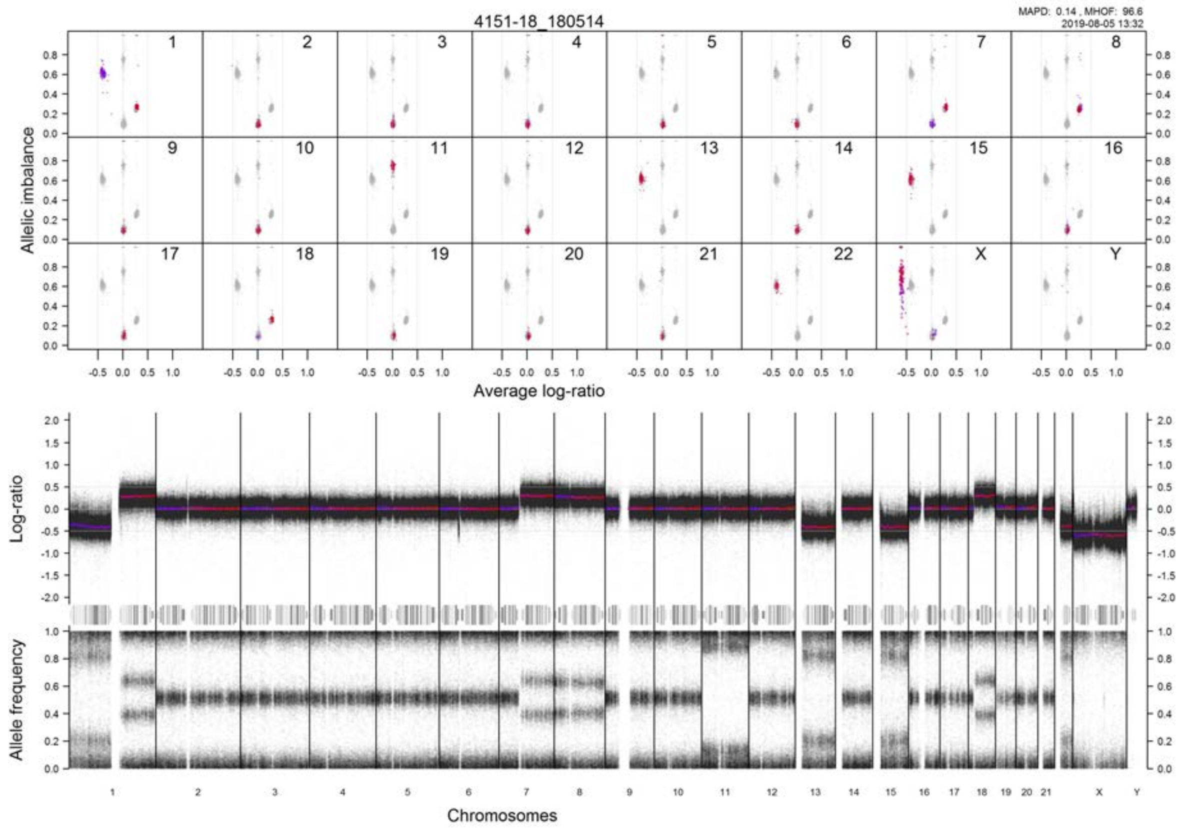

Case 122 (Surgical) Osteochondroma/Chondrosarcoma

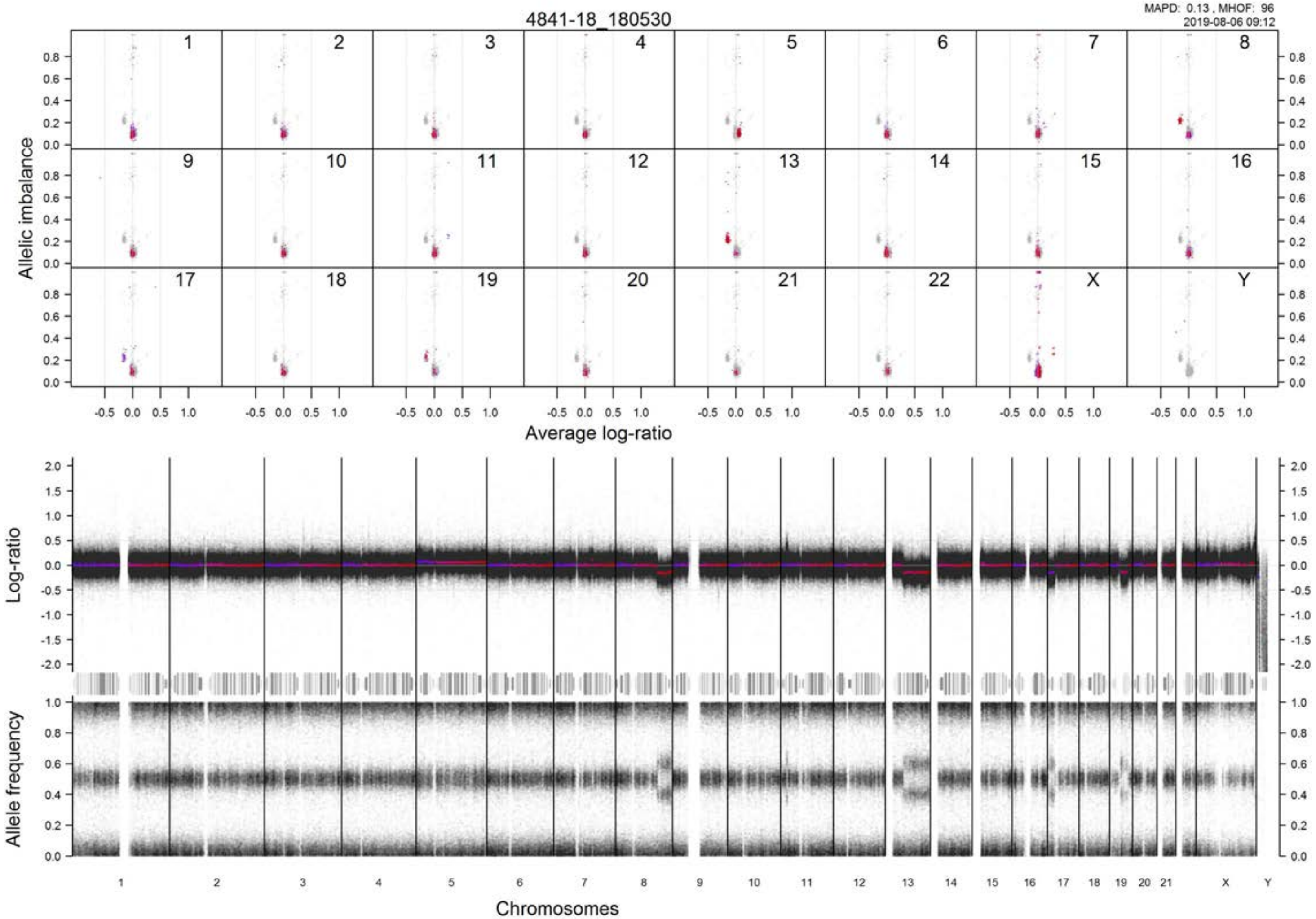

Case 124 (CNB)

Myxofibrosarcoma

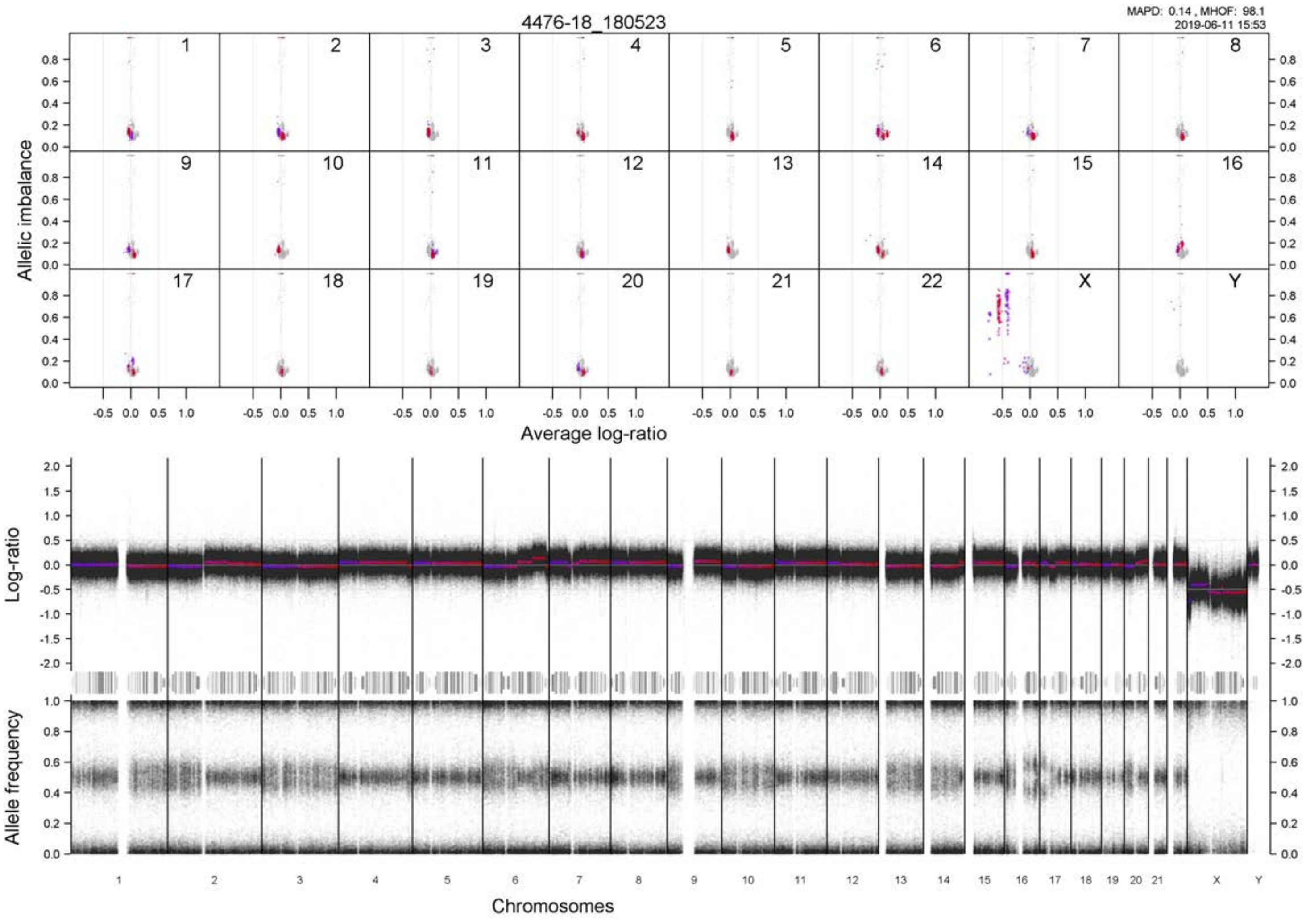

Case 125 (CNB)

Spindle cell lipoma

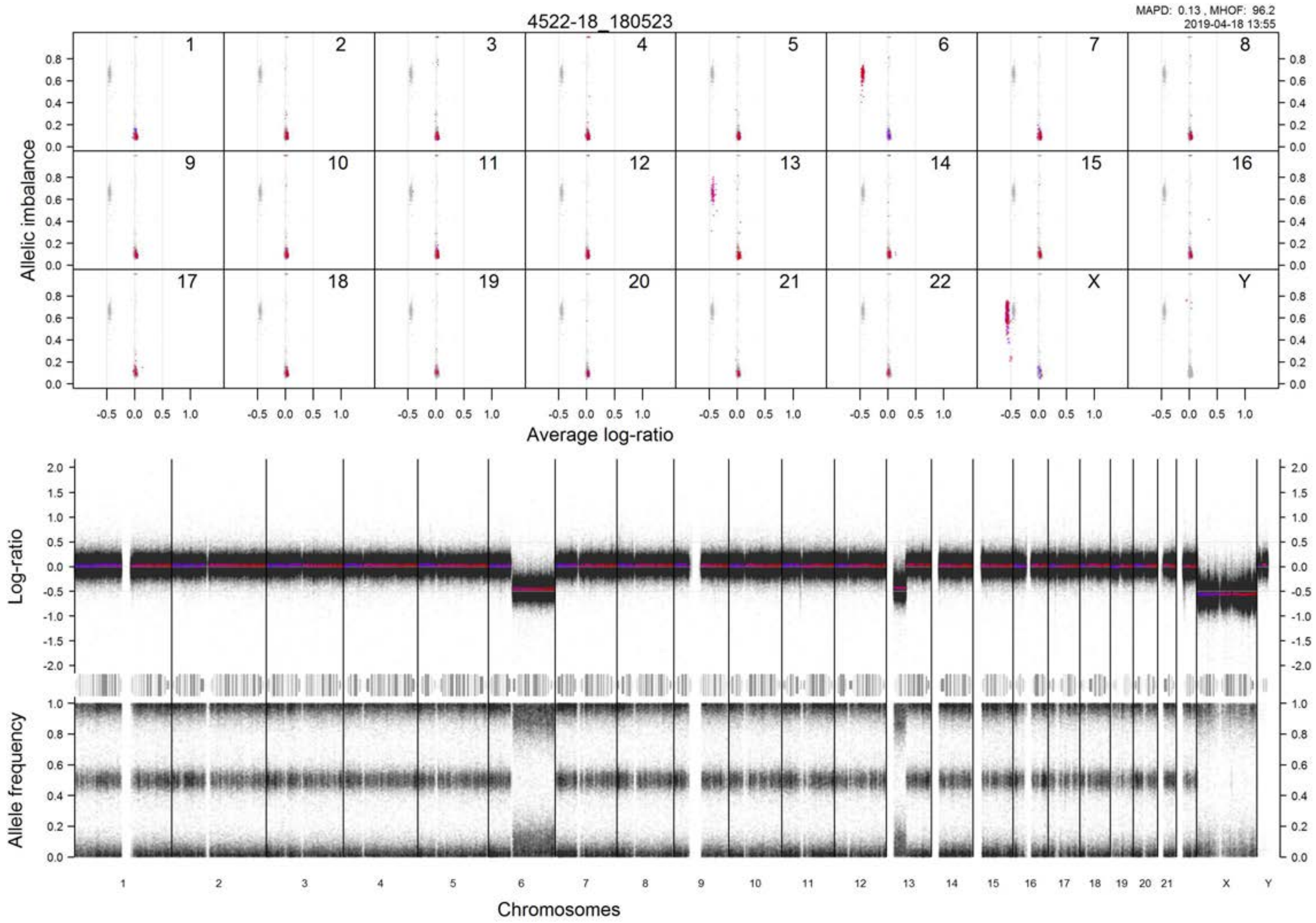

Case 128 (CNB)

Superficial angiomyxoma

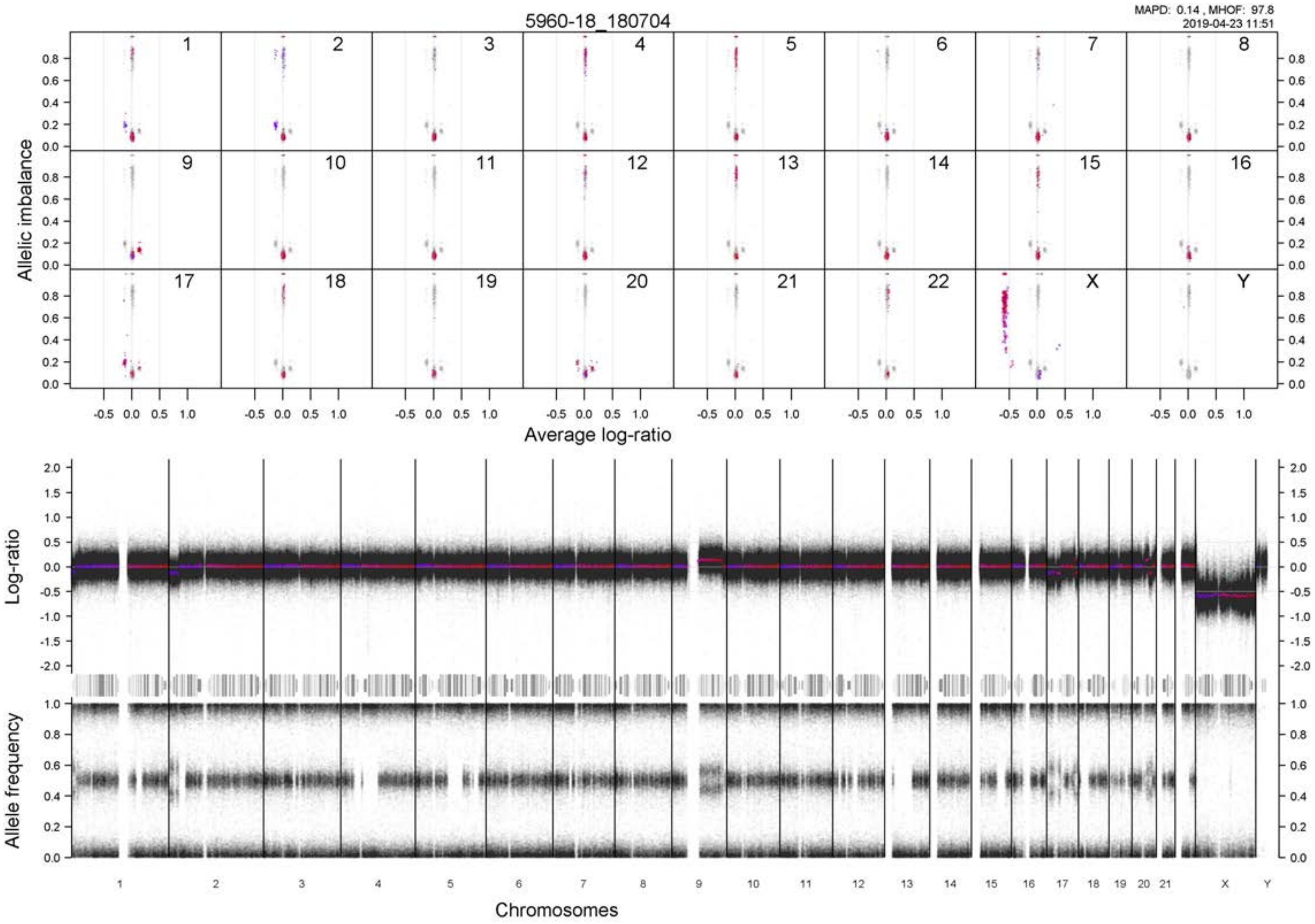

Case 129

UPS

CNB

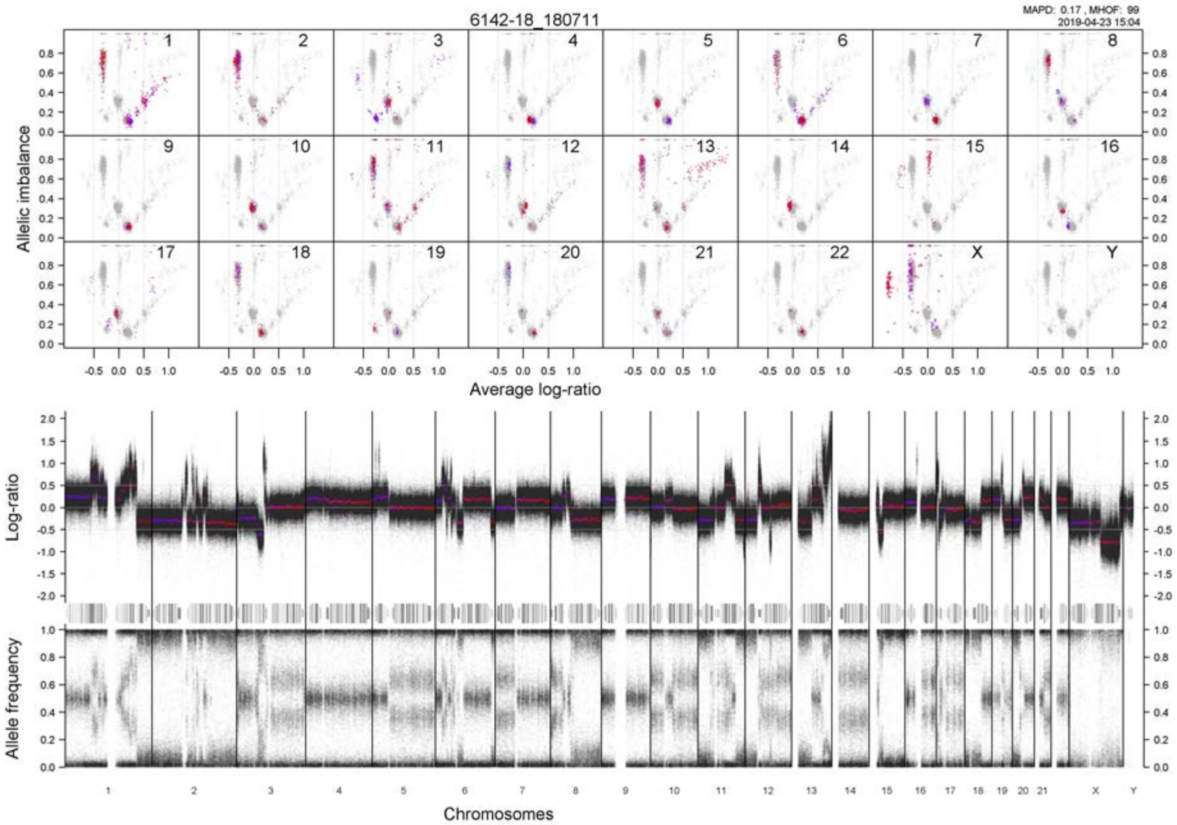

Surgical specimen

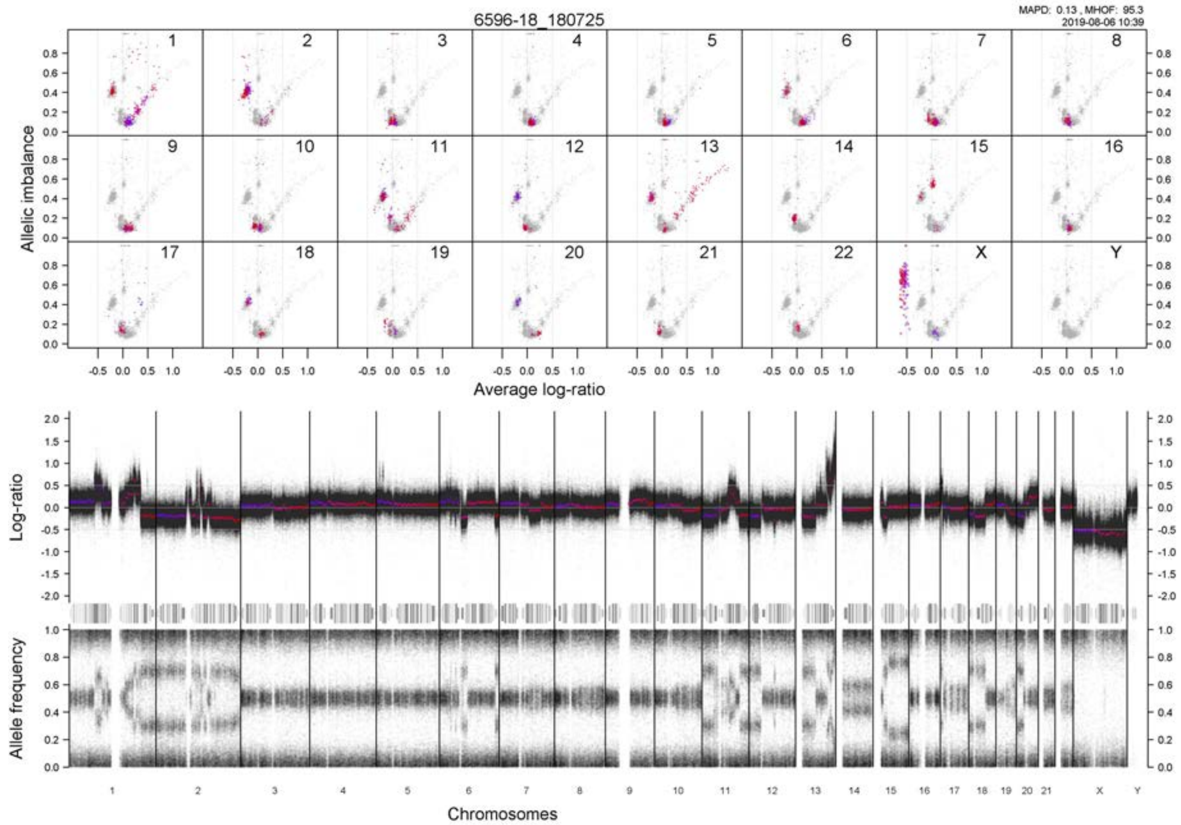

Case 131 (CNB)

Spindle cell sarcoma (probably recurrent MPNST)

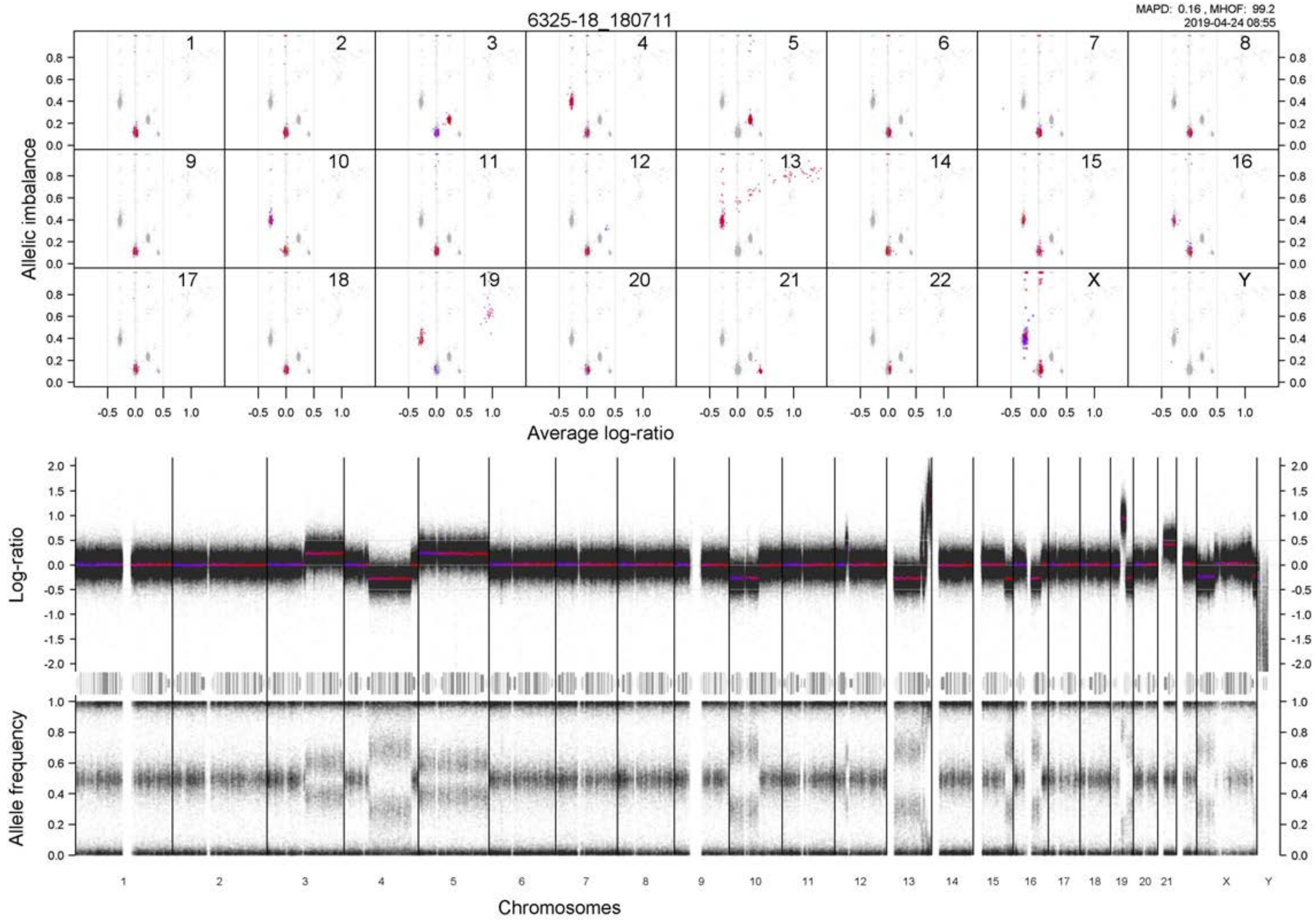

Case 132 UPS

CNB

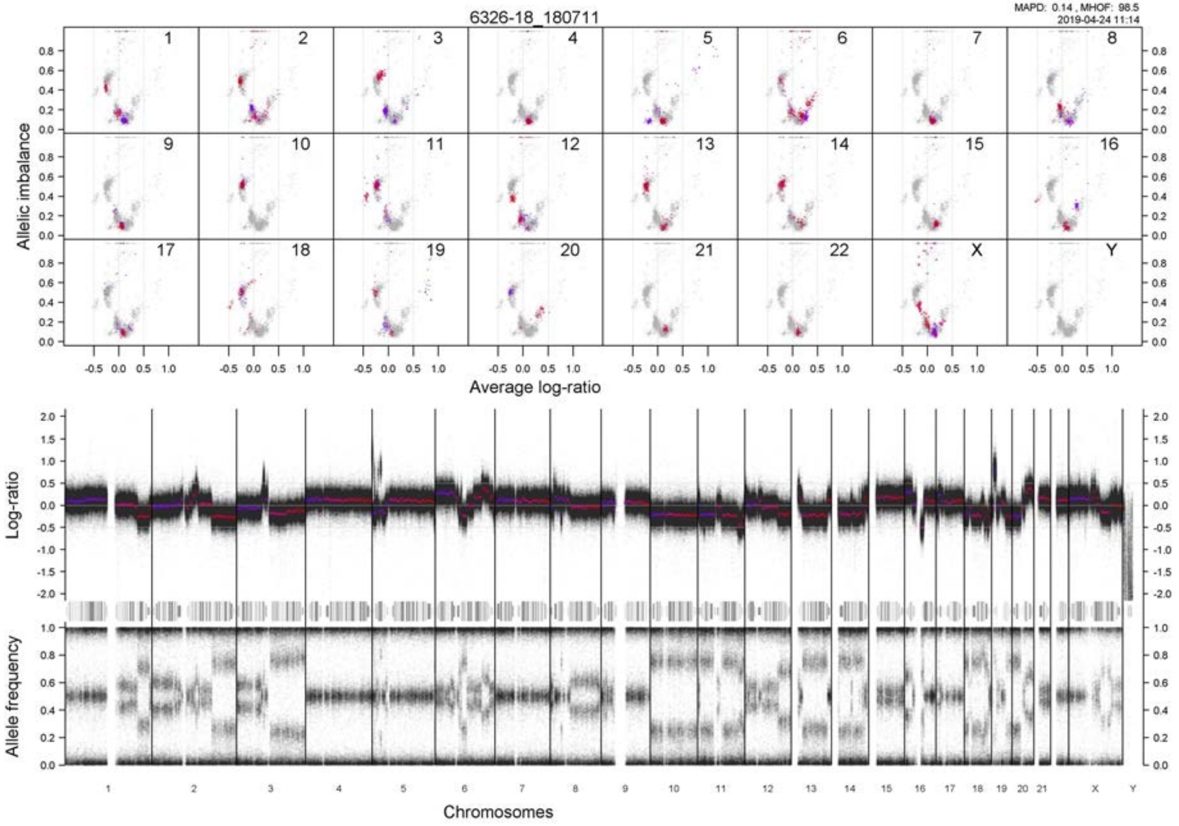

Surgical specimen

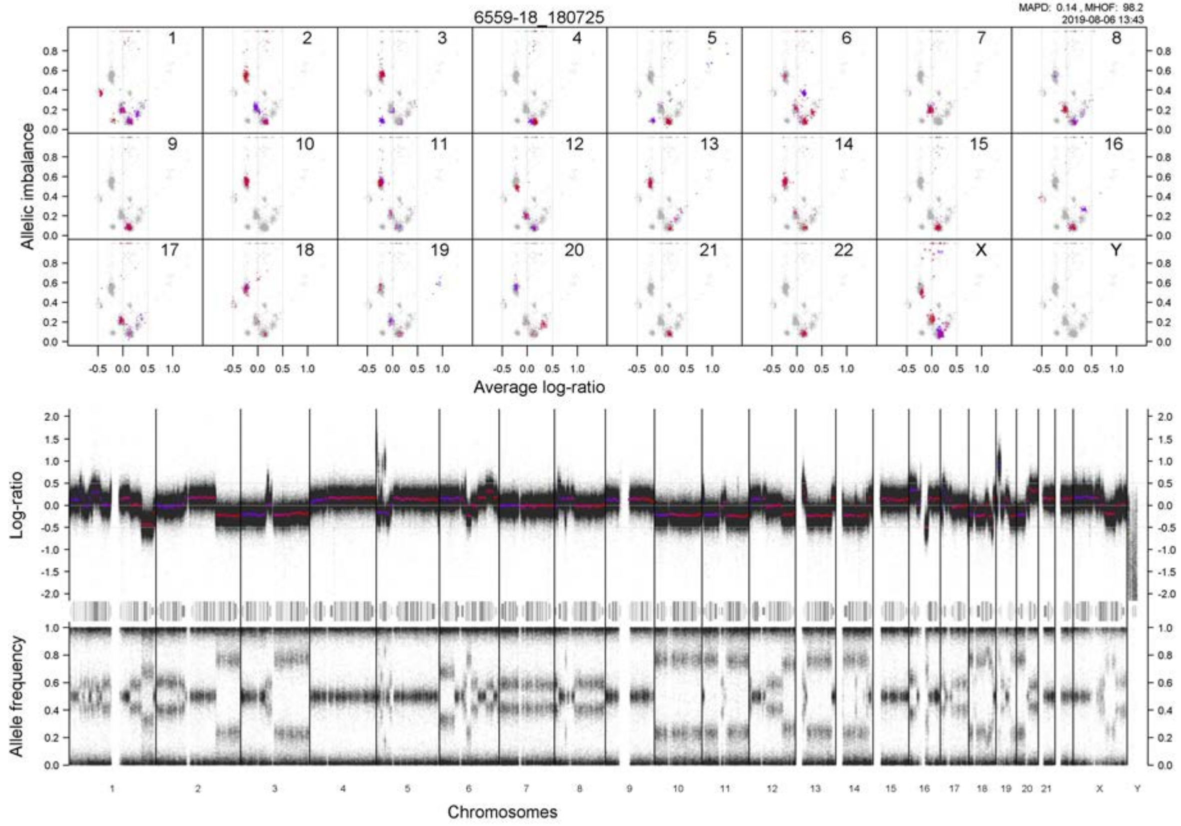

Case 134 Myxofibrosarcoma

CNB

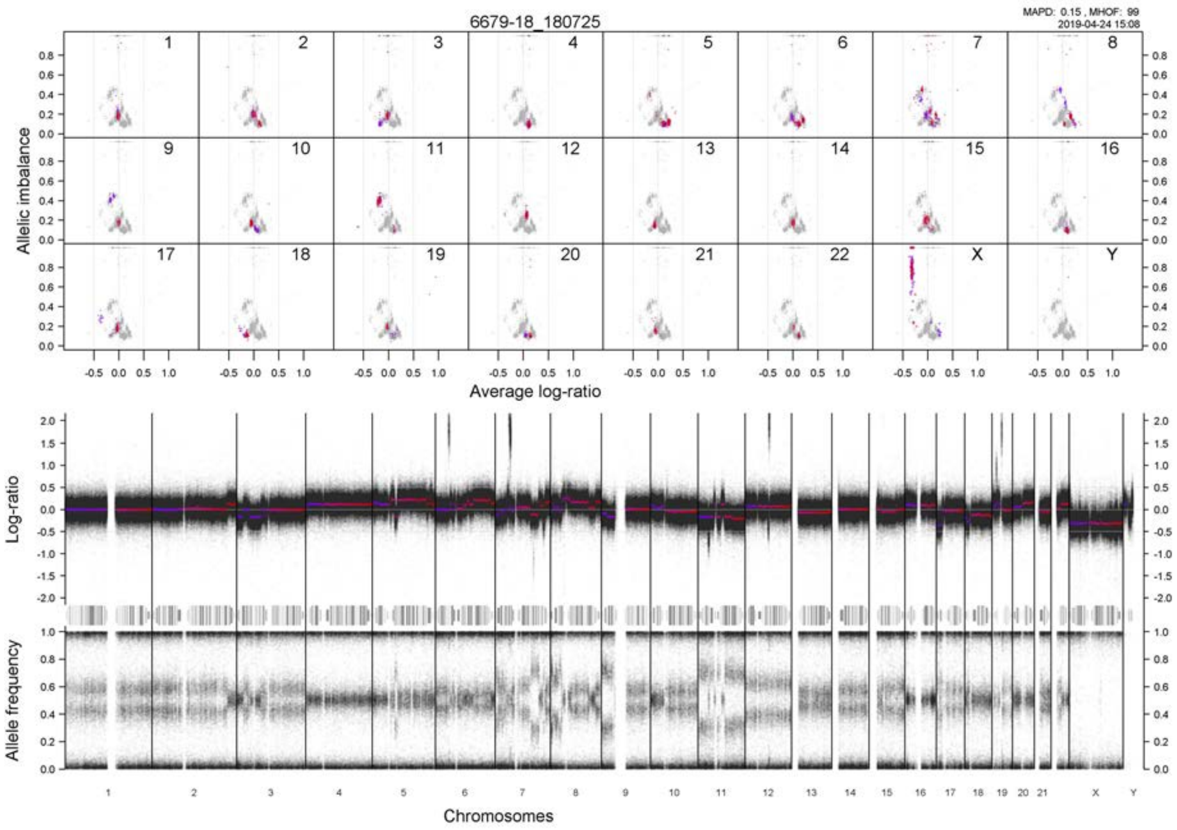

Surgical specimen

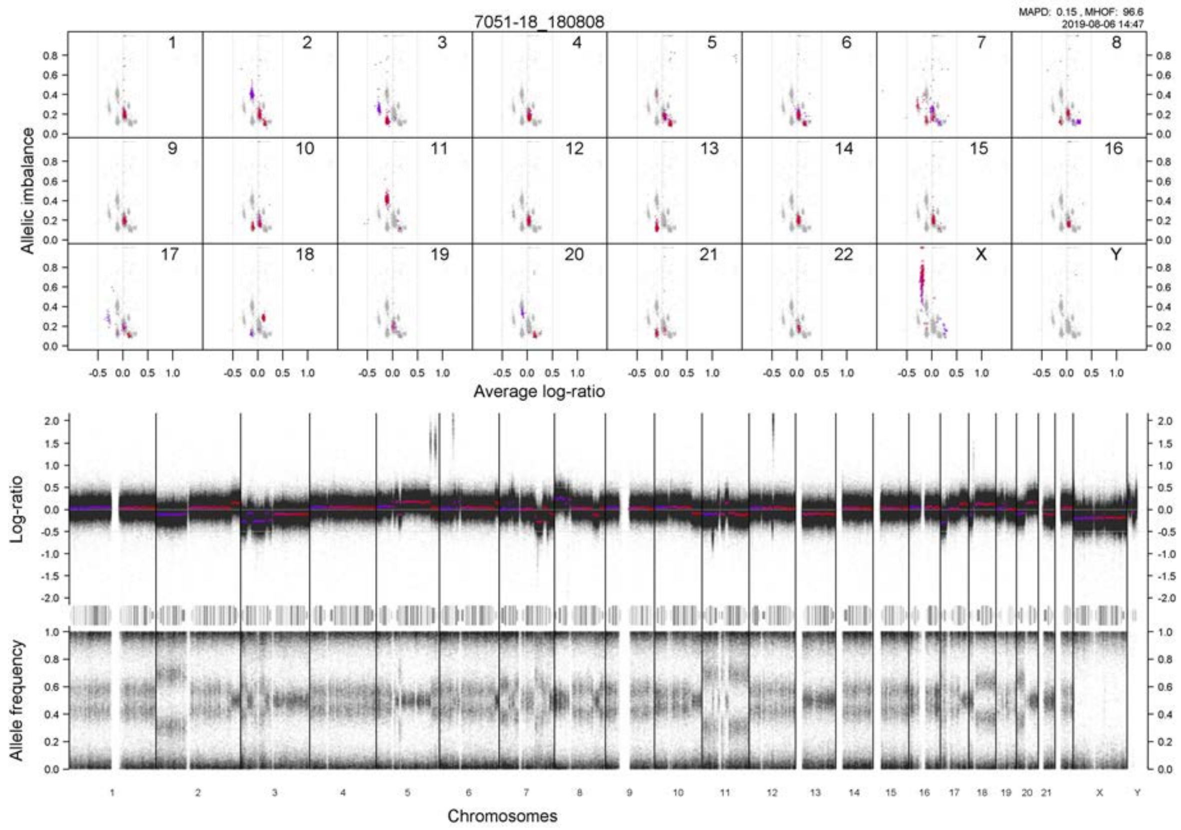

Case 135 (CNB)

Myxoid sarcoma, unknown type

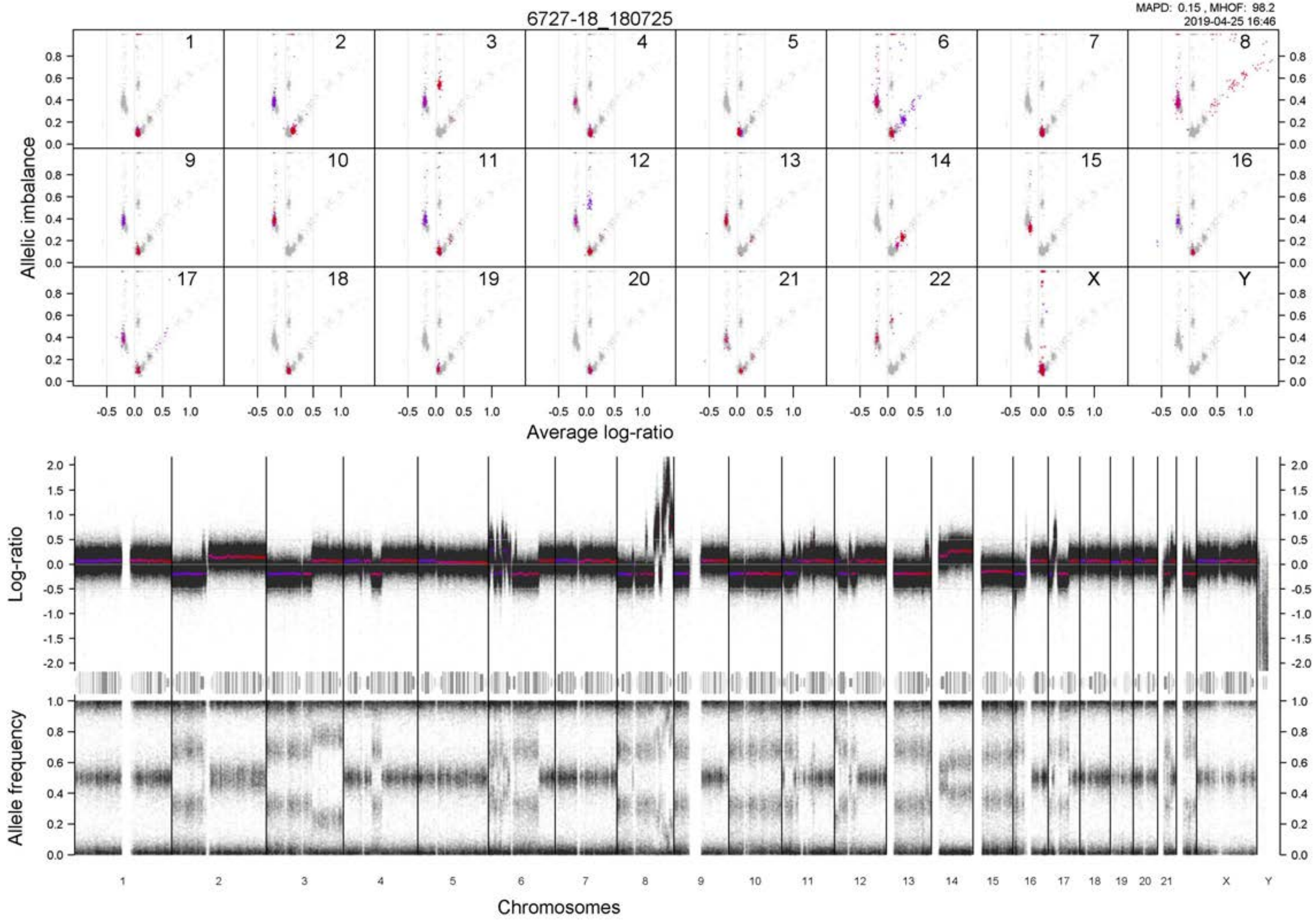

Case 136 UPS

CNB

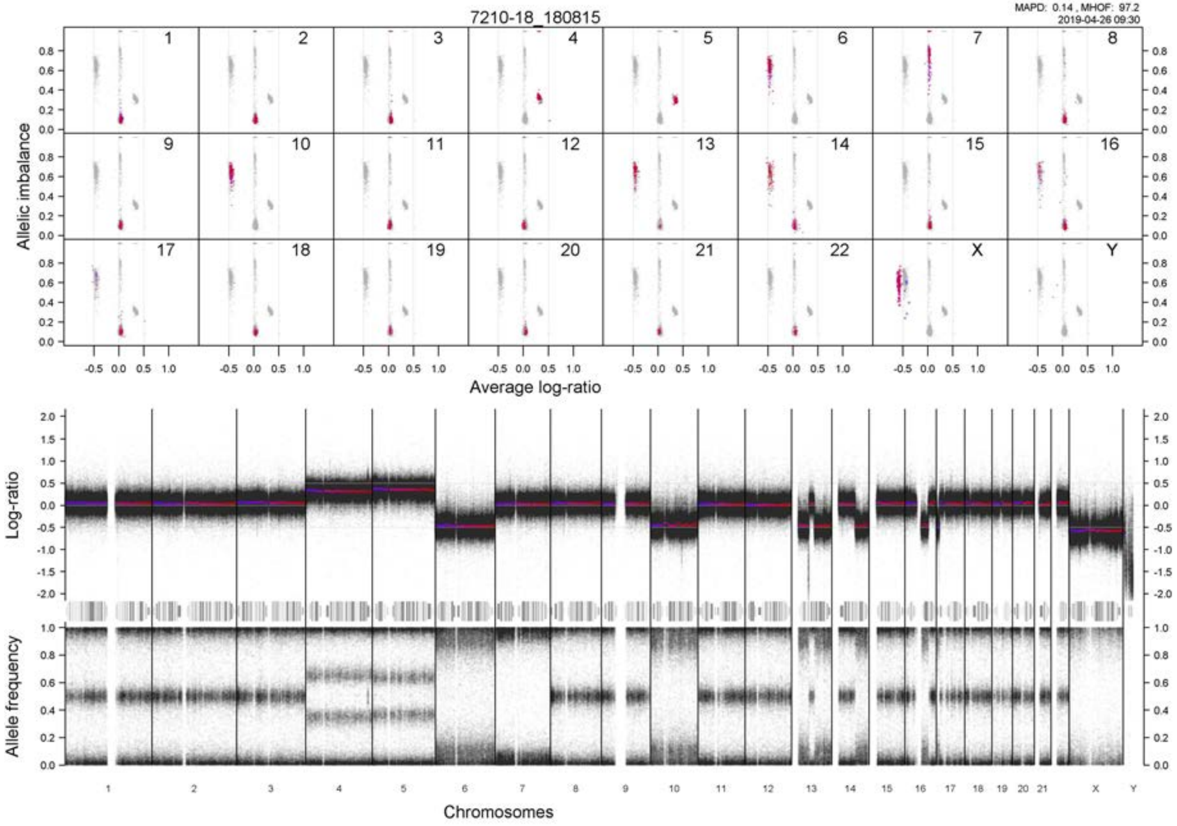

Surgical specimen

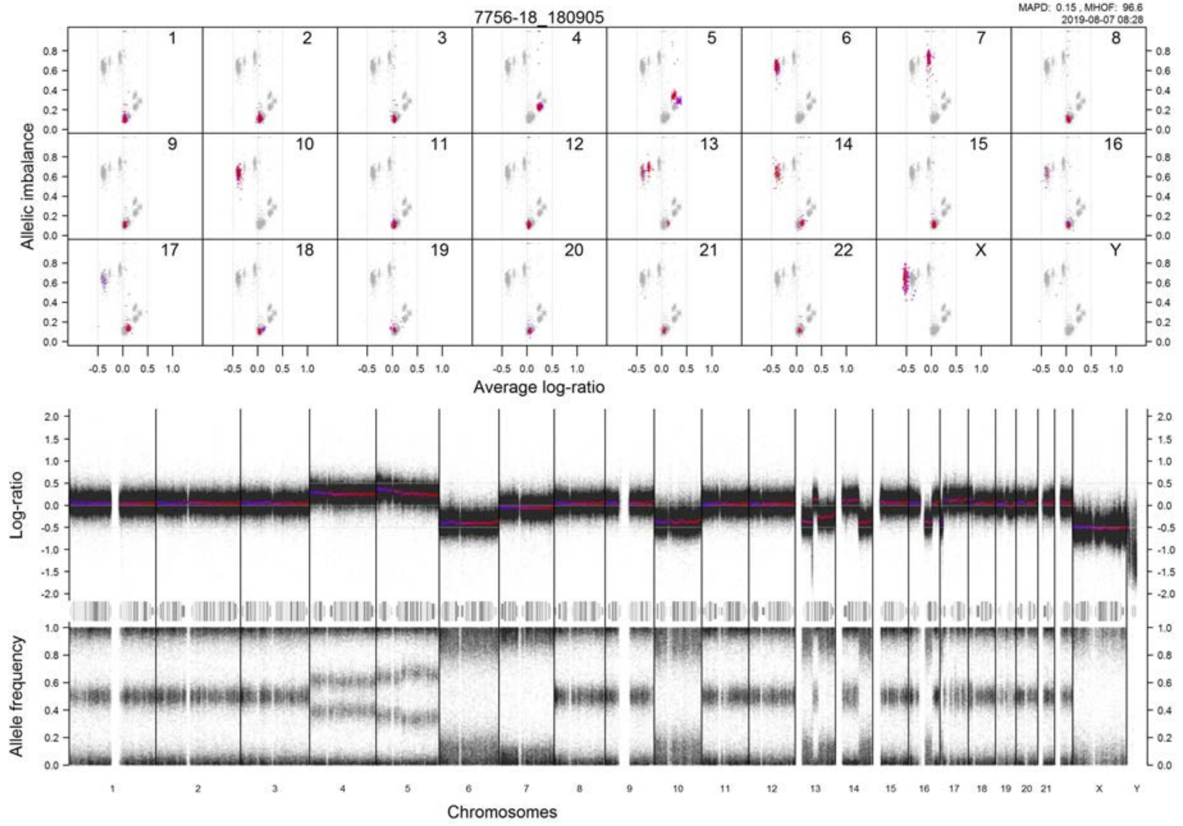

Case 140 Spindle cell rhabdomyosarcoma

CNB

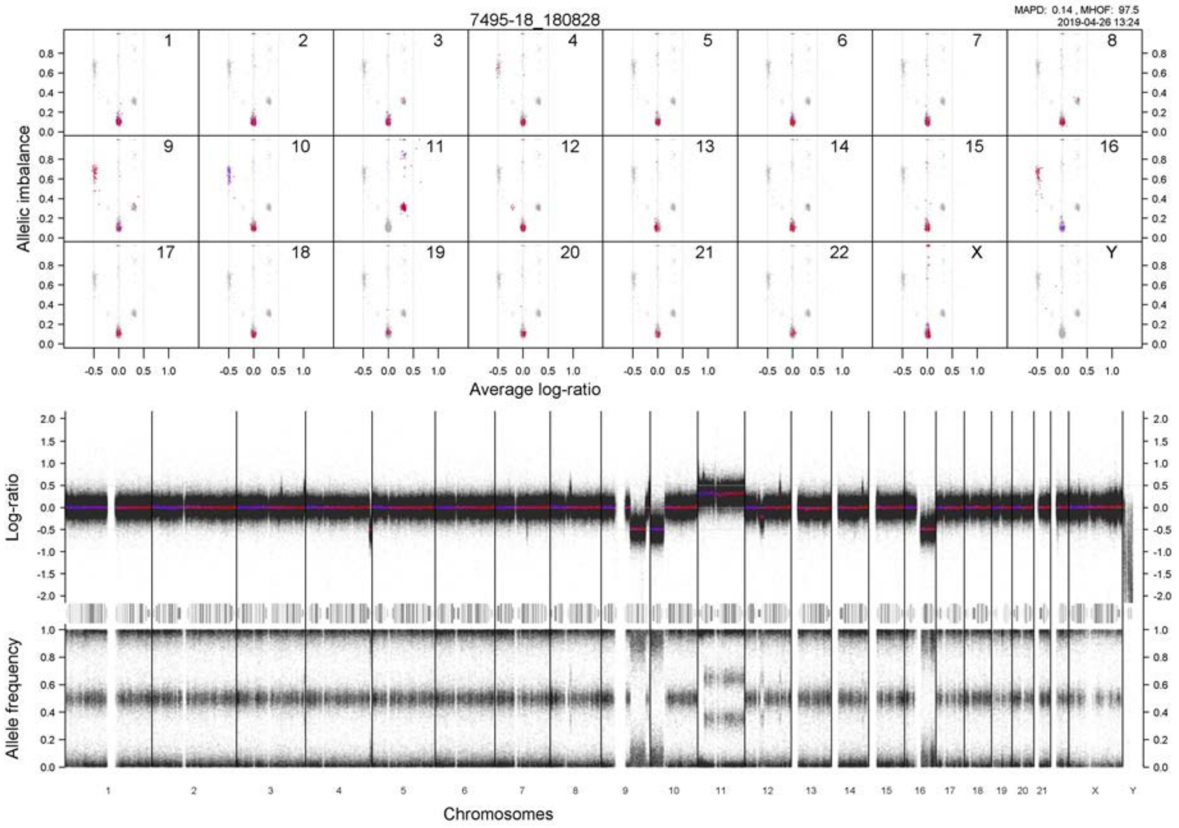

Surgical specimen

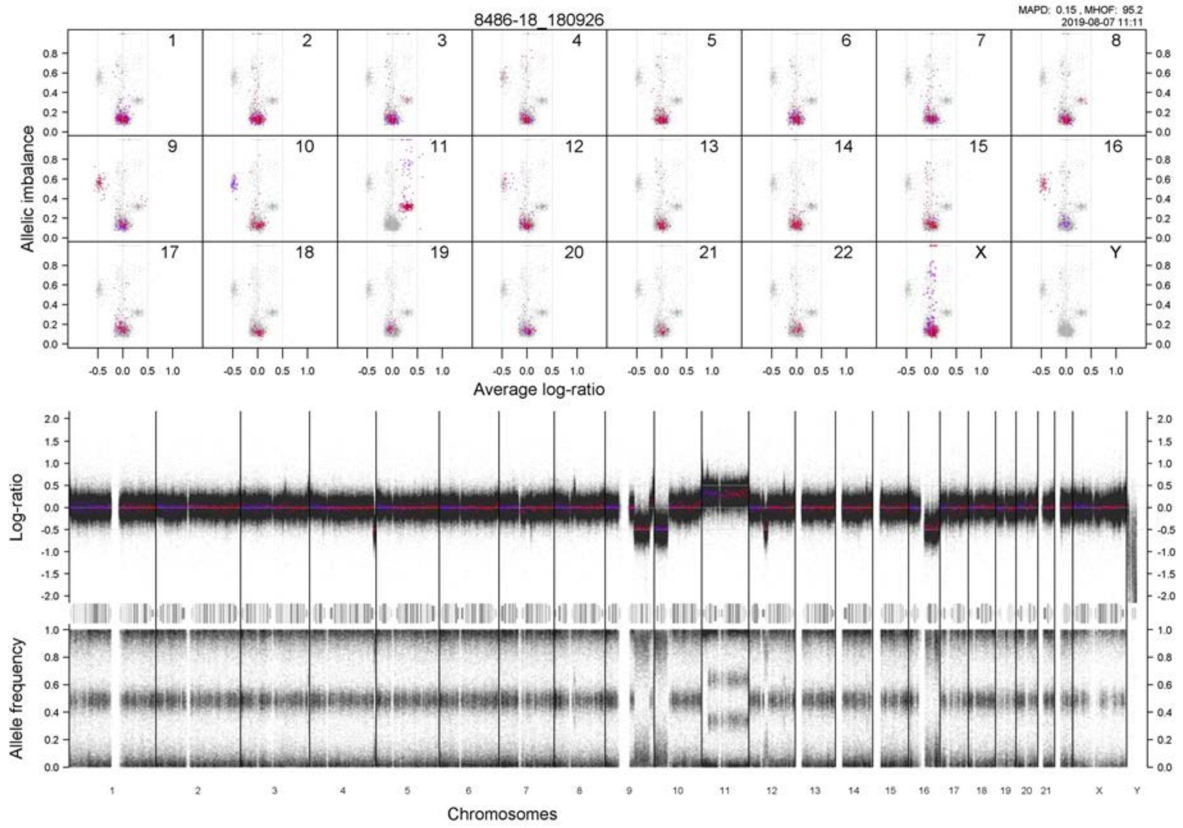

Case 146 (CNB) Medullary renal carcinoma/ rhabdoid tumor

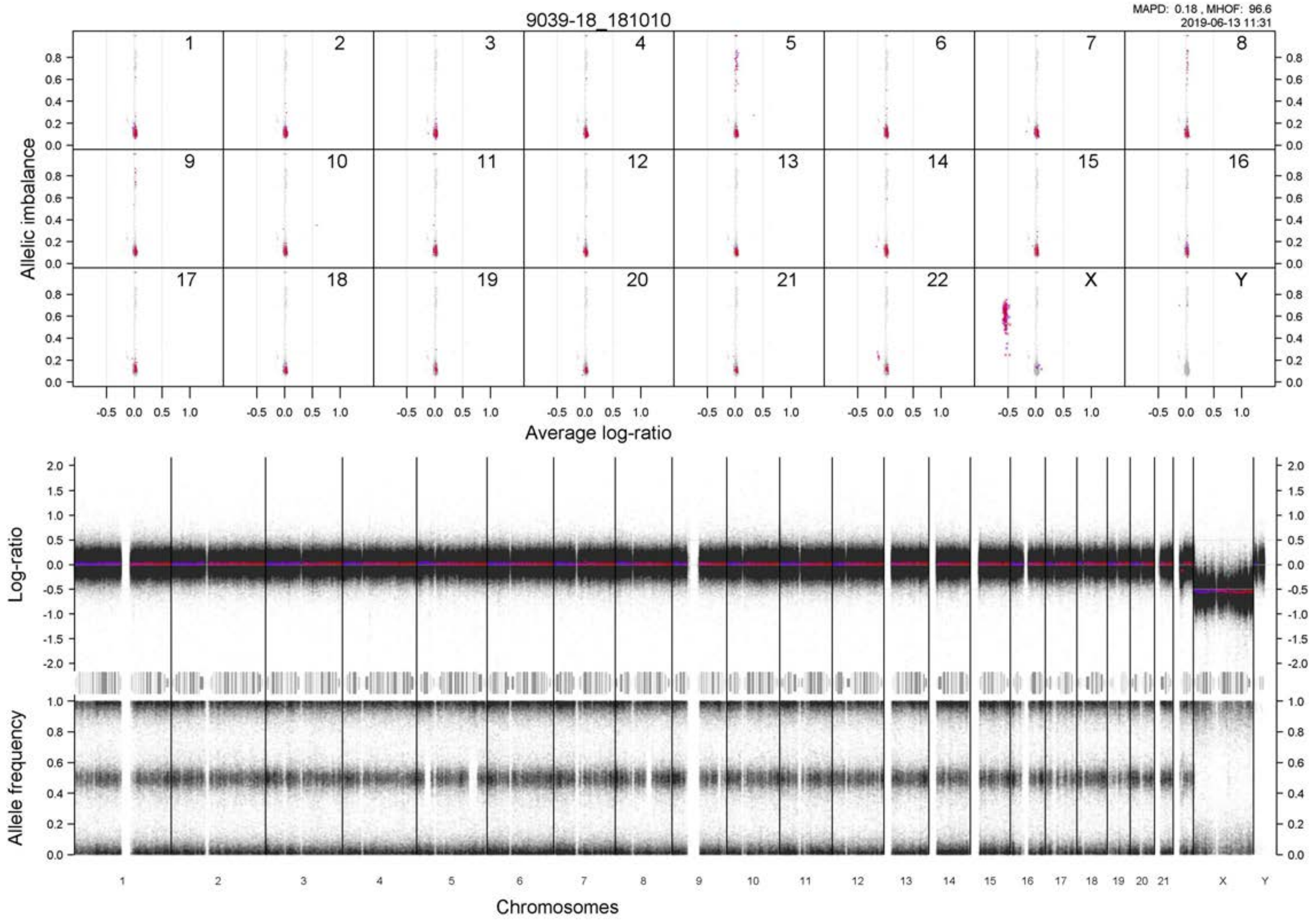

Case 148

UPS

CNB

Surgical specimen

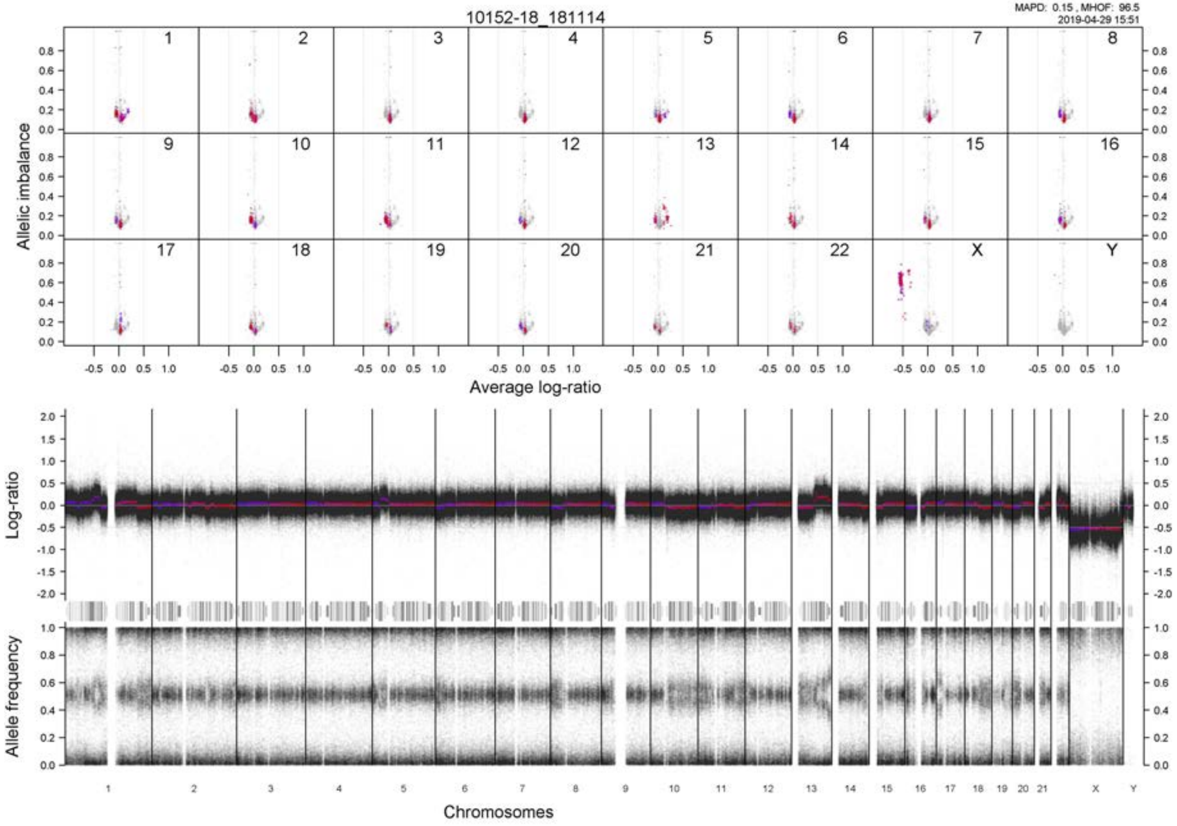

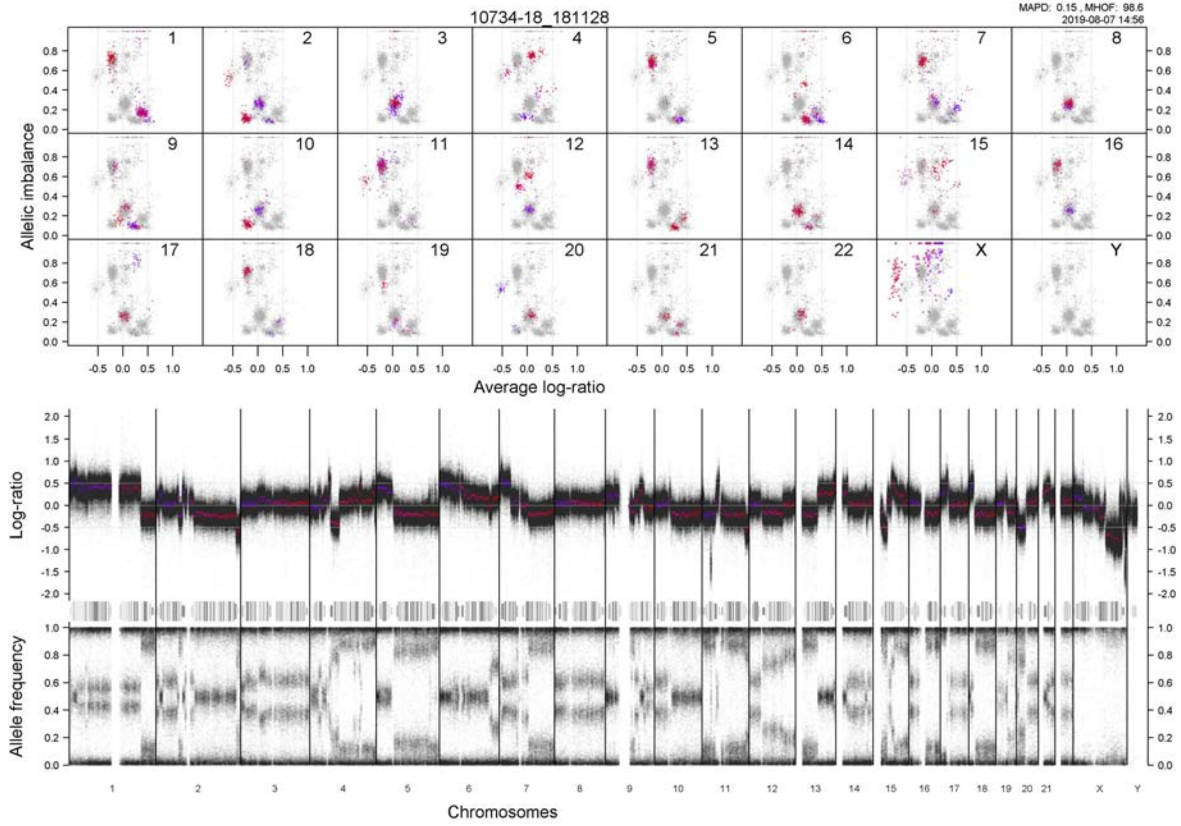

Case 149

Malignant sarcomatous tumor

CNB

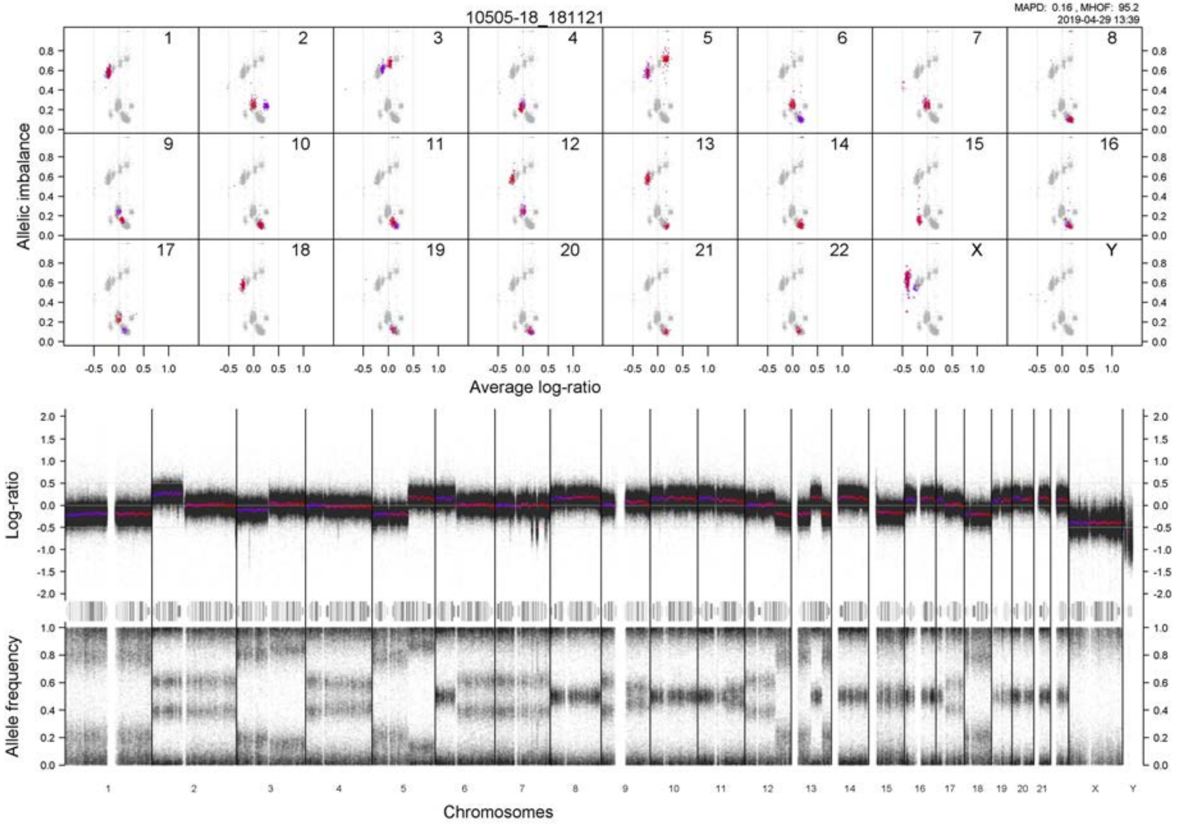

Surgical specimen

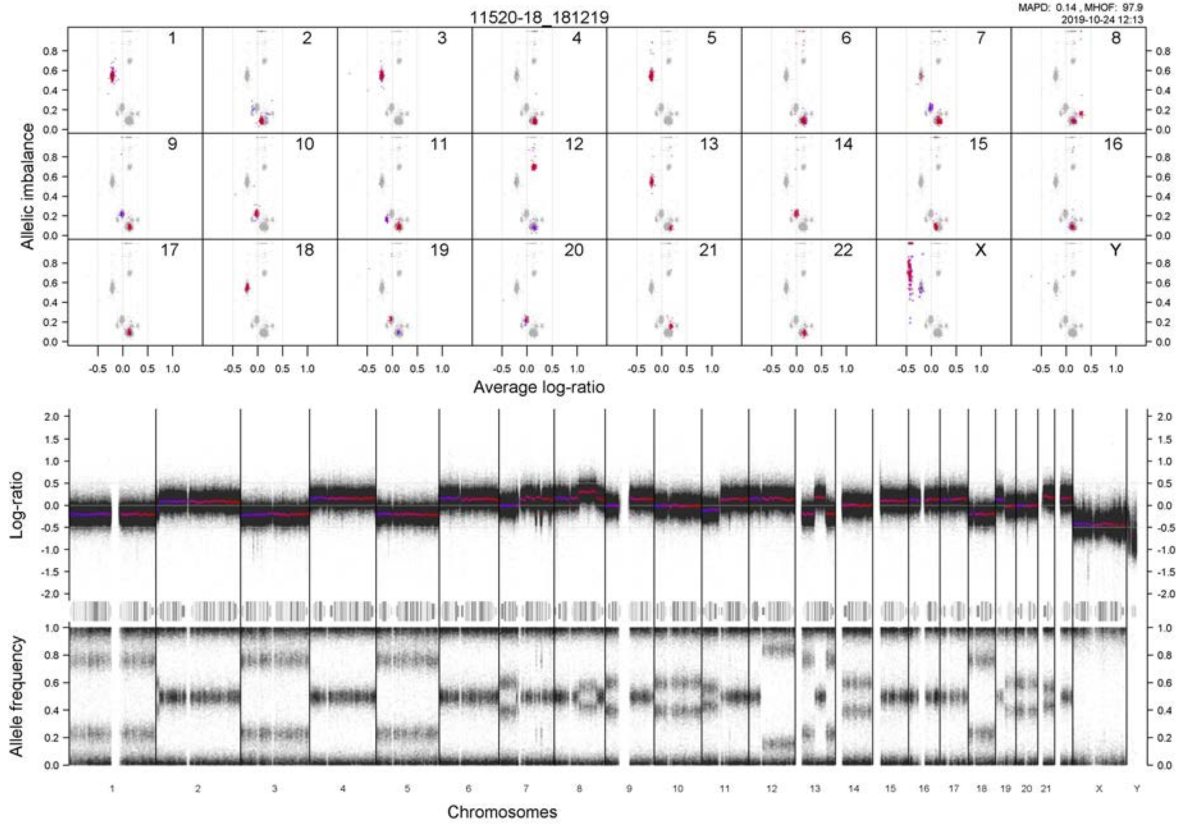

Case 150 (CNB)

Perineurioma

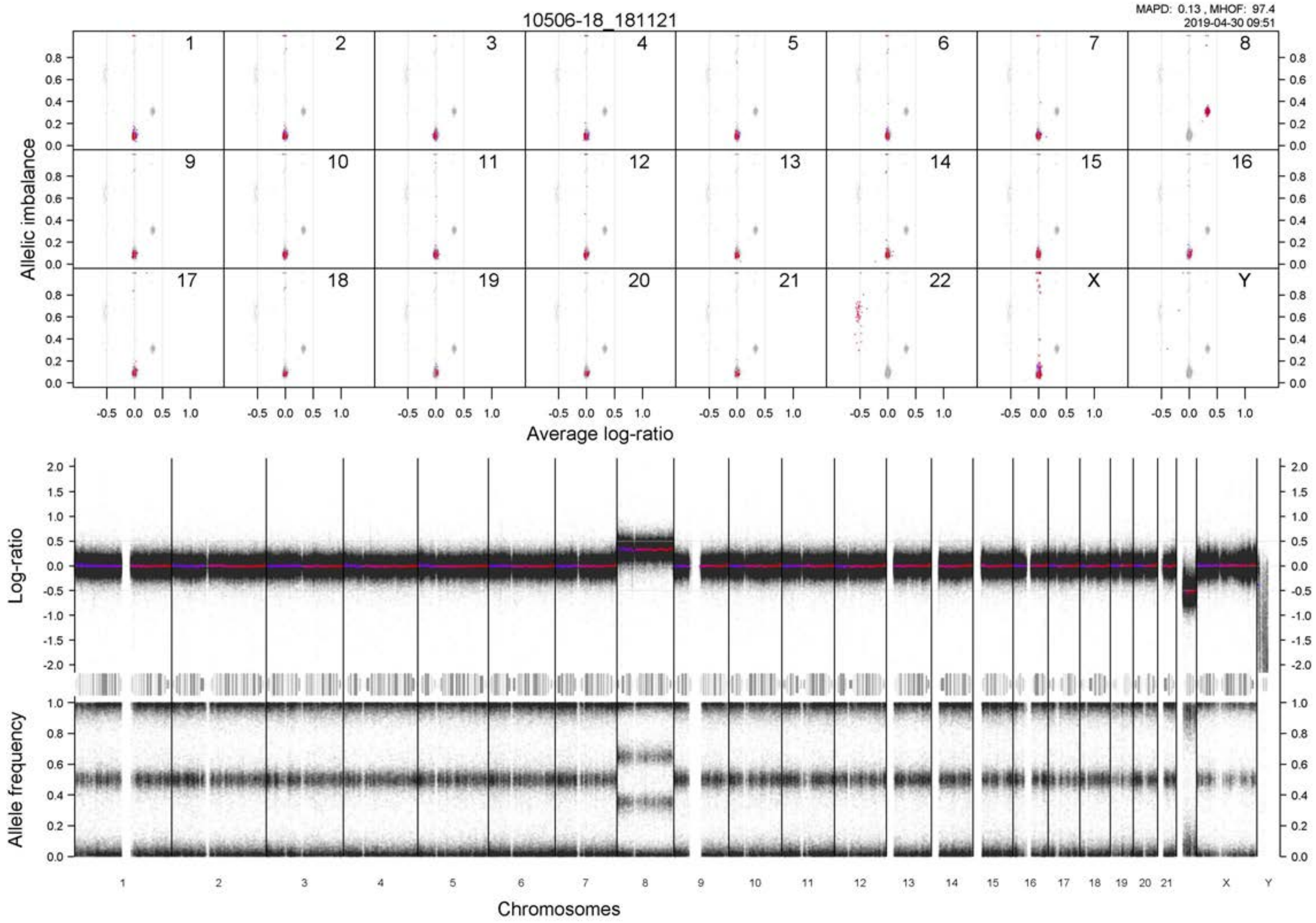

Case 151 (CNB) Desmoid fibromatosis

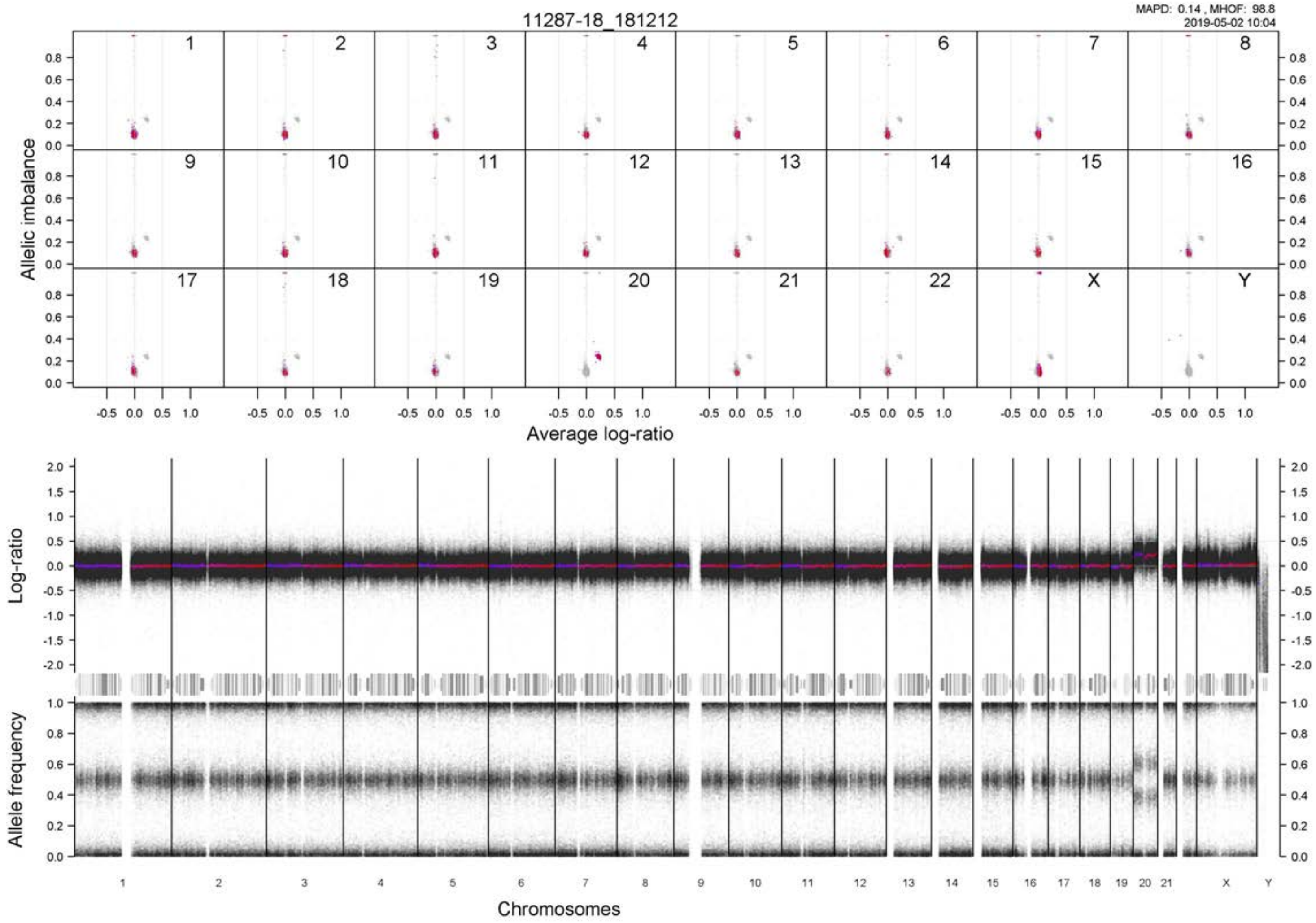

Case 156 (CNB)

Leiomyosarcoma

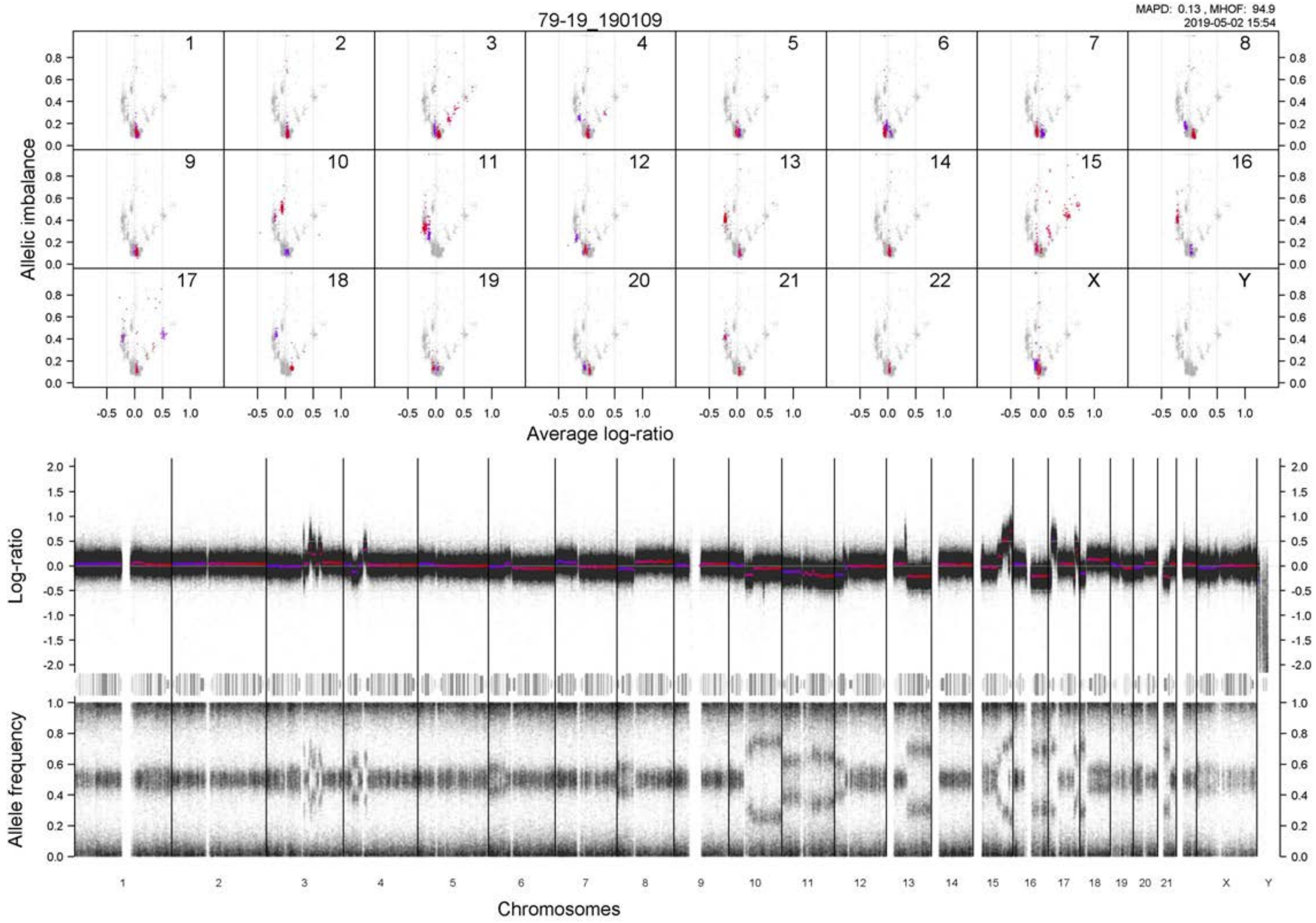

Case 160 Epithelioid sarcoma

CNB

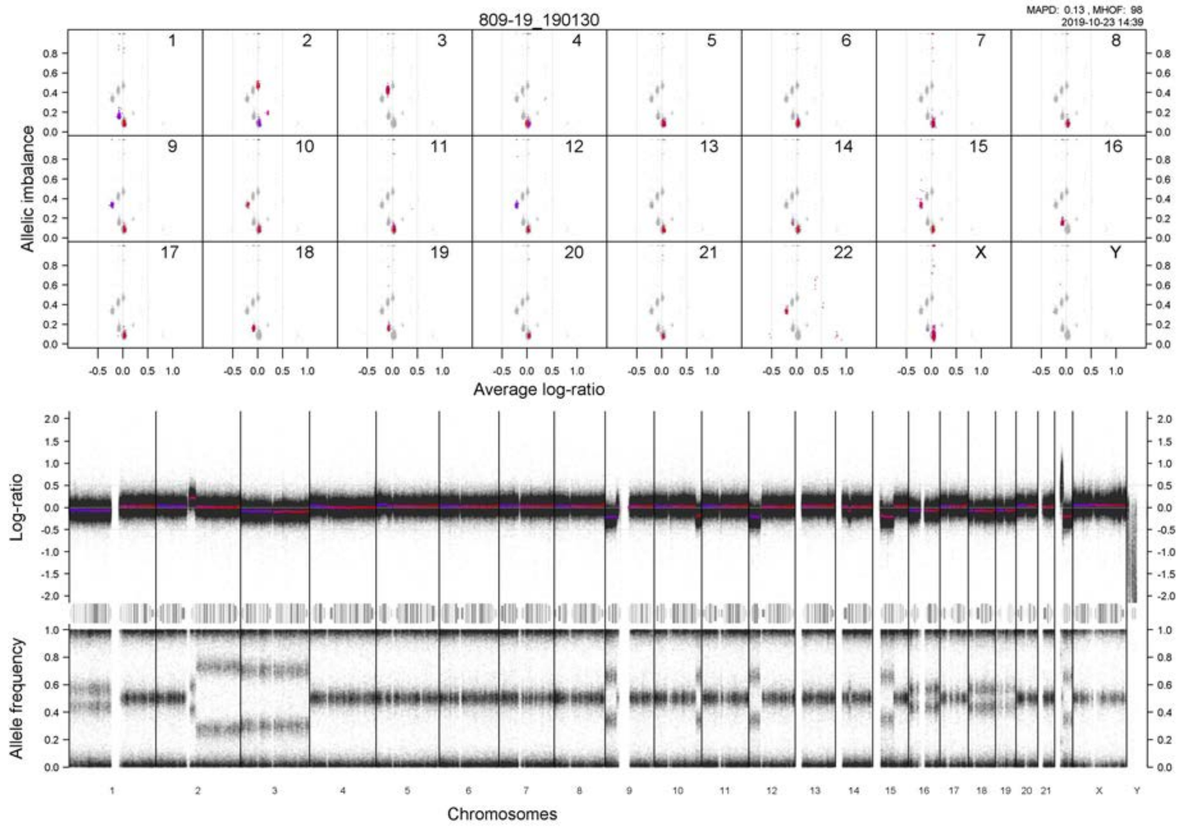

Surgical specimen

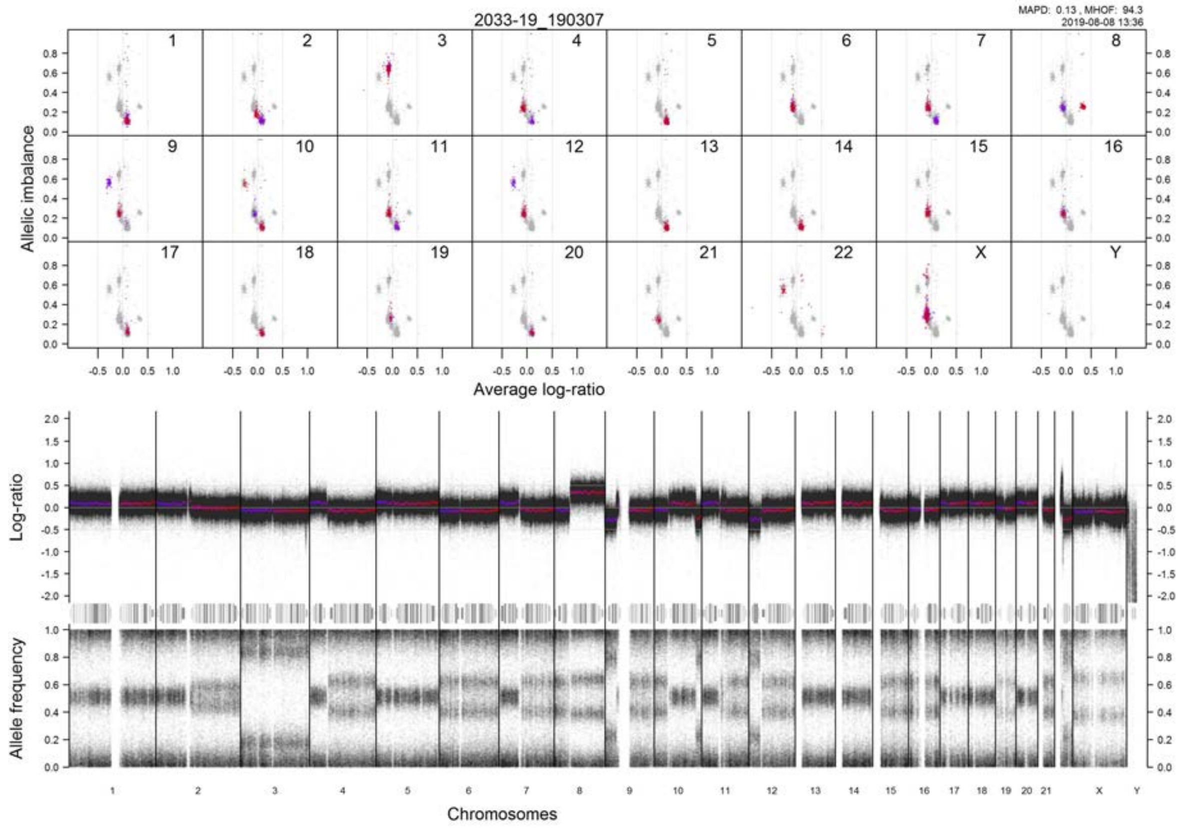

Case 162 (CNB) UPS

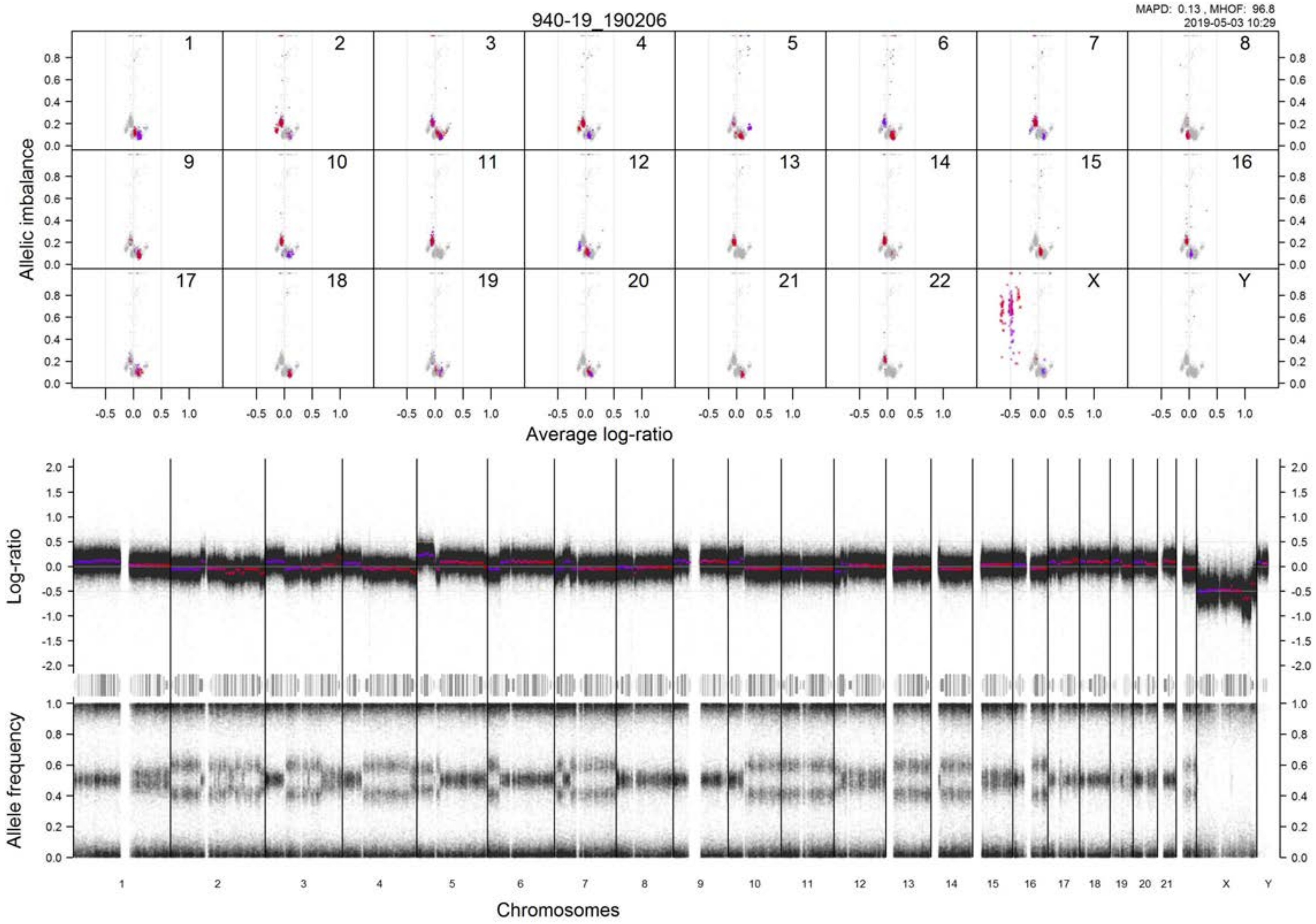

Case 163 (CNB) Giant cell tumor of bone

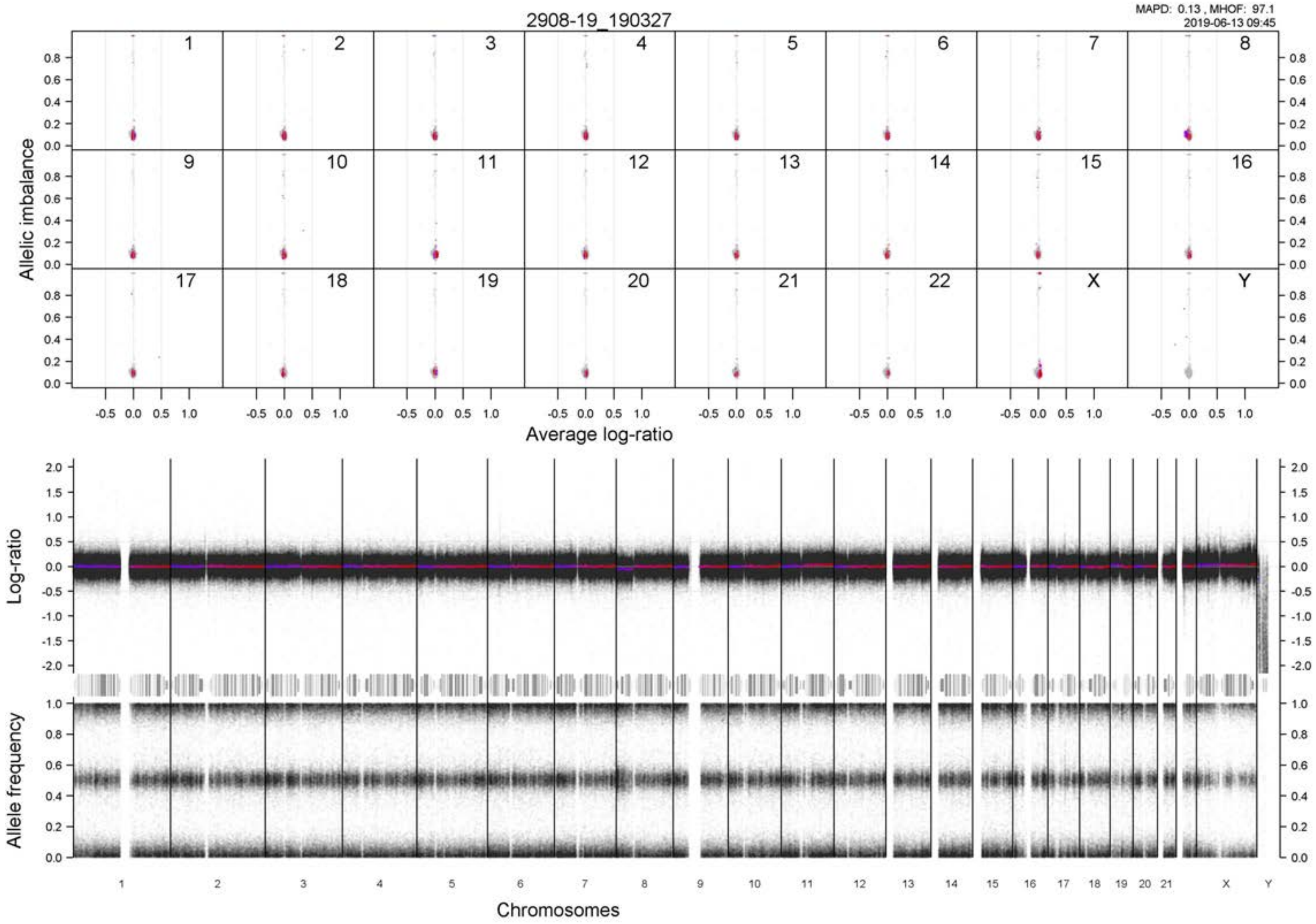

Case 167 (CNB)

Giant cell tumor of bone

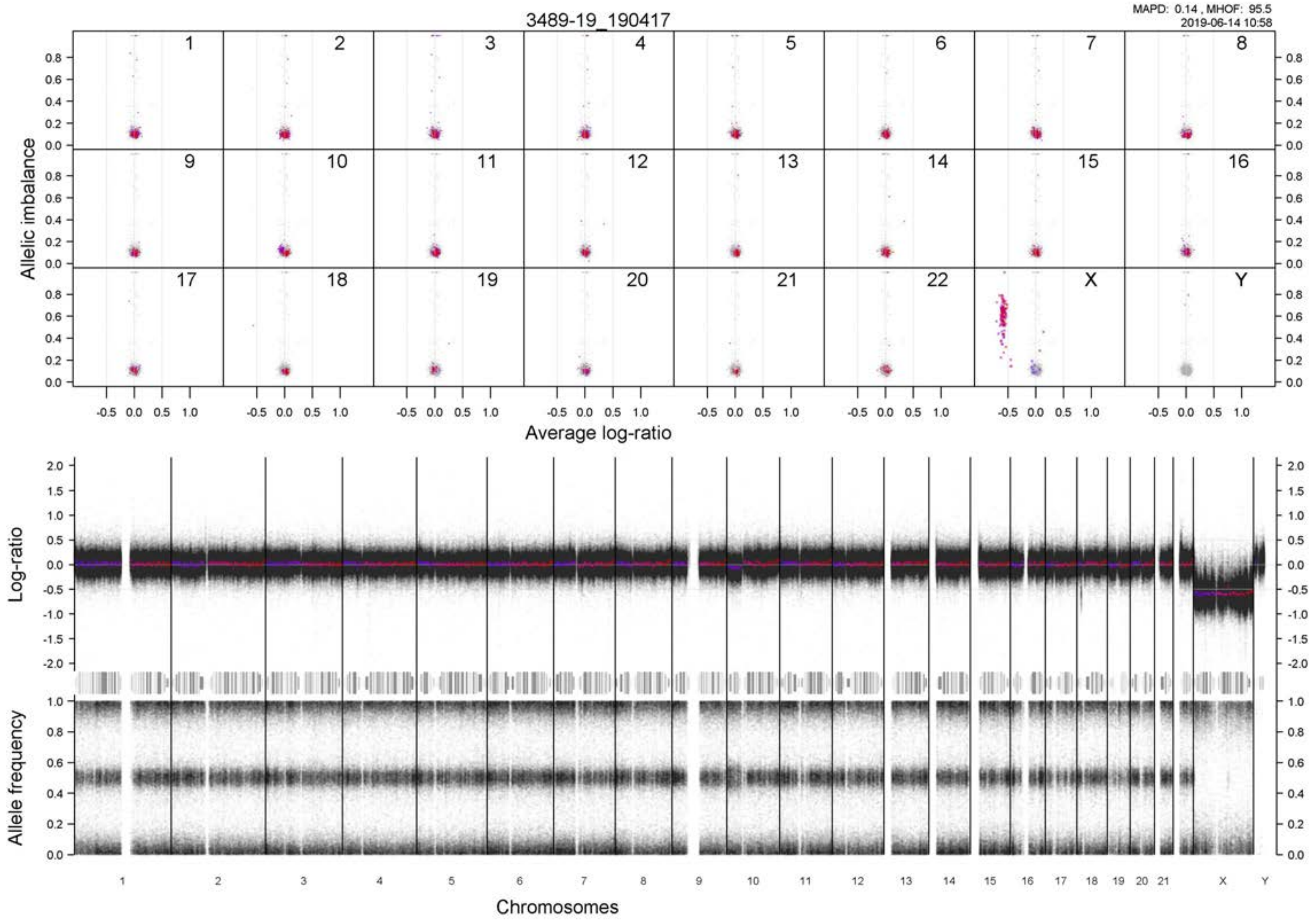

Case 168 (CNB)

Myxoid tumor, UMP

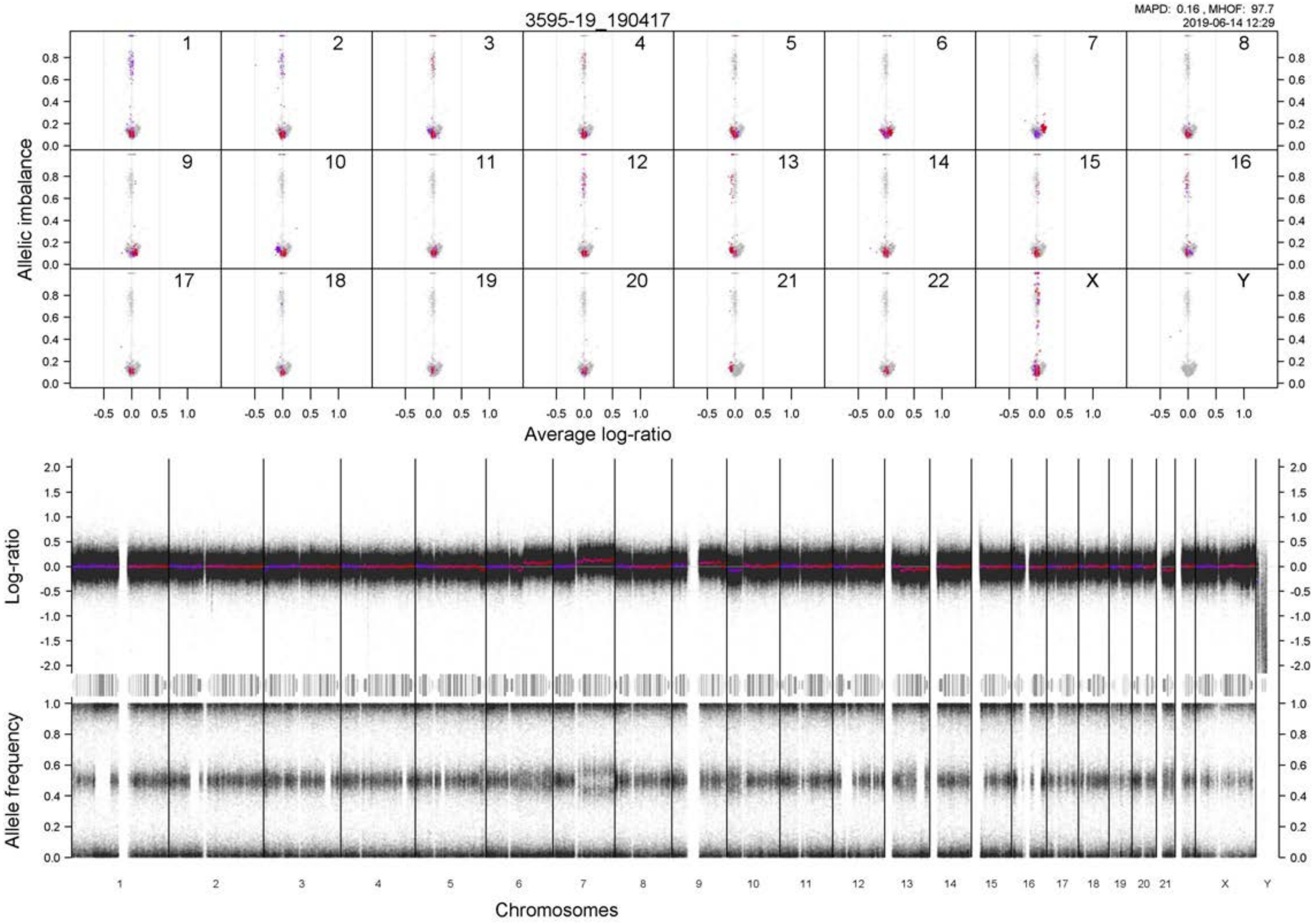

Supplement: Supplementary file 2 — Supplementary Figure 1 [file 41374_2022_770_MOESM2_ESM.pdf]
